# Identification of PRDX5 as A Target for The Treatment of Castration‐Resistant Prostate Cancer

**DOI:** 10.1002/advs.202304939

**Published:** 2023-12-20

**Authors:** Rong Wang, Yuanyuan Mi, Jiang Ni, Yang Wang, Lingwen Ding, Xuebin Ran, Qiaoyang Sun, Soo Yong Tan, H Phillip Koeffler, Ninghan Feng, Yong Q Chen

**Affiliations:** ^1^ Jiangnan University Medical Center Jiangnan University Wuxi 214002 China; ^2^ Affiliated Hospital Jiangnan University Wuxi 214122 China; ^3^ Wuxi School of Medicine Jiangnan University Wuxi 214122 China; ^4^ Department of Pathology Yong Loo Lin School of Medicine National University of Singapore Singapore 117597 Singapore; ^5^ Cancer Science Institute of Singapore National University of Singapore Singapore 117599 Singapore; ^6^ Department of Hematology Singapore General Hospital Singapore 169608 Singapore; ^7^ Division of Hematology/Oncology Cedars‐Sinai Medical Center UCLA School of Medicine Los Angeles California 90048 USA

**Keywords:** castration‐resistant prostate cancer, drug‐tolerant persister, peroxiredoxin 5, polaprezinc, stachyose

## Abstract

Treatment of castration‐resistant prostate cancer (CRPC) is a long‐standing clinical challenge. Traditionally, CRPC drugs work by either reducing dihydrotestosterone biosynthesis or blocking androgen receptor (AR) signaling. Here it is demonstrated that AR inhibitor treatment gives rise to a drug‐tolerant persister (DTP) state. The thioredoxin/peroxiredoxin pathway is up‐regulated in DTP cells. Peroxiredoxin 5 (PRDX5) promotes AR inhibitor resistance and CRPC development. Inhibition of PRDX5 suppresses DTP cell proliferation in culture, dampens CRPC development in animal models, and stabilizes PSA progression and metastatic lesions in patients. Therefore, the study provides a novel mechanism and potential target for the management of castration‐resistant prostate cancer.

## Graphical abstract

Peroxiredoxin 5 (PRDX5) promotes androgen receptor (AR) inhibitor resistance and castration‐resistant prostate cancer (CRPC) development. Inhibition of PRDX5 suppresses drug‐tolerant persister (DTP) cell proliferation in culture, dampens CRPC development in animal models, and stabilizes PSA progression and metastatic lesions in patients with the repurposed drug, polaprezinc. Therefore, the study provides a novel mechanism and potential target for the management of CRPC.

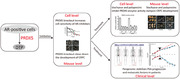

## 1 Introduction

Prostate cancer (PCa) is a major disease that affects 14.1% male population worldwide with a 6.8% mortality rate in 2020 (https://gco.iarc.fr/). Dr. Charles Huggins’ groundbreaking discovery in 1941 laid the foundation for androgen deprivation therapy (ADT) to control the spread of advanced prostate cancer.^[^ ^1^ ^]^ Despite the initial favorable response, nearly all patients progress to castration‐resistant prostate cancer (CRPC) and subsequently succumb to the disease within 1–3 years.^[^ ^2^ ^]^

ADT is accomplished primarily through two approaches: namely androgen reduction such as physical castration^[^ ^1^ ^]^ and chemical castration with gonadotropin‐releasing hormone^[^ ^3^ ^]^ or abiraterone^[^ ^4^ ^]^; androgen receptor (AR) blockade with drugs such as flutamide,^[^ ^5^ ^]^ enzalutamide^[^ ^6^ ^]^ or darolutamide.^[^ ^7^ ^]^ Significant efforts have been made over the last 80 years; however, the castration resistance conundrum remains unresolved.

Drug‐tolerant persister (DTP) state was reported a decade ago.^[^ ^8^ ^]^ Persister cells have enhanced drug resistance, do not carry genetic mutations, ≈20% of which proliferate as drug‐tolerant expanded persister (DTEP) cells, and are phenotypically reversible after drug withdrawal.^[^ ^8^ ^]^ DTP cell populations have been observed in many cancer types, e.g., non‐small‐cell lung cancer, melanoma, colorectal cancer, breast cancer, and gastric cancer.^[^ ^9^ ^]^ Persister state has since been reported as a main form of cancer cell resistance to chemotherapeutic drugs.^[^ ^10^ ^]^ It is unclear, however, whether AR inhibitor‐resistant prostate DTP cells exist.

In the present study, we demonstrate that AR‐blocking drug‐resistant DTP cells play a critical role in the development of CRPC through the peroxiredoxin 5 (PRDX5) pathway. PRDX5 promotes AR inhibitor resistance in vitro and CRPC development in vivo. PRDX5 inhibitory drug suppresses CRPC tumor growth in mice and stabilizes CRPC tumors in patients. Our study delineates a novel mechanism of prostate cancer castration resistance.

## 2 Results

### 2.1 AR Inhibitor Treatment gives rise to DTP and DTEP Cells

**Figure 1:**
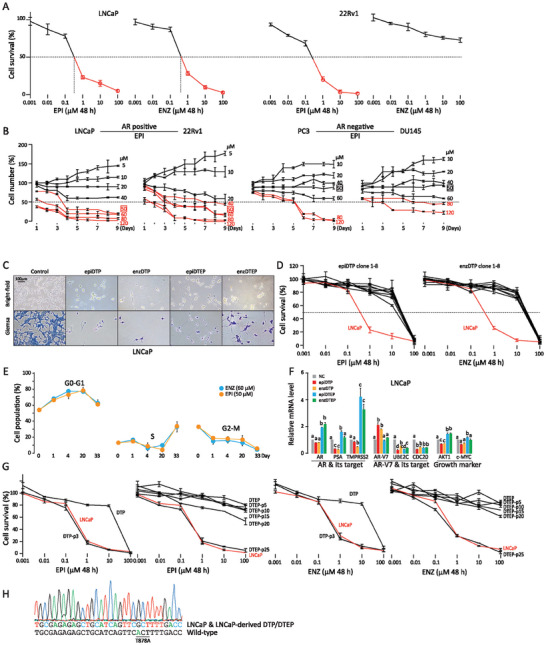
Characterization of AR inhibitor tolerant persister (DTP) and extended persister (DTEP) cells A) LNCaP and 22Rv1 PCa cells were treated with different concentrations (0.001‐100 µM) of AR inhibitor EPI001 (EPI) or enzalutamide (ENZ) for 48 h. IC_50_ values were calculated. Data were expressed as a percentage of viable cells relative to untreated controls as measured by CCK‐8 assay and expressed as mean ± std of triplicates. B) AR‐positive LNCaP, 22Rv1 PCa cells, and AR‐negative PC3, DU145 PCa cells were treated with different concentrations (5‐120 µM) of EPI for 9 days. Less than 50% of AR‐positive cells, but the majority of AR‐negative cells, survived at 50 µM of EPI. Data were expressed as percentages relative to the number of seeded cells (1 × 10^6^) as measured by hemocytometer and as mean ± std of triplicates. C) light microscopic images of LNCaP cells exposed to EPI (epiDTP) and ENZ (enzDTP) for 9 days or to EPI (epiDETP) and ENZ (enzDETP) for 33 days. Scale bars = 100 µm. D) Resistance of epiDTP and enzDTP clones to corresponding drugs. Data were expressed as a percentage of viable cells relative to untreated LNCaP controls as measured by CCK‐8 assay and expressed as mean ± std of triplicates. E) Cell cycle distribution at different days (0, 1, 4, 20, 33) of drug treatment as measured by FACS analysis. G0/1 arrest was seen in the early phase and the release of the arrest in the later phase of treatment. Data are expressed as mean ± std. of triplicates. F) Relative mRNA expression of AR, AR targets (PSA, TMPRS2), AR‐V7, AR‐Vs targets (UBE2C, CDC20), and growth markers (AKT1, c‐MYC) as measured by qRT‐PCR. Data are expressed as mean ± std. of triplicates. One‐way ANOVA with the Turkey test was performed. *p*<0.05 was considered significant and indicated by different letters. G) Reversal of the resistant phenotype after drug withdrawal. LNCaP DTP and DTEP cells became sensitive to drugs after 3 and 25 passages, respectively. Data were expressed as a percentage of viable cells relative to untreated LNCaP controls as measured by CCK‐8 assay and expressed as mean ± std of triplicates. H) DNA sequence analysis of the AR gene in LNCaP and LNCaP DTP and DTEP cells. LNCaP has an AR T878A mutation. No new AR mutation was seen in LNCaP DTP and DTEP cells.

Tumor cells are killed after a 9‐day exposure to a drug concentration 100‐fold greater than the IC_50_ value, and surviving cells are referred to as drug‐tolerant persisters (DTP). Although DTP cells are largely quiescent, approximately 20% of them eventually resume normal proliferation in the presence of the drug, yielding colonies of cells referred to as drug‐tolerant expanded persisters (DTEP), which can be propagated in the presence of the drug indefinitely.^[^ ^8^ ^]^ It is unclear whether there are AR inhibitor‐resistant DTP and DTEP cells. We treated AR‐positive LNCaP and 22Rv1 human PCa cell lines with EPI001 (EPI) and enzalutamide (ENZ), IC_50_ of EPI for LNCaP and 22Rv1 was 0.48 and 0.38 µM, IC_50_ of ENZ for LNCaP was 0.57 µM but 22Rv1 is resistant to ENZ (**Figure** **1**A). To obtain DTP and DTEP cells, we treated AR‐positive and AR‐negative cell lines with inhibitors at concentrations 100‐fold of IC_50_. Less than 50% of AR‐positive cells, but most AR‐negative cells, survived at 50 µM of EPI or 60 µM of ENZ (Figure 1B; Figure S1A, Supporting Information). Thus, 50 µM of EPI and 60 µM of ENZ could differentiate AR‐positive and negative cells.

We then treated LNCaP and 22Rv1 cells with 50 µM of EPI or 60 µM of ENZ for short‐term (9 days) or long‐term (33 days), respectively, and fractions of viable cells were detected (Figure 1C; Figure S1B, Supporting Information). LNCaP‐derived DTPs and DTEPs were about 100‐fold more AR inhibitor‐resistant than parental LNCaP cells (Figure 1D; Figure S8D, Supporting Information). Cells that survived the short‐term treatment had slower proliferation rates due to G1/0 cell cycle arrest (Figure 1E; Figure S1C, Supporting Information) and lower AR‐related activities (Figure 1F). Cells that survived the long‐term treatment regained some proliferative capacities (Figure 1E; Figure S1C, Supporting Information) and AR activities (Figure 1F). Although they express different levels of AR and/or AR mutant, a significant difference between 22Rv1 and LNCaP cells is that 22Rv1 is inherently ENZ‐resistant whereas LNCaP develops ENZ‐resistance after being exposed to ENZ in culture,^[^ ^11^ ^]^ which is in agreement with our results (Figure 1A).

DTP cells should have the characteristics of recovering drug sensitivity after AR inhibitor withdrawal. Indeed, we noticed that DTP or DTEP cells became sensitive to AR inhibitors again after 3 or 25 passages, respectively (Figure 1G). Expression of epithelial‐mesenchymal transition markers CDH1 and VIM was also reversible after drug withdrawal (Figure S1D, Supporting Information). There was no additional AR mutation in treated cells compared to LNCaP and 22Rv1 by sequencing analysis (Figure 1H). These results suggest that short‐term and long‐term AR inhibitor‐treated cells have phenotypes of DTP and DTEP cells.

### 2.2 Drugable Targets are Selected by Proteomics and Drug Database Cross‐Searching

**Figure 2:**
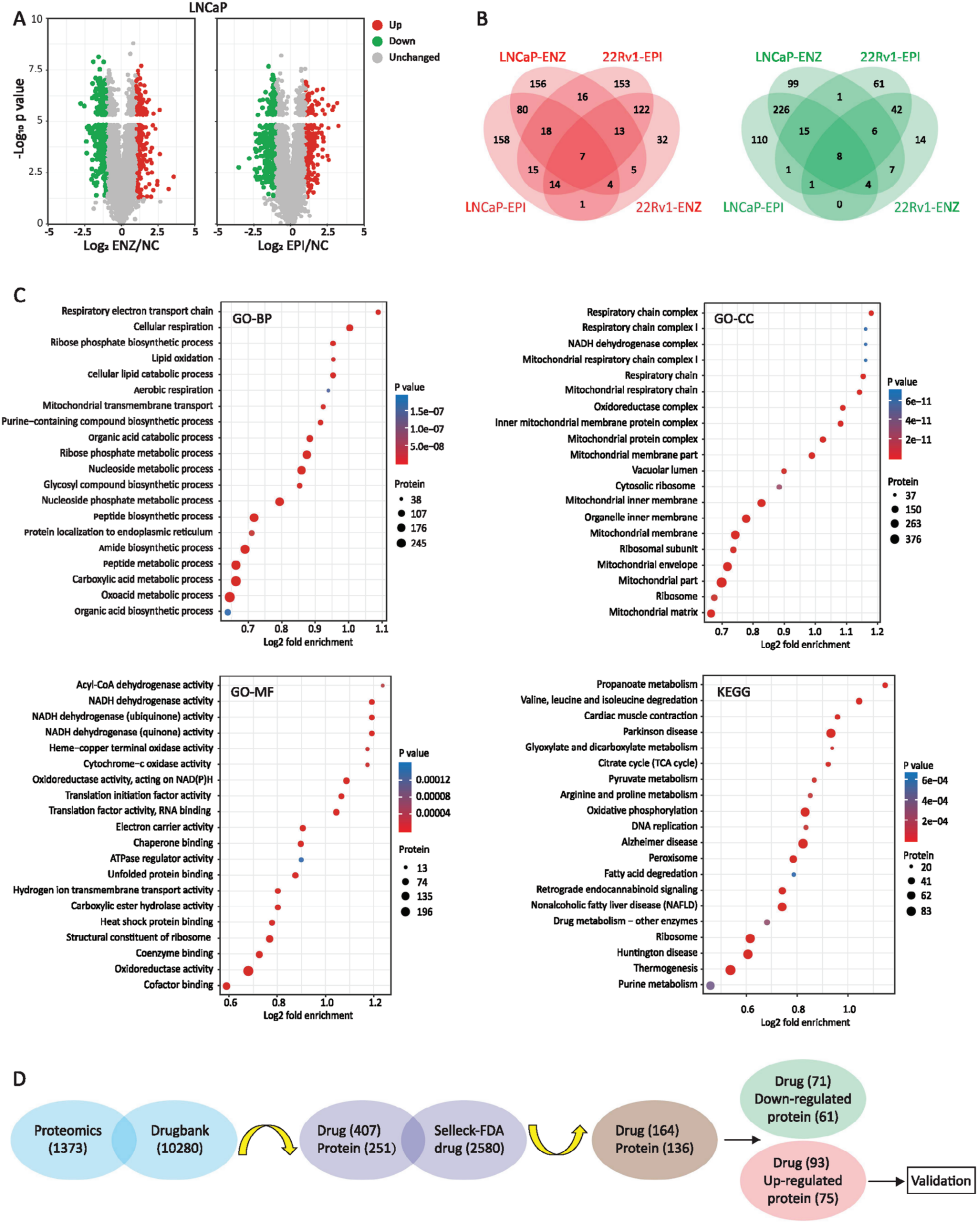
Identification of drugable targets by proteomics and database cross‐searching A) Volcano plots show the differentially expressed proteins (DEP) in LNCaP epiDTP and enzDTP cells compared to untreated controls. The x‐axis is the log2 value of the relative protein level, and the y‐axis is ‐log10 of the *p*‐value. The red dots represent significantly up‐regulated DEPs, the green dots represent significantly down‐regulated DEPs, and the grey dots indicate proteins that were not significantly differentially expressed. B) Venn diagrams show the numbers of DEPs in 4 different comparison groups. Red represents the number of significantly up‐regulated DEPs, and green represents the number of significantly down‐regulated DEPs. C) Gene ontology (GO: biological process, cellular component, and molecular function) enrichment bubble plot of significant DEPs. KEGG pathway enrichment bubble plot of significant DEPs. The size and color of the bubbles reflect the DEP numbers and p‐values, respectively. D) Flow chart depicts proteomics and database cross‐searching process.

To characterize further DTP cells, we identified differentially expressed proteins between persister and parental cells through proteomics. 662 differentially expressed proteins in LNCaP epiDTP cells (297 up‐ and 365 down‐regulated), 665 in LNCaP enzDTP cells (299 up‐ and 366 down‐regulated), 493 in 22Rv1 epiDTP (358 up‐ and 135 down‐regulated), and 280 in 22Rv1 enzDTP cells (198 up‐ and 82 down‐regulated) were found (**Figure** **2**A; Table S1–S4, Supporting Information). There were 7 up‐regulated and 8 down‐regulated proteins common among four treatments (Figure 2B; Data 1–2, Supporting Information).

The differentially expressed proteins were subjected to GO and KEGG enrichment analysis, we found significant GO enrichment in respiratory and oxidative processes as well as KEGG enrichment in metabolism and disease‐related signaling pathways (Figure 2C), suggesting oxidative response may be involved in the development of AR inhibitor resistant persister cells.

To identify potential therapeutic target(s), we cross‐searched 1373 differentially expressed proteins in the Drugbank (https://go.drugbank.com/) and obtained 251 target proteins with corresponding 407 drugs (Figure 2D; Data 3, Supporting Information). We then matched the resulting 407 candidates with 2580 FDA‐approved drugs sold by Selleck (https://www.selleckchem.com/) and selected 136 targets with corresponding 164 drugs (Figure 2D; Data 4, Supporting Information). Lastly, we experimentally validated 75 up‐regulated proteins with corresponding 93 drugs (Figure 2D; Table S5, Supporting Information).

### 2.3 The Thioredoxin/Peroxiredoxin Pathway is Up‐Regulated in DTP Cells

**Figure 3:**
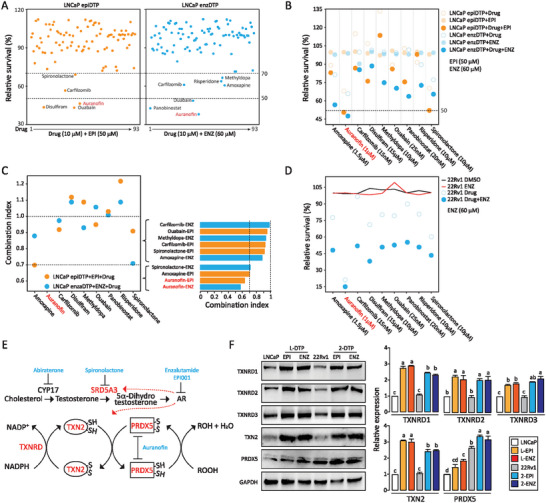
Upregulation of the thioredoxin/peroxiredoxin pathway in DTP cells A) LNCaP epiDTP or enzDTP cells were treated for 48 h with EPI001 (EPI 50 µM) or enzalutamide (ENZ 60 µM), respectively, plus each ninety‐three drugs (10 µM) identified in Figure 2. Experiments identified 9 drugs that reduced cell survival below 70%. Each data point represents the average of three independent experiments and is expressed as a percentage of viable cells relative to untreated controls as measured by CCK‐8 assay. Standard deviations are less than 5%. B) The highest concentration that does not affect LNCaP cell viability (IC_0_) was determined for the above 9 drugs. Subsequently, LNCaP epiDTP or enzDTP cells were treated with each drug (IC_0_) with or without EPI001 (EPI 50 µM) or enzalutamide (ENZ 60 µM) for 48 h. Additive or synergistic effects were seen for all nine drugs. Each data point represents the average of three independent experiments and is expressed as a percentage of viable cells relative to untreated controls as measured by CCK‐8 assay. Standard deviations are less than 5%. C) Combination indexes (CI) of nine drugs were measured. A CI of less than, equal to, and more than 1 indicates synergy, additivity, and antagonism, respectively. D) 22Rv1 cells that are resistant to ENZ were treated for 48 h with ENZ (60 µM), each of the 9 drugs identified above, or in combinations. Results indicated that all 9 drugs either alone or in combination with ENZ could reduce cell survival. Each data point represents the average of three independent experiments and is expressed as a percentage of viable cells relative to untreated controls as measured by CCK‐8 assay. Standard deviations are less than 5%. E) Diagram portrays the thioredoxin/peroxiredoxin pathway and up‐regulation of key proteins (red color) in DTP cells. F: Confirmation of proteomics results (TXNRD1, TXNRD2, TXNRD3, TXN2, and PRDX5) by Western blotting. GAPDH protein is used as the loading control. Data are expressed as mean ± std of triplicates. One‐way ANOVA with the Turkey test was performed. *p*<0.05 was considered significant and indicated by different letters.

Next, we measured the effect of the 93 above‐selected drugs on LNCaP DTP cells. We noticed that nine drugs, namely disulfiram, carfilzomib, ouabain, spironolactone, auranofin, panobinostat, risperidone, methyldopa, and amoxapine, reduced DTP cell viability greater than 30% and four drugs, i.e., disulfiram, ouabain, auranofin and panobinostat, reduced DTP cell viability greater than 50% (**Figure** **3**A). We then tested the effect of nine drugs in combination with AR inhibitors on LNCaP DTP cells and found auranofin was the most effective drug (Figure 3B). Auranofin plus AR inhibitors (EPI and ENZ) had the smallest combination index and hence the strongest synergistic effect (Figure 3C). Interestingly, auranofin alone or in combination with ENZ had a significant inhibitory effect on the ENZ‐resistant 22Rv1 cells (Figure 3D).

Auranofin is an inhibitor of thioredoxin reductase ^[^ ^12^ ^]^ and has also been reported to inhibit PRDX5 (https://go.drugbank.com/drugs/DB00995). Our proteomics data indicate that thioredoxin reductase (TXNRD), thioredoxin 2 (TXN2), and peroxiredoxin 5 (PRDX5) were up‐regulated in LNCaP‐ derived DTPs or EPI/ENZ treated 22Rv1 cells (Figure 3E). We confirmed the outcome by Western blot analysis. In addition, 22Rv1 cells had much higher expression than LNCaP cells for PRDX5, which side confirmed that the resistance of 22Rv1 cells to ENZ led to high expression of PRDX5 (Figure 3F). The expression of PRDX5 is significantly increased in prostate cancer samples (PRAD) compared to normal tissues (Figure S2A, Supporting Information). PRDX5 overexpression occurs also in other types of cancers (Figure S2B, Supporting Information). These results together with GO/KEGG enrichment analyses suggest that the thioredoxin/peroxiredoxin pathway is up‐regulated in DTP cells, and PRDX5 is a potential therapeutic target.

### 2.4 PRDX5 Promotes AR Inhibitor Resistance and CRPC Development

**Figure 4:**
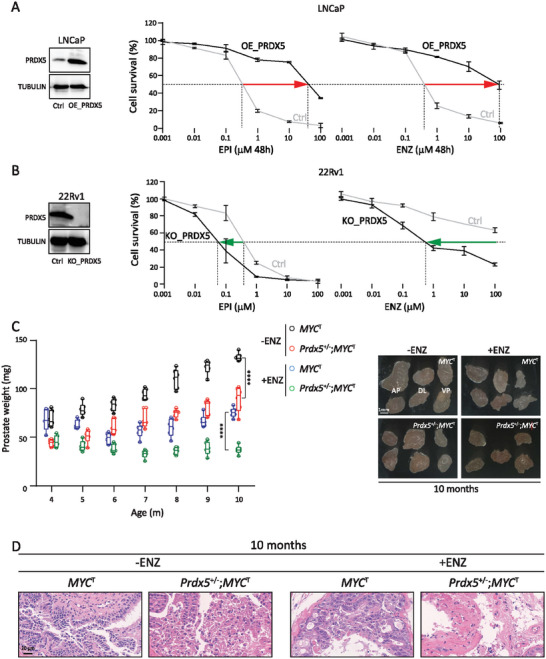
Key role of PRDX5 in AR inhibitor resistance and CRPC development A) PRDX5 overexpression (OE_PRDX5) enhanced LNCaP cell resistance to AR inhibitors (EPI and ENZ). B) PRDX5 knockout (KO_PRDX5) reduced 22Rv1 cell resistance to AR inhibitors (EPI and ENZ). The red and green arrows indicate an increase and decrease in IC_50_, respectively. Data were expressed as a percentage of viable cells relative to untreated LNCaP or 22Rv1 controls as measured by CCK‐8 assay and expressed as mean ± std of triplicates. C) *Prdx5* heterozygosity slowed down PCa progression in mice. Weight and photograph of the prostate are shown from *MYC* ^T^ and *Prdx5* ^+/−^;*MYC* ^T^ mice with or without ENZ treatment (6 mice per group). AP: anterior prostate lobe, DL: dorsolateral prostate lobe, VP: ventral prostate lobe. Student's *t*‐test was performed, and **** indicates *p*<0.0001. D) Representative images of prostate histopathology. Age, genotype, and treatment are labeled.

To substantiate the role of PRDX5 in PCa, we overexpressed *PRDX5* in LNCaP which has a relatively low endogenous PRDX5 level, and treated the cells with different concentrations of EPI/ENZ for 48 h. Overexpression of *PRDX5* significantly increased cell resistance to EPI and ENZ (**Figure** **4**A). We next deleted *PRDX5* from 22Rv1, which has a relatively high endogenous PRDX5 level and is enzalutamide‐resistant, by the CRISPR‐cas9 technique. Knockout of *PRDX5* sensitized cells to EPI and ENZ (Figure 4B). These in vitro data imply that PRDX5 plays a key role in PCa cell resistance to AR inhibitors.

To corroborate the role of PRDX5 in vivo, we obtained *Prdx5* total knockout mice (Figure S3AB). Deletion of *Prdx5* reduced the survival rate of homozygous mice (Figure S3C). Furthermore, we could not obtain *Prdx5* homozygous/*MYC* transgenic mice (Figure S3D). We, therefore, determined the impact of partial loss of *Prdx5* on the prostate in *MYC* transgenic mice. We noticed that *Prdx5* ^+/−^;*MYC* ^T^ mice, compared to *MYC* ^T^, had significantly delayed prostatic intraepithelial neoplasia (PIN)/tumor progression in terms of tissue weight (Figure 4C) and histopathology (Figure 4D). Furthermore, *Prdx5* ^+/−^;*MYC* ^T^ mice were slower in the emergence of ENZ resistance and significantly inhibited the growth of prostate tumors (Figure 4C). Our data demonstrated that PRDX5 is critical in the development of castration‐resistant prostate cancer (CRPC). It is noteworthy that *Prdx5* gene knockout alone did not affect the prostate weight or prostate histopathology (Figure S3E,F, Supporting Information).

### 2.5 PRDX5 Inhibitor Suppresses CRPC Tumor Growth

**Figure 5:**
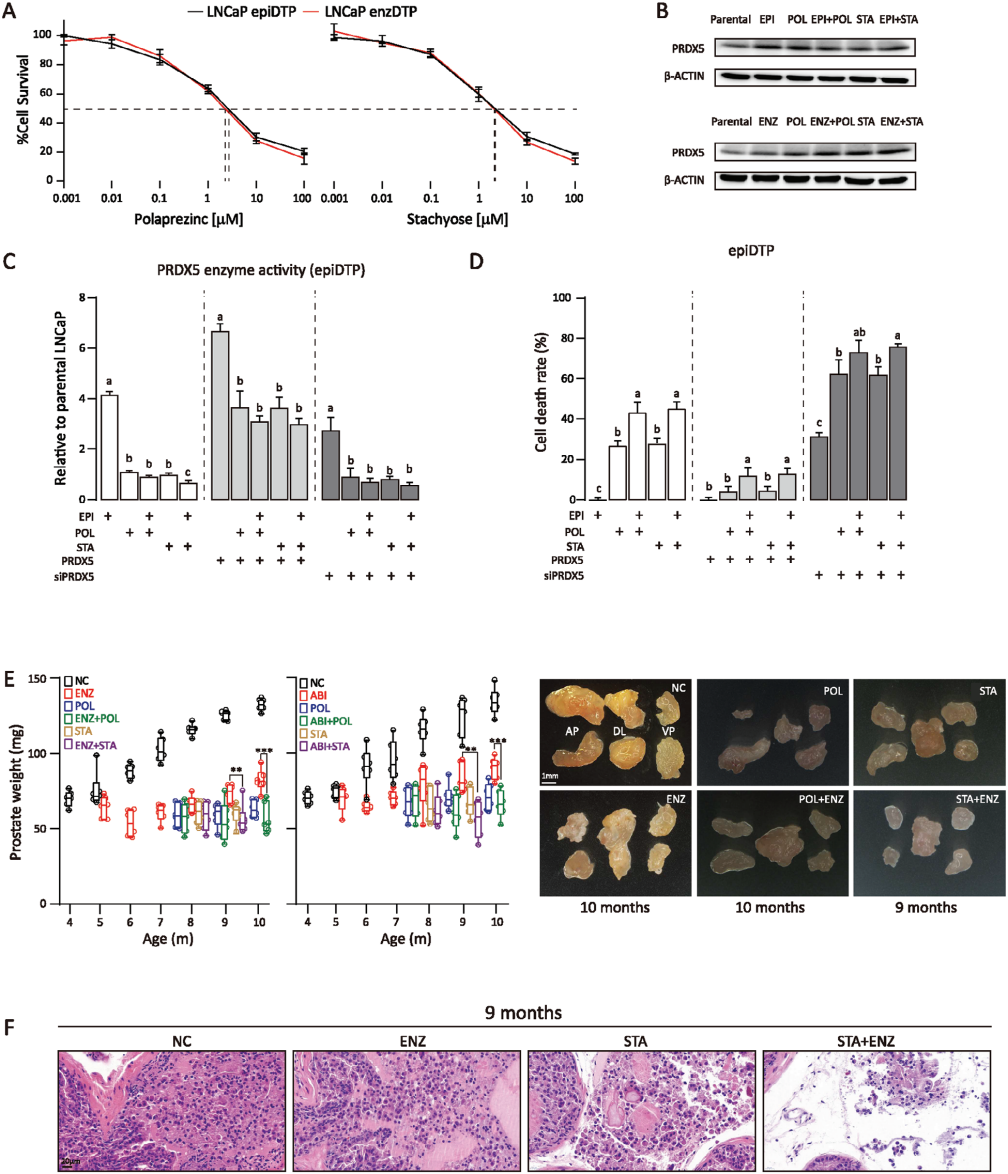
Suppression of CRPC tumor growth by PRDX5 inhibitors A) LNCaP epiDTP and enzDTP cell survivals were reduced by POL with IC_50_ of 4.71 µM and 4.15 µM, and by STA with IC_50_ of 4.08 µM and 3.82 µM, respectively. B) PRDX5 protein level was not changed by the treatment of POL, STA alone, or in combination with EPI, ENZ as measured by Western blotting. β‐ACTIN protein was used as the loading control. C) Cellular PRDX5 activity was measured by enzymatic assay in the presence of POL, STA, EPI, PRDX5 protein, or siPRDX5 alone or in combination. PRDX5 activity was inhibited by POL, STA, and siPRDX5, but increased by PRDX5 protein. D) Cell death rate (%) was measured by LDH release assay in the presence of POL, STA, EPI, PRDX5 protein, or siPRDX5 alone or in combination. Data are expressed as mean ± std of triplicates. One‐way ANOVA with the Turkey test was performed. *p*<0.05 was considered significant and indicated by different letters. E) PRDX5 inhibitor slowed down CRPC progression in mice. Weight and photograph of the prostate are shown from *MYC* ^T^ mice (6 per group) treated with enzalutamide (ENZ), abiraterone (ABI), polaprezinc (POL), stachyose (STA), or in combinations. AP: anterior prostate lobe, DL: dorsolateral prostate lobe, VP: ventral prostate lobe. Student's *t*‐test was performed, ** and ***indicate *p*<0.01 and *p*<0.001, respectively. F) Representative images of prostate histopathology. Age and treatment are labeled.

Due to the uncertainty of the auranofin target (TXNRD or PRDX5) in the literature and the unavailability of auranofin clinically in China, we performed molecular docking experiments using three libraries (natural compound library, chemical library, and clinical drug library) to search for small molecule drugs that can effectively target PRDX5. We found that polaprezinc and stachyose tetrahydrate have high dock scores (Figure S4, Supporting Information). Polaprezinc (POL) is a medication used to treat gastric ulcers and was reported to affect the expression of PRDX5. ^[^ ^13^ ^]^ Stachyose (STA) is an oligosaccharide, a rich component in our diet. POL had IC_50_ of 4.71 and 4.15 µM, STA had IC_50_ of 4.08 and 3.82 µM for LNCaP epiDTP and enzDTP, respectively (**Figure** **5**A). POL and STA did not significantly alter the level of PRDX5 protein (Figure 5B), rather they inhibited PRDX5 enzymatic activity in epiDTP (Figure 5C) and enzDTP (Figure S5A) and increased mortality of epiDTP (Figure 5D) and enzDTP cells (Figure S5B, Supporting Information). The addition of PRDX5 protein enhanced PRDX5 enzymatic activity (Figure 5C) and rescued cell mortality (Figure S5A, Supporting Information) whereas *PRDX5* silencing reduced PRDX5 enzymatic activity (Figure 5D) and exacerbated cell death (Figure S5B, Supporting Information). Therefore, we repurposed POL and developed STA as a selective PRDX5 inhibitor for the treatment of CRPC.

We evaluated the efficacy of these inhibitors in treating CRPC tumors. *MYC* transgenic mice develop prostate PIN/tumor lesions with increasing age, and treatment of the mice with ENZ or abiraterone (ABI) at 4 months of age inhibited prostate growth but CRPC emerged at 7 months of age (Figure 5E). Administration of POL alone at 7 months of age delayed the CRPC progression and POL in combination with ENZ or ABI delayed the progression further (Figure 5E). STA possessed a stronger suppressive effect on CRPC (Figure 5E,F). Administration of STA i.v. (as high as 1 g kg^−1^ body weight) in wild‐type mice had no significant side effects on biochemical parameters (Figure S6A, Supporting Information) nor on mouse body weight and food intake (Figure S5C, Supporting Information). There were no significant side effects on the histology of the liver, spleen, kidney, and intestine tissues (Figure S6B, Supporting Information). Non‐compartmental analysis of plasma data after intravenous bolus input showed favorable PD/PK characteristics (Figure S6C, Supporting Information). Therefore, STA and its derivatives may be explored as drugs for the future treatment of CRPC patients.

### 2.6 PRDX5 Inhibition Stabilizes CRPC Tumors in Patients

**Figure 6:**
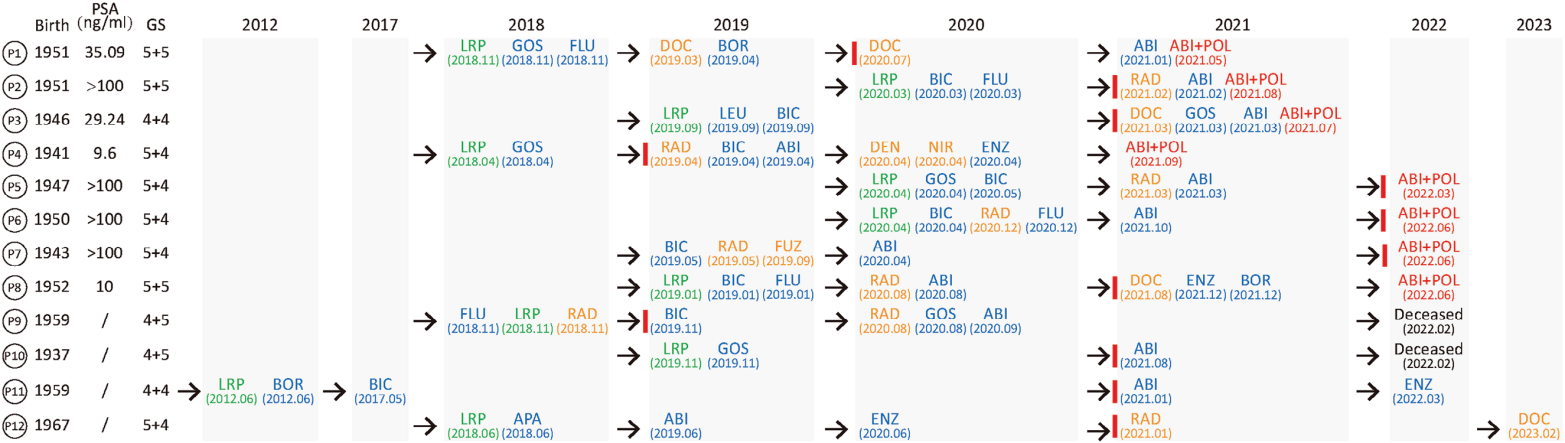
Patient Medical history. The medical history of 12 CRPC patients is shown. Green letters indicate prostate‐related surgery; Blue letters indicate androgen deprivation therapies; Orange letters indicate prostate cancer‐related radio‐ and chemotherapies; Red letters indicate ABI+POL treatment. The red vertical line represents the diagnosis of CRPC. PSA: prostate‐specific antigen; GS: Gleason scores; LRP: Laparoscopic radical prostatectomy; BOR: Bilateral orchidectomy; CHEMO: Chemotherapy; RAD: Radiotherapy; GOS: Goserelin; FLU: Flutamide; BIC: Bicalutamide; DOC: Docetaxel; LEU: Leuprorelin; DEN: Denosumab; NIR: Niraparib; ENZ: Enzalutamide; FUZ: Fuzuloparib; APA: Apalutamide; ABI: Abiraterone.

To verify the effectiveness of PRDX5 inhibition clinically, we recruited 12 CRPC patients, with their fully informed consent, to the Affiliated Hospital of Jiangnan University. These patients are between the age of 71–82, were with high levels of PSA and Gleason scores at the initial diagnosis, had multiple prior treatments with various drugs, and were diagnosed as CRPC (**Figure** **6**). Patients 1–8 were given abiraterone/prednisone plus POL (75 mg b.i.d) for 6 months, and patients 9–12 were on other treatments for CRPC (Figure S7A). We monitored patient PSA levels monthly and took emission computed tomography (ECT) images before and after the treatment for patients who made it to the imaging facility during the COVID‐19 epidemic.

**Figure 7:**
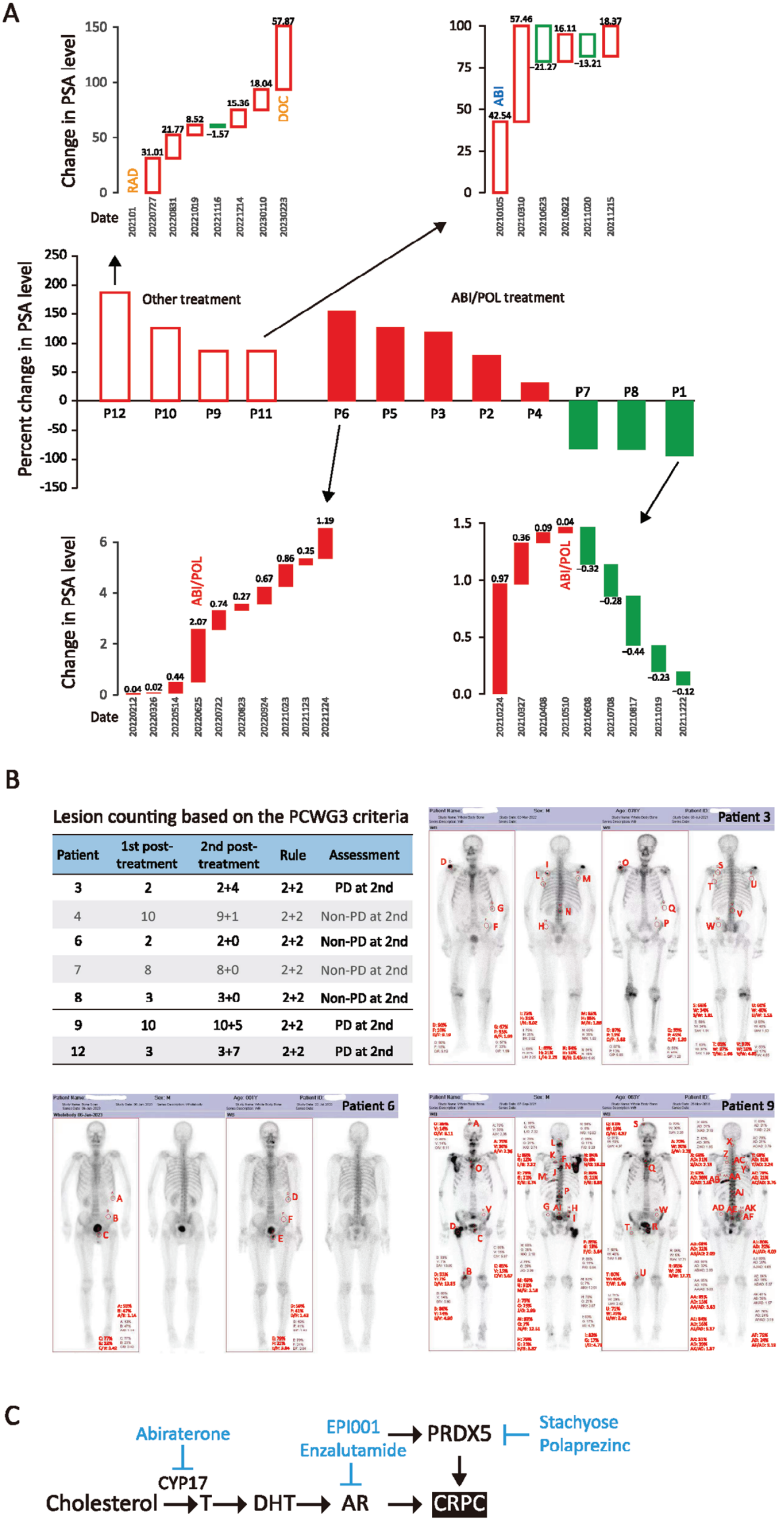
Stabilization of CRPC tumor by PRDX5 inhibitor in patients. A) Waterfall plot of PSA progression in patients treated with ABI+POL (75 mg b.i.d, 6 months) (Solid columns) or other treatments (hollow columns). The red and green columns indicate the percentage of increase and decrease in PSA expression, respectively. Monthly PSA levels for patients with the least and most PSA changes in the ABI+POL treatment group (patients 6 and 1) and the other treatment group (patients 12 and 11) are also shown. B) Prior and post‐treatment ECT images for patients 3 & 6 of the ABI+POL group and patient 9 of the other treatment group. Lesion density was calculated after background subtraction. Lesion numeration according to the PCWG3 criteria is shown. PD: progressive disease. C) Diagram depicts the mechanism of action of POL and STA on CRPC.

From the PSA waterfall plot of all patients, we found that patients 1, 7, and 8 had a significant PSA reduction during the ABI+POL treatment, and patients 2, 3, 4, and 5 had a PSA reduction at some points but higher PSA level after six months of treatment, and patient 6 had PSA progression through the treatment (**Figure** **7**A; Figure S7B, Supporting Information). Patients 9, 10, 11, and 12, who were on other treatments, had PSA progression (Figure 7A; Figure S7B, Supporting Information). The mean percent change of PSA in the ABI+POL group was 31.31% (95% CI [−41.45%, 104.07%]; IQR [207.93]), while the other group was 120.69% (95% CI [56.17%, 149.21%]; IQR [70.13]). In addition, we plotted PSA levels and calculated slopes. A slope ratio (after/before treatment) smaller than 1 means stabilization in PSA progression, ratio 1 means no effect on PSA, and a ratio greater than 1 means acceleration in PSA progression. All patients in the ABI+POL group (8/8) had PSA stabilization, whereas patients in the other treatment group had PSA acceleration (Figure S7C, Supporting Information).

We also performed ECT imaging analysis according to the Prostate Cancer Clinical Trials Working Group 3 (PCWG3) criteria^[^ ^14^ ^]^ (https://www.calyx.ai/wp‐content/uploads/2021/11/CALYX‐MI‐WhitePaper‐ProstateCancer‐RP.pdf). We found that patients 4, 6, 7, and 8 with ABI+POL treatment had non‐progressive disease (Non‐PD), and patient 3 continued to progress (PD). Patients 9, and 12 with other treatments showed disease progression (PD) (Figure 7B; Figure S7D, Supporting Information). No CRPC‐related mortality occurred in the ABI+POL group whereas 2 out of 4 patients on the other treatments had succumbed to CRPC. These results indicate that PRDX5 inhibition can slow down PSA progression (8/8 patients) and stabilize metastatic CRPC (mCRPC) (4/5 patients). Other treatments did not stabilize PSA (0/4 patients) or mCRPC (0/2 patients).

## 3 Discussion

We have demonstrated the involvement of the DTP state in castration resistance and shown the role of the thioredoxin/peroxiredoxin pathway in CRPC development. PRDX5 as a therapeutic target is validated in cell culture, animal models, and CRPC patients. We have repurposed polaprezinc, a gastric ulcer medication, and developed stachyose, a dietary component, as a novel PRDX5 inhibitor (Figure 7C). Our study suggests that PRDX5 inhibition could become a new concept and practice for the management of castration‐resistant prostate cancer.

The notion of DTP originated from microbiology, where a subpopulation of bacteria can survive in antibiotics without initially being genetically resistant.^[^ ^15^ ^]^ Analogously, persister cancer cells are characterized by resistance to drugs, lack new driver genetic alterations, and are phenotypically reversible. A pioneering study using PC‐9 lung cancer cell lines treated with erlotinib revealed a small fraction of viable quiescent cells that survived drug treatment for 9 days, referred to as DTP, that resumed proliferation in continuous drug exposure for 33 days, referred to as DTEP.^[^ ^8^ ^]^ In this study, we showed that no additional mutations occurred on AR mRNA in cells treated with inhibitors and that these cells do resist drugs and were phenotypically reversible. We, however, did not sequence the whole genome to ascertain no other genetic alterations. Therefore, AR inhibitor tolerant persister‐like state and tolerant expanded persister‐like state may be a better description of these cells. The upregulation of PRDX5 in AR inhibitor resistance is consistent with metabolic remodeling. Traditionally, CRPC drugs work by either reducing dihydrotestosterone biosynthesis or blocking AR signaling. Our results not only indicate the existence of an AR inhibitor‐tolerant persister‐like state but also identify a novel pathway for the treatment of CRPC.

PRDX5 is an antioxidant enzyme, which reduces hydrogen peroxide and alkyl hydroperoxides. The peroxidic Cys48 thiol (Cys48‐SH) on PRDX5 is oxidized to sulfenic acid (Cys48‐SOH). The Cys152 then reacts with Cys48‐SOH to form an intramolecular disulfide (Figure 3E). Thioredoxins (cytosolic TXN1 or mitochondrial TXN2) reduce the disulfide bond, regenerate a sulfhydryl group on Cys48, and thereby reactivate PRDX5. TXN activity is then regenerated by thioredoxin reductase (TXNRD) in a NADPH‐dependent manner.^[^ ^16^ ^]^ Upregulation of TXN2, instead of TXN1, implicates mitochondrial association with AR inhibitors‐resistant DTP state. The high expression of TXNRD (TNXRD1, TNXRD2, TNXRD3) and PRDX5 indicates the activation of the thioredoxin/peroxiredoxin pathway. A significant difference between 22Rv1 and LNCaP cells is that the former is inherently ENZ‐resistant^[^ ^11^ ^]^ and expresses a high level of PRDX5 (Figure 3F), whereas the latter developed ENZ‐resistance after being exposed to ENZ in culture. Interestingly, glutathione peroxidase 4 (GPX4), which catalyzes the reduction of hydrogen peroxide, organic hydroperoxides, and lipid hydroperoxides, also plays a key role in therapeutic drug‐resistant DTP cells.^[^ ^17^ ^]^

PRDX5 inhibitor is effective on DTP cells treated with EPI001 or enzalutamide in vitro and on CRPC developed after physical castration, chemical castration with abiraterone, or AR blockade with EPI001 and enzalutamide in vivo. Thus, PRDX5 seems to be broadly involved in various forms of castration. Darolutamide, a new AR inhibitor, is promising for the treatment of nonmetastatic CRPC.^[^ ^7^ ^]^ We have also noticed the upregulation of PRDX5 in darolutamide‐treated DTP cells (Figure S8A–G, Supporting Information). Therefore, PRDX5 inhibitor may also be effective against darolutamide‐resistant CRPC if such a case arises clinically.

In addition, we detected neuroendocrine (NE)‐like cell biomarkers (CgA, CgB, NSE, PTHrP, and AMACR)^[^ ^18^ ^]^ in LNCaP cells, LNCaP‐derived DTPs, 22Rv1 cells, and EPI/ENZ treated 22Rv1 cells, respectively. CgA, NSE, and PTHrP were highly expressed in LNCaP‐derived DTPs and EPI/ENZ treated 22Rv1 cells. CgB was highly expressed in 22Rv1 and EPI/ENZ treated 22Rv1 cells. However, no obvious change in AMACR was found in our experiment. The results indicate that some NE‐like biomarkers are significantly overexpressed in DTP cells and associated with AR inhibitor‐resistance. Interestingly, these NE‐like biomarker‐positive cells are sensitive to PRDX5 inhibition and thus PRDX5 may also be an effective therapeutic target for neuroendocrine PCa.

The majority of patients involved in the present study had high levels of PSA and Gleason scores at the initial diagnosis, had multiple prior treatments such as laparoscopic radical prostatectomy, radiotherapy, chemotherapy, and physical/chemical castration with bilateral orchidectomy, goserelin, leuprorelin, flutamide, bicalutamide, enzalutamide, abiraterone, and were diagnosed as mCRPC before taking polaprezinc (Figure 6). Patient 1, however, had nonmetastatic CRPC (nmCRPC) and his PSA level was declined after POL administration. Likely, PRDX5 inhibitor treatment can also be used in nonmetastatic CRPC (nmCRPC) patients.

Currently, there are a few other options available for the treatment of mCRPC. Poly ADP‐ribose polymerase (PARP) inhibitors such as olaparib (Lynparza) and rucaparib (Rubraca) are limited to patients with BRCA mutations.^[^ ^19^ ^]^ Sipuleucel‐T, an autologous antigen‐presenting cell‐based immunotherapy, prolongs the overall survival of mCRPC but does not affect the time to disease progression.^[^ ^20^ ^]^ Radiotherapy with lutetium‐177 (177Lu‐PSMA‐617) has been used successfully in mCRPC patients but with adverse events such as xerostomia, nephrotoxicity, gastrointestinal disturbance, and myelosuppression.^[^ ^21^ ^]^ Other potential targets such as STEAP‐1, TROP2, CD46, and B7‐H3 are under preclinical or clinical development, which are surface antigens suitable for low toxic antibody‐drug conjugates.^[^ ^22^ ^]^ These molecules are used for drug delivery, rather than therapeutic targets per se. In our study, patients did not experience any noticeable side effects while on POL with 75 mg b.i.d. for six months. STA is an oligosaccharide and accumulates in the colon as a nutrient for gut bacteria. PRDX5 inhibitors such as STA or its derivatives are reasonably safe and may have an excellent therapeutic index.

Although we show that 6‐month POL treatment stabilizes CRPC in many patients, larger clinical trials are needed to determine the appropriate dose, duration, and regimen for optimal CRPC treatment. STA demonstrates a safe profile and higher efficacy than POL in animal models, and thus additional preclinical and clinical investigations are warranted.

## 4 Experimental Section

### Cell Culture

LNCaP (ATCC CRL‐1740), 22Rv1 (ATCC CRL‐2505), DU145 (ATCC HTB‐81), and PC‐3 (ATCC CRL‐1435) cells were grown in phenol red‐free Roswell Park Memorial Institute (RPMI)−1640 medium (Thermo Fisher Scientific, CA, USA) containing 5% fetal bovine serum (FBS; Thermo Fisher Scientific) and 1% streptomycin‐penicillin at 37 °C with 5% CO2. The cell lines were authenticated by short tandem repeat analysis and mycoplasma contamination was evaluated by the PCR Mycoplasma Detection Set (Takara, Otsu, Japan). The cells were treated with the EPI‐001 (EPI), enzalutamide (ENZ), or darolutamide (DAR) with different concentrations and times appropriate to obtain drug‐tolerant persister cells described by Sharma et al. ^[^ ^8^ ^]^

### Chemicals

Enzalutamide (MCE, HY‐70002, NJ, US), EPI‐001 (Selleck, S7955, TX, US), Auranofin (MCE, HY‐B1123, NJ, US), Polaprezinc (MCE, HY‐B0729, NJ, US) and Darolutamide (Selleck, S7559, TX, US) were stored as stock solutions in DMSO (Sigma, MO, US). FDA‐approved drug library (Selleck, L1300, TX, US) was stored at −80 °C.

### Cell Viability Analysis

Cell viability was assessed by Cell Counting Kit (CCK‐8; MCE, HY‐K0301, NJ, US) according to the manufacturer's instructions. Briefly, cells were seeded at a concentration of 4000 cells/200 µL well^−1^ into 96‐well plates, incubated overnight, and changed to fresh medium with various inhibitors. Following treatment, 10 µL CCK‐8 solution was added and cells were incubated for 4 h at 37 °C. The optical density (OD) value was measured at 450 nm by a microplate spectrophotometer (Thermo Fisher Scientific). After trypsinization of cells, placental blue staining was added, and viable cells were enumerated using a hemocytometer. Three independent experiments were performed, each in triplicates.

PRDX5 protein or siPRDX5 was added in the absence or presence of drugs. After 24 h treatment, lactate dehydrogenase (LDH) release assay was performed using a 2‐p‐iodophenyl‐3‐nitrophenyl tetrazolium chloride/ diaphorase‐based kit (C0017, Beyotime, Jiangsu, CN) according to the manufacturer's instructions.

### Generation of DTP and DTEP

Drug‐sensitive cells were treated with the AR‐inhibitors EPI, ENZ, or DAR, at concentrations 100 times the established IC_50_ values, for three rounds, with each treatment lasting 48 h. Viable cells remaining attached on plates at the end of the third round of drug treatment (9 days) were considered as DTP and were collected for analysis. The DTP cells eventually resume normal proliferation on continuous exposure to drugs, and become DTEP cells after 33 days of treatment, which can be propagated in the presence of drugs indefinitely.^[^ ^8^ ^]^

### Drug Combination Index Measurement

The combination index (CI) was determined as described previously.^[^ ^23^ ^]^ Briefly, DTP cells were seeded into 96‐well plates, and IC_50_ concentration for each drug, as well as two drug combinations, were determined. The following equation was used: $CI\;=\frac{(D)1}{(\mathrm{Dx})1}\;+\frac{(D)2}{(\mathrm{Dx})2}$; where (D)_1_ and (D)_2_ were the respective combination doses of drug 1 and drug 2 that yield an effect of 50% growth inhibition, with (Dx)_1_ and (Dx)_2_ being the corresponding single doses for drug 1 and drug 2 that result in the same effect. A CI of less than, equal to, and more than 1 indicates synergy, additivity, and antagonism, respectively.

### Giemsa Staining

Cells were washed 3 times with pre‐cooled PBS and fixed with methanol. After drying, 1x Giemsa solution (Beyotime, C0133, SH, CN) was added to stain for 2 min and then rinsed with pure water. Cells were photographed under a Nikon TE300 Inverted Fluorescence Microscope.

### FACS Cell Cycle Analysis

Cells were harvested, washed, and then fixed with 70% ethanol solution (v/v) at 4 °C for more than 18 h. After washing with pre‐cold PBS, cells were stained with propidium iodide (PI) containing RNase (PI/RNase Staining Solution, CST 4087, MA, US) for 15 min in the dark, and then subjected to cell cycle analysis on a flow cytometer. The cell cycle data were analyzed using ModFit LT 5.0 software (Verity, ME, US).

### Quantitative Real‐Time PCR

Total RNA was extracted using Fastpure cell/tissue total RNA isolation kit (RC101, Vazyme Biotech Co., Ltd, Nanjing, China) according to the manufacturer's instructions. 1 µg of total RNA was used for cDNA synthesis using a cDNA reverse transcription kit (R323, Vazyme, Nanjing, China). Real‐time PCR was performed in triplicate using gene‐specific primers (Table S7, Supporting Information) on a Bio‐Rad CFX96 PCR system.

### DNA Sequence Analysis

After total RNA extraction and reverse transcription, the region of the AR gene was divided into 4 segments and then amplified by PCR (Table S7, Supporting Information). The PCR products of LNCaP and LNCaP‐derived DTPs and DTEPs groups were sequenced and analyzed by Jiangsu Saisofi Biotechnology Co., Ltd (Wuxi, China).

### Immunofluorescence

Immunofluorescent staining was performed as previously described.^[^ ^24^ ^]^ The antibodies used were mouse anti‐E‐cadherin (Cell Signaling Technology, 14 472, Clone 4A2; 1:100), and rabbit anti‐Vimentin (Cell Signaling Technology, 5741, Clone D21H3; 1:100). Secondary antibodies used were AlexaFluor 488 goat anti‐mouse/rabbit IgG antibodies (Thermo Fisher Scientific). The cell nuclei were stained with DAPI (Sigma, D9542, MO, US). Samples were observed under an inverted laser confocal microscope (ZEISS, LSM 900, Jena, DE).

### Proteomics Study

LNCaP, 22Rv1, epiDTP, enzDTP, epiDTEP, and enzDTEP cells were used for TMT quantitative proteomics analysis. Samples were lyzed with 8 M urea (pH = 8.0) and concentration was quantified using a BCA kit (Beyotime, P0012, Shanghai, China). Proteins were reduced with dithiothreitol (DTT) and then alkylated with iodoacetamide (IAM) in the dark. Sequencing‐grade trypsin (Promega, WI, USA) was added for overnight digestion. Peptides were desalted and reconstituted in 0.5 M tetraethylammonium bromide (TEAB) and processed according to the manufacturer's protocol for the TMT kit. Briefly, one unit of TMT reagent was thawed and reconstituted in acetonitrile. The peptide mixtures were then incubated for 2 h at room temperature and pooled, desalted, and dried by vacuum centrifugation. The labeled peptides were fractionated into fractions by high pH reverse‐phase HPLC using Agilent 300Extend C18 column (5 µm X 4.6 mm X 250 mm). The peptides were resuspended in 2% acetonitrile (ACN) and 0.1% formic acid (FA) solution and then analyzed using an EASY‐nLC 1200 system (Thermo Fisher Scientific) coupled with a high‐resolution Orbitrap Fusion Lumos mass spectrum (Thermo Fisher Scientific). Peptides were first separated with an RSLC C18 column (1.9 µm × 100 µm × 20 cm) packed in‐house, then selected for MS/MS using the NCE setting as 28 and the fragments were detected in the Orbitrap at a resolution of 17500. A data‐dependent procedure that alternated between one MS scan followed by 20 MS/MS scans with 15.0 s dynamic exclusion. Automatic gain control (AGC) was set at 5E4. The fixed first mass was set as 100 m z^−1^.

The resulting MS/MS data were analyzed by the MaxQuant with an integrated Andromeda search engine (version 1.4.1.2). The search for tandem mass spectra was implemented in the SwissProt human database concatenated with a reverse decoy database. Trypsin/P was defined as the cleavage enzyme allowing up to two missing cleavages. For proteomic analysis, the first search range was set to 5 ppm for precursor ions, and the main search range was set to 5 ppm and 0.02 Da for fragment ions. The carbamidomethylation of cysteines was defined as the fixed modification, and the oxidation of methionine was defined as the variable modification. The quantification method used was TMT, the FDR was adjusted to <1%, and the minimum score for modified peptides was >40. To identify the differentially expressed proteins (DEPs) between compared groups, the following criteria were used: |Log2 FC| >2 and *p*<0.05.

### Small‐Interfering RNA (siRNA)‐Mediated Gene Knockdown

hPRDX5 siRNA (siPRDX5; 5′‐GGUGGCCUGUCUGAGUGUUTTAACACUCAGACAGGC CACCTT‐3′) was made by Jiangsu Saisofi Biotechnology Co., Ltd (Wuxi, China). The siRNAs were transfected into LNCaP‐derived DTP cells using Polyplus‐transfection (jetPRIME, NY, US) according to the manufacturer's instructions.

### Animal Study

Hi‐*MYC* transgenic prostate cancer mice (gifted from George V. Thomas laboratory)^[^ ^25^ ^]^ were used and all experimental protocols were approved by the Animal Ethics Committee of Jiangnan University, China (JN.No20190630t1360101[191]). To define the temporal development of castration resistance, four‐month‐old mice were randomly assigned into the control (NC) and ENZ (i.g. 10 mg Kg^−1^) group, treated with a drug by intragastric administration every 3 days. The mice were then euthanized every month, and the prostate (anterior lobes, dorsal lateral lobes, and ventral lobes) were dissected, photographed, and weighed. After an initial decrease in prostate weight, regaining prostate growth was considered the emergence of castration resistance.^[^ ^26^ ^]^ The mice were randomly divided into ENZ (i.g. 10 mg Kg^−1^), POL (i.g. 15 mg Kg^−1^), POL plus ENZ, STA (i.v. 1 g Kg^−1^), and STA plus ENZ groups. Similar experiments with abiraterone instead of enzalutamide were also performed. Prostate were dissected, photographed, weighed, and then subjected to histopathological analysis.

Total knockout of *Prdx5* mice was generated by Jiangsu Aniphe Biolaboratory (Nanjing, CN). *Prdx5* ^+/−^;*MYC* ^T^ mice were obtained by cross‐breeding. Experimental protocols were approved by the Animal Ethics Committee of Jiangnan University, China (JN.No20201215t0640811[357]).

### Tissue Hematoxylin‐Eosin (HE) Staining

Briefly, after deparaffinization and rehydration, 5 µm thick longitudinal sections were stained with hematoxylin solution for 22 s, dipped in 1% hydrochloric acid ethanol, rinsed with distilled water, stained with eosin solution for 30 s, dehydrated with graded alcohol and cleared in xylene. Mounted slides were scanned using a Pannoramic Scanner (3DHISTECH, Budapest, HU).

### Serum Biochemical Marker Analysis

Blood samples were collected from mice and left to coagulate at room temperature (25 °C) for 1 h. After centrifuging the blood samples at 3500 rpm for 10 min, serum samples were harvested into an Eppendorf tube and stored at −80 °C until further analysis. Serum glucose (GLU), total cholesterol (TC), triglycerides (TG), low‐density lipoprotein cholesterol (LDL‐C), and high‐density lipoprotein cholesterol (HDL‐C) were measured using an automatic biochemical analyzer (Mindray BS‐480; Mindray, Shenzhen, CN). At the same time, the kidney function markers such as creatinine (CREA‐J) and urea (UREA) and the liver function markers such as alanine aminotransferase (ALT), aspartate aminotransferase (AST), alkaline phosphatase (ALP), glutamyl transpeptidase (GGT), and cholinesterase (CHE) were measured.

### Pharmacokinetic (PK)/ Pharmacodynamics (PD) Analysis

Stachyose (2 g kg^−1^ body weight) were administrated intravenously. Mouse blood was obtained at 5, 15, and 30 min and at 1, 2 4, 8, 12, 24, and 48 h. The serum level of stachyose was quantified by LC mass spectrometry. PD/PK parameters were calculated using a Non‐compartmental analysis of plasma data after the intravenous bolus input model with the PKSolver Excel add‐in.^[^ ^27^ ^]^

### Western Blot

Cells were treated as described and then lysed by boiling for 10 min in sample buffer (2% SDS, 10% glycerol, 10% β‐mercaptoethanol, bromophenol blue, and Tris‐HCl, pH = 6.8). Lysates were fractionated on SDS‐PAGE gels and transferred to PVDF membranes (Millipore, IPVH00010, NH, US). The blots were probed with specific antibodies followed by secondary antibody then membranes were detected by ECL (Sigma, WBULS0500, MO, US). PRDX5 (67599‐1‐Ig; 1:2000), CDKN1A (p21) (60214‐1‐Ig; 1:1000), Cyclin E1 (CCNE1) (11554‐1‐AP; 1:1000), Cyclin B1 (CCNB1) (67686‐1‐Ig; 1:5000), CDC6 (11640‐1‐AP; 1:2000), CDC2 (CDK1) (19532‐1‐AP; 1:1000), TXNRD1 (67728‐1‐Ig; 1:10 000), TXNRD2 (16360‐1‐AP; 1:3000), TXNRD3 (19517‐1‐AP; 1:2000), TXN2 (13089‐1‐AP; 1:1000), NSE (66150‐1‐Ig; 1:10 000), CgA (60135‐2‐Ig; 1:3000), CgB (14968‐1‐AP; 1:2000), PTHrP (29115‐1‐AP; 1:800), AMACR (15918‐1‐AP; 1:500), GAPDH (60004‐1‐Ig; 1:50 000), β‐ACTIN (66009‐1‐Ig; 1:10 000) antibodies were purchased from Proteintech Group (IL, US). β‐TUBLIN (86 298; 1:10 000) antibody was purchased from Cell Signaling Technology (MA, US). Secondary antibodies were conjugated with HRP (Proteintech Group; SA00001‐1, SA00001‐2; 1:10 000).

### Generation of PRDX5 Overexpressed LNCaP Cell Line by Lentivirus

The full‐length coding sequence of the PRDX5 gene was amplified by PCR, inserted into the YOE‐LV001 lentiviral expression vector (Ubigene Biosciences Co., Ltd., Guangzhou, CN), and then transfected with lentiviral packaging vector into 293T cells. PRDX5 and control lentivirus were used to infect prostate cancer cells and successful expression was verified by Western blotting.

### Generation of PRDX5 Knockout 22Rv1 Cell Line by CRISPR‐Cas9

Single‐stranded guided RNAs (sgRNAs) were designed using the online CRISPR design tool (Red Cotton, Guangzhou, CN, https://en.rc‐crispr.com/). The exon 1 region of PRDX5 was targeted by CRISPR/Cas9 genome editing. A ranked list of sgRNAs was generated with specificity and efficiency scores. The pair of oligos for two targeting sites was annealed and ligated to the YKO‐RP003 vector (Ubigene Biosciences Co., Ltd., Guangzhou, China). 22Rv1 cells were transfected with the YKO‐RP003‐hPRDX5 plasmids containing each target sgRNA sequence using Lipofectamine 3000 (Thermo Fisher Scientific). Puromycin was added 24–48 h post‐transfection. After antibiotic selection, a certain number of cells were diluted and inoculated into a 96‐well plate. Selection of single clones was performed after 2–4 weeks and selected PRDX5 knockout clones were selected by limited dilution method and validated by PCR and Sanger sequencing. The sgRNAs and primers were shown in Table S7 (Supporting Information).

### PRDX5 Activity Assay

Enzymatic activity of PRDX5 was measured using the thioredoxin system as described previously (15). The rate of H_2_O_2_ degradation was measured by monitoring the decrease in A340 caused by NADPH oxidation. The assay was performed in a 150 µL reaction mixture, containing 50 mM HEPES‐NaOH (pH = 7), 200 µM NADPH, 760 nM mouse TXNRD1, 11 µM human TRX, and different concentration gradients of PRDX5. The mix was incubated at 37 °C for 5 min, and the reaction was initiated by the addition of 500 µM H_2_O_2_.

### Clinical Study

CRPC patients were enrolled with their fully informed consent. Patients were on abiraterone/prednisone, abiraterone/prednisone plus polaprezinc granules, or with other treatments according to patients’ wishes in consultation with physicians (the Affiliated Hospital of Jiangnan University). Polaprezinc was 75 mg b.i.d., for 6 months. PSA was measured every month. ECT or PET‐CT were taken before and after treatment. The study was approved and authorized by the Ethics Committee of the Affiliated Hospital of Jiangnan University (Approval document number: LS202128) and is registered at ClinicalTrials.gov (NCT05549778).

### Primary outcome measure

1. PSA response (defined as a decrease in PSA level or slowdown in PSA progression).
2. Non‐progressive disease (Non‐PD) by ECT imaging analysis according to the Prostate Cancer Clinical Trials Working Group 3 (PCWG3) criteria.

Inclusion criteria for the study:

1. Male patients were older than 18 years of age.
2. Patients with measurable disease were required to have documented disease progression by Response Evaluation Criteria in Solid Tumors (RECIST) ^[^ ^28^ ^]^ with at least one bone metastatic lesion. Patients with non‐measurable disease were required to have at least two consecutive increases (relative to a reference value measured at least a week apart) in serum PSA.
3. Patients had been taking abiraterone or enzalutamide for at least three consecutive months and showed a persistent rise in PSA.
4. Life Expectancy >6 months

Exclusion criteria for the study:

1. Patients had taken polaprezinc previously.
2. Patients had cancer therapy (other than ADT) within 4 weeks before enrolment.
3. Patients had malignancies other than prostate cancer.
4. Patients had uncontrolled severe illness or medical conditions.

#### 4.1 Molecular Docking

PRDX5 and small molecule (natural library, marketed library, and clinical library) docking were performed as previously described using the Discovery studio software. ^[^ ^29^ ^]^

#### 4.2 Statistical Analysis

Student's *t*‐test was used to compare the means of the two groups. One‐way ANOVA was used to compare means of ≥3 groups (GraphPad, CA, USA). Turkey test was used to perform multiple comparisons (IBM SPSS, NY, USA). Data were presented as mean ± std of biological repetitions. *p*<0.05 was considered significant in all tests. Materials and Methods

## Conflict of Interest

The authors declare no conflict of interest.

## Author Contributions

Conceptualization: YQC, RW, NHF, LWD Methodology: RW, YYM, YW, JN Investigation: RW, YYM, XBR, SYT Visualization: RW, YQC, QYS, JN Funding acquisition: YQC, LWD, HPK Project administration: RW, YQC Supervision: YQC, NHF, HPK, SYT Writing–original draft: RW Writing–review & editing: YQC, LWD, XBR

## Supporting information

Supporting Information

## Data Availability

The data that support the findings of this study are available on request from the corresponding author. The data are not publicly available due to privacy or ethical restrictions.
