# Supplementary material for: Identification of PRDX5 as A Target for The Treatment of Castration‐Resistant Prostate Cancer
Source: Adv Sci (Weinh). 2023 Dec 20;11(9):2304939. doi: 10.1002/advs.202304939 (PMC10916659; doi:10.1002/advs.202304939)
Supplement: Supplementary file 1 — Supporting Information [file ADVS-11-2304939-s001.pdf]

ADVANCED SCIENCE
Open Access

Supporting Information

for *Adv. Sci.*, DOI 10.1002/advs.202304939

Identification of PRDX5 as A Target for The Treatment of Castration-Resistant Prostate Cancer

Rong Wang, Yuanyuan Mi, Jiang Ni, Yang Wang, Lingwen Ding, Xuebin Ran, Qiaoyang Sun, Soo Yong Tan, H Phillip Koeffler, Ninghan Feng and Yong Q Chen**

SUPPLEMENTARY MATERIALS

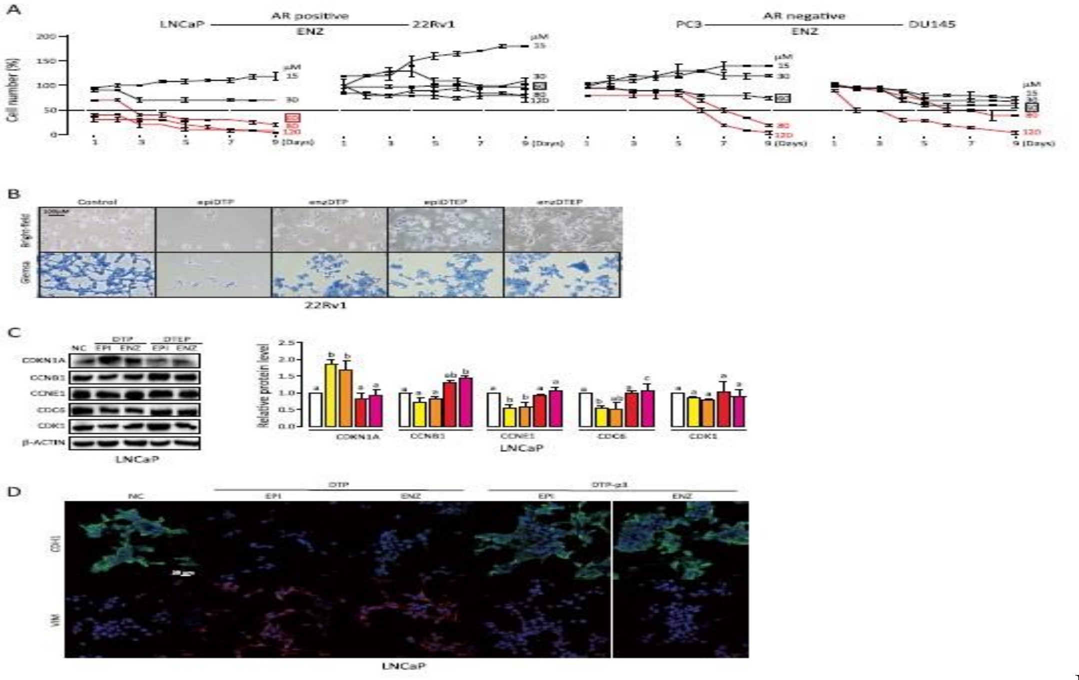

Figure S1

A: AR-positive LNCaP, 22Rv1 PCa cells, and AR-negative PC3, DU145 PCa cells were treated with different concentrations (15-120 mM) of ENZ for 9 days. Less than 50% LNCaP cells, but the majority of AR negative cells, survived at 60 mM of ENZ. 22Rv1 is ENZ resistant. Data were expressed as percentages relative to the number of seeded cells (1x106) as measured by hemocytometer and as mean ± std of triplicates. B: light microscopic images of 22Rv1 cells exposed to EPI (epiDTP) and ENZ (enzDTP) for 9 days or to EPI (epiDETP) and ENZ (enzDETP) for 33 days. C: Expression of cell cycle markers in LNCaP DTP and DETP cells as measured by Western blotting. β-ACTIN protein was used as the loading control. The right panel shows relative protein quantity expression. Data are expressed as mean ± std. of triplicates. One-way ANOVA with the Turkey test was performed. $p<0.05$ was considered significant and indicated by different letters. D: Representative showing Expression of E-cadherin (CDH1 in cyan) and vimentin (VIM in red) in LNCaP, DTP, and DTP p3 cells as measured by immunofluorescent microscopy. Cell nuclei were stained with DAPI (in blue).

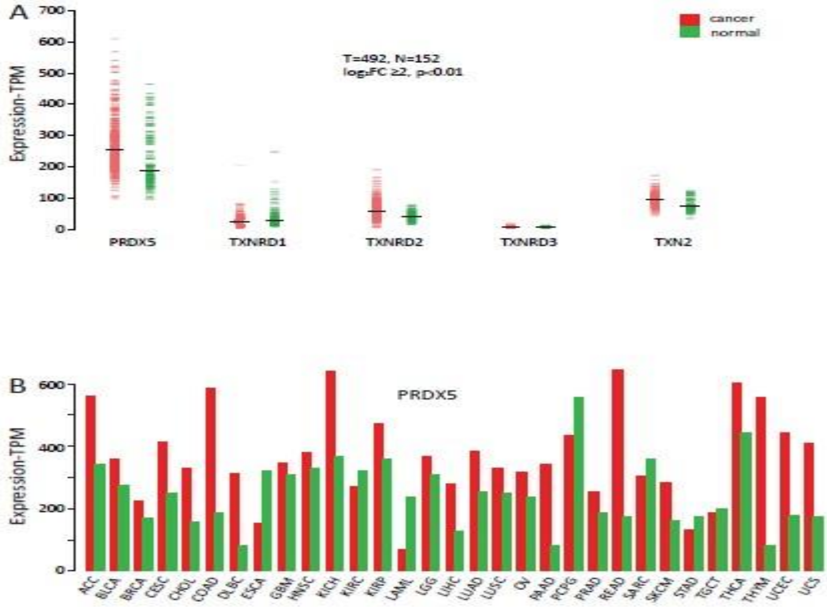

Figure S2

A: Relative expression of PRDX5, TXNRD1, TXNRD2, TXNRD3, and TXN2 in human prostate tumor and normal tissues. B: Relative expression of PRDX5 in different cancer tissues. Data were obtained from the Gene Expression Profiling Interactive Analysis (GEPIA) database (http://gepia.cancer-pku.cn/). TPM = Transcripts Per Million; ACC: adrenocortical carcinoma; BLCA: bladder urothelial carcinoma; BRCA: breast invasive carcinoma; CESC: cervical and endocervical cancers; CHOL: Cholangiocarcinoma; COAD: colon adenocarcinoma; DLBC: lymphoid neoplasm diffuse large B-cell lymphoma; ESCA: esophageal carcinoma; GBM: glioblastoma multiforme; HNSC: head and neck squamous cell carcinoma; KICH: kidney chromophobe; KIRC: kidney renal clear cell carcinoma; KIRP: kidney renal papillary cell carcinoma; LAML: acute myeloid leukemia; LGG: brain lower grade glioma; LIHC: liver hepatocellular carcinoma; LUAD: lung adenocarcinoma; LUSC: lung squamous cell carcinoma; S OV: ovarian serous cystadenocarcinoma; PAAD: pancreatic adenocarcinoma; PCPG: pheochromocytoma and paraganglioma; PRAD: prostate adenocarcinoma; READ: rectum adenocarcinoma; SARC: sarcoma; SKCM: skin cutaneous melanoma; STAD: stomach adenocarcinoma; TGCT: testicular germ cell tumors; THCA: thyroid carcinoma; THYM: thymoma; UCEC: uterine corpus endometrial carcinoma; UCS: uterine carcinosarcoma.

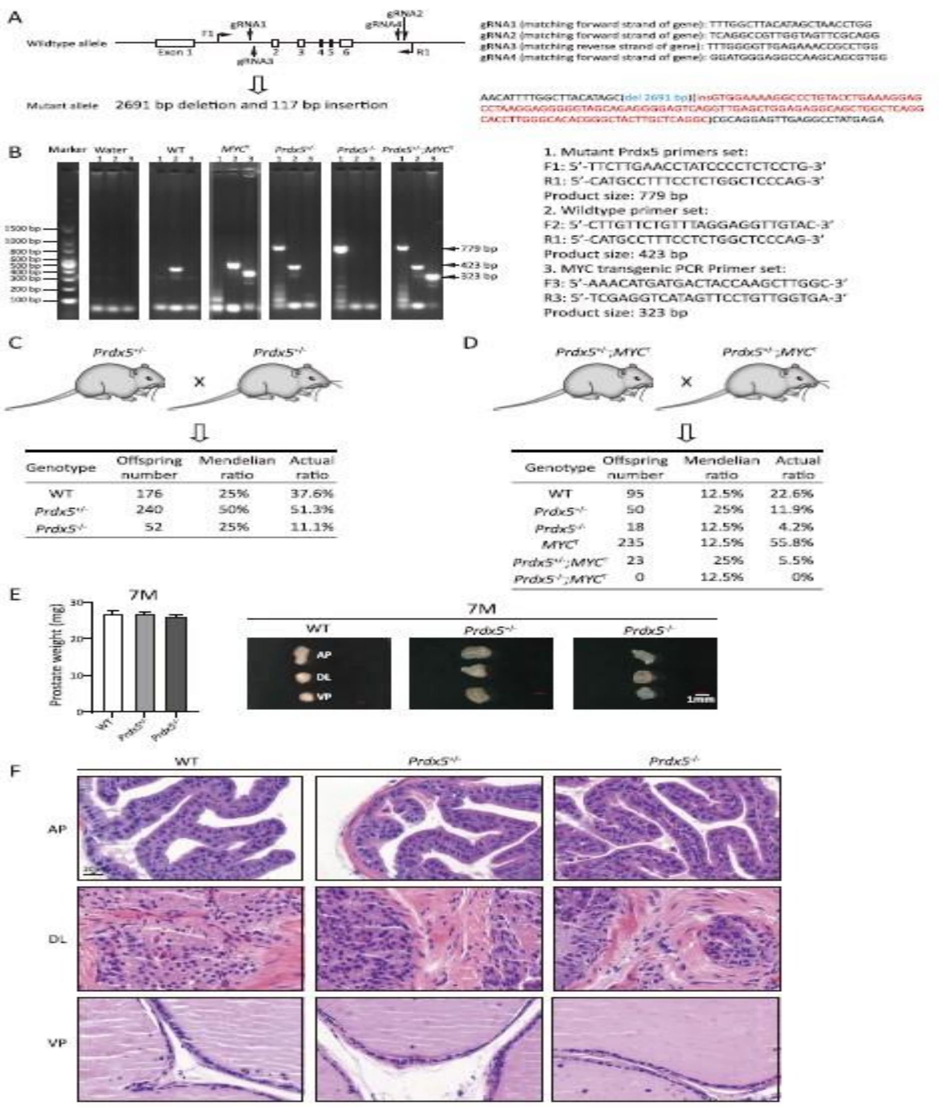

Figure S3

A: Generation of Prdx5 knockout mice. Blue letters represent deleted bases, and red letters represent inserted bases. B: Genotyping of MYCT and Prdx5 KO mice by tail DNA PCR. C: Partial lethality of Prdx5-/- mice. D: Total lethality of Prdx5-/-;MYCT mice. E: Weight and photograph of the prostate from the wild type (WT), Prdx5+/- and Prdx5-/- mice (6 mice per group) at 7 months of age. AP: anterior prostate lobe, DL: dorsolateral prostate lobe, VP: ventral prostate lobe. F: Representative images of prostate histopathology (AP, DL, VP) from WT, Prdx5+/- and Prdx5-/- mice are shown. Prdx5 deletion does not affect mouse prostate development.

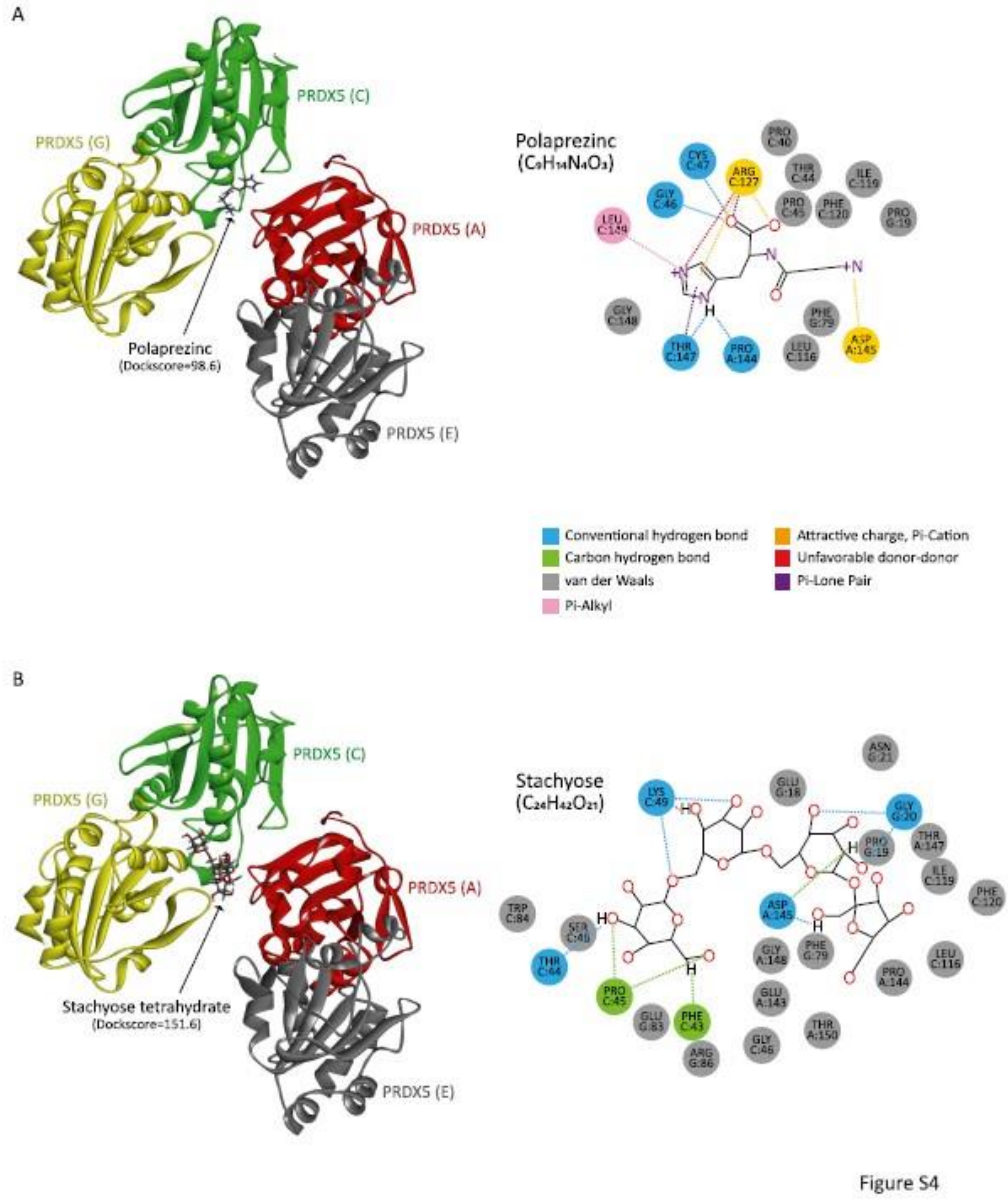

Figure S4

Interaction between PRDX5 protein (4MMM in PDB https://www.rcsb.org/structure/4MMM) and polaprezinc (A) or stachyose tetrahydrate (B) modeled by the Discovery studio software. Dock scores are 98.6 and 151.6 for polaprezinc and stachyose tetrahydrate, respectively.

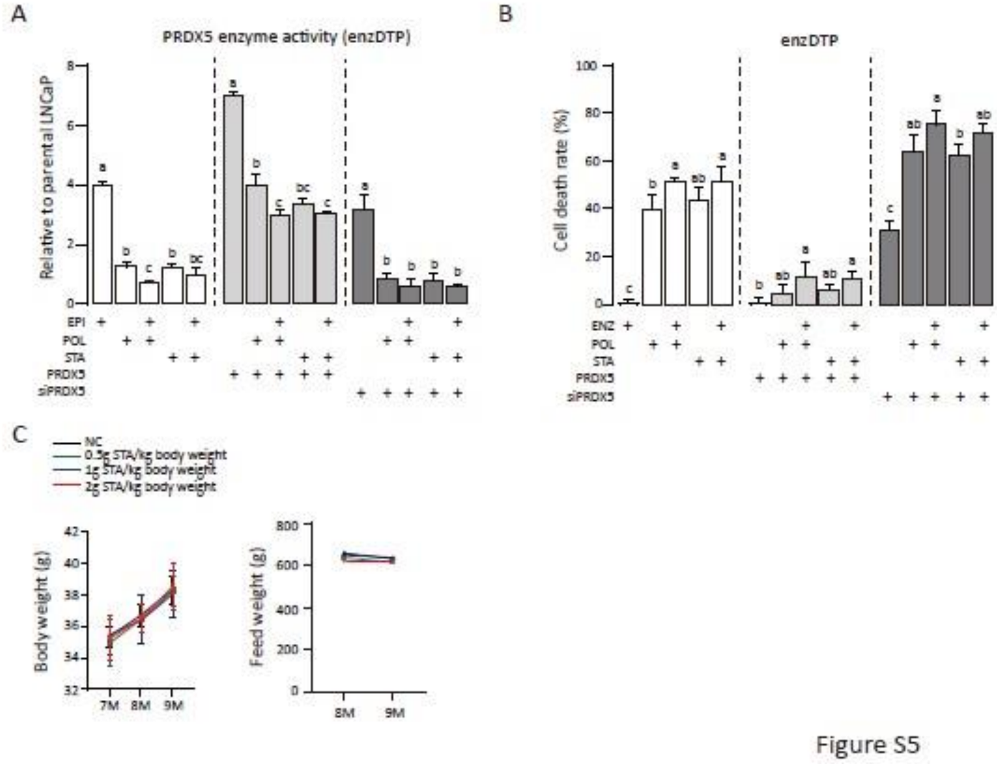

Figure S5

A: PRDX5 activity was measured by enzymatic assay in the presence of POL, STA, ENZ, PRDX5 protein, or siPRDX5 alone or in combination. B: Cell death rate (%) was measured by LDH release assay in the presence of POL, STA, ENZ, PRDX5 protein, or siPRDX5 alone or in combination. Data are expressed as mean ± std of triplicates. One-way ANOVA with the Turkey test was performed. $p<0.05$ was considered significant and indicated by different letters. C: Mice were injected with different concentrations (NC, 0.5 g/kg, 1 g/kg, 2 g/kg, n=6) of STA intravenously at the age of 7 months, and the weight and feed weight (6 mice/cage) of each month were recorded.

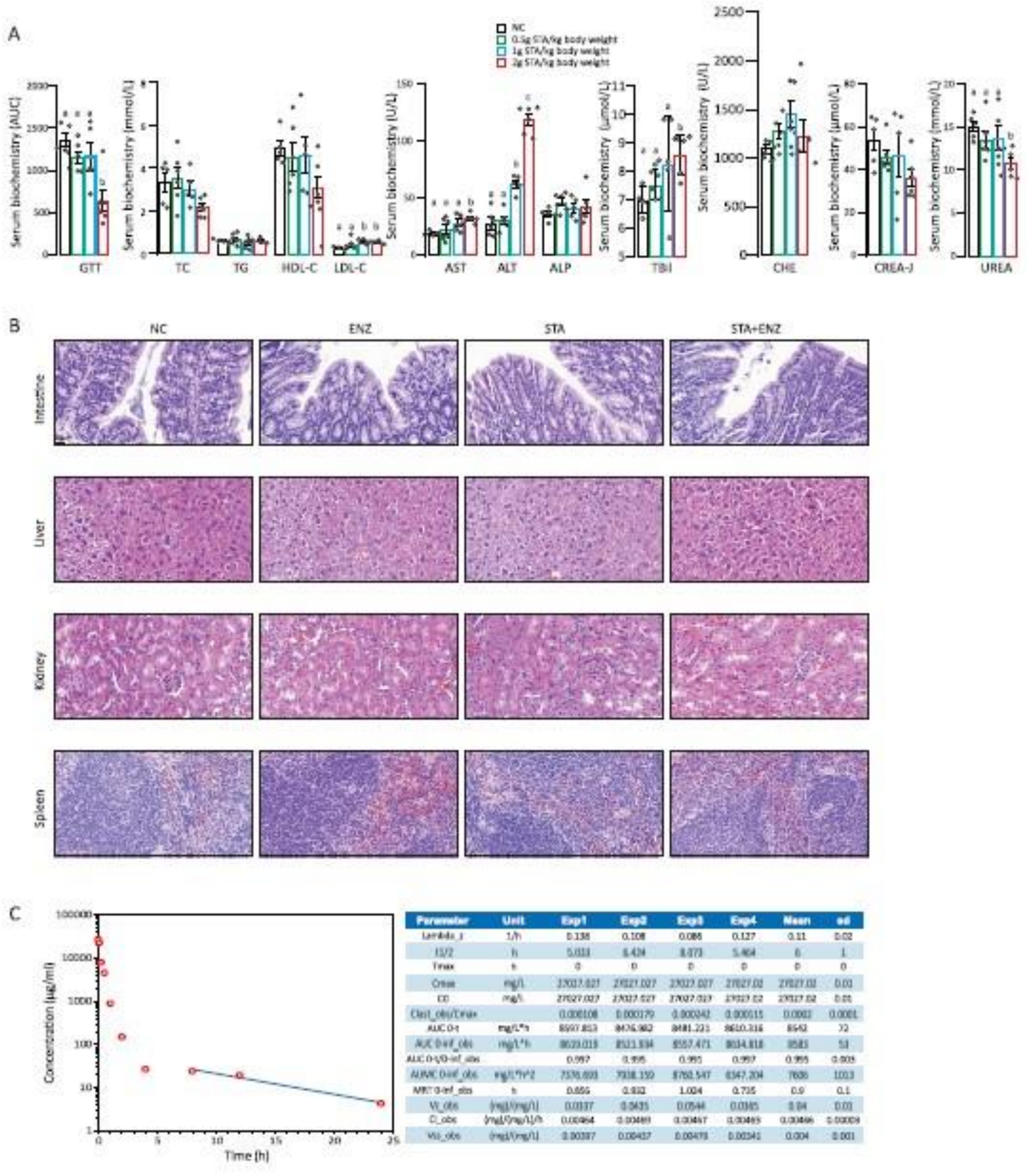

Parameter	Unit	Exp1	Exp2	Exp3	Exp4	Mean	sd
Lambda_z	1/h	0.138	0.108	0.088	0.127	0.11	0.02
t1/2	h	5.033	6.424	8.073	5.464	6	1
Tmax	h	0	0	0	0	0	0
Cmax	mg/L	27027.027	27027.027	27027.027	27027.02	27027.02	0.01
C0	mg/L	27027.027	27027.027	27027.027	27027.02	27027.02	0.01
Clast_obs/Cmax		0.000108	0.000179	0.000242	0.000115	0.0002	0.0001
AUC 0-t	mg/L*h	8597.853	8476.982	8481.221	8610.316	8542	72
AUC 0-inf_obs	mg/L*h	8623.013	8511.534	8557.471	8634.818	8581	53
AUC 0-t/0-inf_obs		0.997	0.995	0.991	0.997	0.995	0.003
AUMC 0-inf_obs	mg/L*h^2	7576.693	7938.159	8760.547	6347.204	7606	1013
MRT 0-inf_obs	h	0.656	0.932	1.024	0.735	0.9	0.1
Vz_obs	(mg)/(mg/L)	0.0337	0.0435	0.0544	0.0365	0.04	0.01
Cl_obs	(mg)/(mg/L)/h	0.00464	0.00469	0.00467	0.00463	0.00466	0.00003
Vss_obs	(mg)/(mg/L)	0.00307	0.00437	0.00478	0.00341	0.004	0.001

Figure S6

Figure S6

A: Serum biochemical parameter analysis in control, treated with 0.5, 1, and 2 g/kg body weight of stachyose (STA). Data are expressed as mean ± std. of triplicates. One-way ANOVA with the Turkey test was performed. $p<0.05$ was considered significant and indicated by different letters. B: Representative histopathological sections images of mouse intestine, liver, kidney, and spleen. Significant differences were observed. C: STA (1 g/kg body weight) was administrated intravenously. Mouse blood was obtained at 5, 15, and 30 minutes and at 1, 2 4, 8, 12, 24, and 48 hours. The serum level of STA was quantified by LC-MS. PD/PK parameters were calculated using

a Non-compartmental analysis of plasma data after the intravenous bolus input model with the PKSolver.

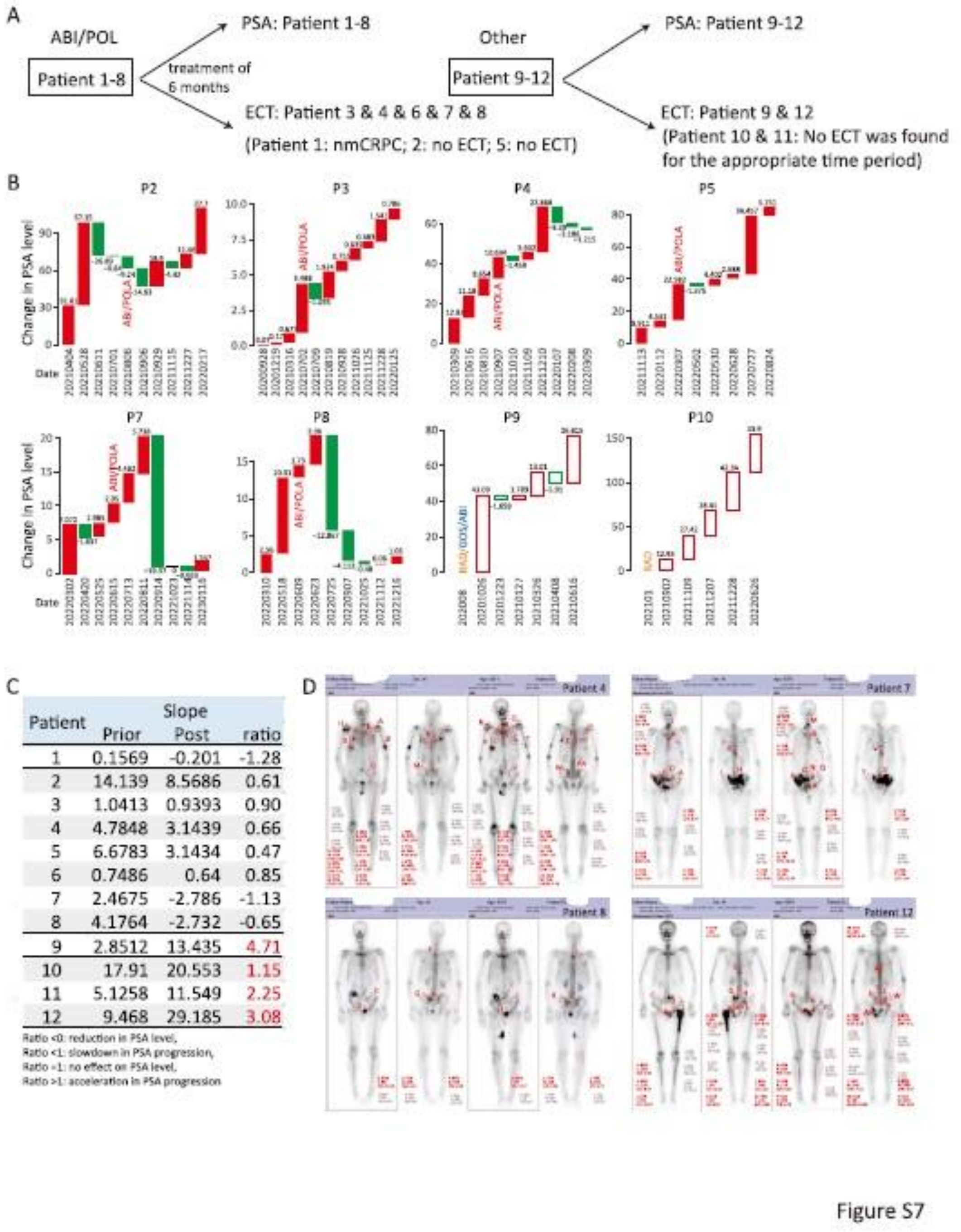

Figure S7

A: Patient group information. B: Waterfall plot of PSA progression in patients treated with ABI+POL (75mg b.i.d, 6 months) (Solid columns) or other treatments (hollow columns). The red and green columns indicate the percentage of increase and decrease in PSA expression, respectively. C: PSA progression in CRPC patients. Slopes were calculated for three PSA measurements prior

and six PSA measurements during treatment. Slope ratios are shown. Ratio <0 means reduction in PSA level, <1 means slowdown in PSA progression, =1 means no effect on PSA level, and>1 means acceleration in PSA progression. Stabilization in PSA was seen in all patients (8/8) treated with the ABI/POL group but not in the other treatment group (4/4). D: Prior and post-treatment ECT images for patients 4, 7, and 8 from the ABI+POL group and patient 12 from the other treatment group. Lesion density was calculated after background subtraction.

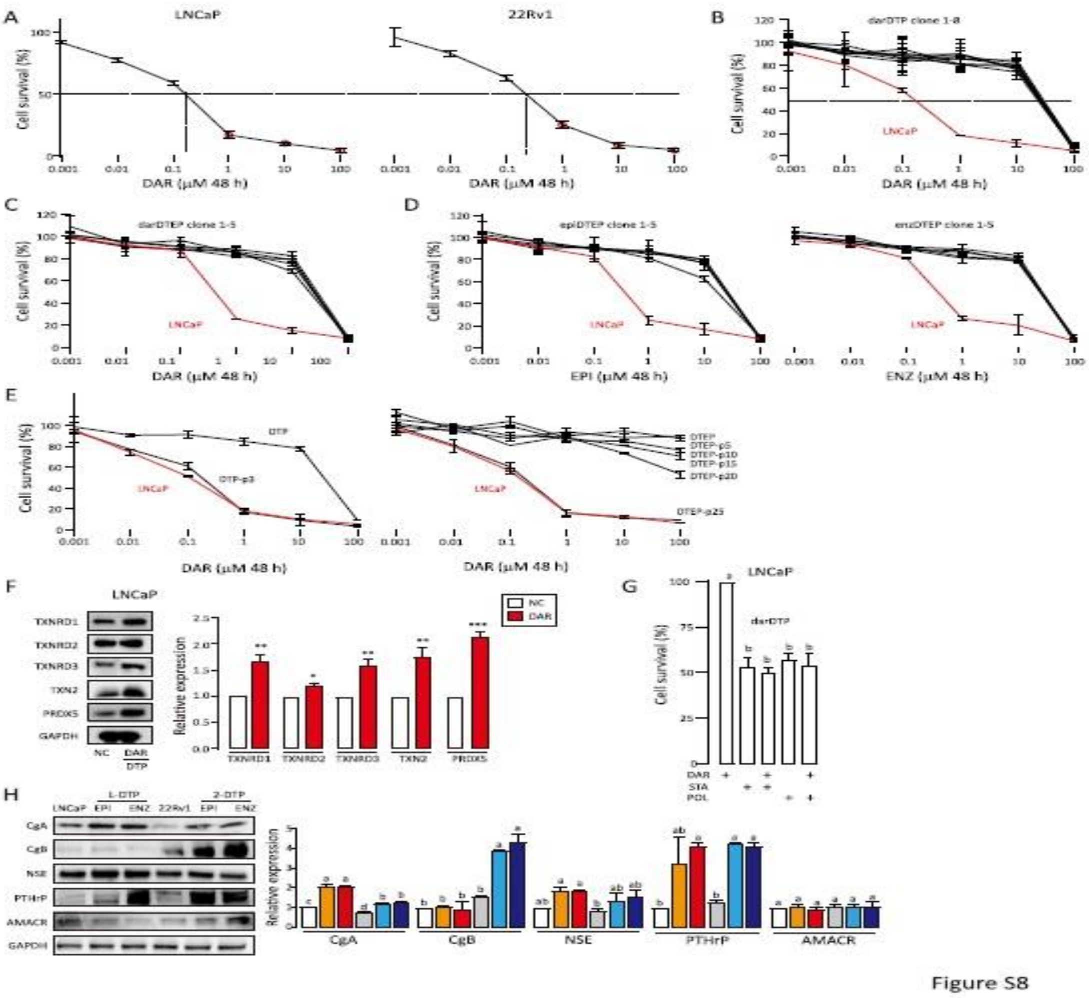

Figure S8

A: LNCaP and 22Rv1 PCa cells were treated with different concentrations (0.001-100 mM) of AR inhibitor darolutamide (DAR) for 48 h. IC50 values were calculated. B: Resistance of darDTP clones to DAR. C: Resistance of darDTEP clones to DAR. D: Resistance of epiDTEP and enzDTEP clones to EPI and ENZ, respectively. E: Reversal of the resistant phenotype after DAR withdrawal. LNCaP DTP and DTEP cells became sensitive to DAR after 3 and 25 passages, respectively. Data were expressed as a percentage of viable cells relative to untreated LNCaP controls as measured by CCK-8 assay and expressed as mean ± std of triplicates. F: Upregulation of TXNRD1, TXNRD2, TXNRD3, TXN2, and PRDX5 as quantified by Western blotting. GAPDH protein was used as the loading control. Student’s t-test was performed, *, **, and ***indicate p<0.05, p<0.01, and

p<0.001, respectively. G: Reduction in darDTP cell survival by POL, STA alone, or in combination with DAR as measured by CCK-8 assay. Data are expressed as mean ± std of triplicates. One-way ANOVA with the Turkey test was performed. p<0.05 was considered significant and indicated by different letters. H: NE-like biomarkers (CgA, CgB, NSE, PTHrP, and AMACR) were detected in LNCaP cells, LNCaP-derived DTPs, 22Rv1 cells, and 22Rv1-derived DTPs by Western blot. GAPDH protein is used as the loading control. Data are expressed as mean ± std of triplicates. One-way ANOVA with the Turkey test was performed. p<0.05 was considered significant and indicated by different letters.

Uncropped scans of the Western blots shown in the indicated figures

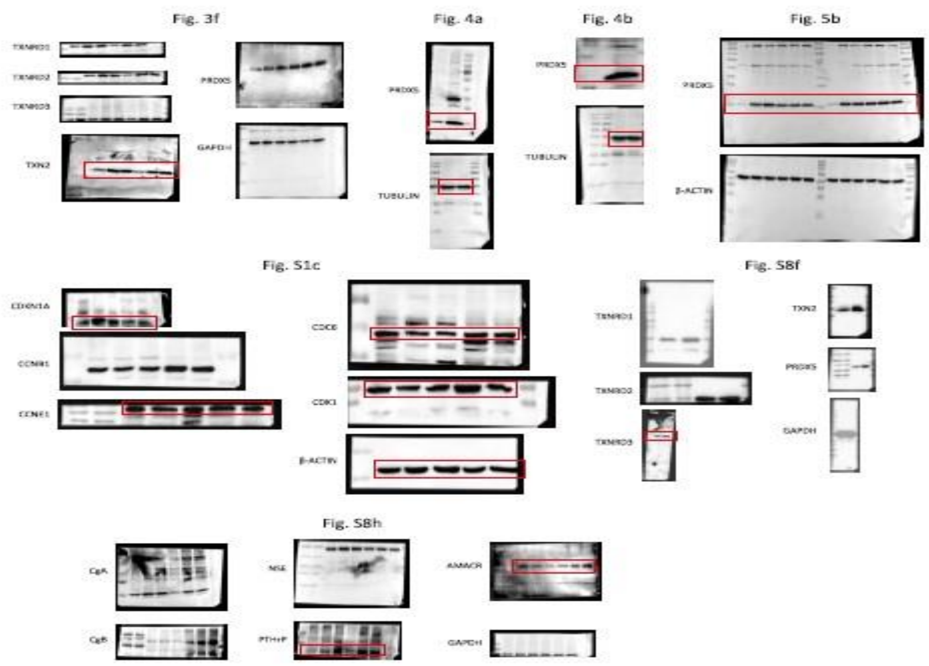

Table S1. Differentially expressed proteins in LNCaP epiDTP cells (>2 fold, p<0.05)

Accession	Gene	Protein description	Ratio	P value
P18463	HLA-B	"HLA class I histocompatibility antigen, B-37 alpha chain OS=Homo sapiens OX=9606 GN=HLA-B"	9.176	1.6282E-05
P55073	DIO3	Thyroxine 5-deiodinase OS=Homo sapiens OX=9606 GN=DIO3	8.605	1.3197E-06
Q14956	GPNMB	Transmembrane glycoprotein NMB OS=Homo sapiens OX=9606 GN=GPNMB	7.36	1.9678E-06
Q0ZGT2	NEXN	Nexilin OS=Homo sapiens OX=9606 GN=NEXN	6.886	2.8237E-06
Q9P1A6	DLGAP2	Disks large-associated protein 2 OS=Homo sapiens OX=9606 GN=DLGAP2	5.825	0.00076141
P25774	CTSS	Cathepsin S OS=Homo sapiens OX=9606 GN=CTSS	5.76	2.1179E-

			8	05
P17693	HLA-G	"HLA class I histocompatibility antigen, alpha chain G OS=Homo sapiens OX=9606 GN=HLA-G"	5.743	1.6681E-06
P08174	CD55	Complement decay-accelerating factor OS=Homo sapiens OX=9606 GN=CD55	5.496	0.00046411
P51648	ALDH3A2	Fatty aldehyde dehydrogenase OS=Homo sapiens OX=9606 GN=ALDH3A2	4.883	4.4394E-06
Q14032	BAAT	Bile acid-CoA:amino acid N-acyltransferase OS=Homo sapiens OX=9606 GN=BAAT	4.593	9.6326E-05
A6NCL1	GMNC	Geminin coiled-coil domain-containing protein 1 OS=Homo sapiens OX=9606 GN=GMNC	4.55	5.9603E-05
P08670	VIM	Vimentin OS=Homo sapiens OX=9606 GN=VIM	4.503	6.4645E-05
P29474	NOS3	"Nitric oxide synthase, endothelial OS=Homo sapiens OX=9606 GN=NOS3"	4.464	2.7533E-07
O95399	UTS2	Urotensin-2 OS=Homo sapiens OX=9606 GN=UTS2	4.439	2.8302E-06
P13501	CCL5	C-C motif chemokine 5 OS=Homo sapiens OX=9606 GN=CCL5	4.41	0.00023634
Q8TA86	RP9	Retinitis pigmentosa 9 protein OS=Homo sapiens OX=9606 GN=RP9	4.391	0.0005408
P08910	ABHD2	Monoacylglycerol lipase ABHD2 OS=Homo sapiens OX=9606 GN=ABHD2	4.307	7.989E-07
O95445	APOM	Apolipoprotein M OS=Homo sapiens OX=9606 GN=APOM	4.236	4.1471E-05
Q16831	UPP1	Uridine phosphorylase 1 OS=Homo sapiens OX=9606 GN=UPP1	4.2	3.5973E-05
P04179	SOD2	"Superoxide dismutase [Mn], mitochondrial OS=Homo sapiens OX=9606 GN=SOD2"	4.176	1.5542E-05
Q9BX59	TAPBPL	Tapasin-related protein OS=Homo sapiens OX=9606 GN=TAPBPL	3.693	2.1755E-05
P61769	B2M	Beta-2-microglobulin OS=Homo sapiens OX=9606 GN=B2M	3.676	1.4584E-06
P10909	CLU	Clusterin OS=Homo sapiens OX=9606 GN=CLU	3.675	3.5226E-05
O43761	SYNGR3	Synaptogyrin-3 OS=Homo sapiens OX=9606 GN=SYNGR3	3.568	0.00041965
Q99685	MGLL	Monoglyceride lipase OS=Homo sapiens OX=9606 GN=MGLL	3.528	0.0041387
Q4W5N1	ABCA11P	Putative ATP-binding cassette sub-family A member 11 OS=Homo sapiens OX=9606 GN=ABCA11P	3.493	0.001264
P11021	HSPA5	Endoplasmic reticulum chaperone BiP OS=Homo sapiens OX=9606 GN=HSPA5	3.471	1.234E-06
Q5T890	ERCC6L2	DNA excision repair protein ERCC-6-like 2 OS=Homo sapiens OX=9606 GN=ERCC6L2	3.457	0.0098219
Q6ZSB9	ZBTB49	Zinc finger and BTB domain-containing protein 49 OS=Homo sapiens OX=9606 GN=ZBTB49	3.429	0.00006014
Q9BY49	PECR	Peroxisomal trans-2-enoyl-CoA reductase OS=Homo sapiens OX=9606 GN=PECR	3.426	2.9881E-07
P38935	IGHMBP2	DNA-binding protein SMUBP-2 OS=Homo sapiens OX=9606 GN=IGHMBP2	3.412	0.00016069
O15533	TAPBP	Tapasin OS=Homo sapiens OX=9606 GN=TAPBP	3.40	1.5643E-

			6	06
Q6IPR1	ETFRF1	Electron transfer flavoprotein regulatory factor 1 OS=Homo sapiens OX=9606 GN=ETFRF1	3.396	0.0065598
Q9Y5U8	MPC1	Mitochondrial pyruvate carrier 1 OS=Homo sapiens OX=9606 GN=MPC1	3.368	3.1191E-07
A0A0U1RRL7	MMP24OS	Protein MMP24OS OS=Homo sapiens OX=9606 GN=MMP24OS	3.344	0.0088219
Q15714	TSC22D1	TSC22 domain family protein 1 OS=Homo sapiens OX=9606 GN=TSC22D1	3.3	2.1195E-05
Q99988	GDF15	Growth/differentiation factor 15 OS=Homo sapiens OX=9606 GN=GDF15	3.255	2.0863E-05
Q9UJF2	RASAL2	Ras GTPase-activating protein nGAP OS=Homo sapiens OX=9606 GN=RASAL2	3.244	3.8965E-05
P78556	CCL20	C-C motif chemokine 20 OS=Homo sapiens OX=9606 GN=CCL20	3.24	4.6609E-06
P42330	AKR1C3	Aldo-keto reductase family 1 member C3 OS=Homo sapiens OX=9606 GN=AKR1C3	3.15	0.0055438
Q14520	HABP2	Hyaluronan-binding protein 2 OS=Homo sapiens OX=9606 GN=HABP2	3.143	0.00013802
O00391	QSOX1	Sulfhydryl oxidase 1 OS=Homo sapiens OX=9606 GN=QSOX1	3.127	4.8826E-06
P58004	SESN2	Sestrin-2 OS=Homo sapiens OX=9606 GN=SESN2	3.118	1.0128E-06
Q9UBS3	DNAJB9	DnaJ homolog subfamily B member 9 OS=Homo sapiens OX=9606 GN=DNAJB9	3.091	4.3071E-05
O75445	USH2A	Usherin OS=Homo sapiens OX=9606 GN=USH2A	3.05	0.00010107
Q9Y5F3	PCDHB1	Protocadherin beta-1 OS=Homo sapiens OX=9606 GN=PCDHB1	3.011	5.5726E-05
P00742	F10	Coagulation factor X OS=Homo sapiens OX=9606 GN=F10	3.009	7.5168E-05
Q8IYK2	CCDC105	Coiled-coil domain-containing protein 105 OS=Homo sapiens OX=9606 GN=CCDC105	3.009	0.00017986
P06396	GSN	Gelsolin OS=Homo sapiens OX=9606 GN=GSN	2.97	1.5342E-05
P05090	APOD	Apolipoprotein D OS=Homo sapiens OX=9606 GN=APOD	2.955	0.0030578
P0C221	CCDC175	Coiled-coil domain-containing protein 175 OS=Homo sapiens OX=9606 GN=CCDC175	2.949	0.00021534
Q9NZ08	ERAP1	Endoplasmic reticulum aminopeptidase 1 OS=Homo sapiens OX=9606 GN=ERAP1	2.933	1.6503E-05
Q9NUQ2	AGPAT5	1-acyl-sn-glycerol-3-phosphate acyltransferase epsilon OS=Homo sapiens OX=9606 GN=AGPAT5	2.927	0.00014265
Q9P246	STIM2	Stromal interaction molecule 2 OS=Homo sapiens OX=9606 GN=STIM2	2.911	0.0069167
P61225	RAP2B	Ras-related protein Rap-2b OS=Homo sapiens OX=9606 GN=RAP2B	2.901	0.028481
Q8N4N3	KLHL36	Kelch-like protein 36 OS=Homo sapiens OX=9606 GN=KLHL36	2.896	0.0017611
O60613	SELENOF	Selenoprotein F OS=Homo sapiens OX=9606 GN=SELENOF	2.887	3.8676E-05
Q96HE7	ERO1A	ERO1-like protein alpha OS=Homo sapiens OX=9606	2.87	1.9675E-

		GN=ERO1A	6	05
O95613	PCNT	Pericentrin OS=Homo sapiens OX=9606 GN=PCNT	2.871	0.02882
Q8N357	SLC35F6	Solute carrier family 35 member F6 OS=Homo sapiens OX=9606 GN=SLC35F6	2.849	5.7417E-05
A0A0B4J266	TRAV41	T cell receptor alpha variable 41 OS=Homo sapiens OX=9606 GN=TRAV41	2.848	0.00123891
Q96FN4	CPNE2	Copine-2 OS=Homo sapiens OX=9606 GN=CPNE2	2.838	0.00018398
Q86TM3	DDX53	Probable ATP-dependent RNA helicase DDX53 OS=Homo sapiens OX=9606 GN=DDX53	2.8	0.00051649
O14773	TPP1	Tripeptidyl-peptidase 1 OS=Homo sapiens OX=9606 GN=TPP1	2.795	3.0512E-06
Q96QU1	PCDH15	Protocadherin-15 OS=Homo sapiens OX=9606 GN=PCDH15	2.784	4.3231E-05
Q460N5	PARP14	Poly [ADP-ribose] polymerase 14 OS=Homo sapiens OX=9606 GN=PARP14	2.782	4.0276E-06
Q9H190	SDCBP2	Syntenin-2 OS=Homo sapiens OX=9606 GN=SDCBP2	2.78	9.6003E-05
A2RUB1	MEIOC	Meiosis-specific coiled-coil domain-containing protein MEIOC OS=Homo sapiens OX=9606 GN=MEIOC	2.762	0.00026122
O95858	TSPAN15	Tetraspanin-15 OS=Homo sapiens OX=9606 GN=TSPAN15	2.755	0.00015758
P80188	LCN2	Neutrophil gelatinase-associated lipocalin OS=Homo sapiens OX=9606 GN=LCN2	2.728	4.1851E-07
Q9NRI5	DISC1	Disrupted in schizophrenia 1 protein OS=Homo sapiens OX=9606 GN=DISC1	2.728	2.4235E-05
Q9H093	NUAK2	NUAK family SNF1-like kinase 2 OS=Homo sapiens OX=9606 GN=NUAK2	2.721	0.00011602
P10242	MYB	Transcriptional activator Myb OS=Homo sapiens OX=9606 GN=MYB	2.72	1.6698E-06
Q9BX68	HINT2	"Histidine triad nucleotide-binding protein 2, mitochondrial OS=Homo sapiens OX=9606 GN=HINT2"	2.714	2.4067E-06
P80365	HSD11B2	Corticosteroid 11-beta-dehydrogenase isozyme 2 OS=Homo sapiens OX=9606 GN=HSD11B2	2.693	1.5991E-05
P31512	FMO4	Dimethylaniline monooxygenase [N-oxide-forming] 4 OS=Homo sapiens OX=9606 GN=FMO4	2.692	0.0039606
Q9BY50	SEC11C	Signal peptidase complex catalytic subunit SEC11C OS=Homo sapiens OX=9606 GN=SEC11C	2.679	1.9224E-06
Q9UJY1	HSPB8	Heat shock protein beta-8 OS=Homo sapiens OX=9606 GN=HSPB8	2.665	2.1445E-05
A8MSI8	LYRM9	LYR motif-containing protein 9 OS=Homo sapiens OX=9606 GN=LYRM9	2.657	0.00002068
Q5U649	C12orf60	Uncharacterized protein C12orf60 OS=Homo sapiens OX=9606 GN=C12orf60	2.645	0.00099999
P32455	GBP1	Guanylate-binding protein 1 OS=Homo sapiens OX=9606 GN=GBP1	2.629	5.9423E-05
Q5TB80	CEP162	Centrosomal protein of 162 kDa OS=Homo sapiens OX=9606 GN=CEP162	2.62	0.00010494
P28289	TMOD1	Tropomodulin-1 OS=Homo sapiens OX=9606 GN=TMOD1	2.62	0.0127189

Q96A73	KIAA1191	Putative monooxygenase p33MONOX OS=Homo sapiens OX=9606 GN=KIAA1191	2.617	4.2134E-05
Q38SD2	LRRK1	Leucine-rich repeat serine/threonine-protein kinase 1 OS=Homo sapiens OX=9606 GN=LRRK1	2.61	0.000636
Q8N9Z2	CCDC71L	Coiled-coil domain-containing protein 71L OS=Homo sapiens OX=9606 GN=CCDC71L	2.604	0.00027758
Q9UNT1	RABL2B	Rab-like protein 2B OS=Homo sapiens OX=9606 GN=RABL2B	2.597	2.0869E-05
Q8NEV8	EXPH5	Exophilin-5 OS=Homo sapiens OX=9606 GN=EXPH5	2.596	8.1165E-05
O60711	LPXN	Leupaxin OS=Homo sapiens OX=9606 GN=LPXN	2.593	2.0507E-05
Q86SR1	GALNT10	Polypeptide N-acetylgalactosaminyltransferase 10 OS=Homo sapiens OX=9606 GN=GALNT10	2.593	6.0124E-05
O00462	MANBA	Beta-mannosidase OS=Homo sapiens OX=9606 GN=MANBA	2.59	0.00004018
O75911	DHRS3	Short-chain dehydrogenase/reductase 3 OS=Homo sapiens OX=9606 GN=DHRS3	2.58	0.00064302
P15586	GNS	N-acetylglucosamine-6-sulfatase OS=Homo sapiens OX=9606 GN=GNS	2.578	1.5142E-05
O15484	CAPN5	Calpain-5 OS=Homo sapiens OX=9606 GN=CAPN5	2.575	1.7483E-05
P10321	HLA-C	"HLA class I histocompatibility antigen, Cw-7 alpha chain OS=Homo sapiens OX=9606 GN=HLA-C"	2.571	0.0171601
Q9BZQ8	FAM129A	Protein Niban OS=Homo sapiens OX=9606 GN=FAM129A	2.565	0.00011543
P14784	IL2RB	Interleukin-2 receptor subunit beta OS=Homo sapiens OX=9606 GN=IL2RB	2.562	0.0172048
A6NNW6	ENO4	Enolase 4 OS=Homo sapiens OX=9606 GN=ENO4	2.559	0.00036008
Q9UHN6	TMEM2	Cell surface hyaluronidase OS=Homo sapiens OX=9606 GN=TMEM2	2.558	1.6827E-05
Q16706	MAN2A1	Alpha-mannosidase 2 OS=Homo sapiens OX=9606 GN=MAN2A1	2.552	4.0451E-05
P31327	CPS1	"Carbamoyl-phosphate synthase [ammonia], mitochondrial OS=Homo sapiens OX=9606 GN=CPS1"	2.542	2.1015E-06
Q6KCM7	SLC25A25	Calcium-binding mitochondrial carrier protein SCaMC-2 OS=Homo sapiens OX=9606 GN=SLC25A25	2.54	2.9333E-06
O75891	ALDH1L1	Cytosolic 10-formyltetrahydrofolate dehydrogenase OS=Homo sapiens OX=9606 GN=ALDH1L1	2.537	4.1529E-05
P15289	ARSA	Arylsulfatase A OS=Homo sapiens OX=9606 GN=ARSA	2.534	0.00025832
Q9NU19	TBC1D22B	TBC1 domain family member 22B OS=Homo sapiens OX=9606 GN=TBC1D22B	2.524	6.4855E-05
P46821	MAP1B	Microtubule-associated protein 1B OS=Homo sapiens OX=9606 GN=MAP1B	2.523	0.0068229
Q96QB1	DLC1	Rho GTPase-activating protein 7 OS=Homo sapiens OX=9606 GN=DLC1	2.517	0.00021903
P07099	EPHX1	Epoxide hydrolase 1 OS=Homo sapiens OX=9606 GN=EPHX1	2.517	0.00027868

O00622	CYR61	Protein CYR61 OS=Homo sapiens OX=9606 GN=CYR61	2.514	0.00015562
P02511	CRYAB	Alpha-crystallin B chain OS=Homo sapiens OX=9606 GN=CRYAB	2.514	0.00030381
Q9NP80	PNPLA8	Calcium-independent phospholipase A2-gamma OS=Homo sapiens OX=9606 GN=PNPLA8	2.512	2.1299E-05
P08195	SLC3A2	4F2 cell-surface antigen heavy chain OS=Homo sapiens OX=9606 GN=SLC3A2	2.511	2.4526E-06
P26006	ITGA3	Integrin alpha-3 OS=Homo sapiens OX=9606 GN=ITGA3	2.504	2.9935E-06
Q9UDR5	AASS	"Alpha-aminoadipic semialdehyde synthase, mitochondrial OS=Homo sapiens OX=9606 GN=AASS"	2.502	1.5811E-05
Q71SY5	MED25	Mediator of RNA polymerase II transcription subunit 25 OS=Homo sapiens OX=9606 GN=MED25	2.498	0.00002177
Q5JTV8	TOR1AIP1	Torsin-1A-interacting protein 1 OS=Homo sapiens OX=9606 GN=TOR1AIP1	2.493	3.523E-06
P10301	RRAS	Ras-related protein R-Ras OS=Homo sapiens OX=9606 GN=RRAS	2.491	4.0311E-05
Q99536	VAT1	Synaptic vesicle membrane protein VAT-1 homolog OS=Homo sapiens OX=9606 GN=VAT1	2.484	0.0009788
P13533	MYH6	Myosin-6 OS=Homo sapiens OX=9606 GN=MYH6	2.477	6.3778E-05
Q8WVV4	POF1B	Protein POF1B OS=Homo sapiens OX=9606 GN=POF1B	2.463	9.7903E-05
O14967	CLGN	Calmegin OS=Homo sapiens OX=9606 GN=CLGN	2.453	2.0575E-05
P23497	SP100	Nuclear autoantigen Sp-100 OS=Homo sapiens OX=9606 GN=SP100	2.453	7.7485E-05
Q6UWY5	OLFML1	Olfactomedin-like protein 1 OS=Homo sapiens OX=9606 GN=OLFML1	2.452	0.0004594
Q9H930	SP140L	Nuclear body protein SP140-like protein OS=Homo sapiens OX=9606 GN=SP140L	2.445	7.7833E-05
Q9ULV0	MYO5B	Unconventional myosin-Vb OS=Homo sapiens OX=9606 GN=MYO5B	2.444	1.8138E-05
Q9ULM6	CNOT6	CCR4-NOT transcription complex subunit 6 OS=Homo sapiens OX=9606 GN=CNOT6	2.438	1.9723E-05
Q9Y623	MYH4	Myosin-4 OS=Homo sapiens OX=9606 GN=MYH4	2.438	0.00021913
Q9UM22	EPDR1	Mammalian ependymin-related protein 1 OS=Homo sapiens OX=9606 GN=EPDR1	2.438	0.00101854
Q08AE8	SPIRE1	Protein spire homolog 1 OS=Homo sapiens OX=9606 GN=SPIRE1	2.436	3.1664E-06
O95210	STBD1	Starch-binding domain-containing protein 1 OS=Homo sapiens OX=9606 GN=STBD1	2.433	3.9218E-06
B2RUZ4	SMIM1	Small integral membrane protein 1 OS=Homo sapiens OX=9606 GN=SMIM1	2.432	0.00088155
Q9Y2Q3	GSTK1	Glutathione S-transferase kappa 1 OS=Homo sapiens OX=9606 GN=GSTK1	2.423	2.2544E-05
P07305	H1F0	Histone H1.0 OS=Homo sapiens OX=9606 GN=H1F0	2.416	0.023198
Q96QI5	HS3ST6	Heparan sulfate glucosamine 3-O-sulfotransferase 6	2.41	5.5342E-

		OS=Homo sapiens OX=9606 GN=HS3ST6	3	05
Q9GZT6	CCDC90B	"Coiled-coil domain-containing protein 90B, mitochondrial OS=Homo sapiens OX=9606 GN=CCDC90B"	2.413	0.0029173
Q6PI78	TMEM65	Transmembrane protein 65 OS=Homo sapiens OX=9606 GN=TMEM65	2.409	7.6592E-05
Q9NUM4	TMEM106B	Transmembrane protein 106B OS=Homo sapiens OX=9606 GN=TMEM106B	2.399	3.8245E-05
P11532	DMD	Dystrophin OS=Homo sapiens OX=9606 GN=DMD	2.394	0.00099795
Q9UJA9	ENPP5	Ectonucleotide pyrophosphatase/phosphodiesterase family member 5 OS=Homo sapiens OX=9606 GN=ENPP5	2.392	3.9604E-05
Q7Z3D6	DGLUCY	"D-glutamate cyclase, mitochondrial OS=Homo sapiens OX=9606 GN=DGLUCY"	2.391	2.2965E-06
P53804	TTC3	E3 ubiquitin-protein ligase TTC3 OS=Homo sapiens OX=9606 GN=TTC3	2.391	0.00011674
Q9BZQ2	SHCBP1L	Testicular spindle-associated protein SHCBP1L OS=Homo sapiens OX=9606 GN=SHCBP1L	2.39	0.0146818
P39210	MPV17	Protein Mpv17 OS=Homo sapiens OX=9606 GN=MPV17	2.387	0.00044311
Q8IYJ3	SYTL1	Synaptotagmin-like protein 1 OS=Homo sapiens OX=9606 GN=SYTL1	2.381	0.0006379
Q9UMX3	BOK	Bcl-2-related ovarian killer protein OS=Homo sapiens OX=9606 GN=BOK	2.378	0.00039893
P07858	CTSB	Cathepsin B OS=Homo sapiens OX=9606 GN=CTSB	2.374	1.8153E-05
Q5JUK3	KCNT1	Potassium channel subfamily T member 1 OS=Homo sapiens OX=9606 GN=KCNT1	2.373	0.00100482
P09493	TPM1	Tropomyosin alpha-1 chain OS=Homo sapiens OX=9606 GN=TPM1	2.371	1.6944E-05
Q9Y6N5	SQOR	"Sulfide:quinone oxidoreductase, mitochondrial OS=Homo sapiens OX=9606 GN=SQOR"	2.369	0.00001941
Q6ZTQ3	RASSF6	Ras association domain-containing protein 6 OS=Homo sapiens OX=9606 GN=RASSF6	2.368	0.00022003
P28288	ABCD3	ATP-binding cassette sub-family D member 3 OS=Homo sapiens OX=9606 GN=ABCD3	2.367	1.6374E-05
O14832	PHYH	"Phytanoyl-CoA dioxygenase, peroxisomal OS=Homo sapiens OX=9606 GN=PHYH"	2.366	9.2492E-07
Q04828	AKR1C1	Aldo-keto reductase family 1 member C1 OS=Homo sapiens OX=9606 GN=AKR1C1	2.363	1.6365E-05
Q5T9C2	FAM102A	Protein FAM102A OS=Homo sapiens OX=9606 GN=FAM102A	2.361	2.8083E-06
Q8NA47	CCDC63	Coiled-coil domain-containing protein 63 OS=Homo sapiens OX=9606 GN=CCDC63	2.361	0.00108195
O95425	SVIL	Supervillin OS=Homo sapiens OX=9606 GN=SVIL	2.356	2.0476E-05
P05165	PCCA	"Propionyl-CoA carboxylase alpha chain, mitochondrial OS=Homo sapiens OX=9606 GN=PCCA"	2.351	6.1369E-07
Q9Y646	CPQ	Carboxypeptidase Q OS=Homo sapiens OX=9606 GN=CPQ	2.347	3.5358E-05

P35914	HMGCL	"Hydroxymethylglutaryl-CoA lyase, mitochondrial OS=Homo sapiens OX=9606 GN=HMGCL"	2.341	1.8044E-05
P10619	CTSA	Lysosomal protective protein OS=Homo sapiens OX=9606 GN=CTSA	2.341	2.3602E-05
Q96DA2	RAB39B	Ras-related protein Rab-39B OS=Homo sapiens OX=9606 GN=RAB39B	2.339	4.6146E-07
Q9NRM1	ENAM	Enamelin OS=Homo sapiens OX=9606 GN=ENAM	2.33	0.00004293
P19087	GNAT2	Guanine nucleotide-binding protein G(t) subunit alpha-2 OS=Homo sapiens OX=9606 GN=GNAT2	2.33	0.00035623
P08913	ADRA2A	Alpha-2A adrenergic receptor OS=Homo sapiens OX=9606 GN=ADRA2A	2.328	0.00068311
Q96FZ7	CHMP6	Charged multivesicular body protein 6 OS=Homo sapiens OX=9606 GN=CHMP6	2.326	0.00063818
Q16822	PCK2	"Phosphoenolpyruvate carboxykinase [GTP], mitochondrial OS=Homo sapiens OX=9606 GN=PCK2"	2.324	5.7137E-07
Q9BSH5	HDHD3	Haloacid dehalogenase-like hydrolase domain-containing protein 3 OS=Homo sapiens OX=9606 GN=HDHD3	2.322	2.0553E-05
P55084	HADHB	"Trifunctional enzyme subunit beta, mitochondrial OS=Homo sapiens OX=9606 GN=HADHB"	2.32	1.6119E-05
P05166	PCCB	"Propionyl-CoA carboxylase beta chain, mitochondrial OS=Homo sapiens OX=9606 GN=PCCB"	2.317	3.9232E-05
Q9HB03	ELOVL3	Elongation of very long chain fatty acids protein 3 OS=Homo sapiens OX=9606 GN=ELOVL3	2.317	0.0058608
Q9NQE9	HINT3	Histidine triad nucleotide-binding protein 3 OS=Homo sapiens OX=9606 GN=HINT3	2.316	2.1076E-05
Q96AQ6	PBXIP1	Pre-B-cell leukemia transcription factor-interacting protein 1 OS=Homo sapiens OX=9606 GN=PBXIP1	2.313	0.00006435
P48740	MASP1	Mannan-binding lectin serine protease 1 OS=Homo sapiens OX=9606 GN=MASP1	2.306	0.00115944
Q8WU67	ABHD3	Phospholipase ABHD3 OS=Homo sapiens OX=9606 GN=ABHD3	2.303	2.1262E-05
Q9UFN0	NIPSNAP3A	Protein NipSnap homolog 3A OS=Homo sapiens OX=9606 GN=NIPSNAP3A	2.301	0.00063918
Q13510	ASAH1	Acid ceramidase OS=Homo sapiens OX=9606 GN=ASAH1	2.296	3.0219E-06
Q9HD67	MYO10	Unconventional myosin-X OS=Homo sapiens OX=9606 GN=MYO10	2.295	0.00046352
P07339	CTSD	Cathepsin D OS=Homo sapiens OX=9606 GN=CTSD	2.292	9.4155E-07
O75363	BCAS1	Breast carcinoma-amplified sequence 1 OS=Homo sapiens OX=9606 GN=BCAS1	2.283	3.9441E-06
P06865	HEXA	Beta-hexosaminidase subunit alpha OS=Homo sapiens OX=9606 GN=HEXA	2.283	2.1879E-05
Q9NUT2	ABCB8	"ATP-binding cassette sub-family B member 8, mitochondrial OS=Homo sapiens OX=9606 GN=ABCB8"	2.282	1.7721E-05
Q93070	ART4	Ecto-ADP-ribosyltransferase 4 OS=Homo sapiens OX=9606 GN=ART4	2.278	0.0140561
P30455	HLA-A	"HLA class I histocompatibility antigen, A-36 alpha	2.27	0.0002028

		chain OS=Homo sapiens OX=9606 GN=HLA-A"	6	1
Q8IZ41	RASEF	Ras and EF-hand domain-containing protein OS=Homo sapiens OX=9606 GN=RASEF	2.27 6	0.0003014 4
P45877	PPIC	Peptidyl-prolyl cis-trans isomerase C OS=Homo sapiens OX=9606 GN=PPIC	2.27 4	0.0043043
Q6EKJ0	GTF2IRD 2B	General transcription factor II-I repeat domain-containing protein 2B OS=Homo sapiens OX=9606 GN=GTF2IRD2B	2.27 2	0.0008378
Q92574	TSC1	Hamartin OS=Homo sapiens OX=9606 GN=TSC1	2.26	0.0010424 7
P17301	ITGA2	Integrin alpha-2 OS=Homo sapiens OX=9606 GN=ITGA2	2.25 4	0.0002198 3
P40939	HADHA	"Trifunctional enzyme subunit alpha, mitochondrial OS=Homo sapiens OX=9606 GN=HADHA"	2.25 2	0.0003583 1
Q04837	SSBP1	"Single-stranded DNA-binding protein, mitochondrial OS=Homo sapiens OX=9606 GN=SSBP1"	2.25 1	0.0000621 4
Q9NSD4	ZNF275	Zinc finger protein 275 OS=Homo sapiens OX=9606 GN=ZNF275	2.25	0.0097216
P39060	COL18A1	Collagen alpha-1(XVIII) chain OS=Homo sapiens OX=9606 GN=COL18A1	2.24 7	0.0002398 4
P28065	PSMB9	Proteasome subunit beta type-9 OS=Homo sapiens OX=9606 GN=PSMB9	2.24 6	3.5054E-05
Q8NHH 1	TTLL11	Tubulin polyglutamylase TTLL11 OS=Homo sapiens OX=9606 GN=TTLL11	2.24 5	0.0001386 7
Q14764	MVP	Major vault protein OS=Homo sapiens OX=9606 GN=MVP	2.24 3	8.2958E-07
Q96CV9	OPTN	Optineurin OS=Homo sapiens OX=9606 GN=OPTN	2.24 3	0.0020014
P31937	HIBADH	"3-hydroxyisobutyrate dehydrogenase, mitochondrial OS=Homo sapiens OX=9606 GN=HIBADH"	2.23 5	6.0017E-05
Q9NTX5	ECHDC1	Ethylmalonyl-CoA decarboxylase OS=Homo sapiens OX=9606 GN=ECHDC1	2.22 5	0.029139
Q9BXK 5	BCL2L13	Bcl-2-like protein 13 OS=Homo sapiens OX=9606 GN=BCL2L13	2.22 3	5.009E-07
P51688	SGSH	N-sulphoglucosamine sulphohydrolase OS=Homo sapiens OX=9606 GN=SGSH	2.21 7	0.0014960 5
Q01459	CTBS	Di-N-acetylchitobiase OS=Homo sapiens OX=9606 GN=CTBS	2.21 6	0.0001775 6
P36776	LONP1	"Lon protease homolog, mitochondrial OS=Homo sapiens OX=9606 GN=LONP1"	2.21 3	7.7225E-07
Q96HH9	GRAMD2 B	GRAM domain-containing protein 2B OS=Homo sapiens OX=9606 GN=GRAMD2B	2.20 3	0.044758
P05783	KRT18	"Keratin, type I cytoskeletal 18 OS=Homo sapiens OX=9606 GN=KRT18"	2.20 2	1.9571E-07
O15320	CTAGE5	Endoplasmic reticulum export factor CTAGE5 OS=Homo sapiens OX=9606 GN=CTAGE5	2.2	4.4612E-06
Q16610	ECM1	Extracellular matrix protein 1 OS=Homo sapiens OX=9606 GN=ECM1	2.2	0.0018986 5
O94933	SLITRK3	SLIT and NTRK-like protein 3 OS=Homo sapiens OX=9606 GN=SLITRK3	2.19 9	0.0001422 6
Q9P0P8	C6orf203	Uncharacterized protein C6orf203 OS=Homo sapiens OX=9606 GN=C6orf203	2.19	3.8469E-05

O14513	NCKAP5	Nck-associated protein 5 OS=Homo sapiens OX=9606 GN=NCKAP5	2.188	0.045924
A6NJG6	ARGFX	Arginine-fifty homeobox OS=Homo sapiens OX=9606 GN=ARGFX	2.184	0.000119
P11117	ACP2	Lysosomal acid phosphatase OS=Homo sapiens OX=9606 GN=ACP2	2.18	0.00053858
Q9UIJ7	AK3	"GTP:AMP phosphotransferase AK3, mitochondrial OS=Homo sapiens OX=9606 GN=AK3"	2.176	1.8041E-05
Q99487	PAFAH2	"Platelet-activating factor acetylhydrolase 2, cytoplasmic OS=Homo sapiens OX=9606 GN=PAFAH2"	2.176	4.2229E-05
Q9NZC3	GDE1	Glycerophosphodiester phosphodiesterase 1 OS=Homo sapiens OX=9606 GN=GDE1	2.175	3.0085E-06
Q9C0D2	CEP295	Centrosomal protein of 295 kDa OS=Homo sapiens OX=9606 GN=CEP295	2.174	0.00089502
O14529	CUX2	Homeobox protein cut-like 2 OS=Homo sapiens OX=9606 GN=CUX2	2.165	0.00018287
Q9HCH5	SYTL2	Synaptotagmin-like protein 2 OS=Homo sapiens OX=9606 GN=SYTL2	2.161	0.00028362
P15260	IFNGR1	Interferon gamma receptor 1 OS=Homo sapiens OX=9606 GN=IFNGR1	2.157	9.7441E-05
Q08AF3	SLFN5	Schlafen family member 5 OS=Homo sapiens OX=9606 GN=SLFN5	2.154	3.9402E-06
P49748	ACADVL	"Very long-chain specific acyl-CoA dehydrogenase, mitochondrial OS=Homo sapiens OX=9606 GN=ACADVL"	2.153	5.6075E-07
Q14249	ENDOG	"Endonuclease G, mitochondrial OS=Homo sapiens OX=9606 GN=ENDOG"	2.15	0.00082309
P07602	PSAP	Prosaposin OS=Homo sapiens OX=9606 GN=PSAP	2.148	1.7292E-05
O60437	PPL	Periplakin OS=Homo sapiens OX=9606 GN=PPL	2.146	3.2199E-06
Q8IXB1	DNAJC10	DnaJ homolog subfamily C member 10 OS=Homo sapiens OX=9606 GN=DNAJC10	2.145	4.3507E-06
P07686	HEXB	Beta-hexosaminidase subunit beta OS=Homo sapiens OX=9606 GN=HEXB	2.142	0.00043549
Q5XPI4	RNF123	E3 ubiquitin-protein ligase RNF123 OS=Homo sapiens OX=9606 GN=RNF123	2.142	0.0042027
O75110	ATP9A	Probable phospholipid-transporting ATPase IIA OS=Homo sapiens OX=9606 GN=ATP9A	2.136	3.6601E-05
Q53GD3	SLC44A4	Choline transporter-like protein 4 OS=Homo sapiens OX=9606 GN=SLC44A4	2.135	0.00036383
P30044	PRDX5	"Peroxiredoxin-5, mitochondrial OS=Homo sapiens OX=9606 GN=PRDX5"	2.134	4.0567E-06
O75460	ERN1	Serine/threonine-protein kinase/endoribonuclease IRE1 OS=Homo sapiens OX=9606 GN=ERN1	2.133	0.00020275
O94986	CEP152	Centrosomal protein of 152 kDa OS=Homo sapiens OX=9606 GN=CEP152	2.132	3.7138E-05
Q9H300	PARL	"Presenilins-associated rhomboid-like protein, mitochondrial OS=Homo sapiens OX=9606 GN=PARL"	2.131	3.605E-06
Q6NUK	SLC25A2	Calcium-binding mitochondrial carrier protein	2.12	1.2345E-

1	4	SCaMC-1 OS=Homo sapiens OX=9606 GN=SLC25A24	3	06
P61224	RAP1B	Ras-related protein Rap-1b OS=Homo sapiens OX=9606 GN=RAP1B	2.121	4.3728E-05
Q86WC4	OSTM1	Osteopetrosis-associated transmembrane protein 1 OS=Homo sapiens OX=9606 GN=OSTM1	2.12	0.00015725
O00116	AGPS	"Alkyldihydroxyacetonephosphate synthase, peroxisomal OS=Homo sapiens OX=9606 GN=AGPS"	2.118	0.00190026
Q969M3	YIPF5	Protein YIPF5 OS=Homo sapiens OX=9606 GN=YIPF5	2.117	0.0001225
Q9Y6Q1	CAPN6	Calpain-6 OS=Homo sapiens OX=9606 GN=CAPN6	2.116	0.00088452
Q9UG56	PISD	"Phosphatidylserine decarboxylase proenzyme, mitochondrial OS=Homo sapiens OX=9606 GN=PISD"	2.115	2.5203E-06
P00973	OAS1	2'-5'-oligoadenylate synthase 1 OS=Homo sapiens OX=9606 GN=OAS1	2.114	0.00026445
Q8TBM8	DNAJB14	DnaJ homolog subfamily B member 14 OS=Homo sapiens OX=9606 GN=DNAJB14	2.113	0.00046099
P14543	NID1	Nidogen-1 OS=Homo sapiens OX=9606 GN=NID1	2.111	7.9332E-05
Q9BRK4	LZTS2	Leucine zipper putative tumor suppressor 2 OS=Homo sapiens OX=9606 GN=LZTS2	2.11	0.0039833
Q4G0N4	NADK2	"NAD kinase 2, mitochondrial OS=Homo sapiens OX=9606 GN=NADK2"	2.109	0.0028244
Q9Y6L6	SLCO1B1	Solute carrier organic anion transporter family member 1B1 OS=Homo sapiens OX=9606 GN=SLCO1B1	2.108	0.00067734
P54707	ATP12A	Potassium-transporting ATPase alpha chain 2 OS=Homo sapiens OX=9606 GN=ATP12A	2.107	2.0303E-05
Q5T2T1	MPP7	MAGUK p55 subfamily member 7 OS=Homo sapiens OX=9606 GN=MPP7	2.106	1.601E-05
Q9HC78	ZBTB20	Zinc finger and BTB domain-containing protein 20 OS=Homo sapiens OX=9606 GN=ZBTB20	2.102	5.6516E-05
P83111	LACTB	"Serine beta-lactamase-like protein LACTB, mitochondrial OS=Homo sapiens OX=9606 GN=LACTB"	2.1	7.5919E-05
P53701	HCCS	Cytochrome c-type heme lyase OS=Homo sapiens OX=9606 GN=HCCS	2.095	2.4361E-06
P31431	SDC4	Syndecan-4 OS=Homo sapiens OX=9606 GN=SDC4	2.09	0.00000429
Q9Y4L1	HYOU1	Hypoxia up-regulated protein 1 OS=Homo sapiens OX=9606 GN=HYOU1	2.09	3.6006E-05
Q5S007	LRRK2	Leucine-rich repeat serine/threonine-protein kinase 2 OS=Homo sapiens OX=9606 GN=LRRK2	2.09	0.00160084
P19256	CD58	Lymphocyte function-associated antigen 3 OS=Homo sapiens OX=9606 GN=CD58	2.088	0.00023829
Q8N8R3	SLC25A29	Mitochondrial basic amino acids transporter OS=Homo sapiens OX=9606 GN=SLC25A29	2.087	3.3856E-06
P14406	COX7A2	"Cytochrome c oxidase subunit 7A2, mitochondrial OS=Homo sapiens OX=9606 GN=COX7A2"	2.083	0.00035602
P28799	GRN	Granulins OS=Homo sapiens OX=9606 GN=GRN	2.078	0.00068045

P67936	TPM4	Tropomyosin alpha-4 chain OS=Homo sapiens OX=9606 GN=TPM4	2.074	0.00058019
Q6IC98	GRAMD4	GRAM domain-containing protein 4 OS=Homo sapiens OX=9606 GN=GRAMD4	2.069	0.00131801
Q15067	ACOX1	Peroxisomal acyl-coenzyme A oxidase 1 OS=Homo sapiens OX=9606 GN=ACOX1	2.068	8.7226E-07
P14927	UQCRB	Cytochrome b-c1 complex subunit 7 OS=Homo sapiens OX=9606 GN=UQCRB	2.067	2.0204E-05
P49257	LMAN1	Protein ERGIC-53 OS=Homo sapiens OX=9606 GN=LMAN1	2.064	1.4772E-07
P26885	FKBP2	Peptidyl-prolyl cis-trans isomerase FKBP2 OS=Homo sapiens OX=9606 GN=FKBP2	2.063	0.00014181
O43795	MYO1B	Unconventional myosin-Ib OS=Homo sapiens OX=9606 GN=MYO1B	2.054	1.9081E-05
O00142	TK2	"Thymidine kinase 2, mitochondrial OS=Homo sapiens OX=9606 GN=TK2"	2.053	6.2538E-05
Q6ZSS7	MFSD6	Major facilitator superfamily domain-containing protein 6 OS=Homo sapiens OX=9606 GN=MFSD6	2.049	1.9096E-05
Q9UPY5	SLC7A11	Cystine/glutamate transporter OS=Homo sapiens OX=9606 GN=SLC7A11	2.049	3.9615E-05
P09110	ACAA1	"3-ketoacyl-CoA thiolase, peroxisomal OS=Homo sapiens OX=9606 GN=ACAA1"	2.048	0.0000232
Q9ULS5	TMCC3	Transmembrane and coiled-coil domain protein 3 OS=Homo sapiens OX=9606 GN=TMCC3	2.047	0.00057717
Q8TBP5	FAM174A	Membrane protein FAM174A OS=Homo sapiens OX=9606 GN=FAM174A	2.047	0.0093225
P33121	ACSL1	Long-chain-fatty-acid--CoA ligase 1 OS=Homo sapiens OX=9606 GN=ACSL1	2.046	0.00003514
P56277	CMC4	Cx9C motif-containing protein 4 OS=Homo sapiens OX=9606 GN=CMC4	2.046	0.00020006
P20020	ATP2B1	Plasma membrane calcium-transporting ATPase 1 OS=Homo sapiens OX=9606 GN=ATP2B1	2.044	9.1595E-07
O14975	SLC27A2	Very long-chain acyl-CoA synthetase OS=Homo sapiens OX=9606 GN=SLC27A2	2.044	2.4523E-06
P61916	NPC2	NPC intracellular cholesterol transporter 2 OS=Homo sapiens OX=9606 GN=NPC2	2.043	4.1683E-05
P22307	SCP2	Non-specific lipid-transfer protein OS=Homo sapiens OX=9606 GN=SCP2	2.042	1.5056E-05
P00488	F13A1	Coagulation factor XIII A chain OS=Homo sapiens OX=9606 GN=F13A1	2.039	0.0050205
Q9GZY0	NXF2	Nuclear RNA export factor 2 OS=Homo sapiens OX=9606 GN=NXF2	2.038	3.5169E-05
P35475	IDUA	Alpha-L-iduronidase OS=Homo sapiens OX=9606 GN=IDUA	2.038	0.00033721
Q9UHQ4	BCAP29	B-cell receptor-associated protein 29 OS=Homo sapiens OX=9606 GN=BCAP29	2.036	1.2018E-07
Q13825	AUH	"Methylglutaconyl-CoA hydratase, mitochondrial OS=Homo sapiens OX=9606 GN=AUH"	2.03	2.4004E-05
P51649	ALDH5A1	"Succinate-semialdehyde dehydrogenase, mitochondrial OS=Homo sapiens OX=9606 GN=ALDH5A1"	2.028	1.1446E-06
P28328	PEX2	Peroxisome biogenesis factor 2 OS=Homo sapiens	2.02	4.6799E-

		OX=9606 GN=PEX2	8	06
Q9Y3B3	TMED7	Transmembrane emp24 domain-containing protein 7 OS=Homo sapiens OX=9606 GN=TMED7	2.02 8	0.0001365 8
Q2NL98	VMAC	Vimentin-type intermediate filament-associated coiled-coil protein OS=Homo sapiens OX=9606 GN=VMAC	2.02 6	0.0076013
Q9Y305	ACOT9	"Acyl-coenzyme A thioesterase 9, mitochondrial OS=Homo sapiens OX=9606 GN=ACOT9"	2.02 1	2.1952E-05
Q15746	MYLK	"Myosin light chain kinase, smooth muscle OS=Homo sapiens OX=9606 GN=MYLK"	2.02	1.1853E-07
P16144	ITGB4	Integrin beta-4 OS=Homo sapiens OX=9606 GN=ITGB4	2.01 9	0.0009214 4
Q9BRT2	UQCC2	Ubiquinol-cytochrome-c reductase complex assembly factor 2 OS=Homo sapiens OX=9606 GN=UQCC2	2.01 8	4.8859E-06
Q86T03	PIP4P1	"Type 1 phosphatidylinositol 4,5-bisphosphate 4-phosphatase OS=Homo sapiens OX=9606 GN=PIP4P1"	2.01 8	0.0007012 8
Q99519	NEU1	Sialidase-1 OS=Homo sapiens OX=9606 GN=NEU1	2.01 8	0.0063585
P80303	NUCB2	Nucleobindin-2 OS=Homo sapiens OX=9606 GN=NUCB2	2.01 2	0.0000431 7
Q9BQE4	SELENOS	Selenoprotein S OS=Homo sapiens OX=9606 GN=SELENOS	2.01 1	0.0001192 3
Q13011	ECH1	"Delta(3,5)-Delta(2,4)-dienoyl-CoA isomerase, mitochondrial OS=Homo sapiens OX=9606 GN=ECH1"	2.00 7	3.0998E-07
Q92953	KCNB2	Potassium voltage-gated channel subfamily B member 2 OS=Homo sapiens OX=9606 GN=KCNB2	2.00 6	0.0008220 4
P38646	HSPA9	"Stress-70 protein, mitochondrial OS=Homo sapiens OX=9606 GN=HSPA9"	2.00 5	4.6528E-06
Q68CR1	SEL1L3	Protein sel-1 homolog 3 OS=Homo sapiens OX=9606 GN=SEL1L3	2.00 5	0.0006003 6
P32119	PRDX2	Peroxiredoxin-2 OS=Homo sapiens OX=9606 GN=PRDX2	0.49 9	1.691E-05
Q9C0C9	UBE2O	(E3-independent) E2 ubiquitin-conjugating enzyme OS=Homo sapiens OX=9606 GN=UBE2O	0.49 9	0.0003375 7
Q9UEU5	GAGE2D	G antigen 2D OS=Homo sapiens OX=9606 GN=GAGE2D	0.49 9	0.0023354
Q04637	EIF4G1	Eukaryotic translation initiation factor 4 gamma 1 OS=Homo sapiens OX=9606 GN=EIF4G1	0.49 8	1.4235E-06
P23258	TUBG1	Tubulin gamma-1 chain OS=Homo sapiens OX=9606 GN=TUBG1	0.49 8	1.5008E-05
P23588	EIF4B	Eukaryotic translation initiation factor 4B OS=Homo sapiens OX=9606 GN=EIF4B	0.49 8	4.0004E-05
P06744	GPI	Glucose-6-phosphate isomerase OS=Homo sapiens OX=9606 GN=GPI	0.49 8	5.5185E-05
O95372	LYPLA2	Acyl-protein thioesterase 2 OS=Homo sapiens OX=9606 GN=LYPLA2	0.49 8	0.0013787 6
Q15527	SURF2	Surfeit locus protein 2 OS=Homo sapiens OX=9606 GN=SURF2	0.49 8	0.003538
Q9UQ80	PA2G4	Proliferation-associated protein 2G4 OS=Homo sapiens OX=9606 GN=PA2G4	0.49 7	2.3893E-05
Q9UEW	STK39	STE20/SPS1-related proline-alanine-rich protein	0.49	4.7495E-

8		kinase OS=Homo sapiens OX=9606 GN=STK39	6	07
O75821	EIF3G	Eukaryotic translation initiation factor 3 subunit G OS=Homo sapiens OX=9606 GN=EIF3G	0.496	1.696E-05
Q6NXE6	ARMC6	Armadillo repeat-containing protein 6 OS=Homo sapiens OX=9606 GN=ARMC6	0.496	4.4399E-05
P47813	EIF1AX	"Eukaryotic translation initiation factor 1A, X-chromosomal OS=Homo sapiens OX=9606 GN=EIF1AX"	0.496	0.00019803
O14530	TXNDC9	Thioredoxin domain-containing protein 9 OS=Homo sapiens OX=9606 GN=TXNDC9	0.496	0.042761
Q9UBQ5	EIF3K	Eukaryotic translation initiation factor 3 subunit K OS=Homo sapiens OX=9606 GN=EIF3K	0.495	1.3275E-06
P46783	RPS10	40S ribosomal protein S10 OS=Homo sapiens OX=9606 GN=RPS10	0.495	2.0348E-05
P58546	MTPN	Myotrophin OS=Homo sapiens OX=9606 GN=MTPN	0.495	8.4004E-05
Q9BW19	KIFC1	Kinesin-like protein KIFC1 OS=Homo sapiens OX=9606 GN=KIFC1	0.495	0.00014407
Q9BTE6	AARSD1	Alanyl-tRNA editing protein Aarsd1 OS=Homo sapiens OX=9606 GN=AARSD1	0.495	0.00037608
Q96BR1	SGK3	Serine/threonine-protein kinase Sgk3 OS=Homo sapiens OX=9606 GN=SGK3	0.495	0.0020366
P83731	RPL24	60S ribosomal protein L24 OS=Homo sapiens OX=9606 GN=RPL24	0.495	0.0107625
Q8TBB5	KLHDC4	Kelch domain-containing protein 4 OS=Homo sapiens OX=9606 GN=KLHDC4	0.494	2.6317E-06
P08237	PFKM	"ATP-dependent 6-phosphofructokinase, muscle type OS=Homo sapiens OX=9606 GN=PFKM"	0.494	1.6971E-05
Q6NS38	ALKBH2	DNA oxidative demethylase ALKBH2 OS=Homo sapiens OX=9606 GN=ALKBH2	0.494	0.00157587
Q9Y5A9	YTHDF2	YTH domain-containing family protein 2 OS=Homo sapiens OX=9606 GN=YTHDF2	0.493	3.9159E-05
Q9UHV9	PFDN2	Prefoldin subunit 2 OS=Homo sapiens OX=9606 GN=PFDN2	0.492	0.00013797
P46109	CRKL	Crk-like protein OS=Homo sapiens OX=9606 GN=CRKL	0.492	0.00032491
Q99614	TTC1	Tetratricopeptide repeat protein 1 OS=Homo sapiens OX=9606 GN=TTC1	0.492	0.00053808
P12081	HARS	"Histidine--tRNA ligase, cytoplasmic OS=Homo sapiens OX=9606 GN=HARS"	0.491	1.0033E-07
O75449	KATNA1	Katanin p60 ATPase-containing subunit A1 OS=Homo sapiens OX=9606 GN=KATNA1	0.491	0.00002331
P20290	BTF3	Transcription factor BTF3 OS=Homo sapiens OX=9606 GN=BTF3	0.491	0.00015701
Q9ULR3	PPM1H	Protein phosphatase 1H OS=Homo sapiens OX=9606 GN=PPM1H	0.49	1.8095E-06
Q9H6D7	HAUS4	HAUS augmin-like complex subunit 4 OS=Homo sapiens OX=9606 GN=HAUS4	0.49	3.5579E-06
P08708	RPS17	40S ribosomal protein S17 OS=Homo sapiens OX=9606 GN=RPS17	0.49	2.1462E-05
P39019	RPS19	40S ribosomal protein S19 OS=Homo sapiens OX=9606 GN=RPS19	0.49	0.00005697

Q5VXD3	SAMD13	Sterile alpha motif domain-containing protein 13 OS=Homo sapiens OX=9606 GN=SAMD13	0.489	0.00081836
Q8NBA8	DTWD2	DTW domain-containing protein 2 OS=Homo sapiens OX=9606 GN=DTWD2	0.488	0.0041809
Q96P70	IPO9	Importin-9 OS=Homo sapiens OX=9606 GN=IPO9	0.487	3.6532E-06
O75223	GGCT	Gamma-glutamylcyclotransferase OS=Homo sapiens OX=9606 GN=GGCT	0.487	1.6154E-05
Q6IPR3	TYW3	tRNA wybutosine-synthesizing protein 3 homolog OS=Homo sapiens OX=9606 GN=TYW3	0.487	5.8156E-05
P35251	RFC1	Replication factor C subunit 1 OS=Homo sapiens OX=9606 GN=RFC1	0.486	0.00024065
Q8NG31	KNL1	Kinetochore scaffold 1 OS=Homo sapiens OX=9606 GN=KNL1	0.486	0.0024021
P68104	EEF1A1	Elongation factor 1-alpha 1 OS=Homo sapiens OX=9606 GN=EEF1A1	0.485	5.9024E-05
Q6PKG0	LARP1	La-related protein 1 OS=Homo sapiens OX=9606 GN=LARP1	0.484	4.5187E-07
P62861	FAU	40S ribosomal protein S30 OS=Homo sapiens OX=9606 GN=FAU	0.484	1.7376E-05
Q14493	SLBP	Histone RNA hairpin-binding protein OS=Homo sapiens OX=9606 GN=SLBP	0.484	0.00188242
P52306	RAP1GDS1	Rap1 GTPase-GDP dissociation stimulator 1 OS=Homo sapiens OX=9606 GN=RAP1GDS1	0.483	1.9239E-05
P83881	RPL36A	60S ribosomal protein L36a OS=Homo sapiens OX=9606 GN=RPL36A	0.483	0.00010228
O94830	DDHD2	Phospholipase DDHD2 OS=Homo sapiens OX=9606 GN=DDHD2	0.483	0.00029655
Q13070	GAGE6	G antigen 6 OS=Homo sapiens OX=9606 GN=GAGE6	0.483	0.0029445
Q9BXJ9	NAA15	"N-alpha-acetyltransferase 15, NatA auxiliary subunit OS=Homo sapiens OX=9606 GN=NAA15"	0.482	3.7307E-07
P68402	PAFAH1B2	Platelet-activating factor acetylhydrolase IB subunit beta OS=Homo sapiens OX=9606 GN=PAFAH1B2	0.482	3.4238E-06
Q05639	EEF1A2	Elongation factor 1-alpha 2 OS=Homo sapiens OX=9606 GN=EEF1A2	0.482	4.1577E-05
P25098	GRK2	Beta-adrenergic receptor kinase 1 OS=Homo sapiens OX=9606 GN=GRK2	0.482	4.2838E-05
Q6GMV2	SMYD5	SET and MYND domain-containing protein 5 OS=Homo sapiens OX=9606 GN=SMYD5	0.482	0.0157782
Q9Y5P4	COL4A3BP	Collagen type IV alpha-3-binding protein OS=Homo sapiens OX=9606 GN=COL4A3BP	0.481	0.00036072
Q14181	POLA2	DNA polymerase alpha subunit B OS=Homo sapiens OX=9606 GN=POLA2	0.48	1.7188E-06
P31948	STIP1	Stress-induced-phosphoprotein 1 OS=Homo sapiens OX=9606 GN=STIP1	0.479	1.0392E-07
P62937	PPIA	Peptidyl-prolyl cis-trans isomerase A OS=Homo sapiens OX=9606 GN=PPIA	0.479	1.7686E-06
Q99961	SH3GL1	Endophilin-A2 OS=Homo sapiens OX=9606 GN=SH3GL1	0.479	4.6771E-06
P57076	CFAP298	Cilia- and flagella-associated protein 298 OS=Homo sapiens OX=9606 GN=CFAP298	0.479	2.4573E-05

Q9H9T3	ELP3	Elongator complex protein 3 OS=Homo sapiens OX=9606 GN=ELP3	0.479	3.7553E-05
O75153	CLUH	Clustered mitochondria protein homolog OS=Homo sapiens OX=9606 GN=CLUH	0.479	4.0718E-05
Q8N806	UBR7	Putative E3 ubiquitin-protein ligase UBR7 OS=Homo sapiens OX=9606 GN=UBR7	0.479	9.6063E-05
P43358	MAGEA4	Melanoma-associated antigen 4 OS=Homo sapiens OX=9606 GN=MAGEA4	0.477	1.0946E-06
O14787	TNPO2	Transportin-2 OS=Homo sapiens OX=9606 GN=TNPO2	0.477	1.6755E-05
O00506	STK25	Serine/threonine-protein kinase 25 OS=Homo sapiens OX=9606 GN=STK25	0.477	0.00010296
Q49AA0	ZFP69	Zinc finger protein 69 homolog OS=Homo sapiens OX=9606 GN=ZFP69	0.477	0.00012493
Q99584	S100A13	Protein S100-A13 OS=Homo sapiens OX=9606 GN=S100A13	0.476	8.0507E-05
Q16763	UBE2S	Ubiquitin-conjugating enzyme E2 S OS=Homo sapiens OX=9606 GN=UBE2S	0.476	0.00042181
O43683	BUB1	Mitotic checkpoint serine/threonine-protein kinase BUB1 OS=Homo sapiens OX=9606 GN=BUB1	0.475	3.9841E-06
Q16543	CDC37	Hsp90 co-chaperone Cdc37 OS=Homo sapiens OX=9606 GN=CDC37	0.475	1.5921E-05
P62495	ETF1	Eukaryotic peptide chain release factor subunit 1 OS=Homo sapiens OX=9606 GN=ETF1	0.475	1.7529E-05
Q9Y6A5	TACC3	Transforming acidic coiled-coil-containing protein 3 OS=Homo sapiens OX=9606 GN=TACC3	0.475	0.00040024
O43847	NRDC	Nardilysin OS=Homo sapiens OX=9606 GN=NRDC	0.474	5.2956E-07
Q86WR0	CCDC25	Coiled-coil domain-containing protein 25 OS=Homo sapiens OX=9606 GN=CCDC25	0.474	2.1891E-05
P08238	HSP90AB1	Heat shock protein HSP 90-beta OS=Homo sapiens OX=9606 GN=HSP90AB1	0.472	2.2156E-07
P61758	VBP1	Prefoldin subunit 3 OS=Homo sapiens OX=9606 GN=VBP1	0.472	5.9572E-07
Q92664	GTF3A	Transcription factor IIIA OS=Homo sapiens OX=9606 GN=GTF3A	0.472	0.0067964
Q02790	FKBP4	Peptidyl-prolyl cis-trans isomerase FKBP4 OS=Homo sapiens OX=9606 GN=FKBP4	0.471	3.9455E-06
Q9UQR0	SCML2	Sex comb on midleg-like protein 2 OS=Homo sapiens OX=9606 GN=SCML2	0.471	5.5148E-05
P14618	PKM	Pyruvate kinase PKM OS=Homo sapiens OX=9606 GN=PKM	0.47	5.4971E-07
O96033	MOCS2	Molybdopterin synthase sulfur carrier subunit OS=Homo sapiens OX=9606 GN=MOCS2	0.47	8.1158E-05
Q96MW1	CCDC43	Coiled-coil domain-containing protein 43 OS=Homo sapiens OX=9606 GN=CCDC43	0.47	0.00010397
Q9BW71	HIRIP3	HIRA-interacting protein 3 OS=Homo sapiens OX=9606 GN=HIRIP3	0.469	3.398E-06
Q9P2S5	WRAP73	WD repeat-containing protein WRAP73 OS=Homo sapiens OX=9606 GN=WRAP73	0.469	0.00032276
P52565	ARHGDIA	Rho GDP-dissociation inhibitor 1 OS=Homo sapiens OX=9606 GN=ARHGDIA	0.469	0.00077696

Q9Y3F4	STRAP	Serine-threonine kinase receptor-associated protein OS=Homo sapiens OX=9606 GN=STRAP	0.468	9.0992E-07
Q9H0H5	RACGAP1	Rac GTPase-activating protein 1 OS=Homo sapiens OX=9606 GN=RACGAP1	0.467	0.00140119
P13798	APEH	Acylamino-acid-releasing enzyme OS=Homo sapiens OX=9606 GN=APEH	0.467	0.00175977
Q15398	DLGAP5	Disks large-associated protein 5 OS=Homo sapiens OX=9606 GN=DLGAP5	0.467	0.002139
Q15185	PTGES3	Prostaglandin E synthase 3 OS=Homo sapiens OX=9606 GN=PTGES3	0.464	1.6208E-05
P13797	PLS3	Plastin-3 OS=Homo sapiens OX=9606 GN=PLS3	0.464	3.5222E-05
P62857	RPS28	40S ribosomal protein S28 OS=Homo sapiens OX=9606 GN=RPS28	0.464	3.8327E-05
O00505	KPNA3	Importin subunit alpha-4 OS=Homo sapiens OX=9606 GN=KPNA3	0.464	0.00018072
P35789	ZNF93	Zinc finger protein 93 OS=Homo sapiens OX=9606 GN=ZNF93	0.463	0.00059547
Q9H467	CUEDC2	CUE domain-containing protein 2 OS=Homo sapiens OX=9606 GN=CUEDC2	0.463	0.0020188
Q14738	PPP2R5D	Serine/threonine-protein phosphatase 2A 56 kDa regulatory subunit delta isoform OS=Homo sapiens OX=9606 GN=PPP2R5D	0.461	1.8833E-05
P19623	SRM	Spermidine synthase OS=Homo sapiens OX=9606 GN=SRM	0.461	8.0608E-05
Q9H2P9	DPH5	Diphthine methyl ester synthase OS=Homo sapiens OX=9606 GN=DPH5	0.461	0.00028121
Q86Y56	DNAAF5	"Dynein assembly factor 5, axonemal OS=Homo sapiens OX=9606 GN=DNAAF5"	0.46	3.2183E-06
Q5UIP0	RIF1	Telomere-associated protein RIF1 OS=Homo sapiens OX=9606 GN=RIF1	0.46	6.4747E-05
Q8IV50	LYSMD2	LysM and putative peptidoglycan-binding domain-containing protein 2 OS=Homo sapiens OX=9606 GN=LYSMD2	0.46	9.7041E-05
Q96PM5	RCHY1	RING finger and CHY zinc finger domain-containing protein 1 OS=Homo sapiens OX=9606 GN=RCHY1	0.459	1.8926E-06
O95433	AHSA1	Activator of 90 kDa heat shock protein ATPase homolog 1 OS=Homo sapiens OX=9606 GN=AHSA1	0.459	4.201E-06
Q92569	PIK3R3	Phosphatidylinositol 3-kinase regulatory subunit gamma OS=Homo sapiens OX=9606 GN=PIK3R3	0.459	2.4202E-05
Q9Y597	KCTD3	BTB/POZ domain-containing protein KCTD3 OS=Homo sapiens OX=9606 GN=KCTD3	0.459	0.00017986
Q9NTK5	OLA1	Obg-like ATPase 1 OS=Homo sapiens OX=9606 GN=OLA1	0.458	3.0927E-06
Q96BN8	OTULIN	Ubiquitin thioesterase otulin OS=Homo sapiens OX=9606 GN=OTULIN	0.458	1.845E-05
Q13905	RAPGEF1	Rap guanine nucleotide exchange factor 1 OS=Homo sapiens OX=9606 GN=RAPGEF1	0.458	0.0025804
P12277	CKB	Creatine kinase B-type OS=Homo sapiens OX=9606 GN=CKB	0.457	1.6142E-05
P61221	ABCE1	ATP-binding cassette sub-family E member 1 OS=Homo sapiens OX=9606 GN=ABCE1	0.456	2.0316E-05

P11586	MTHFD1	"C-1-tetrahydrofolate synthase, cytoplasmic OS=Homo sapiens OX=9606 GN=MTHFD1"	0.455	5.9216E-07
P53611	RABGGTB	Geranylgeranyl transferase type-2 subunit beta OS=Homo sapiens OX=9606 GN=RABGGTB	0.455	0.00055737
Q14019	COTL1	Coactosin-like protein OS=Homo sapiens OX=9606 GN=COTL1	0.455	0.00140132
O75534	CSDE1	Cold shock domain-containing protein E1 OS=Homo sapiens OX=9606 GN=CSDE1	0.454	3.6685E-07
Q9BZ23	PANK2	"Pantothenate kinase 2, mitochondrial OS=Homo sapiens OX=9606 GN=PANK2"	0.453	4.1763E-05
Q9NVI1	FANCI	Fanconi anemia group I protein OS=Homo sapiens OX=9606 GN=FANCI	0.453	0.0034995
P60842	EIF4A1	Eukaryotic initiation factor 4A-I OS=Homo sapiens OX=9606 GN=EIF4A1	0.452	0.0048626
O75179	ANKRD17	Ankyrin repeat domain-containing protein 17 OS=Homo sapiens OX=9606 GN=ANKRD17	0.451	6.9449E-08
Q9H2B2	SYT4	Synaptotagmin-4 OS=Homo sapiens OX=9606 GN=SYT4	0.451	0.0041212
Q9Y6V0	PCLO	Protein piccolo OS=Homo sapiens OX=9606 GN=PCLO	0.451	0.041839
Q8WXX5	DNAJC9	DnaJ homolog subfamily C member 9 OS=Homo sapiens OX=9606 GN=DNAJC9	0.45	2.7831E-06
P26639	TARS	"Threonine--tRNA ligase, cytoplasmic OS=Homo sapiens OX=9606 GN=TARS"	0.449	1.6966E-07
Q99622	C12orf57	Protein C10 OS=Homo sapiens OX=9606 GN=C12orf57	0.449	3.7456E-05
Q969E8	TSR2	Pre-rRNA-processing protein TSR2 homolog OS=Homo sapiens OX=9606 GN=TSR2	0.449	4.2921E-05
Q6IBW4	NCAPH2	Condensin-2 complex subunit H2 OS=Homo sapiens OX=9606 GN=NCAPH2	0.449	6.4366E-05
Q9BZX2	UCK2	Uridine-cytidine kinase 2 OS=Homo sapiens OX=9606 GN=UCK2	0.449	9.7902E-05
Q9NP74	PALMD	Palmdelphin OS=Homo sapiens OX=9606 GN=PALMD	0.449	0.00043619
Q86WQ0	NR2C2AP	Nuclear receptor 2C2-associated protein OS=Homo sapiens OX=9606 GN=NR2C2AP	0.449	0.0043215
Q3KRA9	ALKBH6	Alpha-ketoglutarate-dependent dioxygenase alkB homolog 6 OS=Homo sapiens OX=9606 GN=ALKBH6	0.449	0.010581
O75131	CPNE3	Copine-3 OS=Homo sapiens OX=9606 GN=CPNE3	0.448	1.2984E-06
Q08752	PPID	Peptidyl-prolyl cis-trans isomerase D OS=Homo sapiens OX=9606 GN=PPID	0.448	0.00041521
P49840	GSK3A	Glycogen synthase kinase-3 alpha OS=Homo sapiens OX=9606 GN=GSK3A	0.447	5.5061E-05
Q9NVR5	DNAAF2	Protein kintoun OS=Homo sapiens OX=9606 GN=DNAAF2	0.447	0.00125558
O14965	AURKA	Aurora kinase A OS=Homo sapiens OX=9606 GN=AURKA	0.447	0.0024769
Q13111	CHAF1A	Chromatin assembly factor 1 subunit A OS=Homo sapiens OX=9606 GN=CHAF1A	0.446	3.9152E-05
Q9Y547	HSPB11	Intraflagellar transport protein 25 homolog OS=Homo	0.44	0.037481

		sapiens OX=9606 GN=HSPB11	6	
O14737	PDCD5	Programmed cell death protein 5 OS=Homo sapiens OX=9606 GN=PDCD5	0.44 5	5.7647E-05
O14494	PLPP1	Phospholipid phosphatase 1 OS=Homo sapiens OX=9606 GN=PLPP1	0.44 5	0.0001358 2
Q9UNY 4	TTF2	Transcription termination factor 2 OS=Homo sapiens OX=9606 GN=TTF2	0.44 5	0.0003585 6
Q9H3R5	CENPH	Centromere protein H OS=Homo sapiens OX=9606 GN=CENPH	0.44 5	0.0016406 3
O95229	ZWINT	ZW10 interactor OS=Homo sapiens OX=9606 GN=ZWINT	0.44 5	0.0176015
P07195	LDHB	L-lactate dehydrogenase B chain OS=Homo sapiens OX=9606 GN=LDHB	0.44 4	1.6413E-06
Q9H2J4	PDCL3	Phosducin-like protein 3 OS=Homo sapiens OX=9606 GN=PDCL3	0.44 4	3.477E-06
Q92990	GLMN	Glomulin OS=Homo sapiens OX=9606 GN=GLMN	0.44 4	1.6426E-05
Q7Z7E8	UBE2Q1	Ubiquitin-conjugating enzyme E2 Q1 OS=Homo sapiens OX=9606 GN=UBE2Q1	0.44 4	9.6816E-05
P50395	GDI2	Rab GDP dissociation inhibitor beta OS=Homo sapiens OX=9606 GN=GDI2	0.44 1	9.6542E-08
P17812	CTPS1	CTP synthase 1 OS=Homo sapiens OX=9606 GN=CTPS1	0.44 1	2.2132E-07
Q96GA3	LTV1	Protein LTV1 homolog OS=Homo sapiens OX=9606 GN=LTV1	0.44 1	1.9563E-05
O75310	UGT2B11	UDP-glucuronosyltransferase 2B11 OS=Homo sapiens OX=9606 GN=UGT2B11	0.44 1	0.0021761
P00338	LDHA	L-lactate dehydrogenase A chain OS=Homo sapiens OX=9606 GN=LDHA	0.44	4.7579E-06
Q9UIK5	TMEFF2	Tomoregulin-2 OS=Homo sapiens OX=9606 GN=TMEFF2	0.44	0.0003952
P24941	CDK2	Cyclin-dependent kinase 2 OS=Homo sapiens OX=9606 GN=CDK2	0.43 9	5.615E-08
Q9Y4P1	ATG4B	Cysteine protease ATG4B OS=Homo sapiens OX=9606 GN=ATG4B	0.43 9	2.5873E-06
Q99986	VRK1	Serine/threonine-protein kinase VRK1 OS=Homo sapiens OX=9606 GN=VRK1	0.43 9	8.4458E-05
O00154	ACOT7	Cytosolic acyl coenzyme A thioester hydrolase OS=Homo sapiens OX=9606 GN=ACOT7	0.43 8	3.9594E-07
P08243	ASNS	Asparagine synthetase [glutamine-hydrolyzing] OS=Homo sapiens OX=9606 GN=ASNS	0.43 7	0.0047213
P13693	TPT1	Translationally-controlled tumor protein OS=Homo sapiens OX=9606 GN=TPT1	0.43 6	3.7207E-06
Q4V326	GAGE2E	G antigen 2E OS=Homo sapiens OX=9606 GN=GAGE2E	0.43 6	1.5682E-05
Q9H4H8	FAM83D	Protein FAM83D OS=Homo sapiens OX=9606 GN=FAM83D	0.43 6	0.0003571 7
P34896	SHMT1	"Serine hydroxymethyltransferase, cytosolic OS=Homo sapiens OX=9606 GN=SHMT1"	0.43 6	0.0089759
P37802	TAGLN2	Transgelin-2 OS=Homo sapiens OX=9606 GN=TAGLN2	0.43 5	0.0000223 8
O00244	ATOX1	Copper transport protein ATOX1 OS=Homo sapiens	0.43	0.0001405

		OX=9606 GN=ATOX1	5	5
Q8TAP6	CEP76	Centrosomal protein of 76 kDa OS=Homo sapiens OX=9606 GN=CEP76	0.435	0.00035509
O75347	TBCA	Tubulin-specific chaperone A OS=Homo sapiens OX=9606 GN=TBCA	0.434	3.0884E-06
Q9GZU2	PEG3	Paternally-expressed gene 3 protein OS=Homo sapiens OX=9606 GN=PEG3	0.434	0.00019934
Q8WVJ2	NUDCD2	NudC domain-containing protein 2 OS=Homo sapiens OX=9606 GN=NUDCD2	0.434	0.00063964
Q7Z7J9	CAMK2N1	Calcium/calmodulin-dependent protein kinase II inhibitor 1 OS=Homo sapiens OX=9606 GN=CAMK2N1	0.433	1.6585E-05
Q9BV29	CCDC32	Coiled-coil domain-containing protein 32 OS=Homo sapiens OX=9606 GN=CCDC32	0.433	0.00016229
Q96SB4	SRPK1	SRSF protein kinase 1 OS=Homo sapiens OX=9606 GN=SRPK1	0.433	0.00130189
Q8TCY9	URGCP	Up-regulator of cell proliferation OS=Homo sapiens OX=9606 GN=URGCP	0.433	0.00138367
Q05D60	DEUP1	Deuterosome assembly protein 1 OS=Homo sapiens OX=9606 GN=DEUP1	0.433	0.0070238
Q13572	ITPK1	Inositol-tetrakisphosphate 1-kinase OS=Homo sapiens OX=9606 GN=ITPK1	0.432	0.00032173
Q6FI81	CIAPIN1	Anamorsin OS=Homo sapiens OX=9606 GN=CIAPIN1	0.431	2.8123E-06
P41227	NAA10	N-alpha-acetyltransferase 10 OS=Homo sapiens OX=9606 GN=NAA10	0.431	8.0481E-05
Q9Y266	NUDC	Nuclear migration protein nudC OS=Homo sapiens OX=9606 GN=NUDC	0.429	9.6717E-07
Q9NVW2	RLIM	E3 ubiquitin-protein ligase RLIM OS=Homo sapiens OX=9606 GN=RLIM	0.429	0.0158974
Q13085	ACACA	Acetyl-CoA carboxylase 1 OS=Homo sapiens OX=9606 GN=ACACA	0.428	9.7976E-07
Q13451	FKBP5	Peptidyl-prolyl cis-trans isomerase FKBP5 OS=Homo sapiens OX=9606 GN=FKBP5	0.427	7.2297E-07
Q96DR5	BPIFA2	BPI fold-containing family A member 2 OS=Homo sapiens OX=9606 GN=BPIFA2	0.427	1.5534E-05
P49454	CENPF	Centromere protein F OS=Homo sapiens OX=9606 GN=CENPF	0.427	0.0038819
O15318	POLR3G	DNA-directed RNA polymerase III subunit RPC7 OS=Homo sapiens OX=9606 GN=POLR3G	0.426	2.8207E-06
P54792	DVL1P1	Putative segment polarity protein dishevelled homolog DVL1P1 OS=Homo sapiens OX=9606 GN=DVL1P1	0.426	1.9767E-05
O14732	IMPA2	Inositol monophosphatase 2 OS=Homo sapiens OX=9606 GN=IMPA2	0.426	0.0025161
Q9NVP2	ASF1B	Histone chaperone ASF1B OS=Homo sapiens OX=9606 GN=ASF1B	0.425	0.002341
Q9ULW0	TPX2	Targeting protein for Xklp2 OS=Homo sapiens OX=9606 GN=TPX2	0.425	0.0105813
Q02241	KIF23	Kinesin-like protein KIF23 OS=Homo sapiens OX=9606 GN=KIF23	0.424	0.00012095
Q99615	DNAJC7	DnaJ homolog subfamily C member 7 OS=Homo sapiens OX=9606 GN=DNAJC7	0.423	1.5481E-05

P78330	PSPH	Phosphoserine phosphatase OS=Homo sapiens OX=9606 GN=PSPH	0.423	0.00014134
P39748	FEN1	Flap endonuclease 1 OS=Homo sapiens OX=9606 GN=FEN1	0.422	2.3923E-06
Q9HAN9	NMNAT1	Nicotinamide/nicotinic acid mononucleotide adenylyltransferase 1 OS=Homo sapiens OX=9606 GN=NMNAT1	0.422	8.0717E-05
O14757	CHEK1	Serine/threonine-protein kinase Chk1 OS=Homo sapiens OX=9606 GN=CHEK1	0.422	0.000103
Q9Y2Y1	POLR3K	DNA-directed RNA polymerase III subunit RPC10 OS=Homo sapiens OX=9606 GN=POLR3K	0.422	0.00022447
P49459	UBE2A	Ubiquitin-conjugating enzyme E2 A OS=Homo sapiens OX=9606 GN=UBE2A	0.422	0.0142006
Q9UK76	JPT1	Jupiter microtubule associated homolog 1 OS=Homo sapiens OX=9606 GN=JPT1	0.421	1.8176E-05
Q9NX55	HYPK	Huntingtin-interacting protein K OS=Homo sapiens OX=9606 GN=HYPK	0.421	4.0871E-05
P62987	UBA52	Ubiquitin-60S ribosomal protein L40 OS=Homo sapiens OX=9606 GN=UBA52	0.421	0.0133644
Q8NC51	SERBP1	Plasminogen activator inhibitor 1 RNA-binding protein OS=Homo sapiens OX=9606 GN=SERBP1	0.419	8.9971E-08
Q92995	USP13	Ubiquitin carboxyl-terminal hydrolase 13 OS=Homo sapiens OX=9606 GN=USP13	0.419	1.579E-05
P01130	LDLR	Low-density lipoprotein receptor OS=Homo sapiens OX=9606 GN=LDLR	0.419	4.1267E-05
Q96GD4	AURKB	Aurora kinase B OS=Homo sapiens OX=9606 GN=AURKB	0.419	0.00016361
Q9Y316	MEMO1	Protein MEMO1 OS=Homo sapiens OX=9606 GN=MEMO1	0.418	6.1836E-05
Q9UNS1	TIMELESS	Protein timeless homolog OS=Homo sapiens OX=9606 GN=TIMELESS	0.418	0.00129676
Q9NYP7	ELOVL5	Elongation of very long chain fatty acids protein 5 OS=Homo sapiens OX=9606 GN=ELOVL5	0.417	0.00014139
Q9UI26	IPO11	Importin-11 OS=Homo sapiens OX=9606 GN=IPO11	0.414	4.6162E-06
P63241	EIF5A	Eukaryotic translation initiation factor 5A-1 OS=Homo sapiens OX=9606 GN=EIF5A	0.413	3.8088E-05
P27348	YWHAQ	14-3-3 protein theta OS=Homo sapiens OX=9606 GN=YWHAQ	0.412	2.1161E-06
Q8TCA0	LRRC20	Leucine-rich repeat-containing protein 20 OS=Homo sapiens OX=9606 GN=LRRC20	0.412	0.00147885
P62253	UBE2G1	Ubiquitin-conjugating enzyme E2 G1 OS=Homo sapiens OX=9606 GN=UBE2G1	0.411	5.9214E-05
P52564	MAP2K6	Dual specificity mitogen-activated protein kinase kinase 6 OS=Homo sapiens OX=9606 GN=MAP2K6	0.411	0.00042037
P07737	PFN1	Profilin-1 OS=Homo sapiens OX=9606 GN=PFN1	0.41	2.4893E-05
Q8NEM2	SHCBP1	SHC SH2 domain-binding protein 1 OS=Homo sapiens OX=9606 GN=SHCBP1	0.41	0.0081559
Q9BPX3	NCAPG	Condensin complex subunit 3 OS=Homo sapiens OX=9606 GN=NCAPG	0.409	0.00013902
P26358	DNMT1	DNA (cytosine-5)-methyltransferase 1 OS=Homo	0.40	0.0029835

		sapiens OX=9606 GN=DNMT1	9	
Q96DG6	CMBL	Carboxymethylenebutenolidase homolog OS=Homo sapiens OX=9606 GN=CMBL	0.40 8	2.4192E-06
Q2NKX 8	ERCC6L	DNA excision repair protein ERCC-6-like OS=Homo sapiens OX=9606 GN=ERCC6L	0.40 8	0.0015189 3
P14324	FDPS	Farnesyl pyrophosphate synthase OS=Homo sapiens OX=9606 GN=FDPS	0.40 4	5.6061E-05
Q9UBB4	ATXN10	Ataxin-10 OS=Homo sapiens OX=9606 GN=ATXN10	0.40 2	4.0674E-05
Q96GX2	ATXN7L3 B	Ataxin-7-like protein 3B OS=Homo sapiens OX=9606 GN=ATXN7L3B	0.40 2	0.0003243 1
Q9GZZ1	NAA50	N-alpha-acetyltransferase 50 OS=Homo sapiens OX=9606 GN=NAA50	0.40 1	5.0059E-07
Q14353	GAMT	Guanidinoacetate N-methyltransferase OS=Homo sapiens OX=9606 GN=GAMT	0.40 1	4.3559E-05
P20839	IMPDH1	Inosine-5'-monophosphate dehydrogenase 1 OS=Homo sapiens OX=9606 GN=IMPDH1	0.39 9	5.5842E-05
O00767	SCD	Acyl-CoA desaturase OS=Homo sapiens OX=9606 GN=SCD	0.39 9	0.0010956 9
Q8WVB 6	CHTF18	Chromosome transmission fidelity protein 18 homolog OS=Homo sapiens OX=9606 GN=CHTF18	0.39 7	0.0001023 3
P19174	PLCG1	"1-phosphatidylinositol 4,5-bisphosphate phosphodiesterase gamma-1 OS=Homo sapiens OX=9606 GN=PLCG1"	0.39 7	0.0001366 8
Q9BXW 9	FANCD2	Fanconi anemia group D2 protein OS=Homo sapiens OX=9606 GN=FANCD2	0.39 7	0.0004607 5
Q06203	PPAT	Amidophosphoribosyltransferase OS=Homo sapiens OX=9606 GN=PPAT	0.39 6	1.5311E-05
P49321	NASP	Nuclear autoantigenic sperm protein OS=Homo sapiens OX=9606 GN=NASP	0.39 6	3.6063E-05
Q9NVQ 4	FAIM	Fas apoptotic inhibitory molecule 1 OS=Homo sapiens OX=9606 GN=FAIM	0.39 6	0.0002838 6
P00492	HPRT1	Hypoxanthine-guanine phosphoribosyltransferase OS=Homo sapiens OX=9606 GN=HPRT1	0.39 5	3.1002E-06
P49736	MCM2	DNA replication licensing factor MCM2 OS=Homo sapiens OX=9606 GN=MCM2	0.39 4	1.0954E-06
O43805	SSNA1	Sjoegren syndrome nuclear autoantigen 1 OS=Homo sapiens OX=9606 GN=SSNA1	0.39 4	1.5196E-05
Q9NQW 6	ANLN	Anillin OS=Homo sapiens OX=9606 GN=ANLN	0.39 4	0.0010989 1
Q6PIW4	FIGNL1	Fidgetin-like protein 1 OS=Homo sapiens OX=9606 GN=FIGNL1	0.39 2	0.0000573 1
P23528	CFL1	Cofilin-1 OS=Homo sapiens OX=9606 GN=CFL1	0.39 1	2.1521E-06
P55060	CSE1L	Exportin-2 OS=Homo sapiens OX=9606 GN=CSE1L	0.39 1	3.6864E-05
Q9BV57	ADI1	"1,2-dihydroxy-3-keto-5-methylthiopentene dioxygenase OS=Homo sapiens OX=9606 GN=ADI1"	0.39 1	5.5628E-05
P18858	LIG1	DNA ligase 1 OS=Homo sapiens OX=9606 GN=LIG1	0.39 1	0.0005004 1
Q9BWG 4	SSBP4	Single-stranded DNA-binding protein 4 OS=Homo sapiens OX=9606 GN=SSBP4	0.38 9	2.8917E-06

Q6PL18	ATAD2	ATPase family AAA domain-containing protein 2 OS=Homo sapiens OX=9606 GN=ATAD2	0.388	3.6087E-06
Q15785	TOMM34	Mitochondrial import receptor subunit TOM34 OS=Homo sapiens OX=9606 GN=TOMM34	0.387	1.7778E-05
Q8WU79	SMAP2	Stromal membrane-associated protein 2 OS=Homo sapiens OX=9606 GN=SMAP2	0.387	2.3313E-05
Q01581	HMGCS1	"Hydroxymethylglutaryl-CoA synthase, cytoplasmic OS=Homo sapiens OX=9606 GN=HMGCS1"	0.387	0.0001016
Q9Y248	GINS2	DNA replication complex GINS protein PSF2 OS=Homo sapiens OX=9606 GN=GINS2	0.387	0.00133924
A6NDG6	PGP	Glycerol-3-phosphate phosphatase OS=Homo sapiens OX=9606 GN=PGP	0.386	1.2684E-06
P27707	DCK	Deoxycytidine kinase OS=Homo sapiens OX=9606 GN=DCK	0.381	0.00013724
Q9BQL6	FERMT1	Fermitin family homolog 1 OS=Homo sapiens OX=9606 GN=FERMT1	0.381	0.0025428
P53396	ACLY	ATP-citrate synthase OS=Homo sapiens OX=9606 GN=ACLY	0.379	4.3145E-06
Q9NS91	RAD18	E3 ubiquitin-protein ligase RAD18 OS=Homo sapiens OX=9606 GN=RAD18	0.379	0.00108177
Q9UHD1	CHORDC1	Cysteine and histidine-rich domain-containing protein 1 OS=Homo sapiens OX=9606 GN=CHORDC1	0.379	0.0051799
P22234	PAICS	Multifunctional protein ADE2 OS=Homo sapiens OX=9606 GN=PAICS	0.375	2.2891E-06
Q15054	POLD3	DNA polymerase delta subunit 3 OS=Homo sapiens OX=9606 GN=POLD3	0.374	2.2256E-06
Q15645	TRIP13	Pachytene checkpoint protein 2 homolog OS=Homo sapiens OX=9606 GN=TRIP13	0.374	3.4133E-06
Q13951	CBFB	Core-binding factor subunit beta OS=Homo sapiens OX=9606 GN=CBFB	0.373	4.1623E-06
Q9NRN7	AASDHPPT	L-aminoadipate-semialdehyde dehydrogenase-phosphopantetheinyl transferase OS=Homo sapiens OX=9606 GN=AASDHPPT	0.37	0.00089695
Q9NQS7	INCENP	Inner centromere protein OS=Homo sapiens OX=9606 GN=INCENP	0.37	0.0086154
Q99801	NKX3-1	Homeobox protein Nkx-3.1 OS=Homo sapiens OX=9606 GN=NKX3-1	0.368	2.0699E-05
Q13542	EIF4EBP2	Eukaryotic translation initiation factor 4E-binding protein 2 OS=Homo sapiens OX=9606 GN=EIF4EBP2	0.367	2.0984E-05
Q7L8W6	DPH6	Diphthine--ammonia ligase OS=Homo sapiens OX=9606 GN=DPH6	0.367	0.0029773
O95235	KIF20A	Kinesin-like protein KIF20A OS=Homo sapiens OX=9606 GN=KIF20A	0.364	8.4222E-05
O43692	PI15	Peptidase inhibitor 15 OS=Homo sapiens OX=9606 GN=PI15	0.363	7.7495E-05
Q8N5I9	C12orf45	Uncharacterized protein C12orf45 OS=Homo sapiens OX=9606 GN=C12orf45	0.362	1.6858E-05
Q9HB71	CACYBP	Calcyclin-binding protein OS=Homo sapiens OX=9606 GN=CACYBP	0.361	2.0782E-08
Q9UBK9	UXT	Protein UXT OS=Homo sapiens OX=9606 GN=UXT	0.361	0.033816
P13639	EEF2	Elongation factor 2 OS=Homo sapiens OX=9606	0.36	1.1325E-

		GN=EEF2		06
Q8WUX2	CHAC2	Glutathione-specific gamma-glutamylcyclotransferase 2 OS=Homo sapiens OX=9606 GN=CHAC2	0.36	0.00095512
Q7Z6M1	RABEPK	Rab9 effector protein with kelch motifs OS=Homo sapiens OX=9606 GN=RABEPK	0.358	0.00036268
Q9HBM1	SPC25	Kinetochore protein Spc25 OS=Homo sapiens OX=9606 GN=SPC25	0.358	0.00057646
Q9BTE3	MCMBP	Mini-chromosome maintenance complex-binding protein OS=Homo sapiens OX=9606 GN=MCMBP	0.357	0.0001583
Q8N7N1	FAM86B1	Putative protein N-methyltransferase FAM86B1 OS=Homo sapiens OX=9606 GN=FAM86B1	0.357	0.00123642
Q9H7B4	SMYD3	Histone-lysine N-methyltransferase SMYD3 OS=Homo sapiens OX=9606 GN=SMYD3	0.355	0.00026051
Q86UD0	SAPCD2	Suppressor APC domain-containing protein 2 OS=Homo sapiens OX=9606 GN=SAPCD2	0.355	0.002036
O43663	PRC1	Protein regulator of cytokinesis 1 OS=Homo sapiens OX=9606 GN=PRC1	0.353	0.0061047
O75717	WDHD1	WD repeat and HMG-box DNA-binding protein 1 OS=Homo sapiens OX=9606 GN=WDHD1	0.352	0.00026456
Q9Y4F9	RIPOR2	Rho family-interacting cell polarization regulator 2 OS=Homo sapiens OX=9606 GN=RIPOR2	0.35	0.0031596
Q9BPZ3	PAIP2	Polyadenylate-binding protein-interacting protein 2 OS=Homo sapiens OX=9606 GN=PAIP2	0.348	1.8977E-05
P06454	PTMA	Prothymosin alpha OS=Homo sapiens OX=9606 GN=PTMA	0.347	8.2906E-05
Q9NR48	ASH1L	Histone-lysine N-methyltransferase ASH1L OS=Homo sapiens OX=9606 GN=ASH1L	0.347	0.00058268
P33981	TTK	Dual specificity protein kinase TTK OS=Homo sapiens OX=9606 GN=TTK	0.346	0.0026449
Q9Y6E2	BZW2	Basic leucine zipper and W2 domain-containing protein 2 OS=Homo sapiens OX=9606 GN=BZW2	0.343	4.6665E-06
Q9H9S4	CAB39L	Calcium-binding protein 39-like OS=Homo sapiens OX=9606 GN=CAB39L	0.341	8.628E-07
P07108	DBI	Acyl-CoA-binding protein OS=Homo sapiens OX=9606 GN=DBI	0.341	3.6851E-06
P60891	PRPS1	Ribose-phosphate pyrophosphokinase 1 OS=Homo sapiens OX=9606 GN=PRPS1	0.339	2.1938E-05
P33991	MCM4	DNA replication licensing factor MCM4 OS=Homo sapiens OX=9606 GN=MCM4	0.337	0.00002207
O95239	KIF4A	Chromosome-associated kinesin KIF4A OS=Homo sapiens OX=9606 GN=KIF4A	0.337	0.0052607
P43487	RANBP1	Ran-specific GTPase-activating protein OS=Homo sapiens OX=9606 GN=RANBP1	0.335	6.1309E-05
Q53EZ4	CEP55	Centrosomal protein of 55 kDa OS=Homo sapiens OX=9606 GN=CEP55	0.334	0.0074846
Q99741	CDC6	Cell division control protein 6 homolog OS=Homo sapiens OX=9606 GN=CDC6	0.33	0.00109783
Q9H446	RWDD1	RWD domain-containing protein 1 OS=Homo sapiens OX=9606 GN=RWDD1	0.327	0.00016369
P79522	PRR3	Proline-rich protein 3 OS=Homo sapiens OX=9606 GN=PRR3	0.326	1.5577E-05
P06493	CDK1	Cyclin-dependent kinase 1 OS=Homo sapiens	0.32	0.0001847

		OX=9606 GN=CDK1	6	4
P55209	NAP1L1	Nucleosome assembly protein 1-like 1 OS=Homo sapiens OX=9606 GN=NAP1L1	0.32 5	2.0578E-05
P25205	MCM3	DNA replication licensing factor MCM3 OS=Homo sapiens OX=9606 GN=MCM3	0.32 5	3.9555E-05
Q8TCG1	CIP2A	Protein CIP2A OS=Homo sapiens OX=9606 GN=CIP2A	0.32 5	0.0037016
P33992	MCM5	DNA replication licensing factor MCM5 OS=Homo sapiens OX=9606 GN=MCM5	0.32 2	5.9981E-05
Q9UHW 5	GPN3	GPN-loop GTPase 3 OS=Homo sapiens OX=9606 GN=GPN3	0.32 1	9.5693E-05
Q71F23	CENPU	Centromere protein U OS=Homo sapiens OX=9606 GN=CENPU	0.32 1	0.0004825 7
P61024	CKS1B	Cyclin-dependent kinases regulatory subunit 1 OS=Homo sapiens OX=9606 GN=CKS1B	0.32	0.0017794 1
Q9NSA3	CTNNBIP 1	Beta-catenin-interacting protein 1 OS=Homo sapiens OX=9606 GN=CTNNBIP1	0.31 9	0.0001620 3
Q13112	CHAF1B	Chromatin assembly factor 1 subunit B OS=Homo sapiens OX=9606 GN=CHAF1B	0.31 9	0.0018844 3
Q15021	NCAPD2	Condensin complex subunit 1 OS=Homo sapiens OX=9606 GN=NCAPD2	0.31 9	0.008838
Q53FT3	HIKESHI	Protein Hikeshi OS=Homo sapiens OX=9606 GN=HIKESHI	0.31 6	3.8569E-07
Q9Y448	KNSTRN	Small kinetochore-associated protein OS=Homo sapiens OX=9606 GN=KNSTRN	0.31 6	0.0001034 8
Q6SJ93	FAM111B	Protein FAM111B OS=Homo sapiens OX=9606 GN=FAM111B	0.31 6	0.0003236 8
Q96T88	UHRF1	E3 ubiquitin-protein ligase UHRF1 OS=Homo sapiens OX=9606 GN=UHRF1	0.31 3	0.0001042 5
Q96C90	PPP1R14 B	Protein phosphatase 1 regulatory subunit 14B OS=Homo sapiens OX=9606 GN=PPP1R14B	0.31 3	0.0003154 9
Q99640	PKMYT1	Membrane-associated tyrosine- and threonine-specific cdc2-inhibitory kinase OS=Homo sapiens OX=9606 GN=PKMYT1	0.31 3	0.001045
P50748	KNTC1	Kinetochore-associated protein 1 OS=Homo sapiens OX=9606 GN=KNTC1	0.31 2	0.0003626 5
P52732	KIF11	Kinesin-like protein KIF11 OS=Homo sapiens OX=9606 GN=KIF11	0.30 9	0.0001572 1
Q8IX90	SKA3	Spindle and kinetochore-associated protein 3 OS=Homo sapiens OX=9606 GN=SKA3	0.30 9	0.0056637
Q99618	CDCA3	Cell division cycle-associated protein 3 OS=Homo sapiens OX=9606 GN=CDCA3	0.30 7	1.8362E-05
Q9BRT9	GINS4	DNA replication complex GINS protein SLD5 OS=Homo sapiens OX=9606 GN=GINS4	0.30 7	0.0008368 2
Q9H999	PANK3	Pantothenate kinase 3 OS=Homo sapiens OX=9606 GN=PANK3	0.30 7	0.0011163 7
O95347	SMC2	Structural maintenance of chromosomes protein 2 OS=Homo sapiens OX=9606 GN=SMC2	0.30 7	0.004181
Q9NZJ0	DTL	Denticleless protein homolog OS=Homo sapiens OX=9606 GN=DTL	0.30 6	0.0001410 7
P07288	KLK3	Prostate-specific antigen OS=Homo sapiens OX=9606 GN=KLK3	0.30 4	0.0000354 3

O14777	NDC80	Kinetochore protein NDC80 homolog OS=Homo sapiens OX=9606 GN=NDC80	0.302	6.1943E-05
P49327	FASN	Fatty acid synthase OS=Homo sapiens OX=9606 GN=FASN	0.3	0.0028195
Q14566	MCM6	DNA replication licensing factor MCM6 OS=Homo sapiens OX=9606 GN=MCM6	0.296	0.0024822
O75496	GMNN	Geminin OS=Homo sapiens OX=9606 GN=GMNN	0.287	0.01596
Q9NTJ3	SMC4	Structural maintenance of chromosomes protein 4 OS=Homo sapiens OX=9606 GN=SMC4	0.285	3.7911E-05
P14635	CCNB1	G2/mitotic-specific cyclin-B1 OS=Homo sapiens OX=9606 GN=CCNB1	0.284	3.9594E-05
Q9Y2Y0	ARL2BP	ADP-ribosylation factor-like protein 2-binding protein OS=Homo sapiens OX=9606 GN=ARL2BP	0.282	9.6884E-05
P12004	PCNA	Proliferating cell nuclear antigen OS=Homo sapiens OX=9606 GN=PCNA	0.281	8.8806E-07
P52292	KPNA2	Importin subunit alpha-1 OS=Homo sapiens OX=9606 GN=KPNA2	0.281	9.8429E-05
Q13257	MAD2L1	Mitotic spindle assembly checkpoint protein MAD2A OS=Homo sapiens OX=9606 GN=MAD2L1	0.28	0.00005607
O75330	HMMR	Hyaluronan mediated motility receptor OS=Homo sapiens OX=9606 GN=HMMR	0.28	0.0051636
P11388	TOP2A	DNA topoisomerase 2-alpha OS=Homo sapiens OX=9606 GN=TOP2A	0.276	6.2268E-05
O75419	CDC45	Cell division control protein 45 homolog OS=Homo sapiens OX=9606 GN=CDC45	0.276	0.00049615
P33993	MCM7	DNA replication licensing factor MCM7 OS=Homo sapiens OX=9606 GN=MCM7	0.275	0.00010336
Q9BW04	SARG	Specifically androgen-regulated gene protein OS=Homo sapiens OX=9606 GN=SARG	0.273	2.2038E-06
Q96R06	SPAG5	Sperm-associated antigen 5 OS=Homo sapiens OX=9606 GN=SPAG5	0.273	0.0097758
Q9BVW5	TIPIN	TIMELESS-interacting protein OS=Homo sapiens OX=9606 GN=TIPIN	0.272	2.0761E-05
P46013	MKI67	Proliferation marker protein Ki-67 OS=Homo sapiens OX=9606 GN=MKI67	0.272	0.0053999
Q8N2Z9	CENPS	Centromere protein S OS=Homo sapiens OX=9606 GN=CENPS	0.27	0.00019643
P04183	TK1	"Thymidine kinase, cytosolic OS=Homo sapiens OX=9606 GN=TK1"	0.254	0.00019635
P10144	GZMB	Granzyme B OS=Homo sapiens OX=9606 GN=GZMB	0.253	0.00048379
Q9BZD4	NUF2	Kinetochore protein Nuf2 OS=Homo sapiens OX=9606 GN=NUF2	0.252	8.0812E-05
P00374	DHFR	Dihydrofolate reductase OS=Homo sapiens OX=9606 GN=DHFR	0.247	0.0063833
P33552	CKS2	Cyclin-dependent kinases regulatory subunit 2 OS=Homo sapiens OX=9606 GN=CKS2	0.242	1.6537E-05
O95864	FADS2	Fatty acid desaturase 2 OS=Homo sapiens OX=9606 GN=FADS2	0.241	9.5764E-07
Q15004	PCLAF	PCNA-associated factor OS=Homo sapiens OX=9606 GN=PCLAF	0.241	6.3063E-05

Q96KB5	PBK	Lymphokine-activated killer T-cell-originated protein kinase OS=Homo sapiens OX=9606 GN=PBK	0.24	0.0051617
Q9H900	ZWILCH	Protein zwilch homolog OS=Homo sapiens OX=9606 GN=ZWILCH	0.239	4.2914E-05
O95149	SNUPN	Snurportin-1 OS=Homo sapiens OX=9606 GN=SNUPN	0.239	0.00179595
Q8NBT2	SPC24	Kinetochore protein Spc24 OS=Homo sapiens OX=9606 GN=SPC24	0.236	0.00025747
Q99661	KIF2C	Kinesin-like protein KIF2C OS=Homo sapiens OX=9606 GN=KIF2C	0.233	0.0001158
Q15003	NCAPH	Condensin complex subunit 2 OS=Homo sapiens OX=9606 GN=NCAPH	0.231	7.6071E-05
Q9BXS6	NUSAP1	Nucleolar and spindle-associated protein 1 OS=Homo sapiens OX=9606 GN=NUSAP1	0.221	4.8508E-06
Q96GX5	MASTL	Serine/threonine-protein kinase greatwall OS=Homo sapiens OX=9606 GN=MASTL	0.221	7.9914E-05
Q16222	UAP1	UDP-N-acetylhexosamine pyrophosphorylase OS=Homo sapiens OX=9606 GN=UAP1	0.214	0.0066212
P04818	TYMS	Thymidylate synthase OS=Homo sapiens OX=9606 GN=TYMS	0.213	0.00025631
P23921	RRM1	Ribonucleoside-diphosphate reductase large subunit OS=Homo sapiens OX=9606 GN=RRM1	0.211	0.0030215
Q8WWK9	CKAP2	Cytoskeleton-associated protein 2 OS=Homo sapiens OX=9606 GN=CKAP2	0.209	0.00053806
P16949	STMN1	Stathmin OS=Homo sapiens OX=9606 GN=STMN1	0.207	9.6797E-05
O60566	BUB1B	Mitotic checkpoint serine/threonine-protein kinase BUB1 beta OS=Homo sapiens OX=9606 GN=BUB1B	0.207	0.002221
P20248	CCNA2	Cyclin-A2 OS=Homo sapiens OX=9606 GN=CCNA2	0.203	0.00029845
Q9BRX5	GINS3	DNA replication complex GINS protein PSF3 OS=Homo sapiens OX=9606 GN=GINS3	0.201	0.0049355
Q93045	STMN2	Stathmin-2 OS=Homo sapiens OX=9606 GN=STMN2	0.199	0.00004087
P31350	RRM2	Ribonucleoside-diphosphate reductase subunit M2 OS=Homo sapiens OX=9606 GN=RRM2	0.194	0.00021645
Q9NPD8	UBE2T	Ubiquitin-conjugating enzyme E2 T OS=Homo sapiens OX=9606 GN=UBE2T	0.193	0.0020802
O00762	UBE2C	Ubiquitin-conjugating enzyme E2 C OS=Homo sapiens OX=9606 GN=UBE2C	0.148	0.00063753
Q71DI3	HIST2H3A	Histone H3.2 OS=Homo sapiens OX=9606 GN=HIST2H3A	0.091	0.00172026

Table S1 Differentially expressed proteins in LNCaP epiDTP cells

Table S2. Differentially expressed proteins in LNCaP enzDTP cells (>2 fold, $p<0.05$)

Accession	Gene	Protein description	Ratio	P value
A0A0B4J2F0	PIGBOS1	Protein PIGBOS1 OS=Homo sapiens OX=9606 GN=PIGBOS1	11.713	0.0050798

A0A0U1RRL7	MMP24OS	Protein MMP24OS OS=Homo sapiens OX=9606 GN=MMP24OS	9.264	0.020002
A4UGR9	XIRP2	Xin actin-binding repeat-containing protein 2 OS=Homo sapiens OX=9606 GN=XIRP2	6.095	0.00039584
A6NCL1	GMNC	Geminin coiled-coil domain-containing protein 1 OS=Homo sapiens OX=9606 GN=GMNC	6.028	2.7822E-06
A6NDG6	PGP	Glycerol-3-phosphate phosphatase OS=Homo sapiens OX=9606 GN=PGP	5.442	0.0082974
A6NJG6	ARGFX	Arginine-fifty homeobox OS=Homo sapiens OX=9606 GN=ARGFX	5.4	0.0121636
A8MSI8	LYRM9	LYR motif-containing protein 9 OS=Homo sapiens OX=9606 GN=LYRM9	4.955	2.3672E-05
O00142	TK2	"Thymidine kinase 2, mitochondrial OS=Homo sapiens OX=9606 GN=TK2"	4.521	4.308E-06
O00154	ACOT7	Cytosolic acyl coenzyme A thioester hydrolase OS=Homo sapiens OX=9606 GN=ACOT7	4.499	0.022915
O00217	NDUFS8	"NADH dehydrogenase [ubiquinone] iron-sulfur protein 8, mitochondrial OS=Homo sapiens OX=9606 GN=NDUFS8"	4.404	1.5719E-05
O00462	MANBA	Beta-mannosidase OS=Homo sapiens OX=9606 GN=MANBA	4.184	0.00019906
O00483	NDUFA4	Cytochrome c oxidase subunit NDUFA4 OS=Homo sapiens OX=9606 GN=NDUFA4	4.177	0.0031159
O00625	PIR	Pirin OS=Homo sapiens OX=9606 GN=PIR	4.162	0.00109693
O00762	UBE2C	Ubiquitin-conjugating enzyme E2 C OS=Homo sapiens OX=9606 GN=UBE2C	4.029	0.047223
O14548	COX7A2L	"Cytochrome c oxidase subunit 7A-related protein, mitochondrial OS=Homo sapiens OX=9606 GN=COX7A2L"	3.896	0.00030045
O14598	VCY	Testis-specific basic protein Y 1 OS=Homo sapiens OX=9606 GN=VCY	3.891	0.00076112
O14732	IMPA2	Inositol monophosphatase 2 OS=Homo sapiens OX=9606 GN=IMPA2	3.702	0.0113557
O14737	PDCD5	Programmed cell death protein 5 OS=Homo sapiens OX=9606 GN=PDCD5	3.637	9.0442E-07
O14773	TPP1	Tripeptidyl-peptidase 1 OS=Homo sapiens OX=9606 GN=TPP1	3.543	0.00004078
O14777	NDC80	Kinetochore protein NDC80 homolog OS=Homo sapiens OX=9606 GN=NDC80	3.499	0.0034781
O14832	PHYH	"Phytanoyl-CoA dioxygenase, peroxisomal OS=Homo sapiens OX=9606 GN=PHYH"	3.339	0.045864

O14879	IFIT3	Interferon-induced protein with tetratricopeptide repeats 3 OS=Homo sapiens OX=9606 GN=IFIT3	3.331	1.1722E-06
O14880	MGST3	Microsomal glutathione S-transferase 3 OS=Homo sapiens OX=9606 GN=MGST3	3.316	3.5297E-06
O14949	UQCRQ	Cytochrome b-c1 complex subunit 8 OS=Homo sapiens OX=9606 GN=UQCRQ	3.307	0.00091592
O14965	AURKA	Aurora kinase A OS=Homo sapiens OX=9606 GN=AURKA	3.27	0.00011745
O15067	PFAS	Phosphoribosylformylglycinamidine synthase OS=Homo sapiens OX=9606 GN=PFAS	3.248	5.8241E-05
O15230	LAMA5	Laminin subunit alpha-5 OS=Homo sapiens OX=9606 GN=LAMA5	3.207	0.0021021
O15305	PMM2	Phosphomannomutase 2 OS=Homo sapiens OX=9606 GN=PMM2	3.194	4.7486E-06
O15318	POLR3G	DNA-directed RNA polymerase III subunit RPC7 OS=Homo sapiens OX=9606 GN=POLR3G	3.184	3.7001E-06
O15394	NCAM2	Neural cell adhesion molecule 2 OS=Homo sapiens OX=9606 GN=NCAM2	3.138	0.0162386
O15484	CAPN5	Calpain-5 OS=Homo sapiens OX=9606 GN=CAPN5	3.135	1.9528E-05
O15533	TAPBP	Tapasin OS=Homo sapiens OX=9606 GN=TAPBP	3.092	0.00021812
O43181	NDUFS4	"NADH dehydrogenase [ubiquinone] iron-sulfur protein 4, mitochondrial OS=Homo sapiens OX=9606 GN=NDUFS4"	3.089	3.7962E-07
O43299	AP5Z1	AP-5 complex subunit zeta-1 OS=Homo sapiens OX=9606 GN=AP5Z1	3.068	2.5546E-06
O43663	PRC1	Protein regulator of cytokinesis 1 OS=Homo sapiens OX=9606 GN=PRC1	3.051	0.00043733
O43676	NDUFB3	NADH dehydrogenase [ubiquinone] 1 beta subcomplex subunit 3 OS=Homo sapiens OX=9606 GN=NDUFB3	2.986	0.00013622
O43677	NDUFC1	"NADH dehydrogenase [ubiquinone] 1 subunit C1, mitochondrial OS=Homo sapiens OX=9606 GN=NDUFC1"	2.936	1.8192E-07
O43692	PI15	Peptidase inhibitor 15 OS=Homo sapiens OX=9606 GN=PI15	2.914	0.00069872
O43761	SYNGR3	Synaptogyrin-3 OS=Homo sapiens OX=9606 GN=SYNGR3	2.888	3.387E-06
O43805	SSNA1	Sjoegren syndrome nuclear autoantigen 1 OS=Homo sapiens OX=9606 GN=SSNA1	2.858	0.00022313
O43808	SLC25A17	Peroxisomal membrane protein PMP34 OS=Homo sapiens OX=9606 GN=SLC25A17	2.855	0.00075559

O43847	NRDC	Nardilysin OS=Homo sapiens OX=9606 GN=NRDC	2.84	0.00151841
O60237	PPP1R12B	Protein phosphatase 1 regulatory subunit 12B OS=Homo sapiens OX=9606 GN=PPP1R12B	2.799	0.00023783
O60262	GNG7	Guanine nucleotide-binding protein G(I)/G(S)/G(O) subunit gamma-7 OS=Homo sapiens OX=9606 GN=GNG7	2.799	1.8889E-05
O60333	KIF1B	Kinesin-like protein KIF1B OS=Homo sapiens OX=9606 GN=KIF1B	2.784	8.7612E-07
O60566	BUB1B	Mitotic checkpoint serine/threonine-protein kinase BUB1 beta OS=Homo sapiens OX=9606 GN=BUB1B	2.774	2.497E-06
O60613	SELENOF	Selenoprotein F OS=Homo sapiens OX=9606 GN=SELENOF	2.771	2.2735E-06
O60658	PDE8A	"High affinity cAMP-specific and IBMX-insensitive 3',5'-cyclic phosphodiesterase 8A OS=Homo sapiens OX=9606 GN=PDE8A"	2.761	4.8128E-06
O75192	PEX11A	Peroxisomal membrane protein 11A OS=Homo sapiens OX=9606 GN=PEX11A	2.755	2.1347E-05
O75298	RTN2	Reticulon-2 OS=Homo sapiens OX=9606 GN=RTN2	2.754	4.4168E-05
O75310	UGT2B11	UDP-glucuronosyltransferase 2B11 OS=Homo sapiens OX=9606 GN=UGT2B11	2.752	4.9514E-06
O75330	HMMR	Hyaluronan mediated motility receptor OS=Homo sapiens OX=9606 GN=HMMR	2.744	0.00131701
O75347	TBCA	Tubulin-specific chaperone A OS=Homo sapiens OX=9606 GN=TBCA	2.741	0.049863
O75363	BCAS1	Breast carcinoma-amplified sequence 1 OS=Homo sapiens OX=9606 GN=BCAS1	2.731	2.1741E-05
O75380	NDUFS6	"NADH dehydrogenase [ubiquinone] iron-sulfur protein 6, mitochondrial OS=Homo sapiens OX=9606 GN=NDUFS6"	2.721	0.00048041
O75419	CDC45	Cell division control protein 45 homolog OS=Homo sapiens OX=9606 GN=CDC45	2.719	2.2292E-05
O75445	USH2A	Usherin OS=Homo sapiens OX=9606 GN=USH2A	2.703	1.1368E-06
O75449	KATNA1	Katanin p60 ATPase-containing subunit A1 OS=Homo sapiens OX=9606 GN=KATNA1	2.699	0.00113627
O75489	NDUFS3	"NADH dehydrogenase [ubiquinone] iron-sulfur protein 3, mitochondrial OS=Homo sapiens OX=9606 GN=NDUFS3"	2.678	0.0001753
O75496	GMNN	Geminin OS=Homo sapiens OX=9606	2.677	0.00018186

		GN=GMNN		
O75509	TNFRSF21	Tumor necrosis factor receptor superfamily member 21 OS=Homo sapiens OX=9606 GN=TNFRSF21	2.666	3.6165E-06
O75534	CSDE1	Cold shock domain-containing protein E1 OS=Homo sapiens OX=9606 GN=CSDE1	2.632	3.7713E-05
O75717	WDHD1	WD repeat and HMG-box DNA-binding protein 1 OS=Homo sapiens OX=9606 GN=WDHD1	2.612	0.00153923
O75794	CDC123	Cell division cycle protein 123 homolog OS=Homo sapiens OX=9606 GN=CDC123	2.612	1.8358E-05
O75795	UGT2B17	UDP-glucuronosyltransferase 2B17 OS=Homo sapiens OX=9606 GN=UGT2B17	2.602	3.6154E-06
O75911	DHRS3	Short-chain dehydrogenase/reductase 3 OS=Homo sapiens OX=9606 GN=DHRS3	2.595	1.3846E-06
O75955	FLOT1	Flotillin-1 OS=Homo sapiens OX=9606 GN=FLOT1	2.594	2.1459E-05
O76003	GLRX3	Glutaredoxin-3 OS=Homo sapiens OX=9606 GN=GLRX3	2.585	0.00013948
O76039	CDKL5	Cyclin-dependent kinase-like 5 OS=Homo sapiens OX=9606 GN=CDKL5	2.582	1.7339E-05
O76062	TM7SF2	Delta(14)-sterol reductase OS=Homo sapiens OX=9606 GN=TM7SF2	2.562	1.8405E-05
O76080	ZFAND5	AN1-type zinc finger protein 5 OS=Homo sapiens OX=9606 GN=ZFAND5	2.56	1.9445E-05
O94804	STK10	Serine/threonine-protein kinase 10 OS=Homo sapiens OX=9606 GN=STK10	2.56	6.4844E-05
O94851	MICAL2	[F-actin]-monooxygenase MICAL2 OS=Homo sapiens OX=9606 GN=MICAL2	2.556	0.00080012
O94915	FRYL	Protein furry homolog-like OS=Homo sapiens OX=9606 GN=FRYL	2.551	0.00172359
O95149	SNUPN	Snurportin-1 OS=Homo sapiens OX=9606 GN=SNUPN	2.548	0.00014279
O95167	NDUFA3	NADH dehydrogenase [ubiquinone] 1 alpha subcomplex subunit 3 OS=Homo sapiens OX=9606 GN=NDUFA3	2.548	0.00169981
O95169	NDUFB8	"NADH dehydrogenase [ubiquinone] 1 beta subcomplex subunit 8, mitochondrial OS=Homo sapiens OX=9606 GN=NDUFB8"	2.546	2.4369E-05
O95235	KIF20A	Kinesin-like protein KIF20A OS=Homo sapiens OX=9606 GN=KIF20A	2.542	8.4564E-07
O95239	KIF4A	Chromosome-associated kinesin KIF4A OS=Homo sapiens OX=9606	2.539	2.3442E-05

		GN=KIF4A		
O95299	NDUFA10	"NADH dehydrogenase [ubiquinone] 1 alpha subcomplex subunit 10, mitochondrial OS=Homo sapiens OX=9606 GN=NDUFA10"	2.536	2.0016E-08
O95347	SMC2	Structural maintenance of chromosomes protein 2 OS=Homo sapiens OX=9606 GN=SMC2	2.529	0.021963
O95372	LYPLA2	Acyl-protein thioesterase 2 OS=Homo sapiens OX=9606 GN=LYPLA2	2.517	1.6146E-05
O95433	AHSA1	Activator of 90 kDa heat shock protein ATPase homolog 1 OS=Homo sapiens OX=9606 GN=AHSA1	2.495	8.4117E-05
O95445	APOM	Apolipoprotein M OS=Homo sapiens OX=9606 GN=APOM	2.487	5.8277E-05
O95801	TTC4	Tetratricopeptide repeat protein 4 OS=Homo sapiens OX=9606 GN=TTC4	2.478	0.0060836
O95817	BAG3	BAG family molecular chaperone regulator 3 OS=Homo sapiens OX=9606 GN=BAG3	2.478	0.00185709
O95858	TSPAN15	Tetraspanin-15 OS=Homo sapiens OX=9606 GN=TSPAN15	2.475	1.6937E-07
O95864	FADS2	Fatty acid desaturase 2 OS=Homo sapiens OX=9606 GN=FADS2	2.474	5.2566E-07
P00156	MT-CYB	Cytochrome b OS=Homo sapiens OX=9606 GN=MT-CYB	2.472	6.0924E-07
P00338	LDHA	L-lactate dehydrogenase A chain OS=Homo sapiens OX=9606 GN=LDHA	2.472	0.0020755
P00367	GLUD1	"Glutamate dehydrogenase 1, mitochondrial OS=Homo sapiens OX=9606 GN=GLUD1"	2.47	8.3352E-05
P00374	DHFR	Dihydrofolate reductase OS=Homo sapiens OX=9606 GN=DHFR	2.468	0.00012226
P00403	MT-CO2	Cytochrome c oxidase subunit 2 OS=Homo sapiens OX=9606 GN=MT-CO2	2.456	0.0055754
P00488	F13A1	Coagulation factor XIII A chain OS=Homo sapiens OX=9606 GN=F13A1	2.448	1.9393E-05
P00491	PNP	Purine nucleoside phosphorylase OS=Homo sapiens OX=9606 GN=PNP	2.444	2.3447E-05
P00492	HPRT1	Hypoxanthine-guanine phosphoribosyltransferase OS=Homo sapiens OX=9606 GN=HPRT1	2.443	1.8414E-05
P00973	OAS1	2'-5'-oligoadenylate synthase 1 OS=Homo sapiens OX=9606 GN=OAS1	2.44	1.1918E-06
P01130	LDLR	Low-density lipoprotein receptor OS=Homo sapiens OX=9606 GN=LDLR	2.437	0.00043893
P01833	PIGR	Polymeric immunoglobulin receptor OS=Homo sapiens OX=9606 GN=PIGR	2.428	3.125E-06
P04040	CAT	Catalase OS=Homo sapiens OX=9606 GN=CAT	2.427	6.7945E-07
P04066	FUCA1	Tissue alpha-L-fucosidase OS=Homo	2.425	1.7304E-05

		sapiens OX=9606 GN=FUCA1		
P04083	ANXA1	Annexin A1 OS=Homo sapiens OX=9606 GN=ANXA1	2.425	1.5661E-07
P04179	SOD2	"Superoxide dismutase [Mn], mitochondrial OS=Homo sapiens OX=9606 GN=SOD2"	2.425	1.7977E-05
P04183	TK1	"Thymidine kinase, cytosolic OS=Homo sapiens OX=9606 GN=TK1"	2.423	1.0286E-06
P04792	HSPB1	Heat shock protein beta-1 OS=Homo sapiens OX=9606 GN=HSPB1	2.42	6.3262E-05
P04818	TYMS	Thymidylate synthase OS=Homo sapiens OX=9606 GN=TYMS	2.42	1.3132E-06
P05091	ALDH2	"Aldehyde dehydrogenase, mitochondrial OS=Homo sapiens OX=9606 GN=ALDH2"	2.417	1.5412E-05
P05165	PCCA	"Propionyl-CoA carboxylase alpha chain, mitochondrial OS=Homo sapiens OX=9606 GN=PCCA"	2.413	0.035377
P05166	PCCB	"Propionyl-CoA carboxylase beta chain, mitochondrial OS=Homo sapiens OX=9606 GN=PCCB"	2.412	4.4445E-05
P06454	PTMA	Prothymosin alpha OS=Homo sapiens OX=9606 GN=PTMA	2.409	0.00012234
P06493	CDK1	Cyclin-dependent kinase 1 OS=Homo sapiens OX=9606 GN=CDK1	2.404	2.3043E-06
P06733	ENO1	Alpha-enolase OS=Homo sapiens OX=9606 GN=ENO1	2.404	1.5626E-05
P06744	GPI	Glucose-6-phosphate isomerase OS=Homo sapiens OX=9606 GN=GPI	2.399	1.7751E-05
P06865	HEXA	Beta-hexosaminidase subunit alpha OS=Homo sapiens OX=9606 GN=HEXA	2.398	4.6812E-06
P07099	EPHX1	Epoxide hydrolase 1 OS=Homo sapiens OX=9606 GN=EPHX1	2.398	0.042596
P07108	DBI	Acyl-CoA-binding protein OS=Homo sapiens OX=9606 GN=DBI	2.396	8.1995E-08
P07195	LDHB	L-lactate dehydrogenase B chain OS=Homo sapiens OX=9606 GN=LDHB	2.394	1.6686E-05
P07203	GPX1	Glutathione peroxidase 1 OS=Homo sapiens OX=9606 GN=GPX1	2.392	0.00035694
P07339	CTSD	Cathepsin D OS=Homo sapiens OX=9606 GN=CTSD	2.391	1.7689E-06
P07602	PSAP	Prosaposin OS=Homo sapiens OX=9606 GN=PSAP	2.388	3.8921E-05
P07737	PFN1	Profilin-1 OS=Homo sapiens OX=9606 GN=PFN1	2.375	3.9914E-05
P07858	CTSB	Cathepsin B OS=Homo sapiens OX=9606 GN=CTSB	2.367	0.00004126
P08174	CD55	Complement decay-accelerating factor OS=Homo sapiens OX=9606 GN=CD55	2.367	2.0636E-05
P08238	HSP90AB1	Heat shock protein HSP 90-beta OS=Homo sapiens OX=9606 GN=HSP90AB1	2.366	0.029845

P08243	ASNS	Asparagine synthetase [glutamine-hydrolyzing] OS=Homo sapiens OX=9606 GN=ASNS	2.364	0.024575
P08473	MME	Neprilysin OS=Homo sapiens OX=9606 GN=MME	2.356	4.3109E-05
P08910	ABHD2	Monoacylglycerol lipase ABHD2 OS=Homo sapiens OX=9606 GN=ABHD2	2.353	2.8022E-06
P08913	ADRA2A	Alpha-2A adrenergic receptor OS=Homo sapiens OX=9606 GN=ADRA2A	2.35	0.00028286
P09110	ACAA1	"3-ketoacyl-CoA thiolase, peroxisomal OS=Homo sapiens OX=9606 GN=ACAA1"	2.35	2.3619E-05
P09669	COX6C	Cytochrome c oxidase subunit 6C OS=Homo sapiens OX=9606 GN=COX6C	2.346	1.4568E-06
P09913	IFIT2	Interferon-induced protein with tetratricopeptide repeats 2 OS=Homo sapiens OX=9606 GN=IFIT2	2.339	6.0566E-05
P09914	IFIT1	Interferon-induced protein with tetratricopeptide repeats 1 OS=Homo sapiens OX=9606 GN=IFIT1	2.338	4.4422E-06
P0CG24	ZNF883	Zinc finger protein 883 OS=Homo sapiens OX=9606 GN=ZNF883	2.338	2.0797E-05
P0DPI2	GATD3A	"Glutamine amidotransferase-like class 1 domain-containing protein 3A, mitochondrial OS=Homo sapiens OX=9606 GN=GATD3A"	2.331	1.5717E-05
P10109	FDX1	"Adrenodoxin, mitochondrial OS=Homo sapiens OX=9606 GN=FDX1"	2.323	0.00003952
P10144	GZMB	Granzyme B OS=Homo sapiens OX=9606 GN=GZMB	2.315	5.8065E-05
P10412	HIST1H1E	Histone H1.4 OS=Homo sapiens OX=9606 GN=HIST1H1E	2.314	6.4488E-07
P10632	CYP2C8	Cytochrome P450 2C8 OS=Homo sapiens OX=9606 GN=CYP2C8	2.311	3.7348E-06
P11182	DBT	"Lipoamide acyltransferase component of branched-chain alpha-keto acid dehydrogenase complex, mitochondrial OS=Homo sapiens OX=9606 GN=DBT"	2.31	2.4864E-05
P11234	RALB	Ras-related protein Ral-B OS=Homo sapiens OX=9606 GN=RALB	2.309	1.3381E-06
P11310	ACADM	"Medium-chain specific acyl-CoA dehydrogenase, mitochondrial OS=Homo sapiens OX=9606 GN=ACADM"	2.307	3.7661E-06
P11388	TOP2A	DNA topoisomerase 2-alpha OS=Homo sapiens OX=9606 GN=TOP2A	2.303	8.4421E-05
P11532	DMD	Dystrophin OS=Homo sapiens OX=9606 GN=DMD	2.293	0.00118218
P11586	MTHFD1	"C-1-tetrahydrofolate synthase, cytoplasmic OS=Homo sapiens OX=9606 GN=MTHFD1"	2.293	0.0003958

P11678	EPX	Eosinophil peroxidase OS=Homo sapiens OX=9606 GN=EPX	2.292	0.0000228
P12004	PCNA	Proliferating cell nuclear antigen OS=Homo sapiens OX=9606 GN=PCNA	2.291	3.0135E-07
P12074	COX6A1	"Cytochrome c oxidase subunit 6A1, mitochondrial OS=Homo sapiens OX=9606 GN=COX6A1"	2.289	0.00033623
P12081	HARS	"Histidine--tRNA ligase, cytoplasmic OS=Homo sapiens OX=9606 GN=HARS"	2.289	0.0032648
P12235	SLC25A4	ADP/ATP translocase 1 OS=Homo sapiens OX=9606 GN=SLC25A4	2.288	4.4839E-07
P13501	CCL5	C-C motif chemokine 5 OS=Homo sapiens OX=9606 GN=CCL5	2.288	0.00026028
P13639	EEF2	Elongation factor 2 OS=Homo sapiens OX=9606 GN=EEF2	2.287	7.5099E-05
P13693	TPT1	Translationally-controlled tumor protein OS=Homo sapiens OX=9606 GN=TPT1	2.284	0.00012213
P13797	PLS3	Plastin-3 OS=Homo sapiens OX=9606 GN=PLS3	2.283	0.0030997
P13798	APEH	Acylamino-acid-releasing enzyme OS=Homo sapiens OX=9606 GN=APEH	2.277	0.00082486
P14324	FDPS	Farnesyl pyrophosphate synthase OS=Homo sapiens OX=9606 GN=FDPS	2.276	8.7179E-07
P14406	COX7A2	"Cytochrome c oxidase subunit 7A2, mitochondrial OS=Homo sapiens OX=9606 GN=COX7A2"	2.272	2.2866E-05
P14543	NID1	Nidogen-1 OS=Homo sapiens OX=9606 GN=NID1	2.271	5.9442E-05
P14618	PKM	Pyruvate kinase PKM OS=Homo sapiens OX=9606 GN=PKM	2.27	0.0038427
P14635	CCNB1	G2/mitotic-specific cyclin-B1 OS=Homo sapiens OX=9606 GN=CCNB1	2.262	1.2012E-06
P14784	IL2RB	Interleukin-2 receptor subunit beta OS=Homo sapiens OX=9606 GN=IL2RB	2.254	0.00014293
P14854	COX6B1	Cytochrome c oxidase subunit 6B1 OS=Homo sapiens OX=9606 GN=COX6B1	2.253	8.9848E-07
P14927	UQCRB	Cytochrome b-c1 complex subunit 7 OS=Homo sapiens OX=9606 GN=UQCRB	2.25	0.00012292
P15170	GSPT1	Eukaryotic peptide chain release factor GTP-binding subunit ERF3A OS=Homo sapiens OX=9606 GN=GSPT1	2.248	1.5518E-06
P15289	ARSA	Arylsulfatase A OS=Homo sapiens OX=9606 GN=ARSA	2.245	0.00138294
P15309	ACPP	Prostatic acid phosphatase OS=Homo sapiens OX=9606 GN=ACPP	2.245	0.00012014
P15954	COX7C	"Cytochrome c oxidase subunit 7C, mitochondrial OS=Homo sapiens OX=9606 GN=COX7C"	2.245	1.5517E-05
P16066	NPR1	Atrial natriuretic peptide receptor 1	2.244	0.00016009

		OS=Homo sapiens OX=9606 GN=NPR1		
P16260	SLC25A16	Graves disease carrier protein OS=Homo sapiens OX=9606 GN=SLC25A16	2.243	1.8797E-06
P16401	HIST1H1B	Histone H1.5 OS=Homo sapiens OX=9606 GN=HIST1H1B	2.243	0.00063795
P16949	STMN1	Stathmin OS=Homo sapiens OX=9606 GN=STMN1	2.242	0.00010322
P17174	GOT1	"Aspartate aminotransferase, cytoplasmic OS=Homo sapiens OX=9606 GN=GOT1"	2.242	0.0027018
P17568	NDUFB7	NADH dehydrogenase [ubiquinone] 1 beta subcomplex subunit 7 OS=Homo sapiens OX=9606 GN=NDUFB7	2.242	0.04968
P17693	HLA-G	"HLA class I histocompatibility antigen, alpha chain G OS=Homo sapiens OX=9606 GN=HLA-G"	2.241	1.7592E-06
P17812	CTPS1	CTP synthase 1 OS=Homo sapiens OX=9606 GN=CTPS1	2.241	1.6997E-06
P18124	RPL7	60S ribosomal protein L7 OS=Homo sapiens OX=9606 GN=RPL7	2.235	9.9033E-05
P18463	HLA-B	"HLA class I histocompatibility antigen, B-37 alpha chain OS=Homo sapiens OX=9606 GN=HLA-B"	2.235	1.9092E-06
P18858	LIG1	DNA ligase 1 OS=Homo sapiens OX=9606 GN=LIG1	2.23	0.00005564
P19174	PLCG1	"1-phosphatidylinositol 4,5-bisphosphate phosphodiesterase gamma-1 OS=Homo sapiens OX=9606 GN=PLCG1"	2.229	1.5495E-05
P19623	SRM	Spermidine synthase OS=Homo sapiens OX=9606 GN=SRM	2.226	0.026541
P20020	ATP2B1	Plasma membrane calcium-transporting ATPase 1 OS=Homo sapiens OX=9606 GN=ATP2B1	2.221	0.0022152
P20248	CCNA2	Cyclin-A2 OS=Homo sapiens OX=9606 GN=CCNA2	2.218	2.3658E-05
P20839	IMPDH1	Inosine-5'-monophosphate dehydrogenase 1 OS=Homo sapiens OX=9606 GN=IMPDH1	2.214	4.0492E-05
P21291	CSRP1	Cysteine and glycine-rich protein 1 OS=Homo sapiens OX=9606 GN=CSRP1	2.214	2.0942E-05
P21397	MAOA	Amine oxidase [flavin-containing] A OS=Homo sapiens OX=9606 GN=MAOA	2.213	0.0045425
P21953	BCKDHB	"2-oxoisovalerate dehydrogenase subunit beta, mitochondrial OS=Homo sapiens OX=9606 GN=BCKDHB"	2.212	0.00136086
P22102	GART	Trifunctional purine biosynthetic protein adenosine-3 OS=Homo sapiens OX=9606 GN=GART	2.211	7.7658E-05
P22234	PAICS	Multifunctional protein ADE2 OS=Homo sapiens OX=9606 GN=PAICS	2.207	0.00041711

P22307	SCP2	Non-specific lipid-transfer protein OS=Homo sapiens OX=9606 GN=SCP2	2.203	0.0024018
P23141	CES1	Liver carboxylesterase 1 OS=Homo sapiens OX=9606 GN=CES1	2.196	6.6065E-07
P23528	CFL1	Cofilin-1 OS=Homo sapiens OX=9606 GN=CFL1	2.196	3.7034E-05
P23786	CPT2	"Carnitine O-palmitoyltransferase 2, mitochondrial OS=Homo sapiens OX=9606 GN=CPT2"	2.196	0.011404
P23921	RRM1	Ribonucleoside-diphosphate reductase large subunit OS=Homo sapiens OX=9606 GN=RRM1	2.19	1.6819E-05
P24752	ACAT1	"Acetyl-CoA acetyltransferase, mitochondrial OS=Homo sapiens OX=9606 GN=ACAT1"	2.19	1.602E-05
P25205	MCM3	DNA replication licensing factor MCM3 OS=Homo sapiens OX=9606 GN=MCM3	2.189	1.6337E-05
P25774	CTSS	Cathepsin S OS=Homo sapiens OX=9606 GN=CTSS	2.188	2.1816E-05
P26358	DNMT1	DNA (cytosine-5)-methyltransferase 1 OS=Homo sapiens OX=9606 GN=DNMT1	2.185	4.0653E-06
P26639	TARS	"Threonine--tRNA ligase, cytoplasmic OS=Homo sapiens OX=9606 GN=TARS"	2.182	4.3419E-07
P27338	MAOB	Amine oxidase [flavin-containing] B OS=Homo sapiens OX=9606 GN=MAOB	2.182	3.4664E-08
P27348	YWHAQ	14-3-3 protein theta OS=Homo sapiens OX=9606 GN=YWHAQ	2.18	0.00011594
P27701	CD82	CD82 antigen OS=Homo sapiens OX=9606 GN=CD82	2.18	5.3174E-08
P27707	DCK	Deoxycytidine kinase OS=Homo sapiens OX=9606 GN=DCK	2.175	1.5055E-05
P28288	ABCD3	ATP-binding cassette sub-family D member 3 OS=Homo sapiens OX=9606 GN=ABCD3	2.174	9.8093E-05
P28328	PEX2	Peroxisome biogenesis factor 2 OS=Homo sapiens OX=9606 GN=PEX2	2.173	5.6575E-07
P30520	ADSS	Adenylosuccinate synthetase isozyme 2 OS=Homo sapiens OX=9606 GN=ADSS	2.17	5.6489E-05
P30566	ADSL	Adenylosuccinate lyase OS=Homo sapiens OX=9606 GN=ADSL	2.166	3.8671E-07
P31327	CPS1	"Carbamoyl-phosphate synthase [ammonia], mitochondrial OS=Homo sapiens OX=9606 GN=CPS1"	2.163	1.6167E-06
P31350	RRM2	Ribonucleoside-diphosphate reductase subunit M2 OS=Homo sapiens OX=9606 GN=RRM2	2.16	0.0004354
P31431	SDC4	Syndecan-4 OS=Homo sapiens OX=9606 GN=SDC4	2.153	0.00043603

P31937	HIBADH	"3-hydroxyisobutyrate dehydrogenase, mitochondrial OS=Homo sapiens OX=9606 GN=HIBADH"	2.15	1.8693E-06
P31948	STIP1	Stress-induced-phosphoprotein 1 OS=Homo sapiens OX=9606 GN=STIP1	2.149	5.4743E-07
P31949	S100A11	Protein S100-A11 OS=Homo sapiens OX=9606 GN=S100A11	2.148	8.4386E-05
P33121	ACSL1	Long-chain-fatty-acid--CoA ligase 1 OS=Homo sapiens OX=9606 GN=ACSL1	2.147	0.0183972
P33552	CKS2	Cyclin-dependent kinases regulatory subunit 2 OS=Homo sapiens OX=9606 GN=CKS2	2.147	0.0148975
P33981	TTK	Dual specificity protein kinase TTK OS=Homo sapiens OX=9606 GN=TTK	2.14	3.7582E-06
P33991	MCM4	DNA replication licensing factor MCM4 OS=Homo sapiens OX=9606 GN=MCM4	2.137	3.9462E-06
P33992	MCM5	DNA replication licensing factor MCM5 OS=Homo sapiens OX=9606 GN=MCM5	2.134	3.2425E-06
P33993	MCM7	DNA replication licensing factor MCM7 OS=Homo sapiens OX=9606 GN=MCM7	2.131	0.00026296
P35475	IDUA	Alpha-L-iduronidase OS=Homo sapiens OX=9606 GN=IDUA	2.131	3.2335E-06
P35914	HMGCL	"Hydroxymethylglutaryl-CoA lyase, mitochondrial OS=Homo sapiens OX=9606 GN=HMGCL"	2.127	2.5111E-06
P36776	LONP1	"Lon protease homolog, mitochondrial OS=Homo sapiens OX=9606 GN=LONP1"	2.124	8.8336E-08
P37802	TAGLN2	Transgelin-2 OS=Homo sapiens OX=9606 GN=TAGLN2	2.122	1.6133E-05
P38570	ITGAE	Integrin alpha-E OS=Homo sapiens OX=9606 GN=ITGAE	2.122	2.5451E-07
P39210	MPV17	Protein Mpv17 OS=Homo sapiens OX=9606 GN=MPV17	2.121	2.2946E-06
P39748	FEN1	Flap endonuclease 1 OS=Homo sapiens OX=9606 GN=FEN1	2.12	0.00011538
P40939	HADHA	"Trifunctional enzyme subunit alpha, mitochondrial OS=Homo sapiens OX=9606 GN=HADHA"	2.119	6.2831E-05
P41227	NAA10	N-alpha-acetyltransferase 10 OS=Homo sapiens OX=9606 GN=NAA10	2.113	1.928E-06
P41247	PNPLA4	Patatin-like phospholipase domain-containing protein 4 OS=Homo sapiens OX=9606 GN=PNPLA4	2.112	1.8328E-05
P42126	ECI1	"Enoyl-CoA delta isomerase 1, mitochondrial OS=Homo sapiens OX=9606 GN=ECI1"	2.112	0.0034003
P43251	BTD	Biotinidase OS=Homo sapiens OX=9606	2.106	4.4557E-07

		GN=BTD		
P43358	MAGEA4	Melanoma-associated antigen 4 OS=Homo sapiens OX=9606 GN=MAGEA4	2.103	0.00015588
P43487	RANBP1	Ran-specific GTPase-activating protein OS=Homo sapiens OX=9606 GN=RANBP1	2.102	0.00020136
P45954	ACADSB	"Short/branched chain specific acyl-CoA dehydrogenase, mitochondrial OS=Homo sapiens OX=9606 GN=ACADSB"	2.1	0.00052022
P46013	MKI67	Proliferation marker protein Ki-67 OS=Homo sapiens OX=9606 GN=MKI67	2.098	3.7187E-06
P46109	CRKL	Crk-like protein OS=Homo sapiens OX=9606 GN=CRKL	2.098	0.00015754
P46734	MAP2K3	Dual specificity mitogen-activated protein kinase kinase 3 OS=Homo sapiens OX=9606 GN=MAP2K3	2.095	0.0029558
P46781	RPS9	40S ribosomal protein S9 OS=Homo sapiens OX=9606 GN=RPS9	2.092	3.4742E-07
P46821	MAP1B	Microtubule-associated protein 1B OS=Homo sapiens OX=9606 GN=MAP1B	2.091	0.00028258
P48739	PITPNB	Phosphatidylinositol transfer protein beta isoform OS=Homo sapiens OX=9606 GN=PITPNB	2.089	4.0879E-07
P49023	PXN	Paxillin OS=Homo sapiens OX=9606 GN=PXN	2.089	0.025005
P49321	NASP	Nuclear autoantigenic sperm protein OS=Homo sapiens OX=9606 GN=NASP	2.087	0.00017791
P49327	FASN	Fatty acid synthase OS=Homo sapiens OX=9606 GN=FASN	2.084	0.00168118
P49736	MCM2	DNA replication licensing factor MCM2 OS=Homo sapiens OX=9606 GN=MCM2	2.084	0.00022284
P49748	ACADVL	"Very long-chain specific acyl-CoA dehydrogenase, mitochondrial OS=Homo sapiens OX=9606 GN=ACADVL"	2.081	0.00193734
P49840	GSK3A	Glycogen synthase kinase-3 alpha OS=Homo sapiens OX=9606 GN=GSK3A	2.08	3.9125E-05
P50395	GDI2	Rab GDP dissociation inhibitor beta OS=Homo sapiens OX=9606 GN=GDI2	2.08	1.6452E-05
P50579	METAP2	Methionine aminopeptidase 2 OS=Homo sapiens OX=9606 GN=METAP2	2.078	2.5526E-06
P50748	KNTC1	Kinetochore-associated protein 1 OS=Homo sapiens OX=9606 GN=KNTC1	2.077	1.9102E-06
P50895	BCAM	Basal cell adhesion molecule OS=Homo sapiens OX=9606 GN=BCAM	2.076	0.0073963
P50914	RPL14	60S ribosomal protein L14 OS=Homo sapiens OX=9606 GN=RPL14	2.069	2.7019E-06

P50990	CCT8	T-complex protein 1 subunit theta OS=Homo sapiens OX=9606 GN=CCT8	2.067	4.0462E-05
P51648	ALDH3A2	Fatty aldehyde dehydrogenase OS=Homo sapiens OX=9606 GN=ALDH3A2	2.067	0.00074147
P51649	ALDH5A1	"Succinate-semialdehyde dehydrogenase, mitochondrial OS=Homo sapiens OX=9606 GN=ALDH5A1"	2.065	2.0514E-05
P52292	KPNA2	Importin subunit alpha-1 OS=Homo sapiens OX=9606 GN=KPNA2	2.064	4.7561E-06
P52565	ARHGDIA	Rho GDP-dissociation inhibitor 1 OS=Homo sapiens OX=9606 GN=ARHGDIA	2.063	0.00012179
P52732	KIF11	Kinesin-like protein KIF11 OS=Homo sapiens OX=9606 GN=KIF11	2.063	4.1585E-06
P52797	EFNA3	Ephrin-A3 OS=Homo sapiens OX=9606 GN=EFNA3	2.062	6.0176E-05
P53384	NUBP1	Cytosolic Fe-S cluster assembly factor NUBP1 OS=Homo sapiens OX=9606 GN=NUBP1	2.062	2.4974E-05
P53396	ACLY	ATP-citrate synthase OS=Homo sapiens OX=9606 GN=ACLY	2.061	0.00198396
P53801	PTTG1IP	Pituitary tumor-transforming gene 1 protein-interacting protein OS=Homo sapiens OX=9606 GN=PTTG1IP	2.06	2.2837E-05
P54868	HMGCS2	"Hydroxymethylglutaryl-CoA synthase, mitochondrial OS=Homo sapiens OX=9606 GN=HMGCS2"	2.058	3.7879E-05
P55060	CSE1L	Exportin-2 OS=Homo sapiens OX=9606 GN=CSE1L	2.057	3.7534E-05
P55073	DIO3	Thyroxine 5-deiodinase OS=Homo sapiens OX=9606 GN=DIO3	2.057	5.7153E-05
P55084	HADHB	"Trifunctional enzyme subunit beta, mitochondrial OS=Homo sapiens OX=9606 GN=HADHB"	2.056	0.00030435
P55209	NAP1L1	Nucleosome assembly protein 1-like 1 OS=Homo sapiens OX=9606 GN=NAP1L1	2.054	4.2805E-07
P56181	NDUFV3	"NADH dehydrogenase [ubiquinone] flavoprotein 3, mitochondrial OS=Homo sapiens OX=9606 GN=NDUFV3"	2.05	1.6551E-05
P56277	CMC4	Cx9C motif-containing protein 4 OS=Homo sapiens OX=9606 GN=CMC4	2.05	0.00012425
P57076	CFAP298	Cilia- and flagella-associated protein 298 OS=Homo sapiens OX=9606 GN=CFAP298	2.049	2.7241E-07
P58004	SESN2	Sestrin-2 OS=Homo sapiens OX=9606 GN=SESN2	2.047	1.6267E-05
P60842	EIF4A1	Eukaryotic initiation factor 4A-I OS=Homo sapiens OX=9606 GN=EIF4A1	2.044	2.3044E-06
P60891	PRPS1	Ribose-phosphate pyrophosphokinase 1 OS=Homo sapiens OX=9606	2.043	2.1593E-05

		GN=PRPS1		
P61024	CKS1B	Cyclin-dependent kinases regulatory subunit 1 OS=Homo sapiens OX=9606 GN=CKS1B	2.043	0.0064386
P61221	ABCE1	ATP-binding cassette sub-family E member 1 OS=Homo sapiens OX=9606 GN=ABCE1	2.043	0.00194003
P61758	VBP1	Prefoldin subunit 3 OS=Homo sapiens OX=9606 GN=VBP1	2.04	0.0027752
P61916	NPC2	NPC intracellular cholesterol transporter 2 OS=Homo sapiens OX=9606 GN=NPC2	2.036	0.00038351
P62068	USP46	Ubiquitin carboxyl-terminal hydrolase 46 OS=Homo sapiens OX=9606 GN=USP46	2.036	5.8586E-05
P62081	RPS7	40S ribosomal protein S7 OS=Homo sapiens OX=9606 GN=RPS7	2.035	2.6796E-06
P62253	UBE2G1	Ubiquitin-conjugating enzyme E2 G1 OS=Homo sapiens OX=9606 GN=UBE2G1	2.035	2.3717E-05
P62273	RPS29	40S ribosomal protein S29 OS=Homo sapiens OX=9606 GN=RPS29	2.032	6.4821E-05
P62495	ETF1	Eukaryotic peptide chain release factor subunit 1 OS=Homo sapiens OX=9606 GN=ETF1	2.03	8.7432E-07
P62847	RPS24	40S ribosomal protein S24 OS=Homo sapiens OX=9606 GN=RPS24	2.03	0.00011547
P62857	RPS28	40S ribosomal protein S28 OS=Homo sapiens OX=9606 GN=RPS28	2.03	0.00195819
P62861	FAU	40S ribosomal protein S30 OS=Homo sapiens OX=9606 GN=FAU	2.028	6.1485E-05
P62937	PPIA	Peptidyl-prolyl cis-trans isomerase A OS=Homo sapiens OX=9606 GN=PPIA	2.027	1.6549E-05
P62987	UBA52	Ubiquitin-60S ribosomal protein L40 OS=Homo sapiens OX=9606 GN=UBA52	2.027	2.3434E-06
P63241	EIF5A	Eukaryotic translation initiation factor 5A-1 OS=Homo sapiens OX=9606 GN=EIF5A	2.023	0.00070211
P68104	EEF1A1	Elongation factor 1-alpha 1 OS=Homo sapiens OX=9606 GN=EEF1A1	2.021	1.7447E-05
P78317	RNF4	E3 ubiquitin-protein ligase RNF4 OS=Homo sapiens OX=9606 GN=RNF4	2.021	0.00074455
P78540	ARG2	"Arginase-2, mitochondrial OS=Homo sapiens OX=9606 GN=ARG2"	2.019	1.0795E-07
P78556	CCL20	C-C motif chemokine 20 OS=Homo sapiens OX=9606 GN=CCL20	2.017	0.00081888
P79522	PRR3	Proline-rich protein 3 OS=Homo sapiens OX=9606 GN=PRR3	2.016	1.9685E-05
P80303	NUCB2	Nucleobindin-2 OS=Homo sapiens OX=9606 GN=NUCB2	2.012	0.00010479
P80365	HSD11B2	Corticosteroid 11-beta-dehydrogenase	2.012	0.00046345

		isozyme 2 OS=Homo sapiens OX=9606 GN=HSD11B2		
P80404	ABAT	"4-aminobutyrate aminotransferase, mitochondrial OS=Homo sapiens OX=9606 GN=ABAT"	2.012	6.1317E-05
P83111	LACTB	"Serine beta-lactamase-like protein LACTB, mitochondrial OS=Homo sapiens OX=9606 GN=LACTB"	2.01	2.0224E-07
P83731	RPL24	60S ribosomal protein L24 OS=Homo sapiens OX=9606 GN=RPL24	2.007	0.00015948
P98196	ATP11A	Probable phospholipid-transporting ATPase IH OS=Homo sapiens OX=9606 GN=ATP11A	2.006	9.9712E-07
P99999	CYCS	Cytochrome c OS=Homo sapiens OX=9606 GN=CYCS	2.006	1.8866E-07
Q01459	CTBS	Di-N-acetylchitobiase OS=Homo sapiens OX=9606 GN=CTBS	2.006	6.9423E-07
Q01581	HMGCS1	"Hydroxymethylglutaryl-CoA synthase, cytoplasmic OS=Homo sapiens OX=9606 GN=HMGCS1"	2.001	2.3785E-05
Q02252	ALDH6A1	"Methylmalonate-semialdehyde dehydrogenase [acylating], mitochondrial OS=Homo sapiens OX=9606 GN=ALDH6A1"	0.499	0.00021513
Q02790	FKBP4	Peptidyl-prolyl cis-trans isomerase FKBP4 OS=Homo sapiens OX=9606 GN=FKBP4	0.499	0.00144131
Q02978	SLC25A11	Mitochondrial 2-oxoglutarate/malate carrier protein OS=Homo sapiens OX=9606 GN=SLC25A11	0.498	6.1643E-05
Q04637	EIF4G1	Eukaryotic translation initiation factor 4 gamma 1 OS=Homo sapiens OX=9606 GN=EIF4G1	0.498	4.4803E-05
Q04917	YWHAH	14-3-3 protein eta OS=Homo sapiens OX=9606 GN=YWHAH	0.498	1.6645E-05
Q05D60	DEUP1	Deuterosome assembly protein 1 OS=Homo sapiens OX=9606 GN=DEUP1	0.498	9.9319E-05
Q06203	PPAT	Amidophosphoribosyltransferase OS=Homo sapiens OX=9606 GN=PPAT	0.497	1.5271E-05
Q06830	PRDX1	Peroxiredoxin-1 OS=Homo sapiens OX=9606 GN=PRDX1	0.497	2.4288E-05
Q08380	LGALS3BP	Galectin-3-binding protein OS=Homo sapiens OX=9606 GN=LGALS3BP	0.497	2.8967E-06
Q08752	PPID	Peptidyl-prolyl cis-trans isomerase D OS=Homo sapiens OX=9606 GN=PPID	0.496	0.00130291
Q0P6H9	TMEM62	Transmembrane protein 62 OS=Homo sapiens OX=9606 GN=TMEM62	0.496	0.0085157
Q0ZGT2	NEXN	Nexilin OS=Homo sapiens OX=9606 GN=NEXN	0.496	0.00026285
Q12982	BNIP2	BCL2/adenovirus E1B 19 kDa protein-interacting protein 2 OS=Homo sapiens	0.496	7.7182E-07

		OX=9606 GN=BNIP2		
Q12999	TSPAN31	Tetraspanin-31 OS=Homo sapiens OX=9606 GN=TSPAN31	0.496	0.0024644
Q13085	ACACA	Acetyl-CoA carboxylase 1 OS=Homo sapiens OX=9606 GN=ACACA	0.496	3.2527E-06
Q13112	CHAF1B	Chromatin assembly factor 1 subunit B OS=Homo sapiens OX=9606 GN=CHAF1B	0.496	0.00040021
Q13133	NR1H3	Oxysterols receptor LXR-alpha OS=Homo sapiens OX=9606 GN=NR1H3	0.496	1.5042E-05
Q13232	NME3	Nucleoside diphosphate kinase 3 OS=Homo sapiens OX=9606 GN=NME3	0.496	0.00010361
Q13257	MAD2L1	Mitotic spindle assembly checkpoint protein MAD2A OS=Homo sapiens OX=9606 GN=MAD2L1	0.496	1.7419E-05
Q13418	ILK	Integrin-linked protein kinase OS=Homo sapiens OX=9606 GN=ILK	0.495	1.0191E-06
Q13451	FKBP5	Peptidyl-prolyl cis-trans isomerase FKBP5 OS=Homo sapiens OX=9606 GN=FKBP5	0.495	1.5592E-05
Q13480	GAB1	GRB2-associated-binding protein 1 OS=Homo sapiens OX=9606 GN=GAB1	0.495	1.4407E-06
Q13510	ASAH1	Acid ceramidase OS=Homo sapiens OX=9606 GN=ASAH1	0.495	8.1076E-07
Q13542	EIF4EBP2	Eukaryotic translation initiation factor 4E-binding protein 2 OS=Homo sapiens OX=9606 GN=EIF4EBP2	0.495	4.5522E-06
Q13630	TSTA3	GDP-L-fucose synthase OS=Homo sapiens OX=9606 GN=TSTA3	0.495	0.0001357
Q13740	ALCAM	CD166 antigen OS=Homo sapiens OX=9606 GN=ALCAM	0.493	6.4097E-05
Q13825	AUH	"Methylglutaconyl-CoA hydratase, mitochondrial OS=Homo sapiens OX=9606 GN=AUH"	0.493	4.156E-07
Q13951	CBFB	Core-binding factor subunit beta OS=Homo sapiens OX=9606 GN=CBFB	0.493	3.7571E-05
Q14011	CIRBP	Cold-inducible RNA-binding protein OS=Homo sapiens OX=9606 GN=CIRBP	0.493	0.00020264
Q14019	COTL1	Coactosin-like protein OS=Homo sapiens OX=9606 GN=COTL1	0.493	0.0035229
Q14032	BAAT	Bile acid-CoA:amino acid N-acyltransferase OS=Homo sapiens OX=9606 GN=BAAT	0.493	2.1104E-05
Q14254	FLOT2	Flotillin-2 OS=Homo sapiens OX=9606 GN=FLOT2	0.492	7.1652E-07
Q14353	GAMT	Guanidinoacetate N-methyltransferase OS=Homo sapiens OX=9606 GN=GAMT	0.492	0.00028042
Q14435	GALNT3	Polypeptide N-acetylgalactosaminyltransferase 3	0.492	1.3882E-06

		OS=Homo sapiens OX=9606 GN=GALNT3		
Q14493	SLBP	Histone RNA hairpin-binding protein OS=Homo sapiens OX=9606 GN=SLBP	0.491	1.7419E-06
Q14566	MCM6	DNA replication licensing factor MCM6 OS=Homo sapiens OX=9606 GN=MCM6	0.491	1.8319E-05
Q14749	GNMT	Glycine N-methyltransferase OS=Homo sapiens OX=9606 GN=GNMT	0.49	0.006639
Q15003	NCAPH	Condensin complex subunit 2 OS=Homo sapiens OX=9606 GN=NCAPH	0.489	5.696E-07
Q15004	PCLAF	PCNA-associated factor OS=Homo sapiens OX=9606 GN=PCLAF	0.489	0.00020305
Q15021	NCAPD2	Condensin complex subunit 1 OS=Homo sapiens OX=9606 GN=NCAPD2	0.489	0.00002401
Q15054	POLD3	DNA polymerase delta subunit 3 OS=Homo sapiens OX=9606 GN=POLD3	0.489	0.00091986
Q15056	EIF4H	Eukaryotic translation initiation factor 4H OS=Homo sapiens OX=9606 GN=EIF4H	0.488	1.7314E-05
Q15181	PPA1	Inorganic pyrophosphatase OS=Homo sapiens OX=9606 GN=PPA1	0.488	0.00021661
Q15185	PTGES3	Prostaglandin E synthase 3 OS=Homo sapiens OX=9606 GN=PTGES3	0.488	7.9456E-05
Q15375	EPHA7	Ephrin type-A receptor 7 OS=Homo sapiens OX=9606 GN=EPHA7	0.488	0.00064068
Q15398	DLGAP5	Disks large-associated protein 5 OS=Homo sapiens OX=9606 GN=DLGAP5	0.487	0.0054152
Q15404	RSU1	Ras suppressor protein 1 OS=Homo sapiens OX=9606 GN=RSU1	0.487	0.00015682
Q15418	RPS6KA1	Ribosomal protein S6 kinase alpha-1 OS=Homo sapiens OX=9606 GN=RPS6KA1	0.486	0.00029872
Q15431	SYCP1	Synaptonemal complex protein 1 OS=Homo sapiens OX=9606 GN=SYCP1	0.486	0.00013577
Q15642	TRIP10	Cdc42-interacting protein 4 OS=Homo sapiens OX=9606 GN=TRIP10	0.485	2.1156E-05
Q15645	TRIP13	Pachytene checkpoint protein 2 homolog OS=Homo sapiens OX=9606 GN=TRIP13	0.485	1.5564E-05
Q15646	OASL	2'-5'-oligoadenylate synthase-like protein OS=Homo sapiens OX=9606 GN=OASL	0.485	0.00142444
Q15714	TSC22D1	TSC22 domain family protein 1 OS=Homo sapiens OX=9606 GN=TSC22D1	0.484	0.0142628
Q15785	TOMM34	Mitochondrial import receptor subunit TOM34 OS=Homo sapiens OX=9606 GN=TOMM34	0.484	9.6044E-05
Q16134	ETFDH	"Electron transfer flavoprotein-ubiquinone oxidoreductase,	0.484	1.0802E-06

		mitochondrial OS=Homo sapiens OX=9606 GN=ETFDH"		
Q16222	UAP1	UDP-N-acetylhexosamine pyrophosphorylase OS=Homo sapiens OX=9606 GN=UAP1	0.484	0.00016283
Q16543	CDC37	Hsp90 co-chaperone Cdc37 OS=Homo sapiens OX=9606 GN=CDC37	0.483	0.029119
Q16625	OCLN	Occludin OS=Homo sapiens OX=9606 GN=OCLN	0.483	0.00055607
Q16718	NDUFA5	NADH dehydrogenase [ubiquinone] 1 alpha subcomplex subunit 5 OS=Homo sapiens OX=9606 GN=NDUFA5	0.482	2.9881E-07
Q16760	DGKD	Diacylglycerol kinase delta OS=Homo sapiens OX=9606 GN=DGKD	0.482	0.0001566
Q16763	UBE2S	Ubiquitin-conjugating enzyme E2 S OS=Homo sapiens OX=9606 GN=UBE2S	0.482	3.1853E-06
Q16787	LAMA3	Laminin subunit alpha-3 OS=Homo sapiens OX=9606 GN=LAMA3	0.481	9.0389E-07
Q16822	PCK2	"Phosphoenolpyruvate carboxykinase [GTP], mitochondrial OS=Homo sapiens OX=9606 GN=PCK2"	0.481	2.6079E-06
Q16836	HADH	"Hydroxyacyl-coenzyme A dehydrogenase, mitochondrial OS=Homo sapiens OX=9606 GN=HADH"	0.481	0.022382
Q2NKX8	ERCC6L	DNA excision repair protein ERCC-6-like OS=Homo sapiens OX=9606 GN=ERCC6L	0.481	9.8753E-07
Q3KRA6	C2orf76	UPF0538 protein C2orf76 OS=Homo sapiens OX=9606 GN=C2orf76	0.481	5.7997E-05
Q3KRA9	ALKBH6	Alpha-ketoglutarate-dependent dioxygenase alkB homolog 6 OS=Homo sapiens OX=9606 GN=ALKBH6	0.48	3.5511E-07
Q3MIX3	ADCK5	Uncharacterized aarF domain-containing protein kinase 5 OS=Homo sapiens OX=9606 GN=ADCK5	0.479	0.00015821
Q49AA0	ZFP69	Zinc finger protein 69 homolog OS=Homo sapiens OX=9606 GN=ZFP69	0.479	0.00002064
Q4G0N4	NADK2	"NAD kinase 2, mitochondrial OS=Homo sapiens OX=9606 GN=NADK2"	0.479	0.00013687
Q4LDG9	DNAL1	"Dynein light chain 1, axonemal OS=Homo sapiens OX=9606 GN=DNAL1"	0.478	5.5555E-05
Q53EZ4	CEP55	Centrosomal protein of 55 kDa OS=Homo sapiens OX=9606 GN=CEP55	0.478	0.0029362
Q53FT3	HIKESHI	Protein Hikeshi OS=Homo sapiens OX=9606 GN=HIKESHI	0.478	3.3363E-06
Q53R41	FASTKD1	"FAST kinase domain-containing protein 1, mitochondrial OS=Homo sapiens OX=9606 GN=FASTKD1"	0.478	6.3736E-05
Q58DX5	NAALADL2	Inactive N-acetylated-alpha-linked acidic	0.478	0.0008208

		dipeptidase-like protein 2 OS=Homo sapiens OX=9606 GN=NAALADL2		
Q5JTV8	TOR1AIP1	Torsin-1A-interacting protein 1 OS=Homo sapiens OX=9606 GN=TOR1AIP1	0.478	0.00013873
Q5SVS4	SLC25A30	Kidney mitochondrial carrier protein 1 OS=Homo sapiens OX=9606 GN=SLC25A30	0.477	0.00055708
Q5SWW7	C10orf55	Uncharacterized protein C10orf55 OS=Homo sapiens OX=9606 GN=C10orf55	0.477	1.9023E-05
Q5T2T1	MPP7	MAGUK p55 subfamily member 7 OS=Homo sapiens OX=9606 GN=MPP7	0.477	0.0023982
Q5T6F2	UBAP2	Ubiquitin-associated protein 2 OS=Homo sapiens OX=9606 GN=UBAP2	0.477	2.1104E-06
Q5T890	ERCC6L2	DNA excision repair protein ERCC-6-like 2 OS=Homo sapiens OX=9606 GN=ERCC6L2	0.476	9.5606E-07
Q5TB80	CEP162	Centrosomal protein of 162 kDa OS=Homo sapiens OX=9606 GN=CEP162	0.476	0.00010382
Q5TCQ9	MAGI3	"Membrane-associated guanylate kinase, WW and PDZ domain-containing protein 3 OS=Homo sapiens OX=9606 GN=MAGI3"	0.476	0.00015931
Q5U623	ATF7IP2	Activating transcription factor 7-interacting protein 2 OS=Homo sapiens OX=9606 GN=ATF7IP2	0.476	0.0111618
Q5XPI4	RNF123	E3 ubiquitin-protein ligase RNF123 OS=Homo sapiens OX=9606 GN=RNF123	0.476	0.0016789
Q68CQ7	GLT8D1	Glycosyltransferase 8 domain-containing protein 1 OS=Homo sapiens OX=9606 GN=GLT8D1	0.475	0.00033647
Q6DKK2	TTC19	"Tetratricopeptide repeat protein 19, mitochondrial OS=Homo sapiens OX=9606 GN=TTC19"	0.475	0.00016191
Q6FI81	CIAPIN1	Anamorsin OS=Homo sapiens OX=9606 GN=CIAPIN1	0.475	3.9548E-06
Q6IBW4	NCAPH2	Condensin-2 complex subunit H2 OS=Homo sapiens OX=9606 GN=NCAPH2	0.474	0.036397
Q6NUK1	SLC25A24	Calcium-binding mitochondrial carrier protein SCaMC-1 OS=Homo sapiens OX=9606 GN=SLC25A24	0.474	0.00010252
Q6NVY1	HIBCH	"3-hydroxyisobutyryl-CoA hydrolase, mitochondrial OS=Homo sapiens OX=9606 GN=HIBCH"	0.474	6.8055E-07
Q6NXE6	ARMC6	Armadillo repeat-containing protein 6 OS=Homo sapiens OX=9606 GN=ARMC6	0.473	6.4165E-05
Q6NYC1	JMJD6	Bifunctional arginine demethylase and	0.473	0.00029855

		lysyl-hydroxylase JMJD6 OS=Homo sapiens OX=9606 GN=JMJD6		
Q6P1A2	LPCAT3	Lysophospholipid acyltransferase 5 OS=Homo sapiens OX=9606 GN=LPCAT3	0.473	1.612E-05
Q6P1Q0	LETMD1	LETM1 domain-containing protein 1 OS=Homo sapiens OX=9606 GN=LETMD1	0.473	5.9239E-05
Q6P1Q9	METTL2B	Methyltransferase-like protein 2B OS=Homo sapiens OX=9606 GN=METTL2B	0.472	1.3938E-06
Q6PD74	AAGAB	Alpha- and gamma-adaptin-binding protein p34 OS=Homo sapiens OX=9606 GN=AAGAB	0.472	3.7942E-05
Q6PIW4	FIGNL1	Fidgetin-like protein 1 OS=Homo sapiens OX=9606 GN=FIGNL1	0.471	9.0611E-07
Q6PL18	ATAD2	ATPase family AAA domain-containing protein 2 OS=Homo sapiens OX=9606 GN=ATAD2	0.471	0.00085804
Q6SJ93	FAM111B	Protein FAM111B OS=Homo sapiens OX=9606 GN=FAM111B	0.471	0.00018494
Q6UWH4	FAM198B	Protein FAM198B OS=Homo sapiens OX=9606 GN=FAM198B	0.47	2.2249E-05
Q6UXG2	KIAA1324	UPF0577 protein KIAA1324 OS=Homo sapiens OX=9606 GN=KIAA1324	0.469	6.0829E-07
Q6UXV4	APOOL	MICOS complex subunit MIC27 OS=Homo sapiens OX=9606 GN=APOOL	0.469	3.9407E-05
Q6YN16	HSDL2	Hydroxysteroid dehydrogenase-like protein 2 OS=Homo sapiens OX=9606 GN=HSDL2	0.468	0.00113941
Q71DI3	HIST2H3A	Histone H3.2 OS=Homo sapiens OX=9606 GN=HIST2H3A	0.468	0.00091906
Q71F23	CENPU	Centromere protein U OS=Homo sapiens OX=9606 GN=CENPU	0.467	1.7613E-05
Q7L8W6	DPH6	Diphthine--ammonia ligase OS=Homo sapiens OX=9606 GN=DPH6	0.467	0.00101987
Q7Z3D6	DGLUCY	"D-glutamate cyclase, mitochondrial OS=Homo sapiens OX=9606 GN=DGLUCY"	0.467	0.0071004
Q7Z422	SZRD1	SUZ domain-containing protein 1 OS=Homo sapiens OX=9606 GN=SZRD1	0.466	0.00022209
Q7Z591	AKNA	AT-hook-containing transcription factor OS=Homo sapiens OX=9606 GN=AKNA	0.466	1.9538E-06
Q7Z6M1	RABEPK	Rab9 effector protein with kelch motifs OS=Homo sapiens OX=9606 GN=RABEPK	0.466	3.9112E-05
Q7Z7E8	UBE2Q1	Ubiquitin-conjugating enzyme E2 Q1 OS=Homo sapiens OX=9606 GN=UBE2Q1	0.465	0.00090159

Q86SK9	SCD5	Stearoyl-CoA desaturase 5 OS=Homo sapiens OX=9606 GN=SCD5	0.465	7.4922E-07
Q86UD0	SAPCD2	Suppressor APC domain-containing protein 2 OS=Homo sapiens OX=9606 GN=SAPCD2	0.465	0.00063864
Q86WR0	CCDC25	Coiled-coil domain-containing protein 25 OS=Homo sapiens OX=9606 GN=CCDC25	0.464	0.0146798
Q86XE3	MICU3	"Calcium uptake protein 3, mitochondrial OS=Homo sapiens OX=9606 GN=MICU3"	0.463	2.4909E-05
Q86Y39	NDUFA11	NADH dehydrogenase [ubiquinone] 1 alpha subcomplex subunit 11 OS=Homo sapiens OX=9606 GN=NDUFA11	0.463	6.2086E-05
Q86Y56	DNAAF5	"Dynein assembly factor 5, axonemal OS=Homo sapiens OX=9606 GN=DNAAF5"	0.463	4.1893E-05
Q86YB7	ECHDC2	"Enoyl-CoA hydratase domain-containing protein 2, mitochondrial OS=Homo sapiens OX=9606 GN=ECHDC2"	0.462	0.00085514
Q8IUE6	HIST2H2AB	Histone H2A type 2-B OS=Homo sapiens OX=9606 GN=HIST2H2AB	0.462	2.3335E-05
Q8IUR0	TRAPPC5	Trafficking protein particle complex subunit 5 OS=Homo sapiens OX=9606 GN=TRAPPC5	0.462	0.00015511
Q8IV50	LYSMD2	LysM and putative peptidoglycan-binding domain-containing protein 2 OS=Homo sapiens OX=9606 GN=LYSMD2	0.462	0.00010071
Q8IWY8	ZSCAN29	Zinc finger and SCAN domain-containing protein 29 OS=Homo sapiens OX=9606 GN=ZSCAN29	0.461	1.3253E-06
Q8IX90	SKA3	Spindle and kinetochore-associated protein 3 OS=Homo sapiens OX=9606 GN=SKA3	0.459	0.00009886
Q8IXI2	RHOT1	Mitochondrial Rho GTPase 1 OS=Homo sapiens OX=9606 GN=RHOT1	0.459	0.00038191
Q8IXQ3	C9orf40	Uncharacterized protein C9orf40 OS=Homo sapiens OX=9606 GN=C9orf40	0.458	0.00011557
Q8IY21	DDX60	Probable ATP-dependent RNA helicase DDX60 OS=Homo sapiens OX=9606 GN=DDX60	0.458	0.00047707
Q8N0X4	CLYBL	"Citramalyl-CoA lyase, mitochondrial OS=Homo sapiens OX=9606 GN=CLYBL"	0.458	0.0071362
Q8N283	ANKRD35	Ankyrin repeat domain-containing protein 35 OS=Homo sapiens OX=9606 GN=ANKRD35	0.458	0.00017692
Q8N2F6	ARMC10	Armadillo repeat-containing protein 10 OS=Homo sapiens OX=9606	0.457	0.00026016

		GN=ARMC10		
Q8N2Z9	CENPS	Centromere protein S OS=Homo sapiens OX=9606 GN=CENPS	0.457	1.6007E-06
Q8N357	SLC35F6	Solute carrier family 35 member F6 OS=Homo sapiens OX=9606 GN=SLC35F6	0.457	8.4034E-05
Q8N5I9	C12orf45	Uncharacterized protein C12orf45 OS=Homo sapiens OX=9606 GN=C12orf45	0.456	1.0177E-07
Q8N5M1	ATPAF2	ATP synthase mitochondrial F1 complex assembly factor 2 OS=Homo sapiens OX=9606 GN=ATPAF2	0.456	1.6445E-05
Q8N6N3	C1orf52	UPF0690 protein C1orf52 OS=Homo sapiens OX=9606 GN=C1orf52	0.456	0.0004763
Q8N7N1	FAM86B1	Putative protein N-methyltransferase FAM86B1 OS=Homo sapiens OX=9606 GN=FAM86B1	0.455	1.5655E-05
Q8NAN2	MIGA1	Mitoguardin 1 OS=Homo sapiens OX=9606 GN=MIGA1	0.455	1.3628E-06
Q8NBA8	DTWD2	DTW domain-containing protein 2 OS=Homo sapiens OX=9606 GN=DTWD2	0.455	0.0027572
Q8NBT2	SPC24	Kinetochore protein Spc24 OS=Homo sapiens OX=9606 GN=SPC24	0.455	0.00061568
Q8NBX0	SCCPDH	Saccharopine dehydrogenase-like oxidoreductase OS=Homo sapiens OX=9606 GN=SCCPDH	0.455	2.0295E-06
Q8ND04	SMG8	Protein SMG8 OS=Homo sapiens OX=9606 GN=SMG8	0.454	4.0231E-06
Q8NEM2	SHCBP1	SHC SH2 domain-binding protein 1 OS=Homo sapiens OX=9606 GN=SHCBP1	0.454	4.9566E-06
Q8NF91	SYNE1	Nesprin-1 OS=Homo sapiens OX=9606 GN=SYNE1	0.453	0.0001016
Q8NFW9	MYRIP	Rab effector MyRIP OS=Homo sapiens OX=9606 GN=MYRIP	0.453	3.7335E-05
Q8TB22	SPATA20	Spermatogenesis-associated protein 20 OS=Homo sapiens OX=9606 GN=SPATA20	0.452	3.7953E-07
Q8TBB5	KLHDC4	Kelch domain-containing protein 4 OS=Homo sapiens OX=9606 GN=KLHDC4	0.452	3.5673E-06
Q8TBC5	ZSCAN18	Zinc finger and SCAN domain-containing protein 18 OS=Homo sapiens OX=9606 GN=ZSCAN18	0.452	0.0048187
Q8TBE9	NANP	N-acylneuraminate-9-phosphatase OS=Homo sapiens OX=9606 GN=NANP	0.452	7.8089E-05
Q8TBP5	FAM174A	Membrane protein FAM174A OS=Homo sapiens OX=9606 GN=FAM174A	0.452	1.2691E-06
Q8TCA0	LRRC20	Leucine-rich repeat-containing protein 20 OS=Homo sapiens OX=9606 GN=LRRC20	0.451	1.5651E-07

Q8TCG1	CIP2A	Protein CIP2A OS=Homo sapiens OX=9606 GN=CIP2A	0.451	0.0028838
Q8TCY9	URGCP	Up-regulator of cell proliferation OS=Homo sapiens OX=9606 GN=URGCP	0.451	1.1271E-06
Q8TD22	SFXN5	Sideroflexin-5 OS=Homo sapiens OX=9606 GN=SFXN5	0.45	0.03966
Q8TDR0	TRAF3IP1	TRAF3-interacting protein 1 OS=Homo sapiens OX=9606 GN=TRAF3IP1	0.45	0.00013608
Q8TEW0	PARD3	Partitioning defective 3 homolog OS=Homo sapiens OX=9606 GN=PARD3	0.45	0.00059932
Q8WUX2	CHAC2	Glutathione-specific gamma-glutamylcyclotransferase 2 OS=Homo sapiens OX=9606 GN=CHAC2	0.449	0.00160213
Q8WVJ2	NUDCD2	NudC domain-containing protein 2 OS=Homo sapiens OX=9606 GN=NUDCD2	0.448	1.7792E-05
Q8WWK9	CKAP2	Cytoskeleton-associated protein 2 OS=Homo sapiens OX=9606 GN=CKAP2	0.448	1.7358E-05
Q8WX93	PALLD	Palladin OS=Homo sapiens OX=9606 GN=PALLD	0.447	7.5005E-05
Q8WXG1	RSAD2	Radical S-adenosyl methionine domain-containing protein 2 OS=Homo sapiens OX=9606 GN=RSAD2	0.447	1.5576E-05
Q8WXX5	DNAJC9	DnaJ homolog subfamily C member 9 OS=Homo sapiens OX=9606 GN=DNAJC9	0.447	0.0018018
Q8WZA0	LZIC	Protein LZIC OS=Homo sapiens OX=9606 GN=LZIC	0.446	0.00025672
Q92508	PIEZO1	Piezo-type mechanosensitive ion channel component 1 OS=Homo sapiens OX=9606 GN=PIEZO1	0.446	1.6806E-05
Q92598	HSPH1	Heat shock protein 105 kDa OS=Homo sapiens OX=9606 GN=HSPH1	0.445	4.0643E-05
Q92664	GTF3A	Transcription factor IIIA OS=Homo sapiens OX=9606 GN=GTF3A	0.445	0.006181
Q92835	INPP5D	"Phosphatidylinositol 3,4,5-trisphosphate 5-phosphatase 1 OS=Homo sapiens OX=9606 GN=INPP5D"	0.445	2.0573E-05
Q92990	GLMN	Glomulin OS=Homo sapiens OX=9606 GN=GLMN	0.444	2.3095E-05
Q93045	STMN2	Stathmin-2 OS=Homo sapiens OX=9606 GN=STMN2	0.444	3.0976E-06
Q969Q0	RPL36AL	60S ribosomal protein L36a-like OS=Homo sapiens OX=9606 GN=RPL36AL	0.444	7.8233E-05
Q969U7	PSMG2	Proteasome assembly chaperone 2 OS=Homo sapiens OX=9606 GN=PSMG2	0.442	1.7296E-05
Q96A26	FAM162A	Protein FAM162A OS=Homo sapiens	0.441	6.543E-07

		OX=9606 GN=FAM162A		
Q96A73	KIAA1191	Putative monooxygenase p33MONOX OS=Homo sapiens OX=9606 GN=KIAA1191	0.441	0.0025967
Q96BJ3	AIDA	"Axin interactor, dorsalization-associated protein OS=Homo sapiens OX=9606 GN=AIDA"	0.441	0.00058292
Q96BN8	OTULIN	Ubiquitin thioesterase otulin OS=Homo sapiens OX=9606 GN=OTULIN	0.44	1.722E-05
Q96BR1	SGK3	Serine/threonine-protein kinase Sgk3 OS=Homo sapiens OX=9606 GN=SGK3	0.44	1.6406E-06
Q96C90	PPP1R14B	Protein phosphatase 1 regulatory subunit 14B OS=Homo sapiens OX=9606 GN=PPP1R14B	0.44	4.2336E-06
Q96CM8	ACSF2	"Acyl-CoA synthetase family member 2, mitochondrial OS=Homo sapiens OX=9606 GN=ACSF2"	0.44	4.6561E-06
Q96DA2	RAB39B	Ras-related protein Rab-39B OS=Homo sapiens OX=9606 GN=RAB39B	0.44	3.4861E-06
Q96DG6	CMBL	Carboxymethylenebutenolidase homolog OS=Homo sapiens OX=9606 GN=CMBL	0.44	0.0048836
Q96EB1	ELP4	Elongator complex protein 4 OS=Homo sapiens OX=9606 GN=ELP4	0.44	2.9649E-06
Q96EK6	GNPNAT1	Glucosamine 6-phosphate N-acetyltransferase OS=Homo sapiens OX=9606 GN=GNPNAT1	0.439	1.7349E-05
Q96FE7	PIK3IP1	Phosphoinositide-3-kinase-interacting protein 1 OS=Homo sapiens OX=9606 GN=PIK3IP1	0.439	0.00197887
Q96GA3	LTV1	Protein LTV1 homolog OS=Homo sapiens OX=9606 GN=LTV1	0.438	0.00086281
Q96GX5	MASTL	Serine/threonine-protein kinase greatwall OS=Homo sapiens OX=9606 GN=MASTL	0.438	2.0708E-05
Q96HA9	PEX11G	Peroxisomal membrane protein 11C OS=Homo sapiens OX=9606 GN=PEX11G	0.437	3.9735E-05
Q96HJ9	FMC1	Protein FMC1 homolog OS=Homo sapiens OX=9606 GN=FMC1	0.437	2.2839E-06
Q96I99	SUCLG2	"Succinate--CoA ligase [GDP-forming] subunit beta, mitochondrial OS=Homo sapiens OX=9606 GN=SUCLG2"	0.436	0.00040231
Q96KB5	PBK	Lymphokine-activated killer T-cell-originated protein kinase OS=Homo sapiens OX=9606 GN=PBK	0.435	2.8174E-06
Q96LT4	SAMD8	Sphingomyelin synthase-related protein 1 OS=Homo sapiens OX=9606 GN=SAMD8	0.435	1.8687E-05
Q96P70	IPO9	Importin-9 OS=Homo sapiens OX=9606 GN=IPO9	0.435	9.4214E-07
Q96PE7	MCEE	"Methylmalonyl-CoA epimerase,	0.435	0.00089712

		mitochondrial OS=Homo sapiens OX=9606 GN=MCEE"		
Q96PP9	GBP4	Guanylate-binding protein 4 OS=Homo sapiens OX=9606 GN=GBP4	0.433	1.8836E-05
Q96QB1	DLC1	Rho GTPase-activating protein 7 OS=Homo sapiens OX=9606 GN=DLC1	0.433	0.00012308
Q96R06	SPAG5	Sperm-associated antigen 5 OS=Homo sapiens OX=9606 GN=SPAG5	0.432	3.2679E-06
Q96RR4	CAMKK2	Calcium/calmodulin-dependent protein kinase kinase 2 OS=Homo sapiens OX=9606 GN=CAMKK2	0.432	2.9328E-07
Q96RT7	TUBGCP6	Gamma-tubulin complex component 6 OS=Homo sapiens OX=9606 GN=TUBGCP6	0.432	2.3551E-06
Q96S16	JMJD8	JmjC domain-containing protein 8 OS=Homo sapiens OX=9606 GN=JMJD8	0.432	1.6079E-07
Q96SB4	SRPK1	SRSF protein kinase 1 OS=Homo sapiens OX=9606 GN=SRPK1	0.432	2.3987E-05
Q96T88	UHRF1	E3 ubiquitin-protein ligase UHRF1 OS=Homo sapiens OX=9606 GN=UHRF1	0.431	1.3082E-08
Q99471	PFDN5	Prefoldin subunit 5 OS=Homo sapiens OX=9606 GN=PFDN5	0.431	0.00033633
Q99487	PAFAH2	"Platelet-activating factor acetylhydrolase 2, cytoplasmic OS=Homo sapiens OX=9606 GN=PAFAH2"	0.43	8.2164E-05
Q99543	DNAJC2	DnaJ homolog subfamily C member 2 OS=Homo sapiens OX=9606 GN=DNAJC2	0.429	3.9967E-05
Q99615	DNAJC7	DnaJ homolog subfamily C member 7 OS=Homo sapiens OX=9606 GN=DNAJC7	0.429	1.8576E-06
Q99618	CDCA3	Cell division cycle-associated protein 3 OS=Homo sapiens OX=9606 GN=CDCA3	0.428	1.6286E-06
Q99622	C12orf57	Protein C10 OS=Homo sapiens OX=9606 GN=C12orf57	0.428	6.1448E-05
Q99640	PKMYT1	Membrane-associated tyrosine- and threonine-specific cdc2-inhibitory kinase OS=Homo sapiens OX=9606 GN=PKMYT1	0.428	3.9767E-05
Q99661	KIF2C	Kinesin-like protein KIF2C OS=Homo sapiens OX=9606 GN=KIF2C	0.427	0.0022399
Q99733	NAP1L4	Nucleosome assembly protein 1-like 4 OS=Homo sapiens OX=9606 GN=NAP1L4	0.427	0.00010076
Q99798	ACO2	"Aconitate hydratase, mitochondrial OS=Homo sapiens OX=9606 GN=ACO2"	0.427	0.00041669
Q99985	SEMA3C	Semaphorin-3C OS=Homo sapiens	0.427	2.1766E-05

		OX=9606 GN=SEMA3C		
Q9BPX3	NCAPG	Condensin complex subunit 3 OS=Homo sapiens OX=9606 GN=NCAPG	0.426	0.00044169
Q9BPZ3	PAIP2	Polyadenylate-binding protein-interacting protein 2 OS=Homo sapiens OX=9606 GN=PAIP2	0.425	0.0058002
Q9BQ69	MACROD1	O-acetyl-ADP-ribose deacetylase MACROD1 OS=Homo sapiens OX=9606 GN=MACROD1	0.424	0.00020328
Q9BQI0	AIF1L	Allograft inflammatory factor 1-like OS=Homo sapiens OX=9606 GN=AIF1L	0.424	3.7055E-05
Q9BQL6	FERMT1	Fermitin family homolog 1 OS=Homo sapiens OX=9606 GN=FERMT1	0.423	0.00026499
Q9BRP1	PDCD2L	Programmed cell death protein 2-like OS=Homo sapiens OX=9606 GN=PDCD2L	0.423	1.6014E-05
Q9BRT9	GINS4	DNA replication complex GINS protein SLD5 OS=Homo sapiens OX=9606 GN=GINS4	0.422	1.8271E-05
Q9BRX5	GINS3	DNA replication complex GINS protein PSF3 OS=Homo sapiens OX=9606 GN=GINS3	0.421	1.7641E-05
Q9BTE3	MCMBP	Mini-chromosome maintenance complex-binding protein OS=Homo sapiens OX=9606 GN=MCMBP	0.42	1.5208E-05
Q9BU89	DOHH	Deoxyhypusine hydroxylase OS=Homo sapiens OX=9606 GN=DOHH	0.42	0.00040189
Q9BUT9	MCRIP2	MAPK regulated corepressor interacting protein 2 OS=Homo sapiens OX=9606 GN=MCRIP2	0.42	0.00044384
Q9BV44	THUMPD3	THUMP domain-containing protein 3 OS=Homo sapiens OX=9606 GN=THUMPD3	0.418	0.00034422
Q9BV57	ADI1	"1,2-dihydroxy-3-keto-5-methylthiopentene dioxygenase OS=Homo sapiens OX=9606 GN=ADI1"	0.417	4.1208E-06
Q9BV79	MECR	"Enoyl-[acyl-carrier-protein] reductase, mitochondrial OS=Homo sapiens OX=9606 GN=MECR"	0.416	4.6875E-06
Q9BV86	NTMT1	N-terminal Xaa-Pro-Lys N-methyltransferase 1 OS=Homo sapiens OX=9606 GN=NTMT1	0.416	0.0005199
Q9BVS4	RIOK2	Serine/threonine-protein kinase RIO2 OS=Homo sapiens OX=9606 GN=RIOK2	0.415	4.0964E-05
Q9BVW5	TIPIN	TIMELESS-interacting protein OS=Homo sapiens OX=9606 GN=TIPIN	0.414	4.7813E-06
Q9BW04	SARG	Specifically androgen-regulated gene protein OS=Homo sapiens OX=9606 GN=SARG	0.414	2.5933E-07
Q9BW71	HIRIP3	HIRA-interacting protein 3 OS=Homo sapiens OX=9606 GN=HIRIP3	0.413	0.00020438

Q9BW83	IFT27	Intraflagellar transport protein 27 homolog OS=Homo sapiens OX=9606 GN=IFT27	0.413	9.9505E-07
Q9BWT3	PAPOLG	Poly(A) polymerase gamma OS=Homo sapiens OX=9606 GN=PAPOLG	0.413	1.7963E-05
Q9BX59	TAPBPL	Tapasin-related protein OS=Homo sapiens OX=9606 GN=TAPBPL	0.413	5.5567E-07
Q9BX68	HINT2	"Histidine triad nucleotide-binding protein 2, mitochondrial OS=Homo sapiens OX=9606 GN=HINT2"	0.412	3.5149E-05
Q9BXJ9	NAA15	"N-alpha-acetyltransferase 15, NatA auxiliary subunit OS=Homo sapiens OX=9606 GN=NAA15"	0.411	2.6858E-07
Q9BXS6	NUSAP1	Nucleolar and spindle-associated protein 1 OS=Homo sapiens OX=9606 GN=NUSAP1	0.41	2.8897E-07
Q9BXW9	FANCD2	Fanconi anemia group D2 protein OS=Homo sapiens OX=9606 GN=FANCD2	0.41	3.7256E-05
Q9BY49	PECR	Peroxisomal trans-2-enoyl-CoA reductase OS=Homo sapiens OX=9606 GN=PECR	0.409	1.3844E-06
Q9BYB4	GNB1L	Guanine nucleotide-binding protein subunit beta-like protein 1 OS=Homo sapiens OX=9606 GN=GNB1L	0.408	1.3375E-06
Q9BZD4	NUF2	Kinetochore protein Nuf2 OS=Homo sapiens OX=9606 GN=NUF2	0.408	2.4501E-05
Q9BZG8	DPH1	2-(3-amino-3-carboxypropyl)histidine synthase subunit 1 OS=Homo sapiens OX=9606 GN=DPH1	0.408	0.00077708
Q9BZQ2	SHCBP1L	Testicular spindle-associated protein SHCBP1L OS=Homo sapiens OX=9606 GN=SHCBP1L	0.406	0.00009983
Q9BZX2	UCK2	Uridine-cytidine kinase 2 OS=Homo sapiens OX=9606 GN=UCK2	0.402	0.00028114
Q9C004	SPRY4	Protein sprouty homolog 4 OS=Homo sapiens OX=9606 GN=SPRY4	0.402	1.8548E-05
Q9C0D2	CEP295	Centrosomal protein of 295 kDa OS=Homo sapiens OX=9606 GN=CEP295	0.401	8.4116E-07
Q9C0F1	CEP44	Centrosomal protein of 44 kDa OS=Homo sapiens OX=9606 GN=CEP44	0.4	2.5973E-06
Q9GZN7	ROGDI	Protein rogdi homolog OS=Homo sapiens OX=9606 GN=ROGDI	0.4	1.3093E-06
Q9GZT6	CCDC90B	"Coiled-coil domain-containing protein 90B, mitochondrial OS=Homo sapiens OX=9606 GN=CCDC90B"	0.4	3.9903E-05
Q9GZZ1	NAA50	N-alpha-acetyltransferase 50 OS=Homo sapiens OX=9606 GN=NAA50	0.4	3.8805E-06
Q9H2J4	PDCL3	Phosducin-like protein 3 OS=Homo sapiens OX=9606 GN=PDCL3	0.399	2.2923E-05

Q9H2P9	DPH5	Diphthine methyl ester synthase OS=Homo sapiens OX=9606 GN=DPH5	0.399	2.2778E-06
Q9H2U2	PPA2	"Inorganic pyrophosphatase 2, mitochondrial OS=Homo sapiens OX=9606 GN=PPA2"	0.399	0.00147737
Q9H322	VCX2	Variable charge X-linked protein 2 OS=Homo sapiens OX=9606 GN=VCX2	0.397	9.8302E-05
Q9H446	RWDD1	RWD domain-containing protein 1 OS=Homo sapiens OX=9606 GN=RWDD1	0.395	0.00127769
Q9H467	CUEDC2	CUE domain-containing protein 2 OS=Homo sapiens OX=9606 GN=CUEDC2	0.395	1.9491E-06
Q9H4H8	FAM83D	Protein FAM83D OS=Homo sapiens OX=9606 GN=FAM83D	0.394	0.00034165
Q9H788	SH2D4A	SH2 domain-containing protein 4A OS=Homo sapiens OX=9606 GN=SH2D4A	0.393	0.0001606
Q9H7L9	SUDS3	Sin3 histone deacetylase corepressor complex component SDS3 OS=Homo sapiens OX=9606 GN=SUDS3	0.392	0.00016437
Q9H900	ZWILCH	Protein zwilch homolog OS=Homo sapiens OX=9606 GN=ZWILCH	0.39	4.7622E-06
Q9H910	JPT2	Jupiter microtubule associated homolog 2 OS=Homo sapiens OX=9606 GN=JPT2	0.388	3.3422E-06
Q9H999	PANK3	Pantothenate kinase 3 OS=Homo sapiens OX=9606 GN=PANK3	0.388	1.2635E-07
Q9H9S4	CAB39L	Calcium-binding protein 39-like OS=Homo sapiens OX=9606 GN=CAB39L	0.387	6.3384E-05
Q9HAN9	NMNAT1	Nicotinamide/nicotinic acid mononucleotide adenylyltransferase 1 OS=Homo sapiens OX=9606 GN=NMNAT1	0.385	5.5631E-05
Q9HAT2	SIAE	Sialate O-acetylesterase OS=Homo sapiens OX=9606 GN=SIAE	0.385	0.0008194
Q9HB71	CACYBP	Calcyclin-binding protein OS=Homo sapiens OX=9606 GN=CACYBP	0.384	2.2352E-07
Q9HBA9	FOLH1B	Putative N-acetylated-alpha-linked acidic dipeptidase OS=Homo sapiens OX=9606 GN=FOLH1B	0.381	7.6857E-05
Q9HBI1	PARVB	Beta-parvin OS=Homo sapiens OX=9606 GN=PARVB	0.381	0.0030645
Q9HBL7	PLGRKT	Plasminogen receptor (KT) OS=Homo sapiens OX=9606 GN=PLGRKT	0.379	2.0955E-05
Q9HC78	ZBTB20	Zinc finger and BTB domain-containing protein 20 OS=Homo sapiens OX=9606 GN=ZBTB20	0.376	0.0024395
Q9HCI5	MAGEE1	Melanoma-associated antigen E1 OS=Homo sapiens OX=9606 GN=MAGEE1	0.375	0.0021627
Q9HD42	CHMP1A	Charged multivesicular body protein 1a	0.375	0.00034441

		OS=Homo sapiens OX=9606 GN=CHMP1A		
Q9NP74	PALMD	Palmdelphin OS=Homo sapiens OX=9606 GN=PALMD	0.373	1.5571E-05
Q9NPA3	MID1IP1	Mid1-interacting protein 1 OS=Homo sapiens OX=9606 GN=MID1IP1	0.373	7.7222E-05
Q9NPD8	UBE2T	Ubiquitin-conjugating enzyme E2 T OS=Homo sapiens OX=9606 GN=UBE2T	0.373	0.000597
Q9NQP4	PFDN4	Prefoldin subunit 4 OS=Homo sapiens OX=9606 GN=PFDN4	0.372	4.1446E-07
Q9NQW6	ANLN	Anillin OS=Homo sapiens OX=9606 GN=ANLN	0.371	9.1794E-07
Q9NR19	ACSS2	"Acetyl-coenzyme A synthetase, cytoplasmic OS=Homo sapiens OX=9606 GN=ACSS2"	0.37	5.9346E-05
Q9NR33	POLE4	DNA polymerase epsilon subunit 4 OS=Homo sapiens OX=9606 GN=POLE4	0.37	0.00010399
Q9NRN7	AASDHPPT	L-aminoadipate-semialdehyde dehydrogenase-phosphopantetheinyl transferase OS=Homo sapiens OX=9606 GN=AASDHPPT	0.369	1.9814E-05
Q9NS91	RAD18	E3 ubiquitin-protein ligase RAD18 OS=Homo sapiens OX=9606 GN=RAD18	0.369	5.179E-07
Q9NSA3	CTNNBIP1	Beta-catenin-interacting protein 1 OS=Homo sapiens OX=9606 GN=CTNNBIP1	0.368	0.00195879
Q9NT62	ATG3	Ubiquitin-like-conjugating enzyme ATG3 OS=Homo sapiens OX=9606 GN=ATG3	0.367	1.1332E-06
Q9NTG7	SIRT3	"NAD-dependent protein deacetylase sirtuin-3, mitochondrial OS=Homo sapiens OX=9606 GN=SIRT3"	0.367	0.0024786
Q9NTJ3	SMC4	Structural maintenance of chromosomes protein 4 OS=Homo sapiens OX=9606 GN=SMC4	0.365	3.6822E-05
Q9NTK5	OLA1	Obg-like ATPase 1 OS=Homo sapiens OX=9606 GN=OLA1	0.365	0.0003758
Q9NUJ1	ABHD10	"Mycophenolic acid acyl-glucuronide esterase, mitochondrial OS=Homo sapiens OX=9606 GN=ABHD10"	0.364	2.1904E-06
Q9NUM3	SLC39A9	Zinc transporter ZIP9 OS=Homo sapiens OX=9606 GN=SLC39A9	0.362	8.1714E-08
Q9NUQ8	ABCF3	ATP-binding cassette sub-family F member 3 OS=Homo sapiens OX=9606 GN=ABCF3	0.362	0.00069783
Q9NUT2	ABCB8	"ATP-binding cassette sub-family B member 8, mitochondrial OS=Homo sapiens OX=9606 GN=ABCB8"	0.36	3.0624E-06
Q9NVA1	UQCC1	Ubiquinol-cytochrome-c reductase	0.36	2.4094E-06

		complex assembly factor 1 OS=Homo sapiens OX=9606 GN=UQCC1		
Q9NVQ4	FAIM	Fas apoptotic inhibitory molecule 1 OS=Homo sapiens OX=9606 GN=FAIM	0.36	2.1381E-07
Q9NVR0	KLHL11	Kelch-like protein 11 OS=Homo sapiens OX=9606 GN=KLHL11	0.36	0.0025405
Q9NVR5	DNAAF2	Protein kintoun OS=Homo sapiens OX=9606 GN=DNAAF2	0.359	0.00022412
Q9NW75	GPATCH2	G patch domain-containing protein 2 OS=Homo sapiens OX=9606 GN=GPATCH2	0.359	2.9406E-08
Q9NX55	HYPK	Huntingtin-interacting protein K OS=Homo sapiens OX=9606 GN=HYPK	0.357	0.0001249
Q9NXF8	ZDHHC7	Palmitoyltransferase ZDHHC7 OS=Homo sapiens OX=9606 GN=ZDHHC7	0.357	1.9326E-06
Q9NY33	DPP3	Dipeptidyl peptidase 3 OS=Homo sapiens OX=9606 GN=DPP3	0.356	0.00051908
Q9NYJ8	TAB2	TGF-beta-activated kinase 1 and MAP3K7-binding protein 2 OS=Homo sapiens OX=9606 GN=TAB2	0.355	2.9863E-07
Q9NZJ0	DTL	Denticleless protein homolog OS=Homo sapiens OX=9606 GN=DTL	0.354	4.1923E-06
Q9NZJ7	MTCH1	Mitochondrial carrier homolog 1 OS=Homo sapiens OX=9606 GN=MTCH1	0.35	1.534E-07
Q9NZJ9	NUDT4	Diphosphoinositol polyphosphate phosphohydrolase 2 OS=Homo sapiens OX=9606 GN=NUDT4	0.35	0.00016202
Q9P032	NDUFAF4	NADH dehydrogenase [ubiquinone] 1 alpha subcomplex assembly factor 4 OS=Homo sapiens OX=9606 GN=NDUFAF4	0.349	5.6471E-05
Q9P0J0	NDUFA13	NADH dehydrogenase [ubiquinone] 1 alpha subcomplex subunit 13 OS=Homo sapiens OX=9606 GN=NDUFA13	0.347	0.00030025
Q9P0P8	C6orf203	Uncharacterized protein C6orf203 OS=Homo sapiens OX=9606 GN=C6orf203	0.34	1.5084E-05
Q9P0V9	SEPT10	Septin-10 OS=Homo sapiens OX=9606 GN=SEPT10	0.34	1.538E-05
Q9P1A6	DLGAP2	Disks large-associated protein 2 OS=Homo sapiens OX=9606 GN=DLGAP2	0.339	7.3334E-09
Q9P246	STIM2	Stromal interaction molecule 2 OS=Homo sapiens OX=9606 GN=STIM2	0.339	5.7311E-05
Q9P2K5	MYEF2	Myelin expression factor 2 OS=Homo sapiens OX=9606 GN=MYEF2	0.338	0.00031921
Q9P2P6	STARD9	StAR-related lipid transfer protein 9 OS=Homo sapiens OX=9606 GN=STARD9	0.337	4.4506E-05

Q9UBB4	ATXN10	Ataxin-10 OS=Homo sapiens OX=9606 GN=ATXN10	0.336	0.00019768
Q9UBK9	UXT	Protein UXT OS=Homo sapiens OX=9606 GN=UXT	0.334	2.2875E-05
Q9UDR5	AASS	"Alpha-aminoadipic semialdehyde synthase, mitochondrial OS=Homo sapiens OX=9606 GN=AASS"	0.332	0.0036847
Q9UDY8	MALT1	Mucosa-associated lymphoid tissue lymphoma translocation protein 1 OS=Homo sapiens OX=9606 GN=MALT1	0.331	5.1438E-07
Q9UEW8	STK39	STE20/SPS1-related proline-alanine-rich protein kinase OS=Homo sapiens OX=9606 GN=STK39	0.331	1.5643E-05
Q9UEY8	ADD3	Gamma-adducin OS=Homo sapiens OX=9606 GN=ADD3	0.33	3.8175E-05
Q9UFN0	NIPSNAP3A	Protein NipSnap homolog 3A OS=Homo sapiens OX=9606 GN=NIPSNAP3A	0.329	1.0768E-07
Q9UHD1	CHORDC1	Cysteine and histidine-rich domain-containing protein 1 OS=Homo sapiens OX=9606 GN=CHORDC1	0.325	2.2324E-06
Q9UHL4	DPP7	Dipeptidyl peptidase 2 OS=Homo sapiens OX=9606 GN=DPP7	0.322	4.6472E-06
Q9UHV9	PFDN2	Prefoldin subunit 2 OS=Homo sapiens OX=9606 GN=PFDN2	0.322	0.00053962
Q9UHW5	GPN3	GPN-loop GTPase 3 OS=Homo sapiens OX=9606 GN=GPN3	0.321	5.8378E-05
Q9UI26	IPO11	Importin-11 OS=Homo sapiens OX=9606 GN=IPO11	0.318	3.5319E-06
Q9UII2	ATP5IF1	"ATPase inhibitor, mitochondrial OS=Homo sapiens OX=9606 GN=ATP5IF1"	0.316	4.1909E-05
Q9UII4	HERC5	E3 ISG15--protein ligase HERC5 OS=Homo sapiens OX=9606 GN=HERC5	0.315	1.7215E-05
Q9UIJ7	AK3	"GTP:AMP phosphotransferase AK3, mitochondrial OS=Homo sapiens OX=9606 GN=AK3"	0.314	0.0097211
Q9UJ14	GGT7	Glutathione hydrolase 7 OS=Homo sapiens OX=9606 GN=GGT7	0.314	0.00071921
Q9UJ83	HACL1	2-hydroxyacyl-CoA lyase 1 OS=Homo sapiens OX=9606 GN=HACL1	0.314	2.2223E-06
Q9UK76	JPT1	Jupiter microtubule associated homolog 1 OS=Homo sapiens OX=9606 GN=JPT1	0.312	2.0796E-05
Q9UKG9	CROT	Peroxisomal carnitine O-octanoyltransferase OS=Homo sapiens OX=9606 GN=CROT	0.31	8.3924E-07
Q9UKT5	FBXO4	F-box only protein 4 OS=Homo sapiens OX=9606 GN=FBXO4	0.308	2.0756E-05
Q9UKU7	ACAD8	"Isobutyryl-CoA dehydrogenase, mitochondrial OS=Homo sapiens OX=9606 GN=ACAD8"	0.305	0.039961

Q9UKY7	CDV3	Protein CDV3 homolog OS=Homo sapiens OX=9606 GN=CDV3	0.304	2.6477E-07
Q9UL16	CFAP45	Cilia- and flagella-associated protein 45 OS=Homo sapiens OX=9606 GN=CFAP45	0.303	0.0135972
Q9ULC4	MCTS1	Malignant T-cell-amplified sequence 1 OS=Homo sapiens OX=9606 GN=MCTS1	0.303	0.00012351
Q9ULD0	OGDHL	"2-oxoglutarate dehydrogenase-like, mitochondrial OS=Homo sapiens OX=9606 GN=OGDHL"	0.303	5.8965E-07
Q9ULM6	CNOT6	CCR4-NOT transcription complex subunit 6 OS=Homo sapiens OX=9606 GN=CNOT6	0.301	0.0049555
Q9ULS5	TMCC3	Transmembrane and coiled-coil domain protein 3 OS=Homo sapiens OX=9606 GN=TMCC3	0.299	3.729E-06
Q9ULW0	TPX2	Targeting protein for Xklp2 OS=Homo sapiens OX=9606 GN=TPX2	0.299	3.7376E-05
Q9ULX3	NOB1	RNA-binding protein NOB1 OS=Homo sapiens OX=9606 GN=NOB1	0.299	0.0026605
Q9UM54	MYO6	Unconventional myosin-VI OS=Homo sapiens OX=9606 GN=MYO6	0.297	0.00043505
Q9UNI6	DUSP12	Dual specificity protein phosphatase 12 OS=Homo sapiens OX=9606 GN=DUSP12	0.295	7.9787E-08
Q9UNS1	TIMELESS	Protein timeless homolog OS=Homo sapiens OX=9606 GN=TIMELESS	0.293	1.5991E-05
Q9UNY4	TTF2	Transcription termination factor 2 OS=Homo sapiens OX=9606 GN=TTF2	0.292	3.4947E-07
Q9UPS8	ANKRD26	Ankyrin repeat domain-containing protein 26 OS=Homo sapiens OX=9606 GN=ANKRD26	0.289	0.00015827
Q9UQ80	PA2G4	Proliferation-associated protein 2G4 OS=Homo sapiens OX=9606 GN=PA2G4	0.282	8.3285E-05
Q9UQN3	CHMP2B	Charged multivesicular body protein 2b OS=Homo sapiens OX=9606 GN=CHMP2B	0.281	6.0109E-07
Q9Y248	GINS2	DNA replication complex GINS protein PSF2 OS=Homo sapiens OX=9606 GN=GINS2	0.28	1.8128E-05
Q9Y266	NUDC	Nuclear migration protein nudC OS=Homo sapiens OX=9606 GN=NUDC	0.273	0.0100396
Q9Y2Q3	GSTK1	Glutathione S-transferase kappa 1 OS=Homo sapiens OX=9606 GN=GSTK1	0.266	0.0042977
Q9Y2Y0	ARL2BP	ADP-ribosylation factor-like protein 2-binding protein OS=Homo sapiens OX=9606 GN=ARL2BP	0.263	1.2763E-08
Q9Y2Y1	POLR3K	DNA-directed RNA polymerase III	0.263	5.9502E-05

		subunit RPC10 OS=Homo sapiens OX=9606 GN=POLR3K		
Q9Y316	MEMO1	Protein MEMO1 OS=Homo sapiens OX=9606 GN=MEMO1	0.259	0.00002106
Q9Y3D8	AK6	Adenylate kinase isoenzyme 6 OS=Homo sapiens OX=9606 GN=AK6	0.255	1.4254E-07
Q9Y3F4	STRAP	Serine-threonine kinase receptor-associated protein OS=Homo sapiens OX=9606 GN=STRAP	0.254	2.4076E-05
Q9Y448	KNSTRN	Small kinetochore-associated protein OS=Homo sapiens OX=9606 GN=KNSTRN	0.254	0.00075698
Q9Y4F9	RIPOR2	Rho family-interacting cell polarization regulator 2 OS=Homo sapiens OX=9606 GN=RIPOR2	0.253	3.6965E-05
Q9Y4K4	MAP4K5	Mitogen-activated protein kinase kinase kinase kinase 5 OS=Homo sapiens OX=9606 GN=MAP4K5	0.25	7.3126E-07
Q9Y4P1	ATG4B	Cysteine protease ATG4B OS=Homo sapiens OX=9606 GN=ATG4B	0.247	0.0020606
Q9Y512	SAMM50	Sorting and assembly machinery component 50 homolog OS=Homo sapiens OX=9606 GN=SAMM50	0.244	0.0067759
Q9Y5F3	PCDHB1	Protocadherin beta-1 OS=Homo sapiens OX=9606 GN=PCDHB1	0.233	2.1914E-05
Q9Y5P4	COL4A3BP	Collagen type IV alpha-3-binding protein OS=Homo sapiens OX=9606 GN=COL4A3BP	0.228	3.7431E-05
Q9Y5U8	MPC1	Mitochondrial pyruvate carrier 1 OS=Homo sapiens OX=9606 GN=MPC1	0.215	1.733E-05
Q9Y639	NPTN	Neuroplastin OS=Homo sapiens OX=9606 GN=NPTN	0.21	2.4777E-05
Q9Y646	CPQ	Carboxypeptidase Q OS=Homo sapiens OX=9606 GN=CPQ	0.196	1.9757E-05
Q9Y6A5	TACC3	Transforming acidic coiled-coil-containing protein 3 OS=Homo sapiens OX=9606 GN=TACC3	0.194	0.000357
Q9Y6E2	BZW2	Basic leucine zipper and W2 domain-containing protein 2 OS=Homo sapiens OX=9606 GN=BZW2	0.191	9.5391E-05
Q9Y6M9	NDUFB9	NADH dehydrogenase [ubiquinone] 1 beta subcomplex subunit 9 OS=Homo sapiens OX=9606 GN=NDUFB9	0.173	1.8218E-06
Q9Y6N5	SQOR	"Sulfide:quinone oxidoreductase, mitochondrial OS=Homo sapiens OX=9606 GN=SQOR"	0.146	1.3851E-06

Table S2 Differentially expressed proteins in LNCaP enzDTP cells

Table S3. Differentially expressed proteins in 22Rv1 epiDTP cells (>2 fold, p<0.05)

Accession	Gene	Protein description	Ratio	P value

O15050	TRANK1	TPR and ankyrin repeat-containing protein 1 OS=Homo sapiens OX=9606 GN=TRANK1	20.617	0.00021961
O75969	AKAP3	A-kinase anchor protein 3 OS=Homo sapiens OX=9606 GN=AKAP3	15.018	0.00015813
Q6ZUS6	CCDC149	Coiled-coil domain-containing protein 149 OS=Homo sapiens OX=9606 GN=CCDC149	13.685	9.9252E-05
P08138	NGFR	Tumor necrosis factor receptor superfamily member 16 OS=Homo sapiens OX=9606 GN=NGFR	12.305	5.7457E-05
Q8WV93	AFG1L	AFG1-like ATPase OS=Homo sapiens OX=9606 GN=AFG1L	11.462	0.00040056
Q5MAI5	CDKL4	Cyclin-dependent kinase-like 4 OS=Homo sapiens OX=9606 GN=CDKL4	10.749	0.002985
Q53GL7	PARP10	Poly [ADP-ribose] polymerase 10 OS=Homo sapiens OX=9606 GN=PARP10	9.991	0.00028289
A6NCL1	GMNC	Geminin coiled-coil domain-containing protein 1 OS=Homo sapiens OX=9606 GN=GMNC	9.887	2.0098E-05
Q92835	INPP5D	"Phosphatidylinositol 3,4,5-trisphosphate 5-phosphatase 1 OS=Homo sapiens OX=9606 GN=INPP5D"	9.809	1.7915E-05
P10632	CYP2C8	Cytochrome P450 2C8 OS=Homo sapiens OX=9606 GN=CYP2C8	9.493	2.1409E-06
P0CG24	ZNF883	Zinc finger protein 883 OS=Homo sapiens OX=9606 GN=ZNF883	9.382	0.00089988
Q8TDM6	DLG5	Disks large homolog 5 OS=Homo sapiens OX=9606 GN=DLG5	8.681	0.00054
Q8NHH1	TTLL11	Tubulin polyglutamylase TTLL11 OS=Homo sapiens OX=9606 GN=TTLL11	8.513	0.0002214
Q8IW40	CCDC103	Coiled-coil domain-containing protein 103 OS=Homo sapiens OX=9606 GN=CCDC103	8.102	0.00197954
Q14520	HABP2	Hyaluronan-binding protein 2 OS=Homo sapiens OX=9606 GN=HABP2	7.739	0.0030367
Q9ULW5	RAB26	Ras-related protein Rab-26 OS=Homo sapiens OX=9606 GN=RAB26	7.515	1.9788E-05
Q93075	TATDN2	Putative deoxyribonuclease TATDN2 OS=Homo sapiens OX=9606 GN=TATDN2	7.461	0.0008049
Q5TZF3	ANKRD45	Ankyrin repeat domain-containing protein 45 OS=Homo sapiens OX=9606 GN=ANKRD45	7.418	0.00077833
O95445	APOM	Apolipoprotein M OS=Homo sapiens OX=9606 GN=APOM	7.308	6.4155E-05
P55822	SH3BGR	SH3 domain-binding glutamic acid-rich protein OS=Homo sapiens OX=9606 GN=SH3BGR	7.264	0.00004139
Q8IWY8	ZSCAN29	Zinc finger and SCAN domain-containing protein 29 OS=Homo sapiens OX=9606 GN=ZSCAN29	6.933	0.0027445
Q9GZY6	LAT2	Linker for activation of T-cells family member 2 OS=Homo sapiens OX=9606	6.924	2.1672E-05

		GN=LAT2		
P04733	MT1F	Metallothionein-1F OS=Homo sapiens OX=9606 GN=MT1F	6.864	3.3624E-06
Q9P219	CCDC88C	Protein Daple OS=Homo sapiens OX=9606 GN=CCDC88C	6.82	0.00133932
Q8TF64	GIPC3	PDZ domain-containing protein GIPC3 OS=Homo sapiens OX=9606 GN=GIPC3	6.801	0.00027743
P08910	ABHD2	Monoacylglycerol lipase ABHD2 OS=Homo sapiens OX=9606 GN=ABHD2	6.633	4.0742E-06
P80297	MT1X	Metallothionein-1X OS=Homo sapiens OX=9606 GN=MT1X	6.526	0.00041803
Q9H3T3	SEMA6B	Semaphorin-6B OS=Homo sapiens OX=9606 GN=SEMA6B	6.478	0.0075167
Q86X45	LRRC6	Protein tilB homolog OS=Homo sapiens OX=9606 GN=LRRC6	6.465	0.0005449
Q9NPJ4	PNRC2	Proline-rich nuclear receptor coactivator 2 OS=Homo sapiens OX=9606 GN=PNRC2	6.456	0.00178194
Q16787	LAMA3	Laminin subunit alpha-3 OS=Homo sapiens OX=9606 GN=LAMA3	6.423	0.00107993
P38570	ITGAE	Integrin alpha-E OS=Homo sapiens OX=9606 GN=ITGAE	6.396	0.00054245
Q5SRE7	PHYHD1	Phytanoyl-CoA dioxygenase domain-containing protein 1 OS=Homo sapiens OX=9606 GN=PHYHD1	6.351	0.00090119
Q8IW19	APLF	Aprataxin and PNK-like factor OS=Homo sapiens OX=9606 GN=APLF	5.987	9.7458E-05
Q96P20	NLRP3	"NACHT, LRR and PYD domains-containing protein 3 OS=Homo sapiens OX=9606 GN=NLRP3"	5.979	0.00172035
Q7Z5U6	WDR53	WD repeat-containing protein 53 OS=Homo sapiens OX=9606 GN=WDR53	5.962	0.00093924
P11055	MYH3	Myosin-3 OS=Homo sapiens OX=9606 GN=MYH3	5.95	0.00010385
Q9NQ35	NRIP3	Nuclear receptor-interacting protein 3 OS=Homo sapiens OX=9606 GN=NRIP3	5.735	0.00184011
P25815	S100P	Protein S100-P OS=Homo sapiens OX=9606 GN=S100P	5.647	0.00080328
P58499	FAM3B	Protein FAM3B OS=Homo sapiens OX=9606 GN=FAM3B	5.623	8.0692E-05
Q9BVV6	KIAA0586	Protein TALPID3 OS=Homo sapiens OX=9606 GN=KIAA0586	5.607	0.00181969
Q9NYU1	UGGT2	UDP-glucose:glycoprotein glucosyltransferase 2 OS=Homo sapiens OX=9606 GN=UGGT2	5.593	0.00081758
C9JSJ3	BHMG1	Basic helix-loop-helix and HMG box domain-containing protein 1 OS=Homo sapiens OX=9606 GN=BHMG1	5.57	0.0082754
Q6IEE8	SLFN12L	Schlafen family member 12-like OS=Homo sapiens OX=9606 GN=SLFN12L	5.467	0.0011043
Q9HCF6	TRPM3	Transient receptor potential cation channel subfamily M member 3 OS=Homo sapiens OX=9606 GN=TRPM3	5.377	0.00068493

Q9Y6Q1	CAPN6	Calpain-6 OS=Homo sapiens OX=9606 GN=CAPN6	5.321	0.00402
O60262	GNG7	Guanine nucleotide-binding protein G(I)/G(S)/G(O) subunit gamma-7 OS=Homo sapiens OX=9606 GN=GNG7	5.173	0.00046448
P17936	IGFBP3	Insulin-like growth factor-binding protein 3 OS=Homo sapiens OX=9606 GN=IGFBP3	5.099	0.00011526
O60292	SIPA1L3	Signal-induced proliferation-associated 1-like protein 3 OS=Homo sapiens OX=9606 GN=SIPA1L3	5.083	0.00012441
Q13233	MAP3K1	Mitogen-activated protein kinase kinase kinase 1 OS=Homo sapiens OX=9606 GN=MAP3K1	5.048	1.4445E-08
Q86YW9	MED12L	Mediator of RNA polymerase II transcription subunit 12-like protein OS=Homo sapiens OX=9606 GN=MED12L	5.028	0.00072402
O14763	TNFRSF10B	Tumor necrosis factor receptor superfamily member 10B OS=Homo sapiens OX=9606 GN=TNFRSF10B	5.007	1.5678E-05
Q9BZQ8	FAM129A	Protein Niban OS=Homo sapiens OX=9606 GN=FAM129A	4.955	1.892E-05
Q5S007	LRRK2	Leucine-rich repeat serine/threonine-protein kinase 2 OS=Homo sapiens OX=9606 GN=LRRK2	4.909	0.00012255
Q96MR9	ZNF560	Zinc finger protein 560 OS=Homo sapiens OX=9606 GN=ZNF560	4.898	0.00018457
P54253	ATXN1	Ataxin-1 OS=Homo sapiens OX=9606 GN=ATXN1	4.855	0.015202
Q13009	TIAM1	T-lymphoma invasion and metastasis-inducing protein 1 OS=Homo sapiens OX=9606 GN=TIAM1	4.772	0.0088364
Q99988	GDF15	Growth/differentiation factor 15 OS=Homo sapiens OX=9606 GN=GDF15	4.641	1.2234E-06
Q9HCI5	MAGEE1	Melanoma-associated antigen E1 OS=Homo sapiens OX=9606 GN=MAGEE1	4.613	0.001361
Q9P1A6	DLGAP2	Disks large-associated protein 2 OS=Homo sapiens OX=9606 GN=DLGAP2	4.604	0.032943
Q6ZN28	MACC1	Metastasis-associated in colon cancer protein 1 OS=Homo sapiens OX=9606 GN=MACC1	4.593	3.0375E-06
Q9Y6V0	PCLO	Protein piccolo OS=Homo sapiens OX=9606 GN=PCLO	4.556	0.0002757
O00622	CYR61	Protein CYR61 OS=Homo sapiens OX=9606 GN=CYR61	4.545	0.00064265
Q2KHT4	GSG1	Germ cell-specific gene 1 protein OS=Homo sapiens OX=9606 GN=GSG1	4.493	0.00011855
P17676	CEBPB	CCAAT/enhancer-binding protein beta OS=Homo sapiens OX=9606 GN=CEBPB	4.437	0.00126236
Q8IWZ5	TRIM42	Tripartite motif-containing protein 42 OS=Homo sapiens OX=9606 GN=TRIM42	4.424	0.0021963
Q9HBT6	CDH20	Cadherin-20 OS=Homo sapiens OX=9606 GN=CDH20	4.424	0.0034166

P10635	CYP2D6	Cytochrome P450 2D6 OS=Homo sapiens OX=9606 GN=CYP2D6	4.399	2.4202E-05
Q8TF21	ANKRD24	Ankyrin repeat domain-containing protein 24 OS=Homo sapiens OX=9606 GN=ANKRD24	4.351	0.024299
Q9NU19	TBC1D22B	TBC1 domain family member 22B OS=Homo sapiens OX=9606 GN=TBC1D22B	4.2	1.7644E-06
Q96QU1	PCDH15	Protocadherin-15 OS=Homo sapiens OX=9606 GN=PCDH15	4.133	0.0021806
A4FU49	SH3D21	SH3 domain-containing protein 21 OS=Homo sapiens OX=9606 GN=SH3D21	4.074	0.00224
Q6DHV5	CC2D2B	Protein CC2D2B OS=Homo sapiens OX=9606 GN=CC2D2B	3.977	1.9397E-05
Q8IYK2	CCDC105	Coiled-coil domain-containing protein 105 OS=Homo sapiens OX=9606 GN=CCDC105	3.936	6.6388E-07
Q9GZM7	TINAGL1	Tubulointerstitial nephritis antigen-like OS=Homo sapiens OX=9606 GN=TINAGL1	3.926	0.00015548
Q5SRD1	TIMM23B	Putative mitochondrial import inner membrane translocase subunit Tim23B OS=Homo sapiens OX=9606 GN=TIMM23B	3.841	0.0003607
P56524	HDAC4	Histone deacetylase 4 OS=Homo sapiens OX=9606 GN=HDAC4	3.83	0.00014191
O14967	CLGN	Calmegin OS=Homo sapiens OX=9606 GN=CLGN	3.826	1.4753E-06
Q16822	PCK2	"Phosphoenolpyruvate carboxykinase [GTP], mitochondrial OS=Homo sapiens OX=9606 GN=PCK2"	3.75	4.0385E-05
Q9NXT0	ZNF586	Zinc finger protein 586 OS=Homo sapiens OX=9606 GN=ZNF586	3.743	2.0933E-05
Q9ULE3	DENND2A	DENN domain-containing protein 2A OS=Homo sapiens OX=9606 GN=DENND2A	3.715	0.00181614
P18847	ATF3	Cyclic AMP-dependent transcription factor ATF-3 OS=Homo sapiens OX=9606 GN=ATF3	3.669	3.6638E-05
O95271	TNKS	Tankyrase-1 OS=Homo sapiens OX=9606 GN=TNKS	3.666	0.0003775
O60503	ADCY9	Adenylate cyclase type 9 OS=Homo sapiens OX=9606 GN=ADCY9	3.626	0.00059841
Q9BXU7	USP26	Ubiquitin carboxyl-terminal hydrolase 26 OS=Homo sapiens OX=9606 GN=USP26	3.597	0.00097741
Q9UM63	PLAGL1	Zinc finger protein PLAGL1 OS=Homo sapiens OX=9606 GN=PLAGL1	3.582	0.040417
Q9HB03	ELOVL3	Elongation of very long chain fatty acids protein 3 OS=Homo sapiens OX=9606 GN=ELOVL3	3.565	1.1389E-06
P53667	LIMK1	LIM domain kinase 1 OS=Homo sapiens OX=9606 GN=LIMK1	3.563	1.5739E-06

O43374	RASA4	Ras GTPase-activating protein 4 OS=Homo sapiens OX=9606 GN=RASA4	3.561	0.0062819
Q969I3	GLYATL1	Glycine N-acyltransferase-like protein 1 OS=Homo sapiens OX=9606 GN=GLYATL1	3.558	0.020179
O15484	CAPN5	Calpain-5 OS=Homo sapiens OX=9606 GN=CAPN5	3.5	4.2771E-05
Q9H322	VCX2	Variable charge X-linked protein 2 OS=Homo sapiens OX=9606 GN=VCX2	3.466	0.0021412
Q3MIT2	PUS10	Putative tRNA pseudouridine synthase Pus10 OS=Homo sapiens OX=9606 GN=PUS10	3.447	0.0004982
Q8IVF4	DNAH10	"Dynein heavy chain 10, axonemal OS=Homo sapiens OX=9606 GN=DNAH10"	3.374	0.0045606
Q9BZ71	PITPNM3	Membrane-associated phosphatidylinositol transfer protein 3 OS=Homo sapiens OX=9606 GN=PITPNM3	3.361	0.0019385
Q8IYW2	CFAP46	Cilia- and flagella-associated protein 46 OS=Homo sapiens OX=9606 GN=CFAP46	3.307	3.5356E-05
Q8WVV4	POF1B	Protein POF1B OS=Homo sapiens OX=9606 GN=POF1B	3.293	3.8609E-05
O75638	CTAG2	Cancer/testis antigen 2 OS=Homo sapiens OX=9606 GN=CTAG2	3.29	0.00088419
A6NFE2	SMCO2	Single-pass membrane and coiled-coil domain-containing protein 2 OS=Homo sapiens OX=9606 GN=SMCO2	3.283	6.4828E-05
P19971	TYMP	Thymidine phosphorylase OS=Homo sapiens OX=9606 GN=TYMP	3.273	3.5905E-05
Q8NDH3	NPEPL1	Probable aminopeptidase NPEPL1 OS=Homo sapiens OX=9606 GN=NPEPL1	3.253	0.0211
Q8TCN5	ZNF507	Zinc finger protein 507 OS=Homo sapiens OX=9606 GN=ZNF507	3.227	0.00086076
Q9BXL7	CARD11	Caspase recruitment domain-containing protein 11 OS=Homo sapiens OX=9606 GN=CARD11	3.213	3.7221E-05
Q92563	SPOCK2	Testican-2 OS=Homo sapiens OX=9606 GN=SPOCK2	3.206	0.00118473
Q9UMF0	ICAM5	Intercellular adhesion molecule 5 OS=Homo sapiens OX=9606 GN=ICAM5	3.195	0.00072269
Q9UPY5	SLC7A11	Cystine/glutamate transporter OS=Homo sapiens OX=9606 GN=SLC7A11	3.189	0.00042124
P0C7M7	ACSM4	"Acyl-coenzyme A synthetase ACSM4, mitochondrial OS=Homo sapiens OX=9606 GN=ACSM4"	3.182	0.00095575
Q9UHD4	CIDEB	Cell death activator CIDE-B OS=Homo sapiens OX=9606 GN=CIDEB	3.171	0.0026986
P49747	COMP	Cartilage oligomeric matrix protein OS=Homo sapiens OX=9606 GN=COMP	3.17	0.00023647
P16422	EPCAM	Epithelial cell adhesion molecule OS=Homo sapiens OX=9606 GN=EPCAM	3.147	2.2889E-05
P08631	HCK	Tyrosine-protein kinase HCK OS=Homo	3.141	0.00025727

		sapiens OX=9606 GN=HCK		
P55809	OXCT1	"Succinyl-CoA:3-ketoacid coenzyme A transferase 1, mitochondrial OS=Homo sapiens OX=9606 GN=OXCT1"	3.136	1.8958E-05
Q8N126	CADM3	Cell adhesion molecule 3 OS=Homo sapiens OX=9606 GN=CADM3	3.129	0.00040424
Q96GA7	SDSL	Serine dehydratase-like OS=Homo sapiens OX=9606 GN=SDSL	3.115	0.00042344
P05090	APOD	Apolipoprotein D OS=Homo sapiens OX=9606 GN=APOD	3.11	1.6489E-06
Q96LJ7	DHRS1	Dehydrogenase/reductase SDR family member 1 OS=Homo sapiens OX=9606 GN=DHRS1	3.101	3.7546E-05
P43007	SLC1A4	Neutral amino acid transporter A OS=Homo sapiens OX=9606 GN=SLC1A4	3.093	0.0028006
P55317	FOXA1	Hepatocyte nuclear factor 3-alpha OS=Homo sapiens OX=9606 GN=FOXA1	3.044	0.00081861
Q6ZTQ3	RASSF6	Ras association domain-containing protein 6 OS=Homo sapiens OX=9606 GN=RASSF6	3.03	5.9566E-05
Q9ULD2	MTUS1	Microtubule-associated tumor suppressor 1 OS=Homo sapiens OX=9606 GN=MTUS1	3	0.00163767
Q9UGT4	SUSD2	Sushi domain-containing protein 2 OS=Homo sapiens OX=9606 GN=SUSD2	2.999	0.0021769
Q9UBL6	CPNE7	Copine-7 OS=Homo sapiens OX=9606 GN=CPNE7	2.981	0.00031894
Q9UGL1	KDM5B	Lysine-specific demethylase 5B OS=Homo sapiens OX=9606 GN=KDM5B	2.977	8.0366E-05
Q8TD43	TRPM4	Transient receptor potential cation channel subfamily M member 4 OS=Homo sapiens OX=9606 GN=TRPM4	2.95	1.5428E-07
Q9UKW4	VAV3	Guanine nucleotide exchange factor VAV3 OS=Homo sapiens OX=9606 GN=VAV3	2.937	0.0030236
Q562E7	WDR81	WD repeat-containing protein 81 OS=Homo sapiens OX=9606 GN=WDR81	2.919	4.2067E-05
Q9HC78	ZBTB20	Zinc finger and BTB domain-containing protein 20 OS=Homo sapiens OX=9606 GN=ZBTB20	2.917	4.9017E-07
Q5BJE1	CCDC178	Coiled-coil domain-containing protein 178 OS=Homo sapiens OX=9606 GN=CCDC178	2.883	0.00154316
Q3SY69	ALDH1L2	Mitochondrial 10-formyltetrahydrofolate dehydrogenase OS=Homo sapiens OX=9606 GN=ALDH1L2	2.869	4.0568E-08
Q13740	ALCAM	CD166 antigen OS=Homo sapiens OX=9606 GN=ALCAM	2.861	1.8334E-05
Q96K75	ZNF514	Zinc finger protein 514 OS=Homo sapiens OX=9606 GN=ZNF514	2.859	0.00026132
Q5VTL7	FNDC7	Fibronectin type III domain-containing protein 7 OS=Homo sapiens OX=9606 GN=FNDC7	2.842	0.0041448
Q9Y4C4	MFHAS1	Malignant fibrous histiocytoma-amplified sequence 1 OS=Homo sapiens OX=9606	2.84	0.00115956

		GN=MFHAS1		
Q14114	LRP8	Low-density lipoprotein receptor-related protein 8 OS=Homo sapiens OX=9606 GN=LRP8	2.831	0.00013808
Q9H3R2	MUC13	Mucin-13 OS=Homo sapiens OX=9606 GN=MUC13	2.813	1.9347E-05
P51178	PLCD1	"1-phosphatidylinositol 4,5-bisphosphate phosphodiesterase delta-1 OS=Homo sapiens OX=9606 GN=PLCD1"	2.803	4.0237E-05
Q8N3R3	TCAIM	"T-cell activation inhibitor, mitochondrial OS=Homo sapiens OX=9606 GN=TCAIM"	2.792	0.00038381
P51648	ALDH3A2	Fatty aldehyde dehydrogenase OS=Homo sapiens OX=9606 GN=ALDH3A2	2.783	6.2629E-05
Q5VVW2	GARNL3	GTPase-activating Rap/Ran-GAP domain-like protein 3 OS=Homo sapiens OX=9606 GN=GARNL3	2.781	0.0041581
Q9UPA5	BSN	Protein bassoon OS=Homo sapiens OX=9606 GN=BSN	2.776	0.00034334
Q86YB7	ECHDC2	"Enoyl-CoA hydratase domain-containing protein 2, mitochondrial OS=Homo sapiens OX=9606 GN=ECHDC2"	2.771	0.00024121
Q9H0T7	RAB17	Ras-related protein Rab-17 OS=Homo sapiens OX=9606 GN=RAB17	2.742	0.00072391
Q9UQ49	NEU3	Sialidase-3 OS=Homo sapiens OX=9606 GN=NEU3	2.728	0.00020256
Q14435	GALNT3	Polypeptide N-acetylgalactosaminyltransferase 3 OS=Homo sapiens OX=9606 GN=GALNT3	2.71	0.00014217
P03923	MT-ND6	NADH-ubiquinone oxidoreductase chain 6 OS=Homo sapiens OX=9606 GN=MT-ND6	2.703	0.00109902
P08174	CD55	Complement decay-accelerating factor OS=Homo sapiens OX=9606 GN=CD55	2.7	3.5855E-05
Q9BX66	SORBS1	Sorbin and SH3 domain-containing protein 1 OS=Homo sapiens OX=9606 GN=SORBS1	2.697	1.5632E-05
P49748	ACADVL	"Very long-chain specific acyl-CoA dehydrogenase, mitochondrial OS=Homo sapiens OX=9606 GN=ACADVL"	2.685	9.1342E-08
P46527	CDKN1B	Cyclin-dependent kinase inhibitor 1B OS=Homo sapiens OX=9606 GN=CDKN1B	2.679	7.8824E-05
P42226	STAT6	Signal transducer and activator of transcription 6 OS=Homo sapiens OX=9606 GN=STAT6	2.678	0.02098
Q9UPT6	MAPK8IP3	C-Jun-amino-terminal kinase-interacting protein 3 OS=Homo sapiens OX=9606 GN=MAPK8IP3	2.666	9.5112E-05
Q3KQV9	UAP1L1	UDP-N-acetylhexosamine pyrophosphorylase-like protein 1 OS=Homo sapiens OX=9606 GN=UAP1L1	2.663	0.00017737
Q8NFT2	STEAP2	Metalloreductase STEAP2 OS=Homo sapiens OX=9606 GN=STEAP2	2.659	0.0100019
O43502	RAD51C	DNA repair protein RAD51 homolog 3 OS=Homo sapiens OX=9606 GN=RAD51C	2.658	2.6287E-06

Q6P1A2	LPCAT3	Lysophospholipid acyltransferase 5 OS=Homo sapiens OX=9606 GN=LPCAT3	2.648	0.0029647
P00488	F13A1	Coagulation factor XIII A chain OS=Homo sapiens OX=9606 GN=F13A1	2.646	0.00086301
P0DMV2	CT45A9	Cancer/testis antigen family 45 member A9 OS=Homo sapiens OX=9606 GN=CT45A9	2.634	1.6572E-05
O14841	OPLAH	5-oxoprolinase OS=Homo sapiens OX=9606 GN=OPLAH	2.633	4.4092E-05
O43570	CA12	Carbonic anhydrase 12 OS=Homo sapiens OX=9606 GN=CA12	2.632	0.0001981
O00458	IFRD1	Interferon-related developmental regulator 1 OS=Homo sapiens OX=9606 GN=IFRD1	2.626	0.0040416
Q9Y2M0	FAN1	Fanconi-associated nuclease 1 OS=Homo sapiens OX=9606 GN=FAN1	2.617	0.0032824
P36776	LONP1	"Lon protease homolog, mitochondrial OS=Homo sapiens OX=9606 GN=LONP1"	2.616	1.9711E-05
Q6NXG1	ESRP1	Epithelial splicing regulatory protein 1 OS=Homo sapiens OX=9606 GN=ESRP1	2.616	4.1555E-05
Q9UI32	GLS2	"Glutaminase liver isoform, mitochondrial OS=Homo sapiens OX=9606 GN=GLS2"	2.612	0.0007175
Q9P2P6	STARD9	StAR-related lipid transfer protein 9 OS=Homo sapiens OX=9606 GN=STARD9	2.611	0.0146789
O95359	TACC2	Transforming acidic coiled-coil-containing protein 2 OS=Homo sapiens OX=9606 GN=TACC2	2.61	0.0030356
Q7Z404	TMC4	Transmembrane channel-like protein 4 OS=Homo sapiens OX=9606 GN=TMC4	2.573	3.5437E-05
P08575	PTPRC	Receptor-type tyrosine-protein phosphatase C OS=Homo sapiens OX=9606 GN=PTPRC	2.533	7.9158E-05
Q8IZ52	CHPF	Chondroitin sulfate synthase 2 OS=Homo sapiens OX=9606 GN=CHPF	2.532	0.00044466
P51790	CLCN3	H(+)/Cl(-) exchange transporter 3 OS=Homo sapiens OX=9606 GN=CLCN3	2.53	0.00186062
Q8N4S9	MARVELD2	MARVEL domain-containing protein 2 OS=Homo sapiens OX=9606 GN=MARVELD2	2.53	0.00087787
Q6KCM7	SLC25A25	Calcium-binding mitochondrial carrier protein SCaMC-2 OS=Homo sapiens OX=9606 GN=SLC25A25	2.523	5.8855E-05
P19634	SLC9A1	Sodium/hydrogen exchanger 1 OS=Homo sapiens OX=9606 GN=SLC9A1	2.515	0.00025878
Q86VU5	COMTD1	Catechol O-methyltransferase domain-containing protein 1 OS=Homo sapiens OX=9606 GN=COMTD1	2.506	4.0928E-06
O60568	PLOD3	"Procollagen-lysine,2-oxoglutarate 5-dioxygenase 3 OS=Homo sapiens OX=9606 GN=PLOD3"	2.497	1.4295E-06
Q5CZA5	ZNF805	Zinc finger protein 805 OS=Homo sapiens OX=9606 GN=ZNF805	2.492	0.0052369
P04424	ASL	Argininosuccinate lyase OS=Homo sapiens OX=9606 GN=ASL	2.488	2.2957E-07
Q9NP80	PNPLA8	Calcium-independent phospholipase A2-	2.488	0.00035617

		gamma OS=Homo sapiens OX=9606 GN=PNPLA8		
P78358	CTAG1A	Cancer/testis antigen 1 OS=Homo sapiens OX=9606 GN=CTAG1A	2.484	0.00048347
Q9BY49	PECR	Peroxisomal trans-2-enoyl-CoA reductase OS=Homo sapiens OX=9606 GN=PECR	2.474	0.00019634
Q5RI15	COX20	"Cytochrome c oxidase assembly protein COX20, mitochondrial OS=Homo sapiens OX=9606 GN=COX20"	2.473	0.00033644
Q9H300	PARL	"Presenilins-associated rhomboid-like protein, mitochondrial OS=Homo sapiens OX=9606 GN=PARL"	2.473	2.0454E-05
Q9Y6M5	SLC30A1	Zinc transporter 1 OS=Homo sapiens OX=9606 GN=SLC30A1	2.473	0.00027537
Q58FG1	HSP90AA4P	Putative heat shock protein HSP 90-alpha A4 OS=Homo sapiens OX=9606 GN=HSP90AA4P	2.471	0.0048242
Q53GD3	SLC44A4	Choline transporter-like protein 4 OS=Homo sapiens OX=9606 GN=SLC44A4	2.465	4.3927E-05
Q58DX5	NAALADL2	Inactive N-acetylated-alpha-linked acidic dipeptidase-like protein 2 OS=Homo sapiens OX=9606 GN=NAALADL2	2.465	0.00177798
P21741	MDK	Midkine OS=Homo sapiens OX=9606 GN=MDK	2.46	6.1929E-07
P33121	ACSL1	Long-chain-fatty-acid--CoA ligase 1 OS=Homo sapiens OX=9606 GN=ACSL1	2.46	2.9798E-07
Q8N490	PNKD	Probable hydrolase PNKD OS=Homo sapiens OX=9606 GN=PNKD	2.457	0.00166353
Q3MIN7	RGL3	Ral guanine nucleotide dissociation stimulator-like 3 OS=Homo sapiens OX=9606 GN=RGL3	2.448	0.00083954
Q9P2J8	ZNF624	Zinc finger protein 624 OS=Homo sapiens OX=9606 GN=ZNF624	2.445	0.0102846
Q16623	STX1A	Syntaxin-1A OS=Homo sapiens OX=9606 GN=STX1A	2.433	0.00003739
O00142	TK2	"Thymidine kinase 2, mitochondrial OS=Homo sapiens OX=9606 GN=TK2"	2.429	2.3181E-05
O75635	SERPINB7	Serpin B7 OS=Homo sapiens OX=9606 GN=SERPINB7	2.429	0.0029952
Q8N8U2	CDYL2	Chromodomain Y-like protein 2 OS=Homo sapiens OX=9606 GN=CDYL2	2.419	6.3591E-05
Q5TGZ0	MINOS1	MICOS complex subunit MIC10 OS=Homo sapiens OX=9606 GN=MINOS1	2.409	0.0074161
Q9H0V1	TMEM168	Transmembrane protein 168 OS=Homo sapiens OX=9606 GN=TMEM168	2.394	0.0020435
P07919	UQCRH	"Cytochrome b-c1 complex subunit 6, mitochondrial OS=Homo sapiens OX=9606 GN=UQCRH"	2.393	0.0006783
Q96MH6	TMEM68	Transmembrane protein 68 OS=Homo sapiens OX=9606 GN=TMEM68	2.393	3.6271E-06
Q96CF2	CHMP4C	Charged multivesicular body protein 4c OS=Homo sapiens OX=9606 GN=CHMP4C	2.39	0.035161

Q8NBN3	TMEM87A	Transmembrane protein 87A OS=Homo sapiens OX=9606 GN=TMEM87A	2.387	7.8384E-05
Q9Y2H5	PLEKHA6	Pleckstrin homology domain-containing family A member 6 OS=Homo sapiens OX=9606 GN=PLEKHA6	2.385	4.3594E-05
Q96RT7	TUBGCP6	Gamma-tubulin complex component 6 OS=Homo sapiens OX=9606 GN=TUBGCP6	2.383	0.0124615
Q8IWT6	LRRC8A	Volume-regulated anion channel subunit LRRC8A OS=Homo sapiens OX=9606 GN=LRRC8A	2.378	0.00014477
Q8TB22	SPATA20	Spermatogenesis-associated protein 20 OS=Homo sapiens OX=9606 GN=SPATA20	2.376	4.5804E-07
Q330K2	NDUFAF6	"NADH dehydrogenase (ubiquinone) complex I, assembly factor 6 OS=Homo sapiens OX=9606 GN=NDUFAF6"	2.369	0.00029798
Q9NQE9	HINT3	Histidine triad nucleotide-binding protein 3 OS=Homo sapiens OX=9606 GN=HINT3	2.368	1.1022E-06
Q8TEK3	DOT1L	"Histone-lysine N-methyltransferase, H3 lysine-79 specific OS=Homo sapiens OX=9606 GN=DOT1L"	2.367	0.0115239
P30711	GSTT1	Glutathione S-transferase theta-1 OS=Homo sapiens OX=9606 GN=GSTT1	2.358	0.0043969
O15321	TM9SF1	Transmembrane 9 superfamily member 1 OS=Homo sapiens OX=9606 GN=TM9SF1	2.354	0.0042584
Q14376	GALE	UDP-glucose 4-epimerase OS=Homo sapiens OX=9606 GN=GALE	2.354	2.1508E-05
Q96HD1	CRELD1	Cysteine-rich with EGF-like domain protein 1 OS=Homo sapiens OX=9606 GN=CRELD1	2.354	0.00012142
Q8NFU3	TSTD1	Thiosulfate:glutathione sulfurtransferase OS=Homo sapiens OX=9606 GN=TSTD1	2.352	0.0049025
Q12913	PTPRJ	Receptor-type tyrosine-protein phosphatase eta OS=Homo sapiens OX=9606 GN=PTPRJ	2.351	0.00025509
Q02252	ALDH6A1	"Methylmalonate-semialdehyde dehydrogenase [acylating], mitochondrial OS=Homo sapiens OX=9606 GN=ALDH6A1"	2.347	3.6735E-06
Q9GZR1	SENP6	Sentrin-specific protease 6 OS=Homo sapiens OX=9606 GN=SENP6	2.347	0.020043
P00156	MT-CYB	Cytochrome b OS=Homo sapiens OX=9606 GN=MT-CYB	2.345	0.00014049
P34897	SHMT2	"Serine hydroxymethyltransferase, mitochondrial OS=Homo sapiens OX=9606 GN=SHMT2"	2.333	6.8354E-07
Q8TAA5	GRPEL2	"GrpE protein homolog 2, mitochondrial OS=Homo sapiens OX=9606 GN=GRPEL2"	2.322	3.9281E-05
P21397	MAOA	Amine oxidase [flavin-containing] A OS=Homo sapiens OX=9606 GN=MAOA	2.318	0.00010334

Q8NCG7	DAGLB	Sn1-specific diacylglycerol lipase beta OS=Homo sapiens OX=9606 GN=DAGLB	2.312	0.0081228
Q16610	ECM1	Extracellular matrix protein 1 OS=Homo sapiens OX=9606 GN=ECM1	2.31	0.00033719
Q6ZSB9	ZBTB49	Zinc finger and BTB domain-containing protein 49 OS=Homo sapiens OX=9606 GN=ZBTB49	2.31	0.00070395
Q02447	SP3	Transcription factor Sp3 OS=Homo sapiens OX=9606 GN=SP3	2.308	0.0047181
Q9BRS8	LARP6	La-related protein 6 OS=Homo sapiens OX=9606 GN=LARP6	2.301	4.3437E-05
Q9NUI1	DECR2	"Peroxisomal 2,4-dienoyl-CoA reductase OS=Homo sapiens OX=9606 GN=DECR2"	2.298	1.8714E-05
O43189	PHF1	PHD finger protein 1 OS=Homo sapiens OX=9606 GN=PHF1	2.295	0.0006358
Q9UPQ0	LIMCH1	LIM and calponin homology domains-containing protein 1 OS=Homo sapiens OX=9606 GN=LIMCH1	2.292	6.3894E-07
P13637	ATP1A3	Sodium/potassium-transporting ATPase subunit alpha-3 OS=Homo sapiens OX=9606 GN=ATP1A3	2.291	0.00032124
Q8ND25	ZNRF1	E3 ubiquitin-protein ligase ZNRF1 OS=Homo sapiens OX=9606 GN=ZNRF1	2.291	0.0183385
Q15771	RAB30	Ras-related protein Rab-30 OS=Homo sapiens OX=9606 GN=RAB30	2.283	7.9715E-05
Q92504	SLC39A7	Zinc transporter SLC39A7 OS=Homo sapiens OX=9606 GN=SLC39A7	2.283	0.0036166
Q86YS6	RAB43	Ras-related protein Rab-43 OS=Homo sapiens OX=9606 GN=RAB43	2.282	0.00015786
P04040	CAT	Catalase OS=Homo sapiens OX=9606 GN=CAT	2.275	1.8585E-06
Q15560	TCEA2	Transcription elongation factor A protein 2 OS=Homo sapiens OX=9606 GN=TCEA2	2.275	0.028239
Q04609	FOLH1	Glutamate carboxypeptidase 2 OS=Homo sapiens OX=9606 GN=FOLH1	2.274	0.00050335
Q8IZD9	DOCK3	Dedicator of cytokinesis protein 3 OS=Homo sapiens OX=9606 GN=DOCK3	2.271	0.0020601
P53801	PTTG1IP	Pituitary tumor-transforming gene 1 protein-interacting protein OS=Homo sapiens OX=9606 GN=PTTG1IP	2.269	0.00133984
Q86YJ5	MARCH9	E3 ubiquitin-protein ligase MARCH9 OS=Homo sapiens OX=9606 GN=MARCH9	2.263	8.0034E-05
Q6IN84	MRM1	"rRNA methyltransferase 1, mitochondrial OS=Homo sapiens OX=9606 GN=MRM1"	2.261	0.00027838
Q9NQ86	TRIM36	E3 ubiquitin-protein ligase TRIM36 OS=Homo sapiens OX=9606 GN=TRIM36	2.259	3.5487E-05
Q9ULS5	TMCC3	Transmembrane and coiled-coil domain protein 3 OS=Homo sapiens OX=9606 GN=TMCC3	2.258	0.0028005
Q96HR9	REEP6	Receptor expression-enhancing protein 6 OS=Homo sapiens OX=9606 GN=REEP6	2.254	0.00019642

Q9UDW3	ZMAT5	Zinc finger matrin-type protein 5 OS=Homo sapiens OX=9606 GN=ZMAT5	2.254	0.00062107
Q9Y5U8	MPC1	Mitochondrial pyruvate carrier 1 OS=Homo sapiens OX=9606 GN=MPC1	2.254	0.0025971
Q9H115	NAPB	Beta-soluble NSF attachment protein OS=Homo sapiens OX=9606 GN=NAPB	2.252	3.8047E-06
Q99424	ACOX2	Peroxisomal acyl-coenzyme A oxidase 2 OS=Homo sapiens OX=9606 GN=ACOX2	2.251	3.7788E-05
Q13557	CAMK2D	Calcium/calmodulin-dependent protein kinase type II subunit delta OS=Homo sapiens OX=9606 GN=CAMK2D	2.248	7.7584E-05
Q11201	ST3GAL1	"CMP-N-acetylneuraminate-beta-galactosamide-alpha-2,3-sialyltransferase 1 OS=Homo sapiens OX=9606 GN=ST3GAL1"	2.242	0.00102158
Q9UBN6	TNFRSF10D	Tumor necrosis factor receptor superfamily member 10D OS=Homo sapiens OX=9606 GN=TNFRSF10D	2.238	0.00121527
Q9BYN0	SRXN1	Sulfiredoxin-1 OS=Homo sapiens OX=9606 GN=SRXN1	2.236	0.00108457
Q8IUX1	TMEM126B	"Complex I assembly factor TMEM126B, mitochondrial OS=Homo sapiens OX=9606 GN=TMEM126B"	2.235	0.00015997
Q99801	NKX3-1	Homeobox protein Nkx-3.1 OS=Homo sapiens OX=9606 GN=NKX3-1	2.23	0.0180622
Q16706	MAN2A1	Alpha-mannosidase 2 OS=Homo sapiens OX=9606 GN=MAN2A1	2.221	6.8786E-07
Q16773	KYAT1	Kynurenine--oxoglutarate transaminase 1 OS=Homo sapiens OX=9606 GN=KYAT1	2.221	0.00023502
Q9UHI8	ADAMTS1	A disintegrin and metalloproteinase with thrombospondin motifs 1 OS=Homo sapiens OX=9606 GN=ADAMTS1	2.221	0.00125784
Q9H477	RBKS	Ribokinase OS=Homo sapiens OX=9606 GN=RBKS	2.219	0.00063545
Q13011	ECH1	"Delta(3,5)-Delta(2,4)-dienoyl-CoA isomerase, mitochondrial OS=Homo sapiens OX=9606 GN=ECH1"	2.218	4.0184E-07
Q9BX59	TAPBPL	Tapasin-related protein OS=Homo sapiens OX=9606 GN=TAPBPL	2.217	0.0125764
P18084	ITGB5	Integrin beta-5 OS=Homo sapiens OX=9606 GN=ITGB5	2.21	0.00099516
P12830	CDH1	Cadherin-1 OS=Homo sapiens OX=9606 GN=CDH1	2.209	2.1766E-05
Q6ZS30	NBEAL1	Neurobeachin-like protein 1 OS=Homo sapiens OX=9606 GN=NBEAL1	2.208	0.020238
Q96A26	FAM162A	Protein FAM162A OS=Homo sapiens OX=9606 GN=FAM162A	2.206	0.0049953
Q8NEG4	FAM83F	Protein FAM83F OS=Homo sapiens OX=9606 GN=FAM83F	2.205	0.00079708
Q9NZ45	CISD1	CDGSH iron-sulfur domain-containing protein 1 OS=Homo sapiens OX=9606 GN=CISD1	2.203	0.00080244

P11474	ESRRA	Steroid hormone receptor ERR1 OS=Homo sapiens OX=9606 GN=ESRRA	2.2	1.7812E-05
Q8WZ42	TTN	Titin OS=Homo sapiens OX=9606 GN=TTN	2.192	3.9625E-05
Q96JQ2	CLMN	Calmin OS=Homo sapiens OX=9606 GN=CLMN	2.191	3.6494E-05
Q7L211	ABHD13	Protein ABHD13 OS=Homo sapiens OX=9606 GN=ABHD13	2.187	0.00024064
Q8TCD1	C18orf32	UPF0729 protein C18orf32 OS=Homo sapiens OX=9606 GN=C18orf32	2.18	0.00006453
Q9H6S3	EPS8L2	Epidermal growth factor receptor kinase substrate 8-like protein 2 OS=Homo sapiens OX=9606 GN=EPS8L2	2.18	0.00038457
Q96DT5	DNAH11	"Dynein heavy chain 11, axonemal OS=Homo sapiens OX=9606 GN=DNAH11"	2.173	0.00181785
P30043	BLVRB	Flavin reductase (NADPH) OS=Homo sapiens OX=9606 GN=BLVRB	2.171	0.00018454
Q8N8Q8	COX18	"Cytochrome c oxidase assembly protein COX18, mitochondrial OS=Homo sapiens OX=9606 GN=COX18"	2.169	0.00020021
Q9NPR9	GPR108	Protein GPR108 OS=Homo sapiens OX=9606 GN=GPR108	2.169	0.00018004
Q9P0P8	C6orf203	Uncharacterized protein C6orf203 OS=Homo sapiens OX=9606 GN=C6orf203	2.166	0.003897
O94819	KBTBD11	Kelch repeat and BTB domain-containing protein 11 OS=Homo sapiens OX=9606 GN=KBTBD11	2.165	0.00143839
Q06210	GFPT1	Glutamine--fructose-6-phosphate aminotransferase [isomerizing] 1 OS=Homo sapiens OX=9606 GN=GFPT1	2.165	4.0915E-06
O60831	PRAF2	PRA1 family protein 2 OS=Homo sapiens OX=9606 GN=PRAF2	2.159	0.00109833
Q92521	PIGB	GPI mannosyltransferase 3 OS=Homo sapiens OX=9606 GN=PIGB	2.159	0.00096086
Q9BSJ8	ESYT1	Extended synaptotagmin-1 OS=Homo sapiens OX=9606 GN=ESYT1	2.159	3.1306E-07
O60701	UGDH	UDP-glucose 6-dehydrogenase OS=Homo sapiens OX=9606 GN=UGDH	2.158	6.7922E-08
O15240	VGF	Neurosecretory protein VGF OS=Homo sapiens OX=9606 GN=VGF	2.154	4.3556E-06
Q8N398	VWA5B2	von Willebrand factor A domain-containing protein 5B2 OS=Homo sapiens OX=9606 GN=VWA5B2	2.152	0.0024156
P42126	ECI1	"Enoyl-CoA delta isomerase 1, mitochondrial OS=Homo sapiens OX=9606 GN=ECI1"	2.151	2.4601E-05
Q6UWW8	CES3	Carboxylesterase 3 OS=Homo sapiens OX=9606 GN=CES3	2.151	0.0037816
Q9GZY8	MFF	Mitochondrial fission factor OS=Homo sapiens OX=9606 GN=MFF	2.143	1.808E-05
Q86WU2	LDHD	"Probable D-lactate dehydrogenase,	2.141	0.00054476

		mitochondrial OS=Homo sapiens OX=9606 GN=LDHD"		
O75460	ERN1	Serine/threonine-protein kinase/endoribonuclease IRE1 OS=Homo sapiens OX=9606 GN=ERN1	2.137	0.00076109
Q9Y210	TRPC6	Short transient receptor potential channel 6 OS=Homo sapiens OX=9606 GN=TRPC6	2.133	8.0298E-05
Q8IVH4	MMAA	"Methylmalonic aciduria type A protein, mitochondrial OS=Homo sapiens OX=9606 GN=MMAA"	2.132	0.00011737
Q9BQ69	MACROD1	O-acetyl-ADP-ribose deacetylase MACROD1 OS=Homo sapiens OX=9606 GN=MACROD1	2.128	0.00015788
Q9GZY0	NXF2	Nuclear RNA export factor 2 OS=Homo sapiens OX=9606 GN=NXF2	2.128	2.1424E-05
Q9HA38	ZMAT3	Zinc finger matrin-type protein 3 OS=Homo sapiens OX=9606 GN=ZMAT3	2.128	0.0022783
Q12840	KIF5A	Kinesin heavy chain isoform 5A OS=Homo sapiens OX=9606 GN=KIF5A	2.127	0.0048752
Q92743	HTRA1	Serine protease HTRA1 OS=Homo sapiens OX=9606 GN=HTRA1	2.123	0.0079246
Q969X5	ERGIC1	Endoplasmic reticulum-Golgi intermediate compartment protein 1 OS=Homo sapiens OX=9606 GN=ERGIC1	2.12	3.9118E-05
O43761	SYNGR3	Synaptogyrin-3 OS=Homo sapiens OX=9606 GN=SYNGR3	2.119	0.00059629
Q01415	GALK2	N-acetylgalactosamine kinase OS=Homo sapiens OX=9606 GN=GALK2	2.119	0.00013953
Q14108	SCARB2	Lysosome membrane protein 2 OS=Homo sapiens OX=9606 GN=SCARB2	2.118	3.5074E-07
P14927	UQCRB	Cytochrome b-c1 complex subunit 7 OS=Homo sapiens OX=9606 GN=UQCRB	2.112	2.1895E-05
Q8N5G0	SMIM20	Small integral membrane protein 20 OS=Homo sapiens OX=9606 GN=SMIM20	2.111	0.00170401
O96011	PEX11B	Peroxisomal membrane protein 11B OS=Homo sapiens OX=9606 GN=PEX11B	2.108	0.00025703
P31937	HIBADH	"3-hydroxyisobutyrate dehydrogenase, mitochondrial OS=Homo sapiens OX=9606 GN=HIBADH"	2.107	8.8912E-07
Q69YL0	NCBP2-AS2	Uncharacterized protein NCBP2-AS2 OS=Homo sapiens OX=9606 GN=NCBP2-AS2	2.107	0.020118
Q9BZV1	UBXN6	UBX domain-containing protein 6 OS=Homo sapiens OX=9606 GN=UBXN6	2.103	0.00012138
P35914	HMGCL	"Hydroxymethylglutaryl-CoA lyase, mitochondrial OS=Homo sapiens OX=9606 GN=HMGCL"	2.101	0.00020063
Q9H8P0	SRD5A3	Polyprenol reductase OS=Homo sapiens OX=9606 GN=SRD5A3	2.096	0.0036199
O43822	C21orf2	Protein C21orf2 OS=Homo sapiens OX=9606 GN=C21orf2	2.091	0.007699
P02751	FN1	Fibronectin OS=Homo sapiens OX=9606	2.086	0.0003614

		GN=FN1		
Q9H0Q0	FAM49A	Protein FAM49A OS=Homo sapiens OX=9606 GN=FAM49A	2.086	0.0169771
Q14554	PDIA5	Protein disulfide-isomerase A5 OS=Homo sapiens OX=9606 GN=PDIA5	2.083	0.0020773
Q8WY54	PPM1E	Protein phosphatase 1E OS=Homo sapiens OX=9606 GN=PPM1E	2.083	3.0294E-06
Q9Y2T7	YBX2	Y-box-binding protein 2 OS=Homo sapiens OX=9606 GN=YBX2	2.081	0.027778
Q9UHK6	AMACR	Alpha-methylacyl-CoA racemase OS=Homo sapiens OX=9606 GN=AMACR	2.08	0.002002
Q13451	FKBP5	Peptidyl-prolyl cis-trans isomerase FKBP5 OS=Homo sapiens OX=9606 GN=FKBP5	2.076	0.00024277
Q15526	SURF1	Surfeit locus protein 1 OS=Homo sapiens OX=9606 GN=SURF1	2.072	4.1086E-06
Q9UI15	TAGLN3	Transgelin-3 OS=Homo sapiens OX=9606 GN=TAGLN3	2.07	0.026581
Q9Y3B3	TMED7	Transmembrane emp24 domain-containing protein 7 OS=Homo sapiens OX=9606 GN=TMED7	2.07	2.0795E-05
Q99487	PAFAH2	"Platelet-activating factor acetylhydrolase 2, cytoplasmic OS=Homo sapiens OX=9606 GN=PAFAH2"	2.069	3.7545E-05
Q8IY17	PNPLA6	Neuropathy target esterase OS=Homo sapiens OX=9606 GN=PNPLA6	2.067	0.00010174
Q99828	CIB1	Calcium and integrin-binding protein 1 OS=Homo sapiens OX=9606 GN=CIB1	2.066	0.00045774
P55055	NR1H2	Oxysterols receptor LXR-beta OS=Homo sapiens OX=9606 GN=NR1H2	2.064	7.9417E-07
Q8IZQ5	SELENOH	Selenoprotein H OS=Homo sapiens OX=9606 GN=SELENOH	2.064	0.00008379
O75764	TCEA3	Transcription elongation factor A protein 3 OS=Homo sapiens OX=9606 GN=TCEA3	2.062	0.00051541
Q13795	ARFRP1	ADP-ribosylation factor-related protein 1 OS=Homo sapiens OX=9606 GN=ARFRP1	2.061	2.9393E-06
Q15005	SPCS2	Signal peptidase complex subunit 2 OS=Homo sapiens OX=9606 GN=SPCS2	2.054	0.00023719
O43688	PLPP2	Phospholipid phosphatase 2 OS=Homo sapiens OX=9606 GN=PLPP2	2.053	0.0184606
Q9H8H3	METTL7A	Methyltransferase-like protein 7A OS=Homo sapiens OX=9606 GN=METTL7A	2.052	1.9214E-05
O94766	B3GAT3	Galactosylgalactosylxylosylprotein 3-beta-glucuronosyltransferase 3 OS=Homo sapiens OX=9606 GN=B3GAT3	2.049	0.00050328
Q8TBP6	SLC25A40	Solute carrier family 25 member 40 OS=Homo sapiens OX=9606 GN=SLC25A40	2.048	0.00130032
Q9P1P5	TAAR2	Trace amine-associated receptor 2 OS=Homo sapiens OX=9606 GN=TAAR2	2.045	1.9712E-05
P31949	S100A11	Protein S100-A11 OS=Homo sapiens OX=9606 GN=S100A11	2.043	2.0252E-06

Q6UW68	TMEM205	Transmembrane protein 205 OS=Homo sapiens OX=9606 GN=TMEM205	2.042	8.3676E-05
Q9UHL4	DPP7	Dipeptidyl peptidase 2 OS=Homo sapiens OX=9606 GN=DPP7	2.041	0.00018257
Q6PIJ6	FBXO38	F-box only protein 38 OS=Homo sapiens OX=9606 GN=FBXO38	2.04	0.00052181
Q14764	MVP	Major vault protein OS=Homo sapiens OX=9606 GN=MVP	2.039	2.3989E-06
O00519	FAAH	Fatty-acid amide hydrolase 1 OS=Homo sapiens OX=9606 GN=FAAH	2.036	0.0029786
P16260	SLC25A16	Graves disease carrier protein OS=Homo sapiens OX=9606 GN=SLC25A16	2.034	0.000138
Q6UXV4	APOOL	MICOS complex subunit MIC27 OS=Homo sapiens OX=9606 GN=APOOL	2.033	1.7982E-05
Q02218	OGDH	"2-oxoglutarate dehydrogenase, mitochondrial OS=Homo sapiens OX=9606 GN=OGDH"	2.032	4.3372E-06
Q96EP1	CHFR	E3 ubiquitin-protein ligase CHFR OS=Homo sapiens OX=9606 GN=CHFR	2.03	0.0089169
Q53FZ2	ACSM3	"Acyl-coenzyme A synthetase ACSM3, mitochondrial OS=Homo sapiens OX=9606 GN=ACSM3"	2.029	0.0030045
O15533	TAPBP	Tapasin OS=Homo sapiens OX=9606 GN=TAPBP	2.025	0.00016015
O15427	SLC16A3	Monocarboxylate transporter 4 OS=Homo sapiens OX=9606 GN=SLC16A3	2.024	0.00132405
P36969	GPX4	Phospholipid hydroperoxide glutathione peroxidase OS=Homo sapiens OX=9606 GN=GPX4	2.023	5.696E-07
Q96RQ3	MCCC1	"Methylcrotonoyl-CoA carboxylase subunit alpha, mitochondrial OS=Homo sapiens OX=9606 GN=MCCC1"	2.022	2.0483E-05
Q9H160	ING2	Inhibitor of growth protein 2 OS=Homo sapiens OX=9606 GN=ING2	2.022	0.00101741
Q13367	AP3B2	AP-3 complex subunit beta-2 OS=Homo sapiens OX=9606 GN=AP3B2	2.021	0.00036344
P37268	FDFT1	Squalene synthase OS=Homo sapiens OX=9606 GN=FDFT1	2.019	4.3524E-05
Q9UKU0	ACSL6	Long-chain-fatty-acid--CoA ligase 6 OS=Homo sapiens OX=9606 GN=ACSL6	2.018	0.00036066
Q14693	LPIN1	Phosphatidate phosphatase LPIN1 OS=Homo sapiens OX=9606 GN=LPIN1	2.016	0.045516
Q9ULD0	OGDHL	"2-oxoglutarate dehydrogenase-like, mitochondrial OS=Homo sapiens OX=9606 GN=OGDHL"	2.014	0.00070207
Q9Y252	RNF6	E3 ubiquitin-protein ligase RNF6 OS=Homo sapiens OX=9606 GN=RNF6	2.01	0.00011581
Q96D70	R3HDM4	R3H domain-containing protein 4 OS=Homo sapiens OX=9606 GN=R3HDM4	2.009	0.0033011
Q8IWA5	SLC44A2	Choline transporter-like protein 2 OS=Homo sapiens OX=9606 GN=SLC44A2	2.008	0.00164333
Q9H147	DNTTIP1	Deoxynucleotidyltransferase terminal-	2.003	0.0003822

		interacting protein 1 OS=Homo sapiens OX=9606 GN=DNTTIP1		
O95755	RAB36	Ras-related protein Rab-36 OS=Homo sapiens OX=9606 GN=RAB36	2.002	0.00136449
P83881	RPL36A	60S ribosomal protein L36a OS=Homo sapiens OX=9606 GN=RPL36A	0.499	0.0021246
O14654	IRS4	Insulin receptor substrate 4 OS=Homo sapiens OX=9606 GN=IRS4	0.498	2.1551E-05
P51956	NEK3	Serine/threonine-protein kinase Nek3 OS=Homo sapiens OX=9606 GN=NEK3	0.498	0.0010242
Q7L1Q6	BZW1	Basic leucine zipper and W2 domain-containing protein 1 OS=Homo sapiens OX=9606 GN=BZW1	0.498	1.7966E-05
P33991	MCM4	DNA replication licensing factor MCM4 OS=Homo sapiens OX=9606 GN=MCM4	0.496	7.9086E-05
Q12834	CDC20	Cell division cycle protein 20 homolog OS=Homo sapiens OX=9606 GN=CDC20	0.496	0.041616
Q86SK9	SCD5	Stearoyl-CoA desaturase 5 OS=Homo sapiens OX=9606 GN=SCD5	0.496	0.0098644
P07864	LDHC	L-lactate dehydrogenase C chain OS=Homo sapiens OX=9606 GN=LDHC	0.495	0.00102048
Q9Y4R8	TELO2	Telomere length regulation protein TEL2 homolog OS=Homo sapiens OX=9606 GN=TELO2	0.493	9.5143E-05
Q9NYP9	MIS18A	Protein Mis18-alpha OS=Homo sapiens OX=9606 GN=MIS18A	0.491	9.6845E-05
Q6FIF0	ZFAND6	AN1-type zinc finger protein 6 OS=Homo sapiens OX=9606 GN=ZFAND6	0.49	0.00064199
Q99640	PKMYT1	Membrane-associated tyrosine- and threonine-specific cdc2-inhibitory kinase OS=Homo sapiens OX=9606 GN=PKMYT1	0.49	0.00143895
Q9NVM4	PRMT7	Protein arginine N-methyltransferase 7 OS=Homo sapiens OX=9606 GN=PRMT7	0.49	0.00027513
Q02878	RPL6	60S ribosomal protein L6 OS=Homo sapiens OX=9606 GN=RPL6	0.489	0.00063522
Q8N5W9	RFLNB	Refilin-B OS=Homo sapiens OX=9606 GN=RFLNB	0.489	0.0032211
Q15555	MAPRE2	Microtubule-associated protein RP/EB family member 2 OS=Homo sapiens OX=9606 GN=MAPRE2	0.488	0.00172255
Q8NHU6	TDRD7	Tudor domain-containing protein 7 OS=Homo sapiens OX=9606 GN=TDRD7	0.488	0.00003994
P36873	PPP1CC	Serine/threonine-protein phosphatase PP1-gamma catalytic subunit OS=Homo sapiens OX=9606 GN=PPP1CC	0.487	0.0116007
O95619	YEATS4	YEATS domain-containing protein 4 OS=Homo sapiens OX=9606 GN=YEATS4	0.485	0.024384
P17482	HOXB9	Homeobox protein Hox-B9 OS=Homo sapiens OX=9606 GN=HOXB9	0.485	0.00033796
P49736	MCM2	DNA replication licensing factor MCM2 OS=Homo sapiens OX=9606 GN=MCM2	0.485	1.8334E-05
O95319	CELF2	CUGBP Elav-like family member 2	0.482	0.00074492

		OS=Homo sapiens OX=9606 GN=CELF2		
Q719H9	KCTD1	BTB/POZ domain-containing protein KCTD1 OS=Homo sapiens OX=9606 GN=KCTD1	0.482	0.00097863
Q9H857	NT5DC2	5'-nucleotidase domain-containing protein 2 OS=Homo sapiens OX=9606 GN=NT5DC2	0.482	0.00027858
Q9H8U3	ZFAND3	AN1-type zinc finger protein 3 OS=Homo sapiens OX=9606 GN=ZFAND3	0.482	0.0074787
Q9BZM4	ULBP3	UL16-binding protein 3 OS=Homo sapiens OX=9606 GN=ULBP3	0.48	0.00038196
Q9Y605	MRFAP1	MORF4 family-associated protein 1 OS=Homo sapiens OX=9606 GN=MRFAP1	0.48	0.00026076
Q9NRN7	AASDHPPT	L-aminoadipate-semialdehyde dehydrogenase-phosphopantetheinyl transferase OS=Homo sapiens OX=9606 GN=AASDHPPT	0.479	6.0143E-05
Q9NVR5	DNAAF2	Protein kintoun OS=Homo sapiens OX=9606 GN=DNAAF2	0.479	0.0020227
P62273	RPS29	40S ribosomal protein S29 OS=Homo sapiens OX=9606 GN=RPS29	0.478	0.005282
Q8TED1	GPX8	Probable glutathione peroxidase 8 OS=Homo sapiens OX=9606 GN=GPX8	0.478	7.9162E-05
Q9UKE5	TNIK	TRAF2 and NCK-interacting protein kinase OS=Homo sapiens OX=9606 GN=TNIK	0.478	3.6167E-05
Q9Y5B8	NME7	Nucleoside diphosphate kinase 7 OS=Homo sapiens OX=9606 GN=NME7	0.478	0.00050298
Q13309	SKP2	S-phase kinase-associated protein 2 OS=Homo sapiens OX=9606 GN=SKP2	0.477	0.00129969
P25205	MCM3	DNA replication licensing factor MCM3 OS=Homo sapiens OX=9606 GN=MCM3	0.474	0.00148464
P42766	RPL35	60S ribosomal protein L35 OS=Homo sapiens OX=9606 GN=RPL35	0.474	0.0052779
P83731	RPL24	60S ribosomal protein L24 OS=Homo sapiens OX=9606 GN=RPL24	0.474	0.001061
Q96JG8	MAGED4	Melanoma-associated antigen D4 OS=Homo sapiens OX=9606 GN=MAGED4	0.473	0.0183838
P49006	MARCKSL1	MARCKS-related protein OS=Homo sapiens OX=9606 GN=MARCKSL1	0.472	2.3386E-06
P61925	PKIA	cAMP-dependent protein kinase inhibitor alpha OS=Homo sapiens OX=9606 GN=PKIA	0.472	0.0013156
P09455	RBP1	Retinol-binding protein 1 OS=Homo sapiens OX=9606 GN=RBP1	0.471	6.2749E-05
Q14019	COTL1	Coactosin-like protein OS=Homo sapiens OX=9606 GN=COTL1	0.471	0.00043537
Q9Y2S6	TMA7	Translation machinery-associated protein 7 OS=Homo sapiens OX=9606 GN=TMA7	0.47	0.0059805
O43524	FOXO3	Forkhead box protein O3 OS=Homo sapiens OX=9606 GN=FOXO3	0.469	0.00067588
O75534	CSDE1	Cold shock domain-containing protein E1 OS=Homo sapiens OX=9606 GN=CSDE1	0.469	3.9621E-06
P62837	UBE2D2	Ubiquitin-conjugating enzyme E2 D2	0.468	0.036061

		OS=Homo sapiens OX=9606 GN=UBE2D2		
Q9UBU8	MORF4L1	Mortality factor 4-like protein 1 OS=Homo sapiens OX=9606 GN=MORF4L1	0.466	0.00112199
O76080	ZFAND5	AN1-type zinc finger protein 5 OS=Homo sapiens OX=9606 GN=ZFAND5	0.465	0.00020206
P17612	PRKACA	cAMP-dependent protein kinase catalytic subunit alpha OS=Homo sapiens OX=9606 GN=PRKACA	0.465	0.0063371
Q8NF64	ZMIZ2	Zinc finger MIZ domain-containing protein 2 OS=Homo sapiens OX=9606 GN=ZMIZ2	0.465	0.0052179
Q9P0P0	RNF181	E3 ubiquitin-protein ligase RNF181 OS=Homo sapiens OX=9606 GN=RNF181	0.465	0.00016243
Q9HBU6	ETNK1	Ethanolamine kinase 1 OS=Homo sapiens OX=9606 GN=ETNK1	0.464	0.0191804
Q9Y376	CAB39	Calcium-binding protein 39 OS=Homo sapiens OX=9606 GN=CAB39	0.464	0.0183988
P15121	AKR1B1	Aldose reductase OS=Homo sapiens OX=9606 GN=AKR1B1	0.463	0.0023217
P40222	TXLNA	Alpha-taxilin OS=Homo sapiens OX=9606 GN=TXLNA	0.463	1.5805E-05
Q15800	MSMO1	Methylsterol monooxygenase 1 OS=Homo sapiens OX=9606 GN=MSMO1	0.463	0.00110137
P61254	RPL26	60S ribosomal protein L26 OS=Homo sapiens OX=9606 GN=RPL26	0.461	0.0051629
Q13043	STK4	Serine/threonine-protein kinase 4 OS=Homo sapiens OX=9606 GN=STK4	0.461	0.00015811
Q9NPD8	UBE2T	Ubiquitin-conjugating enzyme E2 T OS=Homo sapiens OX=9606 GN=UBE2T	0.458	1.6181E-06
O00470	MEIS1	Homeobox protein Meis1 OS=Homo sapiens OX=9606 GN=MEIS1	0.456	8.2731E-05
Q4KWH8	PLCH1	"1-phosphatidylinositol 4,5-bisphosphate phosphodiesterase eta-1 OS=Homo sapiens OX=9606 GN=PLCH1"	0.455	0.021964
Q15398	DLGAP5	Disks large-associated protein 5 OS=Homo sapiens OX=9606 GN=DLGAP5	0.454	7.5216E-05
Q04724	TLE1	Transducin-like enhancer protein 1 OS=Homo sapiens OX=9606 GN=TLE1	0.45	1.5688E-05
P49642	PRIM1	DNA primase small subunit OS=Homo sapiens OX=9606 GN=PRIM1	0.449	0.00077598
Q86WW8	COA5	Cytochrome c oxidase assembly factor 5 OS=Homo sapiens OX=9606 GN=COA5	0.449	0.00022194
P49207	RPL34	60S ribosomal protein L34 OS=Homo sapiens OX=9606 GN=RPL34	0.448	0.0089964
P79522	PRR3	Proline-rich protein 3 OS=Homo sapiens OX=9606 GN=PRR3	0.448	0.00018101
Q9UI36	DACH1	Dachshund homolog 1 OS=Homo sapiens OX=9606 GN=DACH1	0.448	3.6264E-05
Q96F45	ZNF503	Zinc finger protein 503 OS=Homo sapiens OX=9606 GN=ZNF503	0.447	2.2273E-05
Q9BVW5	TIPIN	TIMELESS-interacting protein OS=Homo sapiens OX=9606 GN=TIPIN	0.447	2.2796E-08
Q9H7C9	AAMDC	Mth938 domain-containing protein	0.447	5.6716E-05

		OS=Homo sapiens OX=9606 GN=AAMDC		
Q15475	SIX1	Homeobox protein SIX1 OS=Homo sapiens OX=9606 GN=SIX1	0.445	0.00097655
Q68CZ1	RPGRIP1L	Protein fantom OS=Homo sapiens OX=9606 GN=RPGRIP1L	0.445	0.0035214
Q14566	MCM6	DNA replication licensing factor MCM6 OS=Homo sapiens OX=9606 GN=MCM6	0.444	1.6505E-05
O00418	EEF2K	Eukaryotic elongation factor 2 kinase OS=Homo sapiens OX=9606 GN=EEF2K	0.443	0.00012121
O43924	PDE6D	"Retinal rod rhodopsin-sensitive cGMP 3',5'-cyclic phosphodiesterase subunit delta OS=Homo sapiens OX=9606 GN=PDE6D"	0.443	0.00105583
Q5T011	SZT2	KICSTOR complex protein SZT2 OS=Homo sapiens OX=9606 GN=SZT2	0.443	0.00027942
Q14444	CAPRIN1	Caprin-1 OS=Homo sapiens OX=9606 GN=CAPRIN1	0.442	1.8607E-05
Q9NZJ9	NUDT4	Diphosphoinositol polyphosphate phosphohydrolase 2 OS=Homo sapiens OX=9606 GN=NUDT4	0.442	0.034883
P00918	CA2	Carbonic anhydrase 2 OS=Homo sapiens OX=9606 GN=CA2	0.441	0.00004076
P23458	JAK1	Tyrosine-protein kinase JAK1 OS=Homo sapiens OX=9606 GN=JAK1	0.438	0.00027586
P33993	MCM7	DNA replication licensing factor MCM7 OS=Homo sapiens OX=9606 GN=MCM7	0.438	4.6968E-06
Q8NI77	KIF18A	Kinesin-like protein KIF18A OS=Homo sapiens OX=9606 GN=KIF18A	0.438	0.0012649
A6NKD9	CCDC85C	Coiled-coil domain-containing protein 85C OS=Homo sapiens OX=9606 GN=CCDC85C	0.436	0.0029972
O96006	ZBED1	Zinc finger BED domain-containing protein 1 OS=Homo sapiens OX=9606 GN=ZBED1	0.432	0.00111576
P62875	POLR2L	"DNA-directed RNA polymerases I, II, and III subunit RPABC5 OS=Homo sapiens OX=9606 GN=POLR2L"	0.431	0.034762
Q96EL2	MRPS24	"28S ribosomal protein S24, mitochondrial OS=Homo sapiens OX=9606 GN=MRPS24"	0.43	0.0153226
Q6NXE6	ARMC6	Armadillo repeat-containing protein 6 OS=Homo sapiens OX=9606 GN=ARMC6	0.428	1.5251E-06
Q53EZ4	CEP55	Centrosomal protein of 55 kDa OS=Homo sapiens OX=9606 GN=CEP55	0.427	8.1438E-05
Q8IYJ1	CPNE9	Copine-9 OS=Homo sapiens OX=9606 GN=CPNE9	0.427	0.030524
Q16658	FSCN1	Fascin OS=Homo sapiens OX=9606 GN=FSCN1	0.426	0.0026191
Q9NR33	POLE4	DNA polymerase epsilon subunit 4 OS=Homo sapiens OX=9606 GN=POLE4	0.424	0.00011816
Q9NRZ9	HELLS	Lymphoid-specific helicase OS=Homo sapiens OX=9606 GN=HELLS	0.424	0.00011799
P43694	GATA4	Transcription factor GATA-4 OS=Homo sapiens OX=9606 GN=GATA4	0.42	0.00049609

O60911	CTSV	Cathepsin L2 OS=Homo sapiens OX=9606 GN=CTSV	0.417	0.0054015
P60520	GABARAPL2	Gamma-aminobutyric acid receptor-associated protein-like 2 OS=Homo sapiens OX=9606 GN=GABARAPL2	0.411	8.4609E-05
O75147	OBSL1	Obscurin-like protein 1 OS=Homo sapiens OX=9606 GN=OBSL1	0.409	1.3995E-06
Q99576	TSC22D3	TSC22 domain family protein 3 OS=Homo sapiens OX=9606 GN=TSC22D3	0.404	2.3996E-05
Q9Y4C2	TCAF1	TRPM8 channel-associated factor 1 OS=Homo sapiens OX=9606 GN=TCAF1	0.401	8.0561E-05
Q13685	AAMP	Angio-associated migratory cell protein OS=Homo sapiens OX=9606 GN=AAMP	0.4	5.6375E-05
Q96G61	NUDT11	Diphosphoinositol polyphosphate phosphohydrolase 3-beta OS=Homo sapiens OX=9606 GN=NUDT11	0.4	0.0027246
Q99741	CDC6	Cell division control protein 6 homolog OS=Homo sapiens OX=9606 GN=CDC6	0.396	0.0127993
O75794	CDC123	Cell division cycle protein 123 homolog OS=Homo sapiens OX=9606 GN=CDC123	0.395	9.5146E-05
O00264	PGRMC1	Membrane-associated progesterone receptor component 1 OS=Homo sapiens OX=9606 GN=PGRMC1	0.391	0.031041
P15923	TCF3	Transcription factor E2-alpha OS=Homo sapiens OX=9606 GN=TCF3	0.388	0.00023859
Q5KU26	COLEC12	Collectin-12 OS=Homo sapiens OX=9606 GN=COLEC12	0.384	4.8613E-06
P15104	GLUL	Glutamine synthetase OS=Homo sapiens OX=9606 GN=GLUL	0.38	7.7607E-07
Q8IWC1	MAP7D3	MAP7 domain-containing protein 3 OS=Homo sapiens OX=9606 GN=MAP7D3	0.38	0.00011503
O95857	TSPAN13	Tetraspanin-13 OS=Homo sapiens OX=9606 GN=TSPAN13	0.378	0.0052244
Q9H7S9	ZNF703	Zinc finger protein 703 OS=Homo sapiens OX=9606 GN=ZNF703	0.373	1.8773E-05
Q9Y291	MRPS33	"28S ribosomal protein S33, mitochondrial OS=Homo sapiens OX=9606 GN=MRPS33"	0.359	0.024403
Q99538	LGMN	Legumain OS=Homo sapiens OX=9606 GN=LGMN	0.356	0.00135951
O00762	UBE2C	Ubiquitin-conjugating enzyme E2 C OS=Homo sapiens OX=9606 GN=UBE2C	0.353	0.00003741
Q04727	TLE4	Transducin-like enhancer protein 4 OS=Homo sapiens OX=9606 GN=TLE4	0.353	0.0049396
Q4VCS5	AMOT	Angiomotin OS=Homo sapiens OX=9606 GN=AMOT	0.345	3.6939E-06
O14786	NRP1	Neuropilin-1 OS=Homo sapiens OX=9606 GN=NRP1	0.338	0.00005588
Q15375	EPHA7	Ephrin type-A receptor 7 OS=Homo sapiens OX=9606 GN=EPHA7	0.334	1.5608E-05
Q9H2J7	SLC6A15	Sodium-dependent neutral amino acid transporter B(0)AT2 OS=Homo sapiens	0.334	5.5795E-05

		OX=9606 GN=SLC6A15		
Q01826	SATB1	DNA-binding protein SATB1 OS=Homo sapiens OX=9606 GN=SATB1	0.33	0.00012172
Q15928	ZNF141	Zinc finger protein 141 OS=Homo sapiens OX=9606 GN=ZNF141	0.33	0.0079357
Q15004	PCLAF	PCNA-associated factor OS=Homo sapiens OX=9606 GN=PCLAF	0.325	0.00013763
Q6P4I2	WDR73	WD repeat-containing protein 73 OS=Homo sapiens OX=9606 GN=WDR73	0.319	0.006303
Q71DI3	HIST2H3A	Histone H3.2 OS=Homo sapiens OX=9606 GN=HIST2H3A	0.317	0.0026418
A4UGR9	XIRP2	Xin actin-binding repeat-containing protein 2 OS=Homo sapiens OX=9606 GN=XIRP2	0.312	0.0026019
P98179	RBM3	RNA-binding protein 3 OS=Homo sapiens OX=9606 GN=RBM3	0.309	0.00131537
Q96B01	RAD51AP1	RAD51-associated protein 1 OS=Homo sapiens OX=9606 GN=RAD51AP1	0.309	0.00086198
P33552	CKS2	Cyclin-dependent kinases regulatory subunit 2 OS=Homo sapiens OX=9606 GN=CKS2	0.3	0.0108583
Q8IUR0	TRAPPC5	Trafficking protein particle complex subunit 5 OS=Homo sapiens OX=9606 GN=TRAPPC5	0.29	0.0093005
Q8N4C8	MINK1	Misshapen-like kinase 1 OS=Homo sapiens OX=9606 GN=MINK1	0.29	0.00017928
O95149	SNUPN	Snurportin-1 OS=Homo sapiens OX=9606 GN=SNUPN	0.289	1.8176E-05
P61024	CKS1B	Cyclin-dependent kinases regulatory subunit 1 OS=Homo sapiens OX=9606 GN=CKS1B	0.289	0.0059824
P54826	GAS1	Growth arrest-specific protein 1 OS=Homo sapiens OX=9606 GN=GAS1	0.28	0.0102035
Q96GN5	CDCA7L	Cell division cycle-associated 7-like protein OS=Homo sapiens OX=9606 GN=CDCA7L	0.266	0.00021813
P10412	HIST1H1E	Histone H1.4 OS=Homo sapiens OX=9606 GN=HIST1H1E	0.251	0.0083835
Q9BWT1	CDCA7	Cell division cycle-associated protein 7 OS=Homo sapiens OX=9606 GN=CDCA7	0.144	3.7251E-05

Table S3 Differentially expressed proteins in 22Rv1 epiDTP cells

Table S4. Differentially expressed proteins in 22Rv1 enzDTP cells (>2 fold, p<0.05)

Accession	Gene	Protein description	Ratio	P value
A6NCL1	GMNC	Geminin coiled-coil domain-containing protein 1 OS=Homo sapiens OX=9606 GN=GMNC	35.758	1.8356E-06
Q8IWY8	ZSCAN29	Zinc finger and SCAN domain-containing protein 29 OS=Homo sapiens OX=9606 GN=ZSCAN29	18.652	0.0067013
O60292	SIPA1L3	Signal-induced proliferation-associated 1-like protein 3 OS=Homo sapiens OX=9606 GN=SIPA1L3	16.477	2.3753E-07

O15050	TRANK1	TPR and ankyrin repeat-containing protein 1 OS=Homo sapiens OX=9606 GN=TRANK1	15.412	1.9001E-05
Q13233	MAP3K1	Mitogen-activated protein kinase kinase kinase 1 OS=Homo sapiens OX=9606 GN=MAP3K1	14.129	4.8996E-08
Q9P1A6	DLGAP2	Disks large-associated protein 2 OS=Homo sapiens OX=9606 GN=DLGAP2	12.414	0.0127218
P11055	MYH3	Myosin-3 OS=Homo sapiens OX=9606 GN=MYH3	12.231	3.0784E-06
Q6ZUS6	CCDC149	Coiled-coil domain-containing protein 149 OS=Homo sapiens OX=9606 GN=CCDC149	12.2	3.3476E-06
O75969	AKAP3	A-kinase anchor protein 3 OS=Homo sapiens OX=9606 GN=AKAP3	10.585	9.5497E-05
Q9HCF6	TRPM3	Transient receptor potential cation channel subfamily M member 3 OS=Homo sapiens OX=9606 GN=TRPM3	10.181	4.6533E-06
Q16787	LAMA3	Laminin subunit alpha-3 OS=Homo sapiens OX=9606 GN=LAMA3	9.44	0.00032205
Q8NHH1	TTLL11	Tubulin polyglutamylase TTLL11 OS=Homo sapiens OX=9606 GN=TTLL11	9.186	2.2514E-05
Q53GL7	PARP10	Poly [ADP-ribose] polymerase 10 OS=Homo sapiens OX=9606 GN=PARP10	9.129	0.0071811
Q92835	INPP5D	"Phosphatidylinositol 3,4,5-trisphosphate 5-phosphatase 1 OS=Homo sapiens OX=9606 GN=INPP5D"	9.117	4.1722E-06
Q8TDM6	DLG5	Disks large homolog 5 OS=Homo sapiens OX=9606 GN=DLG5	8.939	0.0020995
Q8WV93	AFG1L	AFG1-like ATPase OS=Homo sapiens OX=9606 GN=AFG1L	8.526	0.00005891
P08138	NGFR	Tumor necrosis factor receptor superfamily member 16 OS=Homo sapiens OX=9606 GN=NGFR	7.524	0.00017586
Q9NYU1	UGGT2	UDP-glucose:glycoprotein glucosyltransferase 2 OS=Homo sapiens OX=9606 GN=UGGT2	7.258	0.00006429
Q6IEE8	SLFN12L	Schlafen family member 12-like OS=Homo sapiens OX=9606 GN=SLFN12L	7.081	2.0007E-05
Q8IW40	CCDC103	Coiled-coil domain-containing protein 103 OS=Homo sapiens OX=9606 GN=CCDC103	7.049	2.0346E-05
Q5TZF3	ANKRD45	Ankyrin repeat domain-containing protein 45 OS=Homo sapiens OX=9606 GN=ANKRD45	6.976	0.00000209
Q7Z5U6	WDR53	WD repeat-containing protein 53 OS=Homo sapiens OX=9606 GN=WDR53	6.643	7.8868E-05
Q9P2P6	STARD9	StAR-related lipid transfer protein 9 OS=Homo sapiens OX=9606 GN=STARD9	6.495	6.4449E-05
Q9BXL7	CARD11	Caspase recruitment domain-containing protein 11 OS=Homo sapiens OX=9606 GN=CARD11	6.469	1.5353E-05
Q9P219	CCDC88C	Protein Daple OS=Homo sapiens OX=9606 GN=CCDC88C	6.449	9.9884E-05
Q9UHD4	CIDEB	Cell death activator CIDE-B OS=Homo	6.43	1.5912E-05

		sapiens OX=9606 GN=CIDEB		
Q86X45	LRRC6	Protein tilB homolog OS=Homo sapiens OX=9606 GN=LRRC6	6.275	1.6669E-05
P10632	CYP2C8	Cytochrome P450 2C8 OS=Homo sapiens OX=9606 GN=CYP2C8	6.203	2.1987E-08
Q9HCI5	MAGEE1	Melanoma-associated antigen E1 OS=Homo sapiens OX=9606 GN=MAGEE1	5.779	1.9726E-06
Q8IW19	APLF	Aprataxin and PNK-like factor OS=Homo sapiens OX=9606 GN=APLF	5.7	0.0023801
P08575	PTPRC	Receptor-type tyrosine-protein phosphatase C OS=Homo sapiens OX=9606 GN=PTPRC	5.682	4.0509E-05
Q9ULW5	RAB26	Ras-related protein Rab-26 OS=Homo sapiens OX=9606 GN=RAB26	5.645	1.3681E-06
Q9NPJ4	PNRC2	Proline-rich nuclear receptor coactivator 2 OS=Homo sapiens OX=9606 GN=PNRC2	5.616	0.0012848
P54253	ATXN1	Ataxin-1 OS=Homo sapiens OX=9606 GN=ATXN1	5.5	2.3673E-06
P0CG24	ZNF883	Zinc finger protein 883 OS=Homo sapiens OX=9606 GN=ZNF883	5.448	3.9875E-05
Q9BVV6	KIAA0586	Protein TALPID3 OS=Homo sapiens OX=9606 GN=KIAA0586	5.363	1.8091E-05
Q9UKW4	VAV3	Guanine nucleotide exchange factor VAV3 OS=Homo sapiens OX=9606 GN=VAV3	5.355	0.00004342
Q2KHT4	GSG1	Germ cell-specific gene 1 protein OS=Homo sapiens OX=9606 GN=GSG1	5.32	1.5686E-05
P58499	FAM3B	Protein FAM3B OS=Homo sapiens OX=9606 GN=FAM3B	5.318	1.8341E-06
Q6DHV5	CC2D2B	Protein CC2D2B OS=Homo sapiens OX=9606 GN=CC2D2B	5.308	2.1393E-05
Q96P20	NLRP3	"NACHT, LRR and PYD domains-containing protein 3 OS=Homo sapiens OX=9606 GN=NLRP3"	5.275	0.0020152
Q53FA7	TP53I3	Quinone oxidoreductase PIG3 OS=Homo sapiens OX=9606 GN=TP53I3	5.227	2.4569E-05
C9JSJ3	BHMG1	Basic helix-loop-helix and HMG box domain-containing protein 1 OS=Homo sapiens OX=9606 GN=BHMG1	5.224	8.0217E-05
Q5MAI5	CDKL4	Cyclin-dependent kinase-like 4 OS=Homo sapiens OX=9606 GN=CDKL4	5.208	0.0073814
Q93075	TATDN2	Putative deoxyribonuclease TATDN2 OS=Homo sapiens OX=9606 GN=TATDN2	5.178	3.5815E-05
Q9Y6Q1	CAPN6	Calpain-6 OS=Homo sapiens OX=9606 GN=CAPN6	5.048	1.4022E-09
Q9H3T3	SEMA6B	Semaphorin-6B OS=Homo sapiens OX=9606 GN=SEMA6B	4.958	0.0072421
P38570	ITGAE	Integrin alpha-E OS=Homo sapiens OX=9606 GN=ITGAE	4.95	4.6284E-06
Q13009	TIAM1	T-lymphoma invasion and metastasis-inducing protein 1 OS=Homo sapiens OX=9606 GN=TIAM1	4.945	0.00043826
Q9NQ35	NRIP3	Nuclear receptor-interacting protein 3 OS=Homo sapiens OX=9606 GN=NRIP3	4.901	0.006583

Q8N126	CADM3	Cell adhesion molecule 3 OS=Homo sapiens OX=9606 GN=CADM3	4.804	1.7553E-05
Q9Y6V0	PCLO	Protein piccolo OS=Homo sapiens OX=9606 GN=PCLO	4.764	2.4262E-05
O60262	GNG7	Guanine nucleotide-binding protein G(I)/G(S)/G(O) subunit gamma-7 OS=Homo sapiens OX=9606 GN=GNG7	4.638	2.1049E-05
Q8TF64	GIPC3	PDZ domain-containing protein GIPC3 OS=Homo sapiens OX=9606 GN=GIPC3	4.41	0.00013743
Q86YW9	MED12L	Mediator of RNA polymerase II transcription subunit 12-like protein OS=Homo sapiens OX=9606 GN=MED12L	4.4	7.6188E-05
Q9HBT6	CDH20	Cadherin-20 OS=Homo sapiens OX=9606 GN=CDH20	4.382	4.2252E-05
Q8N431	RASGEF1C	Ras-GEF domain-containing family member 1C OS=Homo sapiens OX=9606 GN=RASGEF1C	4.297	0.008103
Q96QU1	PCDH15	Protocadherin-15 OS=Homo sapiens OX=9606 GN=PCDH15	4.229	0.00049551
A4FU49	SH3D21	SH3 domain-containing protein 21 OS=Homo sapiens OX=9606 GN=SH3D21	4.124	4.698E-06
Q5SRE7	PHYHD1	Phytanoyl-CoA dioxygenase domain-containing protein 1 OS=Homo sapiens OX=9606 GN=PHYHD1	4.117	0.0067011
P0C7M7	ACSM4	"Acyl-coenzyme A synthetase ACSM4, mitochondrial OS=Homo sapiens OX=9606 GN=ACSM4"	4.105	0.00020041
P17676	CEBPB	CCAAT/enhancer-binding protein beta OS=Homo sapiens OX=9606 GN=CEBPB	4.105	0.00045866
A6NFE2	SMCO2	Single-pass membrane and coiled-coil domain-containing protein 2 OS=Homo sapiens OX=9606 GN=SMCO2	4.051	1.6523E-06
Q96MR9	ZNF560	Zinc finger protein 560 OS=Homo sapiens OX=9606 GN=ZNF560	3.895	0.0049172
Q6ZTQ3	RASSF6	Ras association domain-containing protein 6 OS=Homo sapiens OX=9606 GN=RASSF6	3.803	1.881E-05
Q9BXU7	USP26	Ubiquitin carboxyl-terminal hydrolase 26 OS=Homo sapiens OX=9606 GN=USP26	3.799	1.5271E-05
Q9P1P5	TAAR2	Trace amine-associated receptor 2 OS=Homo sapiens OX=9606 GN=TAAR2	3.756	8.0664E-07
O43374	RASA4	Ras GTPase-activating protein 4 OS=Homo sapiens OX=9606 GN=RASA4	3.75	3.4881E-06
Q8TF21	ANKRD24	Ankyrin repeat domain-containing protein 24 OS=Homo sapiens OX=9606 GN=ANKRD24	3.745	3.6187E-05
Q8IVF4	DNAH10	"Dynein heavy chain 10, axonemal OS=Homo sapiens OX=9606 GN=DNAH10"	3.662	0.00104287
Q5S007	LRRK2	Leucine-rich repeat serine/threonine-protein kinase 2 OS=Homo sapiens OX=9606 GN=LRRK2	3.639	0.00017831
P18847	ATF3	Cyclic AMP-dependent transcription factor	3.591	2.2709E-06

		ATF-3 OS=Homo sapiens OX=9606 GN=ATF3		
P17936	IGFBP3	Insulin-like growth factor-binding protein 3 OS=Homo sapiens OX=9606 GN=IGFBP3	3.579	5.8952E-05
Q9GZY6	LAT2	Linker for activation of T-cells family member 2 OS=Homo sapiens OX=9606 GN=LAT2	3.546	9.5373E-05
P56524	HDAC4	Histone deacetylase 4 OS=Homo sapiens OX=9606 GN=HDAC4	3.539	0.00027691
Q9NU19	TBC1D22B	TBC1 domain family member 22B OS=Homo sapiens OX=9606 GN=TBC1D22B	3.512	1.6717E-06
Q8TCN5	ZNF507	Zinc finger protein 507 OS=Homo sapiens OX=9606 GN=ZNF507	3.482	2.2696E-05
P25815	S100P	Protein S100-P OS=Homo sapiens OX=9606 GN=S100P	3.462	2.5955E-07
Q99541	PLIN2	Perilipin-2 OS=Homo sapiens OX=9606 GN=PLIN2	3.46	4.5819E-06
O95445	APOM	Apolipoprotein M OS=Homo sapiens OX=9606 GN=APOM	3.385	0.0006221
Q9ULE3	DENND2A	DENN domain-containing protein 2A OS=Homo sapiens OX=9606 GN=DENND2A	3.36	0.0014391
Q9HA38	ZMAT3	Zinc finger matrin-type protein 3 OS=Homo sapiens OX=9606 GN=ZMAT3	3.281	5.6886E-05
Q5SRD1	TIMM23B	Putative mitochondrial import inner membrane translocase subunit Tim23B OS=Homo sapiens OX=9606 GN=TIMM23B	3.253	0.00048437
Q99988	GDF15	Growth/differentiation factor 15 OS=Homo sapiens OX=9606 GN=GDF15	3.253	1.8596E-05
Q9GZM7	TINAGL1	Tubulointerstitial nephritis antigen-like OS=Homo sapiens OX=9606 GN=TINAGL1	3.219	0.00012297
Q9UM63	PLAGL1	Zinc finger protein PLAGL1 OS=Homo sapiens OX=9606 GN=PLAGL1	3.188	0.0117447
Q5VTL7	FNDC7	Fibronectin type III domain-containing protein 7 OS=Homo sapiens OX=9606 GN=FNDC7	3.186	0.00084299
Q9NXT0	ZNF586	Zinc finger protein 586 OS=Homo sapiens OX=9606 GN=ZNF586	3.141	5.8079E-05
O60658	PDE8A	"High affinity cAMP-specific and IBMX-insensitive 3',5'-cyclic phosphodiesterase 8A OS=Homo sapiens OX=9606 GN=PDE8A"	3.136	0.0126199
Q9UGL1	KDM5B	Lysine-specific demethylase 5B OS=Homo sapiens OX=9606 GN=KDM5B	3.063	8.4335E-05
Q9BZ71	PITPNM3	Membrane-associated phosphatidylinositol transfer protein 3 OS=Homo sapiens OX=9606 GN=PITPNM3	3.06	8.2027E-05
O75635	SERPINB7	Serpin B7 OS=Homo sapiens OX=9606 GN=SERPINB7	3.035	0.00044425
Q5BJE1	CCDC178	Coiled-coil domain-containing protein 178 OS=Homo sapiens OX=9606 GN=CCDC178	3.029	0.00014121

Q92563	SPOCK2	Testican-2 OS=Homo sapiens OX=9606 GN=SPOCK2	3.022	0.00032395
Q96K75	ZNF514	Zinc finger protein 514 OS=Homo sapiens OX=9606 GN=ZNF514	2.907	4.0102E-05
Q9Y4C4	MFHAS1	Malignant fibrous histiocytoma-amplified sequence 1 OS=Homo sapiens OX=9606 GN=MFHAS1	2.892	0.00045952
O95271	TNKS	Tankyrase-1 OS=Homo sapiens OX=9606 GN=TNKS	2.845	4.4533E-05
Q00987	MDM2	E3 ubiquitin-protein ligase Mdm2 OS=Homo sapiens OX=9606 GN=MDM2	2.836	0.00087894
Q5TB80	CEP162	Centrosomal protein of 162 kDa OS=Homo sapiens OX=9606 GN=CEP162	2.835	0.00024422
P02751	FN1	Fibronectin OS=Homo sapiens OX=9606 GN=FN1	2.826	0.00011663
Q58FG1	HSP90AA4P	Putative heat shock protein HSP 90-alpha A4 OS=Homo sapiens OX=9606 GN=HSP90AA4P	2.822	0.00046241
P51790	CLCN3	H(+)/Cl(-) exchange transporter 3 OS=Homo sapiens OX=9606 GN=CLCN3	2.816	0.00026257
P08631	HCK	Tyrosine-protein kinase HCK OS=Homo sapiens OX=9606 GN=HCK	2.801	0.00011512
Q7L211	ABHD13	Protein ABHD13 OS=Homo sapiens OX=9606 GN=ABHD13	2.752	0.00029758
Q5VTY9	HHAT	Protein-cysteine N-palmitoyltransferase HHAT OS=Homo sapiens OX=9606 GN=HHAT	2.738	0.00037729
Q01650	SLC7A5	Large neutral amino acids transporter small subunit 1 OS=Homo sapiens OX=9606 GN=SLC7A5	2.713	3.5787E-05
Q9Y253	POLH	DNA polymerase eta OS=Homo sapiens OX=9606 GN=POLH	2.681	2.4257E-05
Q14108	SCARB2	Lysosome membrane protein 2 OS=Homo sapiens OX=9606 GN=SCARB2	2.679	7.0681E-07
Q8N3R3	TCAIM	"T-cell activation inhibitor, mitochondrial OS=Homo sapiens OX=9606 GN=TCAIM"	2.677	0.00023929
P51178	PLCD1	"1-phosphatidylinositol 4,5-bisphosphate phosphodiesterase delta-1 OS=Homo sapiens OX=9606 GN=PLCD1"	2.655	1.5979E-05
Q02447	SP3	Transcription factor Sp3 OS=Homo sapiens OX=9606 GN=SP3	2.627	0.00041885
Q86YJ5	MARCH9	E3 ubiquitin-protein ligase MARCH9 OS=Homo sapiens OX=9606 GN=MARCH9	2.618	8.4505E-05
Q14520	HABP2	Hyaluronan-binding protein 2 OS=Homo sapiens OX=9606 GN=HABP2	2.612	0.00002261
Q9UN30	SCML1	Sex comb on midleg-like protein 1 OS=Homo sapiens OX=9606 GN=SCML1	2.591	0.00019885
Q6ZN28	MACC1	Metastasis-associated in colon cancer protein 1 OS=Homo sapiens OX=9606 GN=MACC1	2.589	4.0864E-05
P53667	LIMK1	LIM domain kinase 1 OS=Homo sapiens OX=9606 GN=LIMK1	2.574	0.00020376
Q8WZ42	TTN	Titin OS=Homo sapiens OX=9606 GN=TTN	2.567	4.9775E-06

P25445	FAS	Tumor necrosis factor receptor superfamily member 6 OS=Homo sapiens OX=9606 GN=FAS	2.566	0.00052361
O14763	TNFRSF10B	Tumor necrosis factor receptor superfamily member 10B OS=Homo sapiens OX=9606 GN=TNFRSF10B	2.554	1.7786E-05
P49747	COMP	Cartilage oligomeric matrix protein OS=Homo sapiens OX=9606 GN=COMP	2.518	0.00043671
P19971	TYMP	Thymidine phosphorylase OS=Homo sapiens OX=9606 GN=TYMP	2.509	1.9514E-05
O43502	RAD51C	DNA repair protein RAD51 homolog 3 OS=Homo sapiens OX=9606 GN=RAD51C	2.499	0.0045405
Q9GZR1	SENP6	Sentrin-specific protease 6 OS=Homo sapiens OX=9606 GN=SENP6	2.495	1.5374E-05
Q9ULD2	MTUS1	Microtubule-associated tumor suppressor 1 OS=Homo sapiens OX=9606 GN=MTUS1	2.478	0.0002025
Q9BZC7	ABCA2	ATP-binding cassette sub-family A member 2 OS=Homo sapiens OX=9606 GN=ABCA2	2.477	4.4432E-05
P22570	FDXR	"NADPH:adrenodoxin oxidoreductase, mitochondrial OS=Homo sapiens OX=9606 GN=FDXR"	2.449	3.0157E-07
Q8IYW2	CFAP46	Cilia- and flagella-associated protein 46 OS=Homo sapiens OX=9606 GN=CFAP46	2.446	0.0034797
O60503	ADCY9	Adenylate cyclase type 9 OS=Homo sapiens OX=9606 GN=ADCY9	2.445	0.00015547
Q92521	PIGB	GPI mannosyltransferase 3 OS=Homo sapiens OX=9606 GN=PIGB	2.435	0.00002123
Q92597	NDRG1	Protein NDRG1 OS=Homo sapiens OX=9606 GN=NDRG1	2.43	0.00059957
Q8IYK2	CCDC105	Coiled-coil domain-containing protein 105 OS=Homo sapiens OX=9606 GN=CCDC105	2.411	1.5692E-05
Q9UGT4	SUSD2	Sushi domain-containing protein 2 OS=Homo sapiens OX=9606 GN=SUSD2	2.397	0.002844
Q9H160	ING2	Inhibitor of growth protein 2 OS=Homo sapiens OX=9606 GN=ING2	2.385	0.00060402
P43007	SLC1A4	Neutral amino acid transporter A OS=Homo sapiens OX=9606 GN=SLC1A4	2.374	2.4518E-06
Q8IZT6	ASPM	Abnormal spindle-like microcephaly-associated protein OS=Homo sapiens OX=9606 GN=ASPM	2.357	0.0032035
Q9HAT2	SIAE	Sialate O-acetylesterase OS=Homo sapiens OX=9606 GN=SIAE	2.339	0.00025933
Q8IZ52	CHPF	Chondroitin sulfate synthase 2 OS=Homo sapiens OX=9606 GN=CHPF	2.336	0.00074179
P11474	ESRRA	Steroid hormone receptor ERR1 OS=Homo sapiens OX=9606 GN=ESRRA	2.319	4.2755E-06
Q8NCG7	DAGLB	Sn1-specific diacylglycerol lipase beta OS=Homo sapiens OX=9606 GN=DAGLB	2.286	0.0063565
Q96EP1	CHFR	E3 ubiquitin-protein ligase CHFR OS=Homo sapiens OX=9606 GN=CHFR	2.283	0.0100233
Q9Y5U8	MPC1	Mitochondrial pyruvate carrier 1 OS=Homo sapiens OX=9606 GN=MPC1	2.274	0.00017618

Q9H477	RBKS	Ribokinase OS=Homo sapiens OX=9606 GN=RBKS	2.255	0.00030017
P98160	HSPG2	Basement membrane-specific heparan sulfate proteoglycan core protein OS=Homo sapiens OX=9606 GN=HSPG2	2.254	0.0046985
Q562E7	WDR81	WD repeat-containing protein 81 OS=Homo sapiens OX=9606 GN=WDR81	2.254	1.7889E-05
O75638	CTAG2	Cancer/testis antigen 2 OS=Homo sapiens OX=9606 GN=CTAG2	2.247	0.0031761
Q9H322	VCX2	Variable charge X-linked protein 2 OS=Homo sapiens OX=9606 GN=VCX2	2.246	0.0088038
P32929	CTH	Cystathionine gamma-lyase OS=Homo sapiens OX=9606 GN=CTH	2.245	4.4488E-06
Q9BQ04	RBM4B	RNA-binding protein 4B OS=Homo sapiens OX=9606 GN=RBM4B	2.241	0.00025787
O14967	CLGN	Calmegin OS=Homo sapiens OX=9606 GN=CLGN	2.236	1.5627E-05
O00622	CYR61	Protein CYR61 OS=Homo sapiens OX=9606 GN=CYR61	2.223	0.0072952
P11169	SLC2A3	"Solute carrier family 2, facilitated glucose transporter member 3 OS=Homo sapiens OX=9606 GN=SLC2A3"	2.218	0.0033216
P53801	PTTG1IP	Pituitary tumor-transforming gene 1 protein-interacting protein OS=Homo sapiens OX=9606 GN=PTTG1IP	2.213	0.00035669
Q08431	MFGE8	Lactadherin OS=Homo sapiens OX=9606 GN=MFGE8	2.202	0.00049664
Q6P1A2	LPCAT3	Lysophospholipid acyltransferase 5 OS=Homo sapiens OX=9606 GN=LPCAT3	2.201	0.00007694
Q7Z5Q5	POLN	DNA polymerase nu OS=Homo sapiens OX=9606 GN=POLN	2.197	0.0051802
Q3MIT2	PUS10	Putative tRNA pseudouridine synthase Pus10 OS=Homo sapiens OX=9606 GN=PUS10	2.187	0.00052248
Q16610	ECM1	Extracellular matrix protein 1 OS=Homo sapiens OX=9606 GN=ECM1	2.178	6.0428E-05
Q68D85	NCR3LG1	Natural cytotoxicity triggering receptor 3 ligand 1 OS=Homo sapiens OX=9606 GN=NCR3LG1	2.171	9.6004E-05
Q9H0V1	TMEM168	Transmembrane protein 168 OS=Homo sapiens OX=9606 GN=TMEM168	2.168	0.00125961
O60613	SELENOF	Selenoprotein F OS=Homo sapiens OX=9606 GN=SELENOF	2.149	7.8647E-05
Q9NXG6	P4HTM	Transmembrane prolyl 4-hydroxylase OS=Homo sapiens OX=9606 GN=P4HTM	2.147	0.0003406
Q9GZT6	CCDC90B	"Coiled-coil domain-containing protein 90B, mitochondrial OS=Homo sapiens OX=9606 GN=CCDC90B"	2.138	5.5567E-05
P08910	ABHD2	Monoacylglycerol lipase ABHD2 OS=Homo sapiens OX=9606 GN=ABHD2	2.137	0.0020845
Q8WVV4	POF1B	Protein POF1B OS=Homo sapiens OX=9606 GN=POF1B	2.13	8.3128E-05
Q9H3R2	MUC13	Mucin-13 OS=Homo sapiens OX=9606	2.123	4.1726E-05

		GN=MUC13		
P00488	F13A1	Coagulation factor XIII A chain OS=Homo sapiens OX=9606 GN=F13A1	2.115	0.00115974
P08195	SLC3A2	4F2 cell-surface antigen heavy chain OS=Homo sapiens OX=9606 GN=SLC3A2	2.114	6.5718E-07
Q6PIJ6	FBXO38	F-box only protein 38 OS=Homo sapiens OX=9606 GN=FBXO38	2.114	8.0582E-05
Q13563	PKD2	Polycystin-2 OS=Homo sapiens OX=9606 GN=PKD2	2.113	0.00010435
O15427	SLC16A3	Monocarboxylate transporter 4 OS=Homo sapiens OX=9606 GN=SLC16A3	2.111	0.00159941
O95755	RAB36	Ras-related protein Rab-36 OS=Homo sapiens OX=9606 GN=RAB36	2.111	0.00039813
Q01831	XPC	DNA repair protein complementing XP-C cells OS=Homo sapiens OX=9606 GN=XPC	2.11	0.0020156
Q12840	KIF5A	Kinesin heavy chain isoform 5A OS=Homo sapiens OX=9606 GN=KIF5A	2.101	0.00087638
Q8NFT2	STEAP2	Metalloreductase STEAP2 OS=Homo sapiens OX=9606 GN=STEAP2	2.098	1.9126E-05
Q93096	PTP4A1	Protein tyrosine phosphatase type IVA 1 OS=Homo sapiens OX=9606 GN=PTP4A1	2.09	7.5303E-07
Q9UPY5	SLC7A11	Cystine/glutamate transporter OS=Homo sapiens OX=9606 GN=SLC7A11	2.082	0.00146469
Q8NEZ4	KMT2C	Histone-lysine N-methyltransferase 2C OS=Homo sapiens OX=9606 GN=KMT2C	2.075	4.4058E-05
Q9BV19	C1orf50	Uncharacterized protein C1orf50 OS=Homo sapiens OX=9606 GN=C1orf50	2.068	0.030055
Q5CZA5	ZNF805	Zinc finger protein 805 OS=Homo sapiens OX=9606 GN=ZNF805	2.062	0.00814
Q96LJ7	DHRS1	Dehydrogenase/reductase SDR family member 1 OS=Homo sapiens OX=9606 GN=DHRS1	2.061	0.0001378
O14880	MGST3	Microsomal glutathione S-transferase 3 OS=Homo sapiens OX=9606 GN=MGST3	2.059	0.0133215
Q96SQ9	CYP2S1	Cytochrome P450 2S1 OS=Homo sapiens OX=9606 GN=CYP2S1	2.059	0.0032592
Q92743	HTRA1	Serine protease HTRA1 OS=Homo sapiens OX=9606 GN=HTRA1	2.057	0.0030197
Q8N8U2	CDYL2	Chromodomain Y-like protein 2 OS=Homo sapiens OX=9606 GN=CDYL2	2.047	4.2036E-05
O00462	MANBA	Beta-mannosidase OS=Homo sapiens OX=9606 GN=MANBA	2.041	3.6762E-05
Q9UBN6	TNFRSF10D	Tumor necrosis factor receptor superfamily member 10D OS=Homo sapiens OX=9606 GN=TNFRSF10D	2.032	0.00197647
Q8NBN3	TMEM87A	Transmembrane protein 87A OS=Homo sapiens OX=9606 GN=TMEM87A	2.026	1.922E-05
Q9Y2M0	FAN1	Fanconi-associated nuclease 1 OS=Homo sapiens OX=9606 GN=FAN1	2.026	0.0036045
Q9H0T7	RAB17	Ras-related protein Rab-17 OS=Homo sapiens OX=9606 GN=RAB17	2.023	0.00016198
Q5TF21	SOGA3	Protein SOGA3 OS=Homo sapiens	2.016	3.9092E-05

		OX=9606 GN=SOGA3		
Q96A26	FAM162A	Protein FAM162A OS=Homo sapiens OX=9606 GN=FAM162A	2.013	1.8128E-05
P07203	GPX1	Glutathione peroxidase 1 OS=Homo sapiens OX=9606 GN=GPX1	2.012	4.0477E-05
Q99611	SEPHS2	"Selenide, water dikinase 2 OS=Homo sapiens OX=9606 GN=SEPHS2"	2.011	0.00014216
Q8TD22	SFXN5	Sideroflexin-5 OS=Homo sapiens OX=9606 GN=SFXN5	2.01	9.7029E-05
Q96NL8	C8orf37	Protein C8orf37 OS=Homo sapiens OX=9606 GN=C8orf37	2.01	0.020796
Q9BU23	LMF2	Lipase maturation factor 2 OS=Homo sapiens OX=9606 GN=LMF2	2.009	0.00035591
P33121	ACSL1	Long-chain-fatty-acid--CoA ligase 1 OS=Homo sapiens OX=9606 GN=ACSL1	2.006	1.1485E-06
Q9GZX9	TWSG1	Twisted gastrulation protein homolog 1 OS=Homo sapiens OX=9606 GN=TWSG1	2.001	0.00106108
P49327	FASN	Fatty acid synthase OS=Homo sapiens OX=9606 GN=FASN	0.496	2.3562E-06
O00418	EEF2K	Eukaryotic elongation factor 2 kinase OS=Homo sapiens OX=9606 GN=EEF2K	0.494	0.0001981
P43487	RANBP1	Ran-specific GTPase-activating protein OS=Homo sapiens OX=9606 GN=RANBP1	0.494	1.7632E-05
P78317	RNF4	E3 ubiquitin-protein ligase RNF4 OS=Homo sapiens OX=9606 GN=RNF4	0.494	0.040515
Q9UQN3	CHMP2B	Charged multivesicular body protein 2b OS=Homo sapiens OX=9606 GN=CHMP2B	0.494	0.00010453
Q8NF64	ZMIZ2	Zinc finger MIZ domain-containing protein 2 OS=Homo sapiens OX=9606 GN=ZMIZ2	0.492	0.00079934
O75935	DCTN3	Dynactin subunit 3 OS=Homo sapiens OX=9606 GN=DCTN3	0.491	0.0084226
P04792	HSPB1	Heat shock protein beta-1 OS=Homo sapiens OX=9606 GN=HSPB1	0.491	6.4779E-05
Q9NVM4	PRMT7	Protein arginine N-methyltransferase 7 OS=Homo sapiens OX=9606 GN=PRMT7	0.491	7.9902E-05
Q14444	CAPRIN1	Caprin-1 OS=Homo sapiens OX=9606 GN=CAPRIN1	0.489	1.5764E-06
O00762	UBE2C	Ubiquitin-conjugating enzyme E2 C OS=Homo sapiens OX=9606 GN=UBE2C	0.488	0.0037016
Q9NYP9	MIS18A	Protein Mis18-alpha OS=Homo sapiens OX=9606 GN=MIS18A	0.488	0.00021769
O75147	OBSL1	Obscurin-like protein 1 OS=Homo sapiens OX=9606 GN=OBSL1	0.487	4.6871E-06
P15104	GLUL	Glutamine synthetase OS=Homo sapiens OX=9606 GN=GLUL	0.487	0.00004199
Q13685	AAMP	Angio-associated migratory cell protein OS=Homo sapiens OX=9606 GN=AAMP	0.485	6.3348E-05
P62081	RPS7	40S ribosomal protein S7 OS=Homo sapiens OX=9606 GN=RPS7	0.482	2.3098E-05
Q9UKT5	FBXO4	F-box only protein 4 OS=Homo sapiens OX=9606 GN=FBXO4	0.482	6.2049E-05
O76080	ZFAND5	AN1-type zinc finger protein 5 OS=Homo	0.481	1.871E-05

		sapiens OX=9606 GN=ZFAND5		
P62273	RPS29	40S ribosomal protein S29 OS=Homo sapiens OX=9606 GN=RPS29	0.481	0.0149965
Q9BVW5	TIPIN	TIMELESS-interacting protein OS=Homo sapiens OX=9606 GN=TIPIN	0.481	1.2249E-06
P0DPI3	CENPVL2	Centromere protein V-like protein 2 OS=Homo sapiens OX=9606 GN=CENPVL2	0.48	0.00020248
P15923	TCF3	Transcription factor E2-alpha OS=Homo sapiens OX=9606 GN=TCF3	0.478	0.00041746
P30154	PPP2R1B	Serine/threonine-protein phosphatase 2A 65 kDa regulatory subunit A beta isoform OS=Homo sapiens OX=9606 GN=PPP2R1B	0.478	0.00016242
P84101	SERF2	Small EDRK-rich factor 2 OS=Homo sapiens OX=9606 GN=SERF2	0.478	2.1645E-07
P23921	RRM1	Ribonucleoside-diphosphate reductase large subunit OS=Homo sapiens OX=9606 GN=RRM1	0.477	1.0479E-06
P49642	PRIM1	DNA primase small subunit OS=Homo sapiens OX=9606 GN=PRIM1	0.477	0.00161644
Q4VCS5	AMOT	Angiomotin OS=Homo sapiens OX=9606 GN=AMOT	0.476	1.7228E-07
O95989	NUDT3	Diphosphoinositol polyphosphate phosphohydrolase 1 OS=Homo sapiens OX=9606 GN=NUDT3	0.471	1.2498E-07
A6NKD9	CCDC85C	Coiled-coil domain-containing protein 85C OS=Homo sapiens OX=9606 GN=CCDC85C	0.47	2.4977E-06
P0DI82	TRAPPC2B	Trafficking protein particle complex subunit 2B OS=Homo sapiens OX=9606 GN=TRAPPC2B	0.47	0.00064073
O96006	ZBED1	Zinc finger BED domain-containing protein 1 OS=Homo sapiens OX=9606 GN=ZBED1	0.469	1.8053E-05
Q86X27	RALGPS2	Ras-specific guanine nucleotide-releasing factor RalGPS2 OS=Homo sapiens OX=9606 GN=RALGPS2	0.469	4.3429E-05
Q9HCP0	CSNK1G1	Casein kinase I isoform gamma-1 OS=Homo sapiens OX=9606 GN=CSNK1G1	0.469	0.00137514
Q15800	MSMO1	Methylsterol monooxygenase 1 OS=Homo sapiens OX=9606 GN=MSMO1	0.467	1.7554E-05
O75534	CSDE1	Cold shock domain-containing protein E1 OS=Homo sapiens OX=9606 GN=CSDE1	0.465	0.00059724
Q9Y5B8	NME7	Nucleoside diphosphate kinase 7 OS=Homo sapiens OX=9606 GN=NME7	0.465	0.00014008
Q15375	EPHA7	Ephrin type-A receptor 7 OS=Homo sapiens OX=9606 GN=EPHA7	0.463	1.9498E-05
Q5KU26	COLEC12	Collectin-12 OS=Homo sapiens OX=9606 GN=COLEC12	0.463	0.00154492
Q8NCF5	NFATC2IP	NFATC2-interacting protein OS=Homo sapiens OX=9606 GN=NFATC2IP	0.463	7.6557E-05
Q9Y4C2	TCAF1	TRPM8 channel-associated factor 1 OS=Homo sapiens OX=9606 GN=TCAF1	0.463	3.9918E-05

O00264	PGRMC1	Membrane-associated progesterone receptor component 1 OS=Homo sapiens OX=9606 GN=PGRMC1	0.462	0.00014136
P62837	UBE2D2	Ubiquitin-conjugating enzyme E2 D2 OS=Homo sapiens OX=9606 GN=UBE2D2	0.462	1.5171E-05
Q15928	ZNF141	Zinc finger protein 141 OS=Homo sapiens OX=9606 GN=ZNF141	0.46	0.0184389
Q6NXE6	ARMC6	Armadillo repeat-containing protein 6 OS=Homo sapiens OX=9606 GN=ARMC6	0.458	3.7822E-05
Q6NW29	RWDD4	RWD domain-containing protein 4 OS=Homo sapiens OX=9606 GN=RWDD4	0.457	0.000176
Q9P0P0	RNF181	E3 ubiquitin-protein ligase RNF181 OS=Homo sapiens OX=9606 GN=RNF181	0.453	4.4311E-05
P98179	RBM3	RNA-binding protein 3 OS=Homo sapiens OX=9606 GN=RBM3	0.449	0.0008212
Q96B01	RAD51AP1	RAD51-associated protein 1 OS=Homo sapiens OX=9606 GN=RAD51AP1	0.448	0.004341
Q15555	MAPRE2	Microtubule-associated protein RP/EB family member 2 OS=Homo sapiens OX=9606 GN=MAPRE2	0.441	1.5561E-05
Q8NHU6	TDRD7	Tudor domain-containing protein 7 OS=Homo sapiens OX=9606 GN=TDRD7	0.438	0.00012284
Q9GZQ8	MAP1LC3B	Microtubule-associated proteins 1A/1B light chain 3B OS=Homo sapiens OX=9606 GN=MAP1LC3B	0.437	0.00046407
O14558	HSPB6	Heat shock protein beta-6 OS=Homo sapiens OX=9606 GN=HSPB6	0.436	0.0001247
Q9NZE8	MRPL35	"39S ribosomal protein L35, mitochondrial OS=Homo sapiens OX=9606 GN=MRPL35"	0.434	7.9993E-05
Q15004	PCLAF	PCNA-associated factor OS=Homo sapiens OX=9606 GN=PCLAF	0.427	7.5564E-05
Q99741	CDC6	Cell division control protein 6 homolog OS=Homo sapiens OX=9606 GN=CDC6	0.423	0.00060302
Q8IYJ1	CPNE9	Copine-9 OS=Homo sapiens OX=9606 GN=CPNE9	0.413	0.00021933
P62875	POLR2L	"DNA-directed RNA polymerases I, II, and III subunit RPABC5 OS=Homo sapiens OX=9606 GN=POLR2L"	0.409	1.6134E-05
O75794	CDC123	Cell division cycle protein 123 homolog OS=Homo sapiens OX=9606 GN=CDC123	0.408	0.00001895
Q16658	FSCN1	Fascin OS=Homo sapiens OX=9606 GN=FSCN1	0.408	3.466E-07
Q9H8U3	ZFAND3	AN1-type zinc finger protein 3 OS=Homo sapiens OX=9606 GN=ZFAND3	0.396	0.00015539
P50914	RPL14	60S ribosomal protein L14 OS=Homo sapiens OX=9606 GN=RPL14	0.392	0.029176
Q01826	SATB1	DNA-binding protein SATB1 OS=Homo sapiens OX=9606 GN=SATB1	0.386	5.7836E-05
Q4KWH8	PLCH1	"1-phosphatidylinositol 4,5-bisphosphate phosphodiesterase eta-1 OS=Homo sapiens OX=9606 GN=PLCH1"	0.384	2.0999E-05
P18124	RPL7	60S ribosomal protein L7 OS=Homo sapiens	0.382	2.0517E-06

		OX=9606 GN=RPL7		
P54826	GAS1	Growth arrest-specific protein 1 OS=Homo sapiens OX=9606 GN=GAS1	0.381	1.8762E-05
P33552	CKS2	Cyclin-dependent kinases regulatory subunit 2 OS=Homo sapiens OX=9606 GN=CKS2	0.376	0.002444
Q71DI3	HIST2H3A	Histone H3.2 OS=Homo sapiens OX=9606 GN=HIST2H3A	0.373	0.025423
Q96EL2	MRPS24	"28S ribosomal protein S24, mitochondrial OS=Homo sapiens OX=9606 GN=MRPS24"	0.369	5.5184E-05
Q96GN5	CDCA7L	Cell division cycle-associated 7-like protein OS=Homo sapiens OX=9606 GN=CDCA7L	0.365	0.00055526
Q01581	HMGCS1	"Hydroxymethylglutaryl-CoA synthase, cytoplasmic OS=Homo sapiens OX=9606 GN=HMGCS1"	0.36	8.8503E-07
P10412	HIST1H1E	Histone H1.4 OS=Homo sapiens OX=9606 GN=HIST1H1E	0.357	0.0168012
P60520	GABARAPL2	Gamma-aminobutyric acid receptor-associated protein-like 2 OS=Homo sapiens OX=9606 GN=GABARAPL2	0.346	2.0653E-05
O95857	TSPAN13	Tetraspanin-13 OS=Homo sapiens OX=9606 GN=TSPAN13	0.341	0.00162411
O43924	PDE6D	"Retinal rod rhodopsin-sensitive cGMP 3',5'-cyclic phosphodiesterase subunit delta OS=Homo sapiens OX=9606 GN=PDE6D"	0.34	5.2687E-07
Q96JG8	MAGED4	Melanoma-associated antigen D4 OS=Homo sapiens OX=9606 GN=MAGED4	0.339	0.0088431
A4UGR9	XIRP2	Xin actin-binding repeat-containing protein 2 OS=Homo sapiens OX=9606 GN=XIRP2	0.334	0.00026041
Q9BWT1	CDCA7	Cell division cycle-associated protein 7 OS=Homo sapiens OX=9606 GN=CDCA7	0.323	0.0022605
Q15048	LRRC14	Leucine-rich repeat-containing protein 14 OS=Homo sapiens OX=9606 GN=LRRC14	0.315	0.00013847
Q9NZJ9	NUDT4	Diphosphoinositol polyphosphate phosphohydrolase 2 OS=Homo sapiens OX=9606 GN=NUDT4	0.301	1.9926E-07
O95149	SNUPN	Snurportin-1 OS=Homo sapiens OX=9606 GN=SNUPN	0.254	0.00158395
Q9Y291	MRPS33	"28S ribosomal protein S33, mitochondrial OS=Homo sapiens OX=9606 GN=MRPS33"	0.253	0.00011942
Q8IUR0	TRAPPC5	Trafficking protein particle complex subunit 5 OS=Homo sapiens OX=9606 GN=TRAPPC5	0.219	1.8411E-07

Table S4 Differentially expressed proteins in 22Rv1 enzDTP cells

Table S5. Up-regulated proteins and corresponding drugs

Gene	Drug
PCCB	Biotin, L-Valine
ADRA2A	Amitriptyline, Amoxapine, Amoxapine, Amphetamine, Apomorphine, Apraclonidine, Aripiprazole, Aripiprazole lauroxil, Asenapine, Benzphetamine,

	Bethanidine, Brimonidine, Bromocriptine, Cabergoline, Carvedilol, Celiprolol, Chlorpromazine, Clonidine, Clozapine, Desipramine, Dexmedetomidine, Dihydroergocornine, Dihydroergocristine, Dihydroergotamine, Dipivefrin, DL-Methylephedrine, Dosulepin, Doxepin, Dronedarone, Droxidopa, Epinastine, Epinephrine, Ergoloid mesylate, Ergotamine, Fenoldopam, Guanabenz, Guanfacine, Indigotindisulfonic acid, Lamotrigine, Levonordefrin, Lisuride, Lofexidine, Lorpiprazole, Loxapine, Lurasidone, Maprotiline, Mephentermine, Metamfetamine, Methotrimeprazine, Methyldopa, Mianserin, Mirtazapine, Moxisylyte, Moxonidine, Naphazoline, Nefazodone, Norepinephrine, Nortriptyline, Olanzapine, Oxymetazoline, Paliperidone, Pergolide, Periciazine, Phenoxybenzamine, Phentolamine, Phenylpropanolamine, Pizotifen, Pramipexole, Prazosin, Pseudoephedrine, Quetiapine, Racepinephrine, Rilmenidine, Risperidone, Ropinirole, Tizanidine, Tolazoline, Trazodone, Trimipramine, Xylometazoline, Yohimbine, Ziprasidone, Zuclopenthixol
ESRRA	Diethylstilbestrol, Flavone
F10	Albutrepenonacog alfa, Antihemophilic factor, Anti-inhibitor coagulant complex, Antithrombin Alfa, Apixaban, Bemiparin, Betrixaban, Coagulation Factor IX, Coagulation factor VII, Coagulation factor VIIa, Edoxaban, Emicizumab, Enoxaparin, Fondaparinux, Heparin, Kappadione, Lonoctocog alfa, Menadione, Moroctocog alfa, Nonacog beta pegol, Protamine sulfate, Protein S, Rivaroxaban, Turoctocog alfa, Turoctocog alfa pegol
CYP2C8	Isoniazid, Palmitic Acid
ALDH5A1	Chlormerodrin, NADH, Succinic acid, Valproic acid
SUCLG2	Succinic acid
PLOD3	Ascorbic acid, Succinic acid
OXCT1	Succinic acid
HSD11B2	Fluoxymesterone, Hydrocortisone, Hydrocortisone acetate, Hydrocortisone butyrate, Hydrocortisone cypionate, Hydrocortisone phosphate, Hydrocortisone probutate, Hydrocortisone valerate, NADH
FDX1	Mitotane
MAOB	Amphetamine, Flavin adenine dinucleotide, Isocarboxazid, Metamfetamine, Moclobemide, Nialamide, Nomifensine, Pargyline, Phenelzine, Phentermine, Pioglitazone, Procaine, Procarbazine, Rasagiline, Safinamide, Selegiline, Tranylcypromine, Zimelidine, Zonisamide
ACADSB	Isoleucine, Valproic acid
ABAT	Glutamic acid, L-Alanine, Phenelzine, Pyridoxal phosphate, Pyruvic acid, Vigabatrin, Valproic acid
SLC1A4	L-Alanine
MYLK	Fostamatinib
ERN1	Fostamatinib
NUAK2	Fostamatinib
CTSS	Fostamatinib
LRRK2	Fostamatinib
EPHA7	Fostamatinib
HCK	Bosutinib, Fostamatinib
FAAH	Acetaminophen, Fostamatinib, Thiopental
LIMK1	Dabrafenib, Fostamatinib
MAP3K1	Binimetinib, Fostamatinib
CAMK2D	Fostamatinib
AKR1C3	Bimatoprost, Flufenamic acid, Indometacin, NADH, Rutin
ACAA1	Trimetazidine
MAOA	Amphetamine, Flavin adenine dinucleotide, Isocarboxazid, Metamfetamine,

	Minaprine, Moclobemide, Nialamide, Nomifensine, Pargyline, Phenelzine, Phentermine, Procaine, Procarbazine, Selegiline, Tranylcypromine, Zimelidine, Zonisamide
PSMB9	Carfilzomib
MT-CO2	Cholic acid
CES1	Cholic acid, Cocaine, Cyclandelate, Dextropropoxyphene, Diamorphine, Levocarnitine, Meperidine, Naloxone, Oseltamivir, Probucol, Tacrine
COX6C	Cholic Acid
COX7C	Cholic Acid
COX6B1	Cholic Acid
EPX	Melatonin
OGDH	NADH, Valproic acid, Xanthinol
HDAC4	Belinostat, Panobinostat, Romidepsin, Zinc, Zinc acetate, Zinc chloride
GSTK1	Glutathione
MGST3	Glutathione
GPX1	Glutathione
GSTT1	Glutathione
GPX4	Glutathione
AKR1C1	Acetylsalicylic acid, NADH, Salicylic acid
NPR1	Amyl nitrite, Erythrityl tetranitrate, Isosorbide dinitrate, Nesiritide, Nitroglycerin, Nitroprusside
CAT	Fomepizole
PCCA	Biotin
MCCC1	Biotin
CA12	Acetazolamide, Benzthiazide, Hydroflumethiazide, Zonisamide
NOS3	Apremilast, L-Arginine, L-Citrulline, Miconazole, Sapropterin
ARG2	L-Arginine, Ornithine
ASL	L-Arginine
SLC7A11	Acetylcysteine, Cystine, Glutamic acid, Riluzole, Sulfasalazine, Thimerosal
ACAT1	Sulfasalazine
CD55	Chloramphenicol
PRDX5	Auranofin, Benzoic acid
SLCO1B1	Clarithromycin, Rifampicin, Telaprevir
HABP2	Hyaluronic acid
KYAT1	Pyridoxal phosphate
SHMT2	Glycine, Pyridoxal phosphate
SDSL	Pyridoxal phosphate
CTH	L-Cysteine, Pyridoxal phosphate
CPS1	Carglumic acid
GLUD1	Aluminum chloride, Glutamic acid, Hexachlorophene, NADH
HEXB	Pyrimethamine
SRD5A3	Spironolactone
BAAT	Glycine
GLYATL1	Glycine
DHRS3	Vitamin A
APOD	Copper, Vitamin A
ALDH2	Disulfiram, Guanidine, NADH
NR1H3	Diacerein
CYP2D6	Diacerein
NR1H2	Diacerein
ATP1A3	Ouabain

Table S5 Up-regulated proteins and corresponding drugs

Table S6. Primer sequences

Quantitative-PCR primers	
Name of Gene	Primer (5’ to 3’)
AR-FL-F	ACATCAAGGAACTCGATCGTATCATTGC
AR-FL-R	TTGGGCACTTGCACAGAGAT
PSA-F	GGTGACCAAGTTCATGCTGTG
PSA-R	GTGTCCTTGATCCACTTCCG
TMPRSS2-F	CTGGTGGCTGATAGGGGATA
TMPRSS2-R	GGACAAGGGGTTAGGGAGAG
AR-V7-F	CCATCTTGTCGTCTTCGGAAATGTTATGAAGC
AR-V7-R	TTTGAATGAGGCAAGTCAGCCTTTCT
UBE2C-F	TGGTCTGCCCTGTATGATGT
UBE2C-R	AAAAGCTGTGGGGTTTTTCC
CDC20-F	CGGAAGACCTGCCGTTACATTC
CDC20-R	CAGAGCTTGCACTCCACAGGTA
C-MYC-F	CCTGGTGCTCCATGAGGAGAC
C-MYC-R	CAGACTCTGACCTTTTGCCAGG
AKT1-F	TGGACTACCTGCACTCGGAGAA
AKT1-R	GTGCCGCAAAAGGTCTTCATGG
GAPDH-F	ACCCAGAAGACTGTGGATGG
GAPDH-R	TTCAGCTCAGGGATGACCTT
**Androgen receptor PCR primers**	
Name of Gene	Primer (5’ to 3’)
AR-F1	ATGGAAGTGCAGTTAGGGCTGGGAAGGGTCTACCCTCGGCCGCCGTCCAAGACCTAC
AR-R1	GATGCTCCAACGCCTCCACACCCAGGCCCATGGACACCGACACTGCCTTAC
AR-F2	TGTAAGGCAGTGTCGGTGTCCATGGGCCTGGGTGTGGAGGCGTTGGAGCATC
AR-R2	GTCCCCATAGCGGCACTGCGCCGCCGCAGCCGCCCAGGCGCTGCCGTAG
AR-F3	ATCAAGCTGGAGAACCCGCTGGACTA
AR-R3	AGATGGGCTGACATTCATAGCCTTCAAT
AR-F4	TGAGGAGACAACCCAGAAGCTGACAGTGTCACACATTGAAGGCTATG
AR-R4	TCACTGGGTGTGGAAATAGATGGGCTTGACTTTCCCAGAAAGGATCTTGG
**sgRNA for the generation of knockout PRDX5 cell line**	
Name of Gene	Primer (5’ to 3’)
sgRNA1	ATAGCCCGCTGAGCGTCTCAGGG
sgRNA2	CAGCGGCTCTGCTGAAACTGCGG

Table S6 Primer sequences

L-EPI			L-ENZ			2-EPI			2-ENZ			L-EPI & L-ENZ			L
Accession	Gene name	Ratio	Accession	Gene name	Ratio	Accession	Gene name	Ratio	Accession	Gene name	Ratio	Accession	Gene name	Ratio	Aen
A0A0B4J266	TRAV41	2.848	A0A0B4J2F0	PIGBOS1	2.131	O00458	IFRD1	2.626	P11169	SLC2A3	2.218	A0A0U1RRL7	MMP24OS	3.344	C4
A2RUB1	MEIOC	2.762	A4UGR9	XIRP2	2.487	O00519	FAAH	2.036	P22570	FDXR	2.449	A6NJG6	ARGFX	2.184	P0
A6NNW6	ENO4	2.559	O00217	NDUFS8	2.288	O14841	OPLAH	2.633	P25445	FAS	2.566	A8MSI8	LYRM9	2.657	Q0
B2RUZ4	SMIM1	2.432	O00483	NDUFA4	2.14	O15240	VGF	2.154	P32929	CTH	2.245	O14773	TPP1	2.795	Q7
O00116	AGPS	2.118	O14548	COX7A2L	2.229	O15321	TM9SF1	2.354	P98160	HSPG2	2.254	O14832	PHYH	2.366	Q7
O00391	QSOX1	3.127	O14598	VCY	2.721	O43189	PHF1	2.295	Q00987	MDM2	2.836	O75363	BCAS1	2.283	QG
O14513	NCKAP5	2.188	O14879	IFIT3	2.855	O43570	CA12	2.632	Q01650	SLC7A5	2.713	O75445	USH2A	3.05	QC7
O14529	CUX2	2.165	O14949	UQCRQ	2.214	O43688	PLPP2	2.053	Q01831	XPC	2.11	O75911	DHRS3	2.58	QS
O14975	SLC27A2	2.044	O15230	LAMA5	2.054	O43822	C21orf2	2.091	Q08431	MFGE8	2.202	O95858	TSPAN15	2.755	QZ
O15320	CTAGE5	2.2	O15394	NCAM2	2.475	O60568	PLOD3	2.497	Q13563	PKD2	2.113	P00973	OAS1	2.114	QZ
O43795	MYO1B	2.054	O43181	NDUFS4	2.428	O60701	UGDH	2.158	Q53FA7	TP53I3	5.227	P04179	SOD2	4.176	Q3
O60437	PPL	2.146	O43299	AP5Z1	3.499	O60831	PRAF2	2.159	Q5TF21	SOGA3	2.016	P05165	PCCA	2.351	QB
O60711	LPXN	2.593	O43676	NDUFB3	2.067	O75764	TCEA3	2.062	Q5VTY9	HHAT	2.738	P05166	PCCB	2.317	QP

O75110	ATP9A	2.136	O43677	NDUFC1	2.548	O94766	B3GAT3	2.049	Q68D85	NCR3LG1	2.171	P06865	HEXA	2.283	Q[illegible] Q[illegible]
O75891	ALDH1L1	2.537	O43808	SLC25A17	2.612	O94819	KBTBD11	2.165	Q7Z5Q5	POLN	2.197	P07099	EPHX1	2.517	Q[illegible] 3[illegible]
O94933	SLITRK3	2.199	O60237	PPP1R12B	2.84	O95359	TACC2	2.61	Q8IZT6	ASPM	2.357	P07339	CTSD	2.292	
O94986	CEP152	2.132	O75192	PEX11A	3.184	O96011	PEX11B	2.108	Q8N431	RASGEF1C	4.297	P07602	PSAP	2.148	
O95210	STBD1	2.433	O75298	RTN2	2.366	P03923	MT-ND6	2.703	Q8NEZ4	KMT2C	2.075	P07858	CTSB	2.374	
O95399	UTS2	4.439	O75380	NDUFS6	2.112	P04424	ASL	2.488	Q92597	NDRG1	2.43	P08913	ADRA2A	2.328	
O95425	SVIL	2.356	O75489	NDUFS3	2.078	P04733	MT1F	6.864	Q93096	PTP4A1	2.09	P09110	ACAA1	2.048	
O95613	PCNT	2.871	O75509	TNFRSF21	3.307	P07919	UQCRH	2.393	Q96NL8	C8orf37	2.01	P11532	DMD	2.394	
P00742	F10	3.009	O75795	UGT2B17	2.936	P0DMV2	CT45A9	2.634	Q96SQ9	CYP2S1	2.059	P13501	CCL5	4.41	
P02511	CRYAB	2.514	O75955	FLOT1	2.027	P10635	CYP2D6	4.399	Q99541	PLIN2	3.46	P14406	COX7A2	2.083	
P05783	KRT18	2.202	O76039	CDKL5	2.095	P12830	CDH1	2.209	Q99611	SEPHS2	2.011	P14543	NID1	2.111	
P06396	GSN	2.97	O76062	TM7SF2	3.135	P13637	ATP1A3	2.291	Q9BQ04	RBM4B	2.241	P14784	IL2RB	2.562	
P07305	H1F0	2.416	O95167	NDUFA3	2.594	P16422	EPCAM	3.147	Q9BU23	LMF2	2.009	P15289	ARSA	2.534	
P07686	HEXB	2.142	O95169	NDUFB8	2.242	P18084	ITGB5	2.21	Q9BV19	C1orf50	2.068	P17693	HLA-G	5.743	
P08670	VIM	4.503	O95299	NDUFA10	2.19	P19634	SLC9A1	2.515	Q9BZC7	ABCA2	2.477	P18463	HLA-B	9.176	
P09493	TPM1	2.371	P00367	GLUD1	2.293	P21741	MDK	2.46	Q9GZX9	TWSG1	2.001	P20020	ATP2B1	2.044	
P0C221	CCDC175	2.949	P00403	MT-CO2	2.311	P30043	BLVRB	2.171	Q9NXG6	P4HTM	2.147	P22307	SCP2	2.042	
P10242	MYB	2.72	P01833	PIGR	2.356	P30711	GSTT1	2.358	Q9UN30	SCML1	2.591	P25774	CTSS	5.768	

P10301	RRAS	2.491	P04066	FUCA1	2.392	P31949	S100A11	2.043	Q9Y253	POLH	2.681	P28288	ABCD3	2.367
P10321	HLA-C	2.571	P05091	ALDH2	3.331	P34897	SHMT2	2.333				P28328	PEX2	2.028
P10619	CTSA	2.341	P08473	MME	2.089	P36969	GPX4	2.023				P31327	CPS1	2.542
P10909	CLU	3.675	P09669	COX6C	2.05	P37268	FDFT1	2.019				P31431	SDC4	2.09
P11021	HSPA5	3.471	P09913	IFIT2	2.551	P42226	STAT6	2.678				P35475	IDUA	2.038
P11117	ACP2	2.18	P09914	IFIT1	2.367	P46527	CDKN1B	2.679				P39210	MPV17	2.387
P13533	MYH6	2.477	P0DPI2	GATD3A	2.331	P55055	NR1H2	2.064				P40939	HADHA	2.252
P15260	IFNGR1	2.157	P10109	FDX1	2.043	P55317	FOXA1	3.044				P46821	MAP1B	2.523
P15586	GNS	2.578	P11182	DBT	2.245	P55809	OXCT1	3.136				P51649	ALDH5A1	2.028
P16144	ITGB4	2.019	P11234	RALB	2.23	P55822	SH3BGR	7.264				P55073	DIO3	8.605
P17301	ITGA2	2.254	P11310	ACADM	2.03	P78358	CTAG1A	2.484				P55084	HADHB	2.32
P19087	GNAT2	2.33	P11678	EPX	4.177	P80297	MT1X	6.526				P56277	CMC4	2.046
P19256	CD58	2.088	P12074	COX6A1	2.27	Q01415	GALK2	2.119				P58004	SESN2	3.118
P23497	SP100	2.453	P12235	SLC25A4	2.03	Q02218	OGDH	2.032				P61916	NPC2	2.043
P26006	ITGA3	2.504	P14854	COX6B1	2.063	Q04609	FOLH1	2.274				P78556	CCL20	3.24
P26885	FKBP2	2.063	P15309	ACPP	2.539	Q06210	GFPT1	2.165				P80303	NUCB2	2.012
P28065	PSMB9	2.246	P15954	COX7C	2.218	Q11201	ST3GAL1	2.242				P80365	HSD11B2	2.693
P28289	TMOD1	2.62	P16066	NPR1	4.162	Q12913	PTPRJ	2.351				P83111	LACTB	2.1
P28799	GRN	2.078	P17568	NDUFB7	2.212	Q13367	AP3B2	2.021				Q01459	CTBS	2.216

P29474	NOS3	4.464	P21953	BCKDHB	2.03	Q13451	FKBP5	2.076	Q0ZGT2	NEXN	6.886
P30044	PRDX5	2.134	P23141	CES1	2.012	Q13557	CAMK2D	2.248	Q13510	ASAH1	2.296
P30455	HLA-A	2.276	P23786	CPT2	2.044	Q13795	ARFRP1	2.061	Q13825	AUH	2.03
P31512	FMO4	2.692	P24752	ACAT1	2.472	Q14114	LRP8	2.831	Q14032	BAAT	4.593
P32455	GBP1	2.629	P27338	MAOB	2.444	Q14376	GALE	2.354	Q15714	TSC22D1	3.3
P38646	HSPA9	2.005	P27701	CD82	2.548	Q14554	PDIA5	2.083	Q4G0N4	NADK2	2.109
P38935	IGHMBP2	3.412	P41247	PNPLA4	2.062	Q14693	LPIN1	2.016	Q5JTV8	TOR1AIP1	2.493
P39060	COL18A1	2.247	P43251	BTD	2.719	Q15005	SPCS2	2.054	Q5T2T1	MPP7	2.106
P42330	AKR1C3	3.15	P45954	ACADSB	2.182	Q15526	SURF1	2.072	Q5T890	ERCC6L2	3.457
P45877	PPIC	2.274	P50895	BCAM	2.211	Q15560	TCEA2	2.275	Q5XPI4	RNF123	2.142
P48740	MASP1	2.306	P52797	EFNA3	2.036	Q15771	RAB30	2.283	Q6NUK1	SLC25A24	2.123
P49257	LMAN1	2.064	P54868	HMGCS2	2.761	Q16623	STX1A	2.433	Q7Z3D6	DGLUCY	2.391
P51688	SGSH	2.217	P56181	NDUFV3	2.036	Q16773	KYAT1	2.221	Q8N357	SLC35F6	2.849
P53701	HCCS	2.095	P62068	USP46	5.442	Q330K2	NDUFAF6	2.369	Q8TBP5	FAM174A	2.047
P53804	TTC3	2.391	P78540	ARG2	3.637	Q3KQV9	UAP1L1	2.663	Q96A73	KIAA1191	2.617
P54707	ATP12A	2.107	P80404	ABAT	2.542	Q3MIN7	RGL3	2.448	Q96DA2	RAB39B	2.339
P61224	RAP1B	2.121	P98196	ATP11A	2.049	Q3SY69	ALDH1L2	2.869	Q96QB1	DLC1	2.517
P61225	RAP2B	2.901	P99999	CYCS	2.087	Q53FZ2	ACSM3	2.029	Q9BX68	HINT2	2.714

P61769	B2M	3.676
P67936	TPM4	2.074
P80188	LCN2	2.728
Q04828	AKR1C1	2.363
Q04837	SSBP1	2.251
Q08AE8	SPIRE1	2.436
Q08AF3	SLFN5	2.154
Q14249	ENDOG	2.15
Q14956	GPNMB	7.36
Q15067	ACOX1	2.068
Q15746	MYLK	2.02
Q16831	UPP1	4.2
Q2NL98	VMAC	2.026
Q38SD2	LRRK1	2.61
Q460N5	PARP14	2.782
Q4W5N1	ABCA11P	3.493
Q5JUK3	KCNT1	2.373
Q5T9C2	FAM102A	2.361
Q02978	SLC25A11	2.064
Q08380	LGALS3BP	2.287
Q0P6H9	TMEM62	2.091
Q12999	TSPAN31	2.084
Q13133	NR1H3	2.047
Q13232	NME3	2.166
Q13480	GAB1	2.472
Q14011	CIRBP	2.062
Q14254	FLOT2	2.131
Q15375	EPHA7	2.16
Q15646	OASL	2.799
Q16134	ETFDH	2.124
Q16625	OCLN	2.396
Q16718	NDUFA5	2.027
Q16760	DGKD	5.4
Q16836	HADH	2.121
Q3MIX3	ADCK5	2.495
Q53R41	FASTKD1	2.021
Q5RI15	COX20	2.473
Q5TGZ0	MINOS1	2.409
Q5VVW2	GARNL3	2.781
Q69YL0	NCBP2-AS2	2.107
Q6IN84	MRM1	2.261
Q6NXG1	ESRP1	2.616
Q6UW68	TMEM205	2.042
Q6UWW8	CES3	2.151
Q6ZS30	NBEAL1	2.208
Q7Z404	TMC4	2.573
Q86VU5	COMTD1	2.506
Q86WU2	LDHD	2.141
Q86YS6	RAB43	2.282
Q8IUX1	TMEM126B	2.235
Q8IVH4	MMAA	2.132
Q8IWA5	SLC44A2	2.008
Q8IWT6	LRRC8A	2.378
Q8IWZ5	TRIM42	4.424
Q9BZQ2	SHCBP1L	2.39
Q9C0D2	CEP295	2.174
Q9NUT2	ABCB8	2.282
Q9P246	STIM2	2.911
Q9UDR5	AASS	2.502
Q9UFN0	NIPSNAP3A	2.301
Q9UIJ7	AK3	2.176
Q9ULM6	CNOT6	2.438
Q9Y2Q3	GSTK1	2.423
Q9Y5F3	PCDHB1	3.011
Q9Y646	CPQ	2.347
Q9Y6N5	SQOR	2.369

Q5U649	C12orf60	2.645	Q5SVS4	SLC25A30	2.061	Q8IY17	PNPLA6	2.067
Q68CR1	SEL1L3	2.005	Q5SWW7	C10orf55	2.741	Q8IZD9	DOCK3	2.271
Q6EKJ0	GTF2IRD2B	2.272	Q5TCQ9	MAGI3	2.413	Q8IZQ5	SELENOH	2.064
Q6IC98	GRAMD4	2.069	Q5U623	ATF7IP2	3.138	Q8N398	VWA5B2	2.152
Q6IPR1	ETFRF1	3.396	Q68CQ7	GLT8D1	2.084	Q8N490	PNKD	2.457
Q6PI78	TMEM65	2.409	Q6DKK2	TTC19	2.307	Q8N4S9	MARVELD2	2.53
Q6UWY5	OLFML1	2.452	Q6NVY1	HIBCH	2.127	Q8N5G0	SMIM20	2.111
Q6ZSS7	MFSD6	2.049	Q6P1Q0	LETMD1	2.058	Q8N8Q8	COX18	2.169
Q71SY5	MED25	2.498	Q6UWH4	FAM198B	2.417	Q8ND25	ZNRF1	2.291
Q86SR1	GALNT10	2.593	Q6UXG2	KIAA1324	2.771	Q8NDH3	NPEPL1	3.253
Q86T03	PIP4P1	2.018	Q6YN16	HSDL2	2.175	Q8NEG4	FAM83F	2.205
Q86TM3	DDX53	2.8	Q7Z591	AKNA	2.25	Q8NFU3	TSTD1	2.352
Q86WC4	OSTM1	2.12	Q86SK9	SCD5	2.293	Q8TAA5	GRPEL2	2.322
Q8IXB1	DNAJC10	2.145	Q86XE3	MICU3	2.149	Q8TBP6	SLC25A40	2.048
Q8IYJ3	SYTL1	2.381	Q86Y39	NDUFA11	2.012	Q8TCD1	C18orf32	2.18
Q8IZ41	RASEF	2.276	Q8IXI2	RHOT1	2.05	Q8TD43	TRPM4	2.95
Q8N4N3	KLHL36	2.896	Q8IY21	DDX60	2.196	Q8TEK3	DOT1L	2.367
Q8N8R3	SLC25A29	2.087	Q8N0X4	CLYBL	2.098	Q8WY54	PPM1E	2.083

Q8N9Z2	CCDC71L	2.604	Q8N283	ANKRD35	2.398	Q92504	SLC39A7	2.283
Q8NA47	CCDC63	2.361	Q8N2F6	ARMC10	2.065	Q969I3	GLYATL1	3.558
Q8NEV8	EXPH5	2.596	Q8N5M1	ATPAF2	2.06	Q969X5	ERGIC1	2.12
Q8TA86	RP9	4.391	Q8NAN2	MIGA1	2.315	Q96CF2	CHMP4C	2.39
Q8TBM8	DNAJB14	2.113	Q8NBX0	SCCPDH	2.242	Q96D70	R3HDM4	2.009
Q8WU67	ABHD3	2.303	Q8NF91	SYNE1	2.147	Q96DT5	DNAH11	2.173
Q92574	TSC1	2.26	Q8NFW9	MYRIP	2.254	Q96GA7	SDSL	3.115
Q92953	KCNB2	2.006	Q8TBC5	ZSCAN18	2.007	Q96HD1	CRELD1	2.354
Q93070	ART4	2.278	Q8TDR0	TRAF3IP1	2.244	Q96HR9	REEP6	2.254
Q969M3	YIPF5	2.117	Q8WXG1	RSAD2	2.289	Q96JQ2	CLMN	2.191
Q96AQ6	PBXIP1	2.313	Q92508	PIEZO1	2.12	Q96MH6	TMEM68	2.393
Q96CV9	OPTN	2.243	Q96BJ3	AIDA	2.113	Q96RQ3	MCCC1	2.022
Q96FN4	CPNE2	2.838	Q96CM8	ACSF2	2.478	Q99424	ACOX2	2.251
Q96FZ7	CHMP6	2.326	Q96FE7	PIK3IP1	2.185	Q99801	NKX3-1	2.23
Q96HE7	ERO1A	2.876	Q96HA9	PEX11G	2.076	Q99828	CIB1	2.066
Q96HH9	GRAMD2B	2.203	Q96HJ9	FMC1	2.057	Q9BRS8	LARP6	2.301
Q96QI5	HS3ST6	2.413	Q96I99	SUCLG2	2.069	Q9BSJ8	ESYT1	2.159
Q99519	NEU1	2.018	Q96LT4	SAMD8	2.456	Q9BX66	SORBS1	2.697

Q99536	VAT1	2.484	Q96PE7	MCEE	2.043	Q9BYN0	SRXN1	2.236
Q99685	MGLL	3.528	Q96PP9	GBP4	2.196	Q9BZV1	UBXN6	2.103
Q9BQE4	SELENOS	2.011	Q96S16	JMJD8	3.339	Q9GZY8	MFF	2.143
Q9BRK4	LZTS2	2.11	Q99798	ACO2	2.006	Q9H0Q0	FAM49A	2.086
Q9BRT2	UQCC2	2.018	Q99985	SEMA3C	2.248	Q9H115	NAPB	2.252
Q9BSH5	HDHD3	2.322	Q9BV79	MECR	2.001	Q9H147	DNTTIP1	2.003
Q9BXK5	BCL2L13	2.223	Q9BWT3	PAPOLG	9.264	Q9H6S3	EPS8L2	2.18
Q9BY50	SEC11C	2.679	Q9C004	SPRY4	3.248	Q9H8H3	METTL7A	2.052
Q9H093	NUAK2	2.721	Q9GZN7	ROGDI	2.089	Q9H8P0	SRD5A3	2.096
Q9H190	SDCBP2	2.78	Q9H2U2	PPA2	2.309	Q9NPR9	GPR108	2.169
Q9H930	SP140L	2.445	Q9HBA9	FOLH1B	2.699	Q9NQ86	TRIM36	2.259
Q9HCH5	SYTL2	2.161	Q9HBL7	PLGRKT	2.784	Q9NUI1	DECR2	2.298
Q9HD67	MYO10	2.295	Q9NTG7	SIRT3	2.134	Q9NZ45	CISD1	2.203
Q9NRI5	DISC1	2.728	Q9NUJ1	ABHD10	2.235	Q9P2J8	ZNF624	2.445
Q9NRM1	ENAM	2.33	Q9NUM3	SLC39A9	2.147	Q9UBL6	CPNE7	2.981
Q9NSD4	ZNF275	2.25	Q9NVA1	UQCC1	2.057	Q9UDW3	ZMAT5	2.254
Q9NTX5	ECHDC1	2.225	Q9NXF8	ZDHHC7	2.153	Q9UHI8	ADAMTS1	2.221
Q9NUM4	TMEM106B	2.399	Q9NZJ7	MTCH1	2.774	Q9UHK6	AMACR	2.08
Q9NUQ2	AGPAT5	2.927	Q9P032	NDUFAF4	2.394	Q9UI15	TAGLN3	2.07

Q9NZ08	ERAP1	2.933	Q9P0J0	NDUFA13	2.032	Q9UI32	GLS2	2.612
Q9NZC3	GDE1	2.175	Q9P2K5	MYEF2	2.021	Q9UKU0	ACSL6	2.018
Q9UBS3	DNAJB9	3.091	Q9UBK9	UXT	2.242	Q9UMF0	ICAM5	3.195
Q9UG56	PISD	2.115	Q9UEY8	ADD3	2.028	Q9UPA5	BSN	2.776
Q9UHN6	TMEM2	2.558	Q9UII2	ATP5IF1	2.585	Q9UPQ0	LIMCH1	2.292
Q9UHQ4	BCAP29	2.036	Q9UII4	HERC5	2.35	Q9UPT6	MAPK8IP3	2.666
Q9UJA9	ENPP5	2.392	Q9UJ14	GGT7	2.112	Q9UQ49	NEU3	2.728
Q9UJF2	RASAL2	3.244	Q9UJ83	HACL1	2.44	Q9Y210	TRPC6	2.133
Q9UJY1	HSPB8	2.665	Q9UKG9	CROT	2.035	Q9Y252	RNF6	2.01
Q9ULV0	MYO5B	2.444	Q9UKU7	ACAD8	2.006	Q9Y2H5	PLEKHA6	2.385
Q9UM22	EPDR1	2.438	Q9UL16	CFAP45	2.017	Q9Y2T7	YBX2	2.081
Q9UMX3	BOK	2.378	Q9UPS8	ANKRD26	4.499	Q9Y6M5	SLC30A1	2.473
Q9UNT1	RAB2B	2.597	Q9Y512	SAMM50	2.314			
Q9Y305	ACOT9	2.021	Q9Y639	NPTN	2.56			
Q9Y4L1	HYOU1	2.09	Q9Y6M9	NDUFB9	2.035			
Q9Y623	MYH4	2.438						
Q9Y6L6	SLCO1B1	2.108						

Data 1 Venn diagram of upregulated proteins

L-EPI			L-ENZ			2-EPI			2-ENZ			L-EPI & L-ENZ		
Accession	Gene name	Ratio	Accession	Gene name	Ratio	Accession	Gene name	Ratio	Accession	Gene name	Ratio	Accession	Gene name	R

O00244	ATOX1	0.435	O00625	PIR	0.454	O00470	MEIS1	0.456	O14558	HSPB6	0.436	A6NDG6	PGP	[illegible]
O00505	KPNA3	0.464	O15067	PFAS	0.468	O14654	IRS4	0.498	O75935	DCTN3	0.491	O00154	ACOT7	[illegible]
O00506	STK25	0.477	O15305	PMM2	0.421	O14786	NRP1	0.338	O95989	NUDT3	0.471	O14732	IMPA2	[illegible]
O00767	SCD	0.399	O60333	KIF1B	0.485	O43524	FOXO3	0.469	P0DI82	TRAPPC2B	0.47	O14737	PDCD5	[illegible]
O14494	PLPP1	0.445	O76003	GLRX3	0.493	O60911	CTSV	0.417	P0DPI3	CENPVL2	0.48	O14777	NDC80	[illegible]
O14530	TXNDC9	0.496	O94804	STK10	0.375	O95319	CELF2	0.482	P30154	PPP2R1B	0.478	O14965	AURKA	[illegible]
O14757	CHEK1	0.422	O94851	MICAL2	0.465	O95619	YEATS4	0.485	P84101	SERF2	0.478	O15318	POLR3G	[illegible]
O14787	TNPO2	0.477	O94915	FRYL	0.453	P00918	CA2	0.441	Q15048	LRRC14	0.315	O43663	PRC1	[illegible]
O43683	BUB1	0.475	O95801	TTC4	0.433	P07864	LDHC	0.495	Q6NW29	RWDD4	0.457	O43692	PI15	[illegible]
O75131	CPNE3	0.448	O95817	BAG3	0.448	P09455	RBP1	0.471	Q86X27	RALGPS2	0.469	O43805	SSNA1	[illegible]
O75153	CLUH	0.479	P00491	PNP	0.466	P15121	AKR1B1	0.463	Q8NCF5	NFATC2IP	0.463	O43847	NRDC	[illegible]
O75179	ANKRD17	0.451	P04083	ANXA1	0.477	P17482	HOXB9	0.485	Q9GZQ8	MAP1LC3B	0.437	O60566	BUB1B	[illegible]
O75223	GGCT	0.487	P06733	ENO1	0.482	P17612	PRKACA	0.465	Q9HCP0	CSNK1G1	0.469	O75310	UGT2B11	[illegible]
O75821	EIF3G	0.496	P15170	GSPT1	0.469	P23458	JAK1	0.438	Q9NZE8	MRPL35	0.434	O75330	HMMR	[illegible]
O94830	DDHD2	0.483	P16401	HIST1H1B	0.445	P36873	PPP1CC	0.487				O75347	TBCA	[illegible]
O95229	ZWINT	0.445	P17174	GOT1	0.497	P40222	TXLNA	0.463				O75419	CDC45	[illegible]
O96033	MOCS2	0.47	P21291	CSRP1	0.446	P42766	RPL35	0.474				O75449	KATNA1	[illegible]
P07288	KLK3	0.304	P22102	GART	0.444	P43694	GATA4	0.42				O75496	GMNN	[illegible]
P08237	PFKM	0.494	P30520	ADSS	0.482	P49006	MARCKSL1	0.472				O75717	WDHD1	[illegible]
P08708	RPS17	0.49	P30566	ADSL	0.478	P49207	RPL34	0.448				O95235	KIF20A	[illegible]

P12277	CKB	0.457	P31949	S100A11	0.439	P51956	NEK3	0.498	O95239	KIF4A	[illegible]
P20290	BTF3	0.491	P46734	MAP2K3	0.473	P61254	RPL26	0.461	O95347	SMC2	[illegible]
P23258	TUBG1	0.498	P46781	RPS9	0.493	P61925	PKIA	0.472	O95372	LYPLA2	[illegible]
P23588	EIF4B	0.498	P48739	PITPNB	0.491	Q02878	RPL6	0.489	O95433	AHSA1	[illegible]
P24941	CDK2	0.439	P49023	PXN	0.441	Q04724	TLE1	0.45	O95864	FADS2	[illegible]
P25098	GRK2	0.482	P50579	METAP2	0.428	Q04727	TLE4	0.353	P00338	LDHA	[illegible]
P32119	PRDX2	0.499	P50990	CCT8	0.485	Q12834	CDC20	0.496	P00374	DHFR	[illegible]
P34896	SHMT1	0.436	P53384	NUBP1	0.412	Q13043	STK4	0.461	P00492	HPRT1	[illegible]
P35251	RFC1	0.486	P62847	RPS24	0.466	Q13309	SKP2	0.477	P01130	LDLR	[illegible]
P35789	ZNF93	0.463	Q04917	YWHAH	0.495	Q15475	SIX1	0.445	P04183	TK1	[illegible]
P39019	RPS19	0.49	Q06830	PRDX1	0.477	Q5T011	SZT2	0.443	P04818	TYMS	[illegible]
P46783	RPS10	0.495	Q12982	BNIP2	0.37	Q68CZ1	RPGRIP1L	0.445	P06454	PTMA	[illegible]
P47813	EIF1AX	0.496	Q13418	ILK	0.462	Q6FIF0	ZFAND6	0.49	P06493	CDK1	[illegible]
P49454	CENPF	0.427	Q13630	TSTA3	0.448	Q6P4I2	WDR73	0.319	P06744	GPI	[illegible]
P49459	UBE2A	0.422	Q14749	GNMT	0.273	Q719H9	KCTD1	0.482	P07108	DBI	[illegible]
P52306	RAP1GDS1	0.483	Q15056	EIF4H	0.455	Q7L1Q6	BZW1	0.498	P07195	LDHB	[illegible]
P52564	MAP2K6	0.411	Q15181	PPA1	0.496	Q86SK9	SCD5	0.496	P07737	PFN1	[illegible]
P53611	RABGGTB	0.455	Q15404	RSU1	0.455	Q86WW8	COA5	0.449	P08238	HSP90AB1	[illegible]
P54792	DVL1P1	0.426	Q15418	RPS6KA1	0.446	Q8IWC1	MAP7D3	0.38	P08243	ASNS	[illegible]
P58546	MTPN	0.495	Q15431	SYCP1	0.399	Q8N4C8	MINK1	0.29	P10144	GZMB	[illegible]
P68402	PAFAH1B2	0.482	Q15642	TRIP10	0.474	Q8N5W9	RFLNB	0.489	P11388	TOP2A	[illegible]
P78330	PSPH	0.423	Q3KRA6	C2orf76	0.462	Q8NI77	KIF18A	0.438	P11586	MTHFD1	[illegible]

Q02241	KIF23	0.424
Q05639	EEF1A2	0.482
Q13070	GAGE6	0.483
Q13111	CHAF1A	0.446
Q13572	ITPK1	0.432
Q13905	RAPGEF1	0.458
Q14181	POLA2	0.48
Q14738	PPP2R5D	0.461
Q15527	SURF2	0.498
Q4V326	GAGE2E	0.436
Q5UIP0	RIF1	0.46
Q5VXD3	SAMD13	0.489
Q6GMV2	SMYD5	0.482
Q6IPR3	TYW3	0.487
Q6NS38	ALKBH2	0.494
Q6PKG0	LARP1	0.484
Q7Z7J9	CAMK2N1	0.433
Q86WQ0	NR2C2AP	0.449
Q8N806	UBR7	0.479
Q8NC51	SERBP1	0.419
Q8NG31	KNL1	0.486

Q4LDG9	DNAL1	0.478
Q5T6F2	UBAP2	0.477
Q6NYC1	JMJD6	0.471
Q6P1Q9	METTL2B	0.478
Q6PD74	AAGAB	0.495
Q7Z422	SZRD1	0.481
Q8IUE6	HIST2H2AB	0.445
Q8IXQ3	C9orf40	0.476
Q8N6N3	C1orf52	0.449
Q8ND04	SMG8	0.451
Q8TBE9	NANP	0.486
Q8TEW0	PARD3	0.475
Q8WX93	PALLD	0.49
Q8WZA0	LZIC	0.488
Q92598	HSPH1	0.437
Q969Q0	RPL36AL	0.496
Q969U7	PSMG2	0.497
Q96EB1	ELP4	0.483
Q96EK6	GNPNAT1	0.447
Q96RR4	CAMKK2	0.426
Q99471	PFDN5	0.429

Q8TED1	GPX8	0.478
Q96F45	ZNF503	0.447
Q96G61	NUDT11	0.4
Q99538	LGMN	0.356
Q99576	TSC22D3	0.404
Q9BZM4	ULBP3	0.48
Q9H2J7	SLC6A15	0.334
Q9H7C9	AAMDC	0.447
Q9H7S9	ZNF703	0.373
Q9H857	NT5DC2	0.482
Q9HBU6	ETNK1	0.464
Q9NRZ9	HELLS	0.424
Q9UBU8	MORF4L1	0.466
Q9UI36	DACH1	0.448
Q9UKE5	TNIK	0.478
Q9Y2S6	TMA7	0.47
Q9Y376	CAB39	0.464
Q9Y4R8	TELO2	0.493
Q9Y605	MRFAP1	0.48

P12004	PCNA	[illegible]
P12081	HARS	[illegible]
P13639	EEF2	[illegible]
P13693	TPT1	[illegible]
P13797	PLS3	[illegible]
P13798	APEH	[illegible]
P14324	FDPS	[illegible]
P14618	PKM	[illegible]
P14635	CCNB1	[illegible]
P16949	STMN1	[illegible]
P17812	CTPS1	[illegible]
P18858	LIG1	[illegible]
P19174	PLCG1	[illegible]
P19623	SRM	[illegible]
P20248	CCNA2	[illegible]
P20839	IMPDH1	[illegible]
P22234	PAICS	[illegible]
P23528	CFL1	[illegible]
P26358	DNMT1	[illegible]
P26639	TARS	[illegible]
P27348	YWHAQ	[illegible]

Q8TAP6	CEP76	0.435
Q8WU79	SMAP2	0.387
Q8WVB6	CHTF18	0.397
Q92569	PIK3R3	0.459
Q92995	USP13	0.419
Q969E8	TSR2	0.449
Q96DR5	BPIFA2	0.427
Q96GD4	AURKB	0.419
Q96GX2	ATXN7L3B	0.402
Q96MW1	CCDC43	0.47
Q96PM5	RCHY1	0.459
Q99584	S100A13	0.476
Q99614	TTC1	0.492
Q99801	NKX3-1	0.368
Q99961	SH3GL1	0.479
Q99986	VRK1	0.439
Q9BTE6	AARSD1	0.495
Q9BV29	CCDC32	0.433
Q9BW19	KIFC1	0.495
Q9BWG4	SSBP4	0.389
Q9BZ23	PANK2	0.453
Q9C0C9	UBE2O	0.499
Q9GZU2	PEG3	0.434

Q99543	DNAJC2	0.484
Q99733	NAP1L4	0.43
Q9BQI0	AIF1L	0.439
Q9BRP1	PDCD2L	0.454
Q9BU89	DOHH	0.493
Q9BUT9	MCRIP2	0.492
Q9BV44	THUMPD3	0.488
Q9BV86	NTMT1	0.488
Q9BVS4	RIOK2	0.447
Q9BW83	IFT27	0.456
Q9BYB4	GNB1L	0.487
Q9BZG8	DPH1	0.459
Q9C0F1	CEP44	0.463
Q9H788	SH2D4A	0.44
Q9H7L9	SUDS3	0.299
Q9H910	JPT2	0.484
Q9HBI1	PARVB	0.483
Q9HD42	CHMP1A	0.47
Q9NPA3	MID1IP1	0.45
Q9NQP4	PFDN4	0.456
Q9NR19	ACSS2	0.408
Q9NT62	ATG3	0.495
Q9NUQ8	ABCF3	0.486

P27707	DCK	[illegible]
P31350	RRM2	[illegible]
P31948	STIP1	[illegible]
P33981	TTK	[illegible]
P33992	MCM5	[illegible]
P37802	TAGLN2	[illegible]
P39748	FEN1	[illegible]
P41227	NAA10	[illegible]
P43358	MAGEA4	[illegible]
P46013	MKI67	[illegible]
P46109	CRKL	[illegible]
P49321	NASP	[illegible]
P49840	GSK3A	[illegible]
P50395	GDI2	[illegible]
P50748	KNTC1	[illegible]
P52292	KPNA2	[illegible]
P52565	ARHGDIA	[illegible]
P52732	KIF11	[illegible]
P53396	ACLY	[illegible]
P55060	CSE1L	[illegible]
P55209	NAP1L1	[illegible]
P57076	CFAP298	[illegible]
P60842	EIF4A1	[illegible]

Q9H0H5	RACGAP1	0.467
Q9H2B2	SYT4	0.451
Q9H3R5	CENPH	0.445
Q9H6D7	HAUS4	0.49
Q9H7B4	SMYD3	0.355
Q9H9T3	ELP3	0.479
Q9HBM1	SPC25	0.358
Q9NQS7	INCENP	0.37
Q9NR48	ASH1L	0.347
Q9NVI1	FANCI	0.453
Q9NVP2	ASF1B	0.425
Q9NVW2	RLIM	0.429
Q9NYP7	ELOVL5	0.417
Q9P2S5	WRAP73	0.469
Q9UBK9	UXT	0.361
Q9UBQ5	EIF3K	0.495
Q9UEU5	GAGE2D	0.499
Q9UIK5	TMEFF2	0.44
Q9ULR3	PPM1H	0.49
Q9UQR0	SCML2	0.471
Q9Y547	HSPB11	0.446
Q9Y597	KCTD3	0.459
Q9Y5A9	YTHDF2	0.493

Q9NVR0	KLHL11	0.475
Q9NW75	GPATCH2	0.465
Q9NY33	DPP3	0.48
Q9NYJ8	TAB2	0.478
Q9P0V9	SEPT10	0.498
Q9UDY8	MALT1	0.41
Q9UKY7	CDV3	0.475
Q9ULC4	MCTS1	0.481
Q9ULX3	NOB1	0.493
Q9UM54	MYO6	0.474
Q9UNI6	DUSP12	0.447
Q9Y3D8	AK6	0.496
Q9Y4K4	MAP4K5	0.45

P60891	PRPS1	[illegible]
P61221	ABCE1	[illegible]
P61758	VBP1	[illegible]
P62253	UBE2G1	[illegible]
P62495	ETF1	[illegible]
P62857	RPS28	[illegible]
P62861	FAU	[illegible]
P62937	PPIA	[illegible]
P62987	UBA52	[illegible]
P63241	EIF5A	[illegible]
P68104	EEF1A1	[illegible]
Q02790	FKBP4	[illegible]
Q04637	EIF4G1	[illegible]
Q05D60	DEUP1	[illegible]
Q06203	PPAT	[illegible]
Q08752	PPID	[illegible]
Q13085	ACACA	[illegible]
Q13112	CHAF1B	[illegible]
Q13257	MAD2L1	[illegible]
Q13451	FKBP5	[illegible]
Q13542	EIF4EBP2	[illegible]
Q13951	CBFB	[illegible]
Q14353	GAMT	[illegible]

Q9Y	PCL	0.45
6V0	O	1

Q144	SLB	[illegible]
93	P	
Q150	NCA	[illegible]
03	PH	
Q150	NCA	[illegible]
21	PD2	
Q150	POL	[illegible]
54	D3	
Q151	PTG	[illegible]
85	ES3	
Q156	TRIP	[illegible]
45	13	
Q157	TOM	[illegible]
85	M34	
Q162	UAP	[illegible]
22	1	
Q165	CDC	[illegible]
43	37	
Q167	UBE	[illegible]
63	2S	
Q2N	ERC	[illegible]
KX8	C6L	
Q3K	ALK	[illegible]
RA9	BH6	
Q49	ZFP6	[illegible]
AA0	9	
Q53	HIK	[illegible]
FT3	ESHI	
Q6FI	CIA	[illegible]
81	PIN1	
Q6IB	NCA	[illegible]
W4	PH2	
Q6PI	FIG	[illegible]
W4	NL1	
Q6P	ATA	[illegible]
L18	D2	
Q6SJ	FAM	[illegible]
93	111B	
Q71	CEN	[illegible]
F23	PU	
Q7L	DPH	[illegible]
8W6	6	
Q7Z	RAB	[illegible]
6M1	EPK	
Q7Z	UBE	[illegible]
7E8	2Q1	
Q86	SAP	[illegible]
UD0	CD2	
Q86	CCD	[illegible]
WR0	C25	
Q86	DNA	[illegible]
Y56	AF5	

Q8I V50	LYS MD2	[illegible]
Q8I X90	SKA 3	[illegible]
Q8N 2Z9	CEN PS	[illegible]
Q8N 5I9	C12o rf45	[illegible]
Q8N 7N1	FAM 86B1	[illegible]
Q8N BA8	DT WD2	[illegible]
Q8N BT2	SPC 24	[illegible]
Q8N EM2	SHC BP1	[illegible]
Q8T BB5	KLH DC4	[illegible]
Q8T CA0	LRR C20	[illegible]
Q8T CG1	CIP2 A	[illegible]
Q8T CY9	URG CP	[illegible]
Q8W UX2	CHA C2	[illegible]
Q8W VJ2	NUD CD2	[illegible]
Q8W WK9	CKA P2	[illegible]
Q8W XX5	DNA JC9	[illegible]
Q926 64	GTF 3A	[illegible]
Q929 90	GLM N	[illegible]
Q930 45	STM N2	[illegible]
Q96 BN8	OTU LIN	[illegible]
Q96 BR1	SGK 3	[illegible]
Q96 C90	PPP1 R14 B	[illegible]
Q96 DG6	CMB L	[illegible]
Q96 GA3	LTV 1	[illegible]
Q96 GX5	MAS TL	[illegible]

Q96KB5	PBK	[illegible]
Q96P70	IPO9	[illegible]
Q96R06	SPAG5	[illegible]
Q96SB4	SRPK1	[illegible]
Q96T88	UHRF1	[illegible]
Q99615	DNAJC7	[illegible]
Q99618	CDCA3	[illegible]
Q99622	C12orf57	[illegible]
Q99661	KIF2C	[illegible]
Q9BPX3	NCAPG	[illegible]
Q9BPZ3	PAIP2	[illegible]
Q9BQL6	FERMT1	[illegible]
Q9BRT9	GINS4	[illegible]
Q9BRX5	GINS3	[illegible]
Q9BTE3	MCMBP	[illegible]
Q9BV57	ADI1	[illegible]
Q9BW04	SARG	[illegible]
Q9BW71	HIRIP3	[illegible]
Q9BXJ9	NAA15	[illegible]
Q9BXS6	NUSAP1	[illegible]
Q9BXW9	FANCD2	[illegible]
Q9BZD4	NUF2	[illegible]
Q9BZX2	UCK2	[illegible]
Q9GZZ1	NAA50	[illegible]
Q9H2J4	PDCL3	[illegible]
Q9H2P9	DPH5	[illegible]

Q9H446	RWDD1	[illegible]
Q9H467	CUEDC2	[illegible]
Q9H4H8	FAM83D	[illegible]
Q9H900	ZWILCH	[illegible]
Q9H999	PANK3	[illegible]
Q9H9S4	CAB39L	[illegible]
Q9HAN9	NMNAT1	[illegible]
Q9HB71	CACYBP	[illegible]
Q9NP74	PALMD	[illegible]
Q9NQW6	ANLN	[illegible]
Q9NS91	RAD18	[illegible]
Q9NSA3	CTNNBIP1	[illegible]
Q9NTJ3	SMC4	[illegible]
Q9NTK5	OLA1	[illegible]
Q9NVQ4	FAIM	[illegible]
Q9NX55	HYPK	[illegible]
Q9NZJ0	DTL	[illegible]
Q9UBB4	ATXN10	[illegible]
Q9UEW8	STK39	[illegible]
Q9UHD1	CHORDC1	[illegible]
Q9UHV9	PFDN2	[illegible]
Q9UHW5	GPN3	[illegible]
Q9UI26	IPO11	[illegible]
Q9UK76	JPT1	[illegible]

Q9U LW0	TPX 2	[illegible]
Q9U NS1	TIM ELE SS	[illegible]
Q9U NY4	TTF 2	[illegible]
Q9U Q80	PA2 G4	[illegible]
Q9Y 248	GIN S2	[illegible]
Q9Y 266	NUD C	[illegible]
Q9Y 2Y0	ARL 2BP	[illegible]
Q9Y 2Y1	POL R3K	[illegible]
Q9Y 316	ME MO1	[illegible]
Q9Y 3F4	STR AP	[illegible]
Q9Y 448	KNS TRN	[illegible]
Q9Y 4F9	RIP OR2	[illegible]
Q9Y 4P1	ATG 4B	[illegible]
Q9Y 5P4	COL 4A3 BP	[illegible]
Q9Y 6A5	TAC C3	[illegible]
Q9Y 6E2	BZW 2	[illegible]

Data 2 Venn diagram of downregulated proteins

Drug	Target
Hyaluronic acid	HABP2 ,HMMR
Iron	FEN1
Ferrous gluconate	FEN1
Ferrous succinate	FEN1
Ferrous ascorbate	FEN1
Ferrous fumarate	FEN1
Ferrous glycine sulfate	FEN1
NADH	LDHB, AKR1C

3,DHF
R,HAD
HA,HS
D11B2,
ALDH5
A1,HIB
ADH,A
KR1C1,
MTHF
D1,LD
HA,IM
PDH1,
ALDH3
A2,AA
SS,ND
UFB8,
ALDH2
,NDUF
V3,ND
UFA13,
NDUFA
4,NDU
FA11,N
DUFB9
,NDUF
S3,ND
UFS6,N
DUFA5
,HADH
,NDUF
C1,GL
UD1,N
DUFS8,
NDUF
B3,ND
UFB7,T
STA3,A
LDH6A
1,NDU
FA10,N
DUFS4,
NDUFA
3,AKR1
B1,MS
MO1,L
DHC,U
GDH,O
GDH,B
LVRB,
MT-
ND6

Stiripentol	LDHB, LDHA
Cisplatin	ATOX1
Sucrose	ATOX1
Dacarbazine	POLA2
Isoprenaline	PIK3R3
Ascorbic acid	ALKBH2,PHYH,PLOD3,P4HTM
Pyrimethamine	HEXB, DHFR
Indometacin	AKR1C3
Bimatoprost	AKR1C3
Rutin	AKR1C3
Flufenamic Acid	AKR1C3
Copper	GPI,PKM,B2M,PTGES3,CFL1, EEF1A1,PPIA, EIF4A1,CLU,LDHA,RANBP1,GSN,PRDX2, HSPA5, APOD, STIP1,PRDX1, ENO1, HIST1H1E,GOT1,UGDH
Fostamatinib	TTK,CDK1,CHEK1,STK39, MYLK, ERN1, MAP2K6,NUAK2,GSK3A,

	CTSS,P KMYT 1,AUR KA,AU RKB,L RRK2,S GK3,C AMKK 2,RIOK 2,RPS6 KA1,M AP2K3, STK10, MAP4 K5,EPH A7,HC K,FAA H,PRK ACA,M INK1,L IMK1, MAP3 K1,NE K3,JAK 1,CAM K2D,T NIK
Mifepristone	KLK3
Cyproterone acetate	KLK3
Bleomycin	LIG1
Alpha-Linolenic Acid	FADS2
Carfilzomib	PSMB9
Porfimer sodium	LDLR
Histidine	HARS
Interferon gamma-1b	IFNGR 1
L-Aspartic Acid	ASNS,P AICS,A DSS,G OT1
Glutamic Acid	ASNS, AASS,S LC7A1 1,CPQ, ABAT, GLUD1 ,PFAS, GOT1,F OLH1, GLS2,O

	PLAH, GLUL
Asparagine	ASNS
Proline	PPIC,PPIA
Trimethoprim	DHFR, TYMS
Methotrexate	DHFR
Pemetrexed	DHFR, TYMS, GART
Gentamicin	DHFR
Proguanil	DHFR
Trimetrexate	DHFR
Pralatrexate	DHFR, TYMS
L-Arginine	NOS3, ARG2, ASL
L-Citrulline	NOS3
Sapropterin	NOS3
Miconazole	NOS3
Apremilast	NOS3
Pyruvic acid	PKM,ABAT,SLC16A3
Adenine	PECR
Isopropyl alcohol	ADI1
Hydrocortisone	HSD11B2,ANXA1
Fluoxymesterone	HSD11B2
Hydrocortisone acetate	HSD11B2,ANXA1
Hydrocortisone butyrate	HSD11B2,ANXA1
Hydrocortisone cypionate	HSD11B2,ANXA1
Hydrocortisone phosphate	HSD11B2,ANXA1
Hydrocortisone probutate	HSD11B2,ANXA1
Hydrocortisone valerate	HSD11B2,ANXA1

Pyridoxal phosphate	SHMT1 ,ABAT, GOT1, KYAT1, SHMT2 ,SDSL, CTH
Glycine	SHMT1 ,BAAT, GNMT, SHMT2 ,GLYAT L1
Carglumic acid	CPS1
Calcium Citrate	NUCB2 ,S100A 13,TPT 1,CIB1
Calcium Phosphate	NUCB2 ,S100A 13,TPT 1,CIB1
Calcium phosphate dihydrate	NUCB2 ,S100A 13,TPT 1,CIB1
Rifampicin	SLCO1 B1
Clarithromycin	SLCO1 B1
Telaprevir	SLCO1 B1
Iopodic acid	DIO3
Denileukin diftitox	IL2RB
Aldesleukin	IL2RB
Basiliximab	IL2RB
Oseltamivir	NEU1, CES1
Orlistat	FASN
Cladribine	RRM1, RRM2, POLE4, PNP
Gemcitabine	RRM1, TYMS
Clofarabine	RRM1
Hydroxyurea	RRM1
Fludarabine	RRM1, DCK
Gallium nitrate	RRM2
Insulin Pork	CTSD

Esketamine	EEF2
Moxetumomab Pasudotox	EEF2
Liothyronine	PCNA
Azacitidine	DNMT 1
Procainamide	DNMT 1
Flucytosine	DNMT 1
Decitabine	DNMT 1
Zinc	EEF1A 1,CLU, PSPH,G SN,PR DX1,E NO1,F N1,HD AC4,M DM2
Zinc acetate	EEF1A 1,CLU, PSPH,G SN,PR DX1,E NO1,F N1,HD AC4,M DM2
Zinc chloride	EEF1A 1,CLU, PSPH,G SN,PR DX1,E NO1,F N1,HD AC4,M DM2
Glutathione	GSTK1, MGST3 ,GPX1, AKR1B 1,GSTT 1,GPX4 ,GPX8
Ciclosporin	PPIA
Antihemophilic factor	PHYH, LMAN 1,F10,H SPA5

human recombinant	PHYH, LMAN 1,F10,H SPA5
Lonoctocog alfa	PHYH, LMAN 1,F10,H SPA5
Moroctocog alfa	PHYH, LMAN 1,F10,H SPA5
Moxifloxacin	TOP2A
Amsacrine	TOP2A
Dexrazoxane	TOP2A
Valrubicin	TOP2A
Teniposide	TOP2A
Epirubicin	TOP2A
Enoxacin	TOP2A
Pefloxacin	TOP2A
Ciprofloxacin	TOP2A
Trovafloxacin	TOP2A
Daunorubicin	TOP2A
Etoposide	TOP2A
Dactinomycin	TOP2A
Lomefloxacin	TOP2A
Doxorubicin	TOP2A
Norfloxacin	TOP2A
Levofloxacin	TOP2A
Ofloxacin	TOP2A
Idarubicin	TOP2A
Podofilox	TOP2A
Mitoxantrone	TOP2A
Sparfloxacin	TOP2A
Finafloxacin	TOP2A
Azathioprine	HPRT1
Mercaptopurine	HPRT1, PPAT,I MPDH 1
5-O-phosphono-alpha-D-ribofuranosyl diphosphate	HPRT1
Creatine	CKB,G AMT
Succinic acid	ALDH5 A1,SU CLG2,P LOD3, OXCT1

Valproic Acid	ALDH5 A1,AC ADSB, ABAT, OGDH
Chlormerodrin	ALDH5 A1
Auranofin	PRDX5
Benzoic Acid	PRDX5
Dalfampridine	KCNB2
Adenosine phosphate	ACSL1, ACSS2
Citric Acid	UCK2, GNMT, AKR1B 1
Salicylic acid	AKR1C 1
Acetylsalicylic acid	AKR1C 1,HSPA 5
Raltitrexed	TYMS
Floxuridine	TYMS
Trifluridine	TYMS
Fluorouracil	TYMS
Capecitabine	TYMS
Tegafur	TYMS
Tegafur-uracil	TYMS
Vinblastine	TUBG1
Propylene glycol	F13A1
Thrombin	F13A1
Prothrombin	F13A1
Human Thrombin	F13A1
Thrombin Alfa	F13A1
Anti-inhibitor coagulant complex	F13A1, F10
Trimetazidine	ACAA1
Phosphatidyl serine	PISD,D GKD
Bosutinib	CDK2, HCK
L-Glutamine	PPAT,C TPS1,G LUL
Dasatinib	PPAT
Vitamin A	DHRS3 ,APOD, RBP1
Levocarnitine	SLC25 A29,CE

	S1,CROT,CPT2
Nicotinamide	LDHA
Urokinase	NID1
Amphetamine	ADRA2A,MAOA,MAOB,MAOB
Bethanidine	ADRA2A
Ziprasidone	ADRA2A
Cabergoline	ADRA2A
Ropinirole	ADRA2A
Dihydroergotamine	ADRA2A
Amitriptyline	ADRA2A
Olanzapine	ADRA2A
Clozapine	ADRA2A
Norepinephrine	ADRA2A
Mirtazapine	ADRA2A
Phenylpropanolamine	ADRA2A
Loxapine	ADRA2A
Pramipexole	ADRA2A
Dipivefrin	ADRA2A
Prazosin	ADRA2A
Chlorpromazine	ADRA2A
Brimonidine	ADRA2A
Nortriptyline	ADRA2A,PGRMC1
Amoxapine	ADRA2A,ADRA2A
Lamotrigine	ADRA2A

Clonidine	ADRA2A
Lisuride	ADRA2A
Guanabenz	ADRA2A
Dexmedetomidine	ADRA2A
Trazodone	ADRA2A
Epinephrine	ADRA2A
Phentolamine	ADRA2A
Ergotamine	ADRA2A
Tizanidine	ADRA2A
Apomorphine	ADRA2A
Trimipramine	ADRA2A
Risperidone	ADRA2A
Epinastine	ADRA2A
Tolazoline	ADRA2A
Fenoldopam	ADRA2A
Pseudoephedrine	ADRA2A,ATF3
Benzphetamine	ADRA2A
Phenoxybenzamine	ADRA2A
Maprotiline	ADRA2A
Oxymetazoline	ADRA2A
Apraclonidine	ADRA2A
Methyldopa	ADRA2A
Guanfacine	ADRA2A
Ergoloid mesylate	ADRA2A
Carvedilol	ADRA2A
Doxepin	ADRA2A

Nefazodone	ADRA2A
Desipramine	ADRA2A
Pergolide	ADRA2A
Bromocriptine	ADRA2A
Quetiapine	ADRA2A
Aripiprazole	ADRA2A
Paliperidone	ADRA2A
Mephentermine	ADRA2A
Yohimbine	ADRA2A
Methotrimeprazine	ADRA2A
Metamfetamine	ADRA2A,MAOA,MAOB
Periciazine	ADRA2A
Zuclopenthixol	ADRA2A
Celiprolol	ADRA2A
Dronedarone	ADRA2A
Lofexidine	ADRA2A
Mianserin	ADRA2A
Pizotifen	ADRA2A
Asenapine	ADRA2A
Droxidopa	ADRA2A
Xylometazoline	ADRA2A
Levonordefrin	ADRA2A
Naphazoline	ADRA2A
Lurasidone	ADRA2A
Dosulepin	ADRA2A

Lorpiprazole	ADRA2A
Moxisylyte	ADRA2A
Moxonidine	ADRA2A
Racepinephrine	ADRA2A
Dihydroergocornine	ADRA2A
DL-Methylephedrine	ADRA2A,ADRA2A
Indigotindisulfonic Acid	ADRA2A
Rilmenidine	ADRA2A
Dihydroergocristine	ADRA2A
Aripiprazole lauroxil	ADRA2A
Fluoxetine	CKS1B
Mycophenolate mofetil	IMPDH1
Ribavirin	IMPDH1
Mycophenolic acid	IMPDH1
Pamidronic acid	FDPS
Zoledronic acid	FDPS
Alendronic acid	FDPS
Ibandronate	FDPS
Risedronic acid	FDPS
Coagulation factor VIIa Recombinant Human	F10
Coagulation Factor IX (Recombinant)	F10
Menadione	F10
Fondaparinux	F10
Heparin	F10
Enoxaparin	F10
Rivaroxaban	F10
Apixaban	F10
Edoxaban	F10
Turoctocog alfa	F10
Protamine sulfate	F10
Bemiparin	F10
Kappadione	F10

Antithrombin Alfa	F10
Betrixaban	F10
Protein S human	F10
Coagulation factor VII human	F10
Coagulation Factor IX Human	F10
Antihemophilic factor human	F10
Albutrepenonacog alfa	F10
Emicizumab	F10
Nonacog beta pegol	F10
Turoctocog alfa pegol	F10
Biotin	ACACA,PCCA,PCCB,MCCC1
Olopatadine	S100A13
Amlexanox	S100A13
Calcium Chloride	S100A13
Calcium	S100A13,COMP
Guanidine	GAMT,ALDH2
Lithium citrate	IMPA2
Lithium carbonate	IMPA2
Flavin adenine dinucleotide	ACOX1,MAOA,ACADM,ACAD8,MAOB,FDXR
Cystine	SLC7A11
Riluzole	SLC7A11
Sulfasalazine	SLC7A11,ACAT1
Acetylcysteine	SLC7A11

Thimerosal	SLC7A11
Hesperidin	AURKB
L-Threonine	TARS
L-Valine	PCCB
Chloramphenicol	CD55
Cholic Acid	MT-CO2,CES1,COX6C,COX7C, COX6B1
Ornithine	ARG2
Disulfiram	ALDH2
Caffeine	PDE8A
Clodronic acid	SLC25A4
Phentermine	MAOA, MAOB
Procaine	MAOA, MAOB
Tranylcypromine	MAOA, MAOB
Phenelzine	MAOA, ABAT, MAOB
Minaprine	MAOA
Zonisamide	MAOA, MAOB, CA2,CA12
Selegiline	MAOA, MAOB
Procarbazine	MAOA, MAOB
Moclobemide	MAOA, MAOA, MAOB, MAOB
Isocarboxazid	MAOA, MAOB
Pargyline	MAOA, MAOB
Nialamide	MAOA, MAOB
Nomifensine	MAOA, MAOB
Zimelidine	MAOA, MAOB
Tacrine	CES1

Meperidine	CES1
Dextropropoxyphene	CES1
Cocaine	CES1
Naloxone	CES1
Diamorphine	CES1
Probucol	CES1
Cyclandelate	CES1
Ubidecarenone	NDUFV3
Diacerein	NR1H3, CYP2D6,NR1H2
Didanosine	PNP
Isoleucine	ACADSB
Amcinonide	ANXA1
Fluocinolone acetonide	ANXA1
Dexamethasone	ANXA1
L-Alanine	ABAT,SLC1A4
Vigabatrin	ABAT
Ademetionine	GNMT
Minocycline	CYCS
Nitroprusside	NPR1
Nitroglycerin	NPR1
Isosorbide dinitrate	NPR1
Amyl Nitrite	NPR1
Erythrityl tetranitrate	NPR1
Nesiritide	NPR1
Omega-3-carboxylic acids	HADH
Melatonin	EPX
Hexachlorophene	GLUD1
Aluminum chloride	GLUD1
Mitotane	FDX1
Pioglitazone	MAOB
Rasagiline	MAOB
Safinamide	MAOB
Sacubitril	MME
L-Cysteine	GOT1, CTH
Methionine	METAP2

D-Methionine	METAP2
Sipuleucel-T	ACPP
Fomepizole	CAT
Perhexiline	CPT2
Isoniazid	CYP2C8
Palmitic Acid	CYP2C8
Cyanocobalamin	MMAA
Hydroxocobalamin	MMAA
Sulindac	AKR1B1
Spironolactone	SRD5A3
Palifermin	NRP1, HSPG2
Pegaptanib	NRP1
Silver	MT1F, MT1X
Methyclothiazide	CA2
Topiramate	CA2
Sulpiride	CA2
Bendroflumethiazide	CA2
Celecoxib	CA2
Benzthiazide	CA2,CA12
Valdecoxib	CA2
Cyclothiazide	CA2
Furosemide	CA2
Methazolamide	CA2
Hydroflumethiazide	CA2,CA12
Acetazolamide	CA2,CA12
Dorzolamide	CA2
Chlorothiazide	CA2
Hydrochlorothiazide	CA2
Trichlormethiazide	CA2
Ethinamate	CA2
Diazoxide	CA2
Diclofenamide	CA2
Brinzolamide	CA2
Quinethazone	CA2
Urea	CA2
Sodium carbonate	CA2
Sodium sulfate	CA2

Acitretin	RBP1
Alitretinoin	IGFBP3
Mecasermin	IGFBP3
Diethylstilbestrol	ESRRA
Flavone	ESRRA
Acetaminophen	FAAH
Thiopental	FAAH
Dabrafenib	LIMK1
Cromoglicic acid	S100P
Binimetinib	MAP3K1
Ocriplasmin	FN1
Ruxolitinib	JAK1
Tofacitinib	JAK1
Baricitinib	JAK1
Xanthinol	OGDH
Amiloride	SLC9A1
Dextromethorphan	PGRMC1
Sertraline	PGRMC1
Capromab pendetide	FOLH1
Catumaxomab	EPCAM
Technetium Tc-99m nofetumomab merpentan	EPCAM
Hypromellose	EPCAM
Belinostat	HDAC4
Romidepsin	HDAC4
Panobinostat	HDAC4
Ouabain	ATP1A3
Tipiracil	TYMP
Riboflavin	BLVRB
Flavin mononucleotide	BLVRB

Data 3 The 251 matched proteins corresponded to 407 drugs

Gene name
RBP1
TOP2A
CA2
RRM1
TYMS

PCCB
ADRA2A
ESRRA
F10
CYP2C8
ALDH5A
1
SUCLG2
PLOD3
OXCT1
HSD11B2
ANXA1
FDX1
DHFR
IMPDH1
MAOB
ACADSB
ABAT
SLC1A4
TTK
CDK1
CHEK1
STK39
MYLK
ERN1
MAP2K6
NUAK2
GSK3A
CTSS
PKMYT1
AURKA
AURKB
LRRK2
SGK3
CAMKK2
RIOK2
RPS6KA1
MAP2K3
STK10
MAP4K5
EPHA7
HCK
FAAH
PRKACA
MINK1
LIMK1
MAP3K1
NEK3
JAK1
CAMK2D

TNIK
AKR1C3
ACAA1
GNMT
MAOA
HPRT1
PSMB9
CKB
GAMT
MT-CO2
CES1
COX6C
COX7C
COX6B1
EPX
OGDH
HDAC4
GSTK1
MGST3
GPX1
AKR1B1
GSTT1
GPX4
GPX8
AKR1C1
NPR1
CAT
ACACA
PCCA
MCCC1
CA12
NOS3
ARG2
ASL
SLC7A11
ACAT1
PPAT
CTPS1
GLUL
DCK
CD55
GART
PRDX5
KLK3
SLCO1B1
LDHA
POLA2
HABP2
HMMR
CDK2

LDHB
PNP
UCK2
SHMT1
GOT1
KYAT1
SHMT2
SDSL
CTH
CPS1
GLUD1
HEXB
RRM2
POLE4
FASN
ASNS
PAICS
ADSS
SRD5A3
BAAT
GLYATL1
DNMT1
DHRS3
APOD
S100A13
ALDH2
NR1H3
CYP2D6
NR1H2
TARS
ATP1A3
IMPA2

Data 4 The 136 matched proteins corresponded to 164 drugs
